# Synthesis of α,α‐Difluoroketones via Zinc‐Mediated Cross‐Electrophile Coupling of *gem*‐Difluoroenol Sulfonates and *N*‐(acyloxy)phthalimides

**DOI:** 10.1002/open.70285

**Published:** 2026-08-20

**Authors:** Jing Zhao, Li Yang, Xiaohong Wang, Baojian Xiong, Xinmin Li, Huan Yang

**Affiliations:** ^1^ School of Pharmacy Guizhou International Science & Technology Cooperation Base of Medical Optical Theranostics Research Zunyi Medical University Zunyi Guizhou China

**Keywords:** α,α‐difluoroketones, cross‐electrophile coupling, *gem*‐difluoroenol sulfonates, *N*‐(acyloxy)phthalimides, zinc‐mediated

## Abstract

A zinc‐mediated catalyst‐free cross‐electrophile coupling of *gem*‐difluoroenol sulfonates with *N*‐(acyloxy)phthalimides is developed. It efficiently affords diverse α,α‐difluoroketones with broad radical compatibility, good gram‐scale scalability, and functional group tolerance, enabling access to complex amino acid and glycosyl derivatives.

## Graphical abstract

A zinc‐mediated catalyst‐free cross‐electrophile couplingbetween *gem*‐difluoroenol sulfonates and *N*‐(acyloxy) phthalimides accesses structurally diverse α,α‐difluoroketones. It exhibits broad radical compatibility, reliable gram‐scale performance, and enables construction of elaborate fluorinated amino acid and glycosyl derivatives.

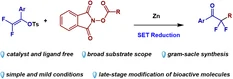

## 1 Introduction

**SCHEME 1:**
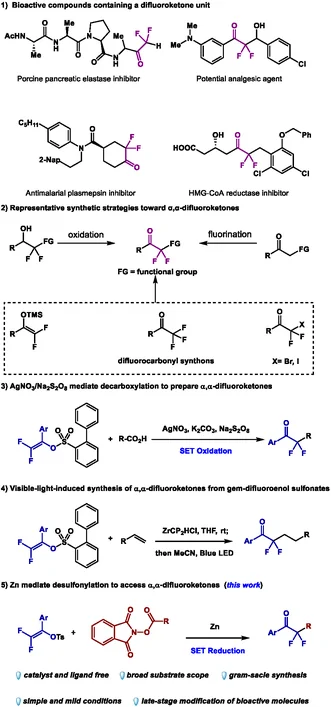
Bioactive molecules containing the—difluoroketone motif and synthetic approaches to—difluoroketones from *gem*‐difluoroenol sulfonates.

The selective incorporation of fluorinated groups into organic molecules is of great importance, as it often has a beneficial impact on the properties of bioactive molecules [1, 2, 3]. Among various organofluorine compounds, α,α‐difluoroketones are valuable, serving not only as key intermediates in the synthesis of fluorinated compounds [4, 5, 6, 7] but also holding significant potential in medicinal chemistry [8, 9, 10, 11, 12]. Bioactive molecules containing the difluoroketone unit exhibit a wide range of biological activities, particularly finding extensive applications as enzyme inhibitors (Scheme 1‐1). Consequently, developing efficient synthetic methods for α,α‐difluoroketones has attracted significant attention in synthetic and medicinal research [13].

**SCHEME 2:**
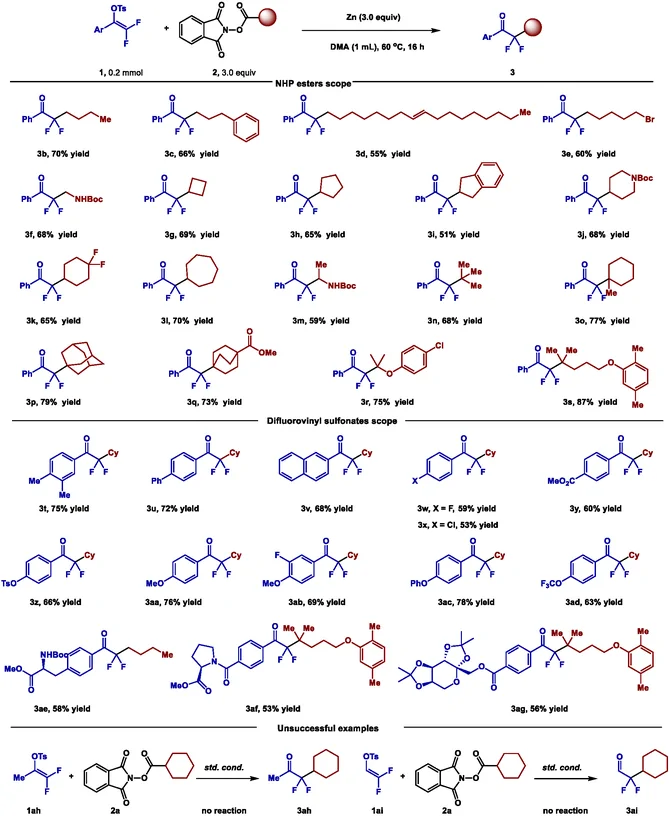
Substrate scope. Reaction conditions: **1** (0.2 mmol, 1.0 equiv.), **2** (0.6 mmol, 3.0 equiv.), Zn (0.6 mmol, 3.0 equiv.), DMA (1.0 mL), Ar, 60 °C, 16 h, the yield is the isolated yield based on **1.**

Traditionally, difluoroketone units are generated through ketone fluorination [14, 15, 16, 17, 18] or α,α‐difluoroalcohol oxidation (Scheme 1‐2) [19, 20, 21]. These methods are efficient but often suffer from poor selectivity, harsh conditions, and limited substrate scope. Recent advances in the accessibility of difluorocarbonyl synthons [22, 23, 24], such as difluoroenol silyl ethers, difluorocarbonyl halides, and trifluoroketones have established these compounds as valuable precursors for synthesizing α,α‐difluoroketones. Among these developments, *gem*‐difluoroenol sulfonates have emerged as privileged fluorinated building blocks in organic synthesis. These versatile synthons can be efficiently prepared from readily available aldehydes [25] or aryl halides [26], offering both synthetic reliability and remarkable structural diversity [27]. Their unique reactivity profile and structural tunability enable exceptional performance in transition‐metal‐catalyzed cross‐coupling reactions [28, 29, 30, 31, 32, 33]. However, such synthons have relatively limited applications in the synthesis of α,α‐difluoroketones.

**SCHEME 3:**
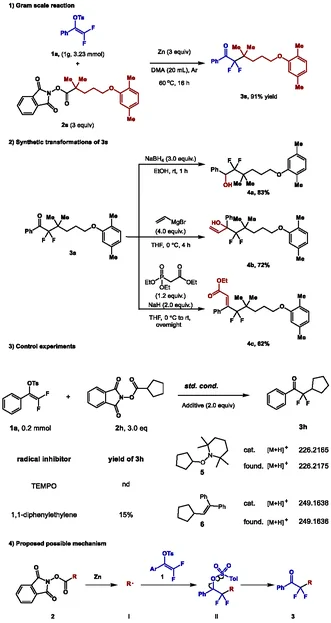
(1) Gram‐scale reaction, (2) synthetic transformations of **3s**, (3) control experiments, and (4) proposed possible mechanism.

Recently, the Feng group was the first to use *gem*‐difluoroenol sulfonates to synthesize α,α‐difluoroketones via silver‐promoted decarboxylative coupling with carboxylic acids (Scheme 1‐3) [34]. Subsequently, the Yue group developed a visible‐light‐induced radical substitution of alkylzirconium species with *gem*‐difluoroenol sulfonates, providing a mild and photocatalyst‐free approach to α,α‐difluoroketones (Scheme 1‐4) [35]. Despite these advances, cross‐electrophile coupling represents an attractive alternative, as it generally features broad functional group tolerance under mild reaction conditions [36, 37, 38, 39, 40]. Herein, we developed a zinc‐mediated cross‐electrophile coupling between *N*‐(acyloxy)phthalimides and *gem*‐difluoroenol sulfonates for constructing structurally diverse α,α‐difluorinated ketones (Scheme 1‐5). This protocol not only features operational simplicity and good functional group tolerance but also overcomes the limitation of the narrow substrate scope of *gem*‐difluoroenol sulfonates [34, 35]. Its practical utility has been demonstrated through successful gram‐scale synthesis and diverse product derivatization.

## 2 Results and Discussion

Our initial studies focused on the reaction between *gem*‐difluoroenol sulfonate **1a** and *N*‐(acyloxy)phthalimide **2a**. After thorough screening of reaction parameters, the optimal conditions were identified as follows: a 1:3:3 molar ratio of **1a**, **2a**, and Zn in DMA under Ar at 60 °C for 16 h, affording **3a** in 82% isolated yield (Table 1, entry 1). Solvent evaluation revealed that replacing DMA with MeCN,DMF, DMSO, or THF significantly diminished the yield of **3a** (entries 2–5). Furthermore, screening of alternative reductants (Mg, Fe, Mn) revealed either negligible or no activity in this cross‐coupling reaction (entries 6–8). Reducing the amounts of **2a** and Zn also adversely affected the yield (entries 9–10). Temperature optimization confirmed 60 °C as the ideal reaction temperature (entries 11–12). Notably, the reaction proceeded smoothly under ambient air (entry 13), with only a marginal decrease in product yield.

**TABLE 1:** Optimization of reaction conditions.^a^

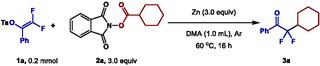		
Entry	Deviation from std. conditions	Yield of **3a** b
1	None	82%
2	MeCN instead of DMA	19%
3	DMF instead of DMA	68%
4	DMSO instead of DMA	15%
5	THF instead of DMA	Trace
6	Mg instead of Zn	nd
7	Fe instead of Zn	nd
8	Mn instead of Zn	Trace
9	1 equiv. of **2a** and 1 equiv. Zn	45%
10	2 equiv. of **2a** and 2 equiv. Zn	66%
11	40 °C instead of 60 °C	50%
12	r.t. instead of 60 °C	Trace
13	Under air	76%

a Reaction conditions: **1a** (0.2 mmol, 1.0 equiv.), **2a** (0.6 mmol, 3 equiv.), Zn (0.6 mmol, 3 equiv.), DMA (1.0 mL), Ar, 60 °C, 16 h.

b Isolated yield based on **1a**.

With the optimized reaction conditions in hand, we next sought to explore the substrate scope of NHP esters (Scheme 2). We first evaluated the reactivity of simple primary alkyl carboxylic esters. The results showed that these esters were compatible with the reaction conditions, providing the desired products in moderate yields (**3b**–**3d**). Furthermore, substrates containing primary alkyl bromide or amide functional groups also underwent the reaction efficiently, leading to the successful formation of products **3e** and **3f** with smooth conversions. Next, we turned our attention to secondary alkyl redox esters, which exhibited a wide range of structural diversity. A variety of cyclic reagents with differing ring sizes were well tolerated, and the reactions proceeded to afford the corresponding products in good yields (**3g**–**3l**). Additionally, an acyclic substrate containing an acidic proton was also examined, and it participated in the reaction with good efficiency, affording **3m** in 59% yield. Encouraged by the successful transformations of primary and secondary alkyl esters, we proceeded to investigate the cross‐coupling of more sterically hindered tertiary alkyl carboxylic esters. Remarkably, all of these sterically challenging substrates underwent the reaction with high efficiency, resulting in the formation of the corresponding products (**3n**–**3s**) in good yields ranging from 68% to 87%.

Finally, the substrate scope of this reaction was explored using the *gem*‐difluoroenol sulfonates to further evaluate the generality of this transformation. A range of *gem*‐difluoroenol sulfonates reacted efficiently with cyclohexanecarboxylic acid‐derived ester **2a**, delivering the corresponding α,α‐difluoroketones. Modest to good yields were obtained when the substrates contained alkyl groups (**3t**), as well as biphenyl and naphthalene derivatives (**3u**–**3v**). Substrates containing halogens exhibited reduced reaction efficiency (**3w**–**3x**). The ester and sulfonate groups were compatible in the reaction, yielding the desired products **3y** and **3z**. The methoxy, phenoxy, and trifluoromethoxy substituted substrates were smoothly transformed into the target products (**3aa**–**3ad**) in 63%–78% yields. It is noteworthy that substrates derived from amino acids, such as *L*‐phenylalanine and *D*‐proline, underwent transformation with acceptable yields using this protocol (**3ae**–**3af**). Furthermore, a fructose‐containing reagent reacted efficiently with a drug molecule (gemfibrozil)‐derived ester, producing product **3ag** in 56% yield. Unfortunately, the reaction was effective primarily for aryl *gem*‐difluoroenol sulfonates, whereas alkyl‐ and hydrogen‐substituted analogs (**1ah**, **1ai**) remained unreactive under the optimized conditions. This lack of reactivity was likely attributable to the inability of these substrates to stabilize the radical intermediates via conjugation, in contrast to their aryl‐substituted counterparts.

To validate the robustness of this protocol, a gram‐scale reaction between **1a** and **2s** was performed. To our satisfaction, the reaction proceeded smoothly under standard conditions, yielding **3s** in an improved yield compared to the 0.2 mmol‐scale reaction (Scheme 3‐1). The practical utility of this method is further highlighted in Scheme 3‐2, which demonstrates the potential for diverse derivatizations of **3s**.

As anticipated, the difluoroketone moiety serves as a versatile divergence point, enabling the synthesis of a variety of structurally distinct fluorinated compounds. The benzoyl group was easily reduced by NaBH_4_, affording **4a** in high yield. A reaction with vinylmagnesium bromide resulted in the formation of alcohol **4b**. Additionally, ethyl 2‐ (diethoxyphosphoryl)acetate was employed to derivatize an alkenyl ester, delivering **4c** in good yield under basic condition.

To better understand the reaction mechanism, radical trapping experiments were conducted (Scheme 3‐3). The addition of TEMPO under standard conditions completely suppressed the reaction, and the TEMPO adduct **5** was detected by HRMS. Furthermore, the addition of 1,1‐diphenylethylene under standard conditions also led to the detection of compound **6**. These findings strongly suggest that the reaction likely proceeds through a radical pathway. Based on the experimental results and reported literature [41, 42, 43], a possible reaction mechanism is outlined in Scheme 3‐4. The process begins with a single‐electron transfer from Zn to *N*‐(acyloxy)phthalimide, leading to its fragmentation and the generation of an alkyl radical (**I**). This radical subsequently undergoes a radical addition on the *gem*‐difluoroenol sulfonates, forming the key radical intermediate (**II**) [34, 35]. Intermediate **II** then undergoes β‐fragmentation, resulting in the formation of the desired products.

## 3 Conclusion

In summary, we have developed an efficient zinc‐mediated cross‐electrophile coupling for the synthesis of α,α‐difluoroketones under mild conditions. This protocol demonstrates broad functional group tolerance toward various *gem*‐difluoroenol sulfonates and *N*‐(acyloxy)phthalimides, including primary, secondary, and tertiary alkyl radical precursors. Moreover, gram‐scale scalability and successful downstream transformations highlight its utility in synthetic application.

## Funding

This study was supported by the Guizhou Provincial Science and Technology Department 2026 Basic Research Program (Natural Sciences) Youth Guidance Project (QKHJC [2026] 002‐54), the National Natural Science Foundation of China (Grant 22267025) and the Science and Technology Innovation Team of Higher Education of Guizhou Provincial Education Department (Grant Qianjiaoji[2023J073).

## Conflicts of Interest

The authors declare no conflicts of interest.

## Supporting information

Supplementary Material

## Data Availability

The data that support the findings of this study are available in the supporting information of this article.
