# Supplementary material for: Synthesis of α,α‐Difluoroketones via Zinc‐Mediated Cross‐Electrophile Coupling of gem‐Difluoroenol Sulfonates and N‐(acyloxy)phthalimides
Source: ChemistryOpen. 2026 Aug 20;15(9):e70285. doi: 10.1002/open.70285 (PMC13494356; doi:10.1002/open.70285)

Supporting Information

Synthesis of α,α-difluoroketones via zinc-mediated cross-electrophile coupling of *gem*-difluoroenol sulfonates and N-(acyloxy)phthalimides

Jing Zhao [a§], Li Yang [a§], Xiaohong Wang [a], Baojian Xiong [a], Xinmin Li [a*], Huan Yang [a*]

[a] School of Pharmacy, Guizhou International Science & Technology Cooperation Base of Medical Optical Theranostics Research, Zunyi Medical University, Zunyi, Guizhou 563003, China.

[§] These authors contributed equally to this work.

E-mail addresses: lixm@zmu.edu.cn; yanghuan837@163.com.

List of Contents

1) General information and materials..…………………………………………..S3
2) Synthesis of the *gem*-difluoroenol sulfonates **1**………….................................S4
3) Synthesis of the N-(acyloxy)phthalimide **2**……...S4
4) General procedure………………………………………………………...…..S5
5) Gram scale synthesis of **3s**...S5
6) Synthetic transformations of **3s**..S6
7) Characterization data of products...................………………………………...S8

General information and Materials:

Unless otherwise noted, all reactions or reagents were obtained from commercial suppliers and used as received. Unless otherwise noted, the oxygen-free conditions were carried in an argon atmosphere glovebox (Vigor, SGI800-750TS-F). All work-up and purification procedures were carried out with reagent-grade solvents in air.

Thin Layer Chromatography analyses were performed on silica gel coated glass plates (0.25 mm) with fluorescence indicator UV254. For detection of spots, irradiation of UV light at 254 nm or staining reagent using phosphomolybdic acid solution was used. Flash column chromatography was conducted with silica gel 60 (particle size 230–400 mesh, Huanghai) at room temperature and under elevated pressure.

^{1}H NMR, ^{13}C NMR and ^{19}F NMR spectra were recorded at 400 MHz, 101 MHz and 376 MHz, respectively in $CDCl_3$ (or DMSO-d_6) at room temperature. ^{1}H NMR was reported as follows: chemical shift, multiplicity (s = singlet, d = doublet, t = triplet, q = quadruplet, m = multiplet), coupling constant (*J* values) in Hz and integration.

Experimental Section

Synthesis of the *gem*-difluoroenol sulfonates 1 [1]

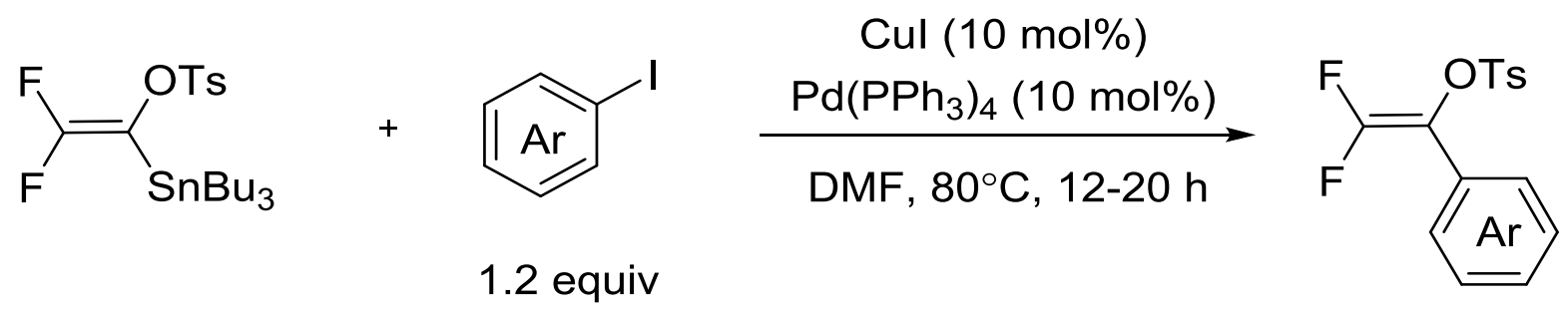

A 25 mL two-necked round-bottom flask equipped with a magnetic stir bar, rubber septum, and reflux condenser connected to an argon line was charged sequentially with 2,2-difluoro-1-(tributylstannyl)vinyl p-toluenesulfonate (0.3 g, 0.57 mmol), aryl iodide (0.69 mmol), tetrakis(triphenylphosphine)palladium(0) (10 mol%), copper(I) iodide (10 mol%), and N,N-dimethylformamide (12 mL). The reaction mixture was stirred at 80°C under argon atmosphere for 16 h, then cooled to room temperature and quenched with water. The mixture was extracted with diethyl ether (2×15 mL), and the combined organic layers were washed successively with 5% aqueous potassium fluoride solution and brine, dried over anhydrous sodium sulfate, filtered, and concentrated under reduced pressure. The crude product was purified by flash column chromatography on silica gel using a gradient elution of ethyl acetate/hexane to afford the pure product.

Synthesis of the N-(acyloxy)phthalimide 2 [2]

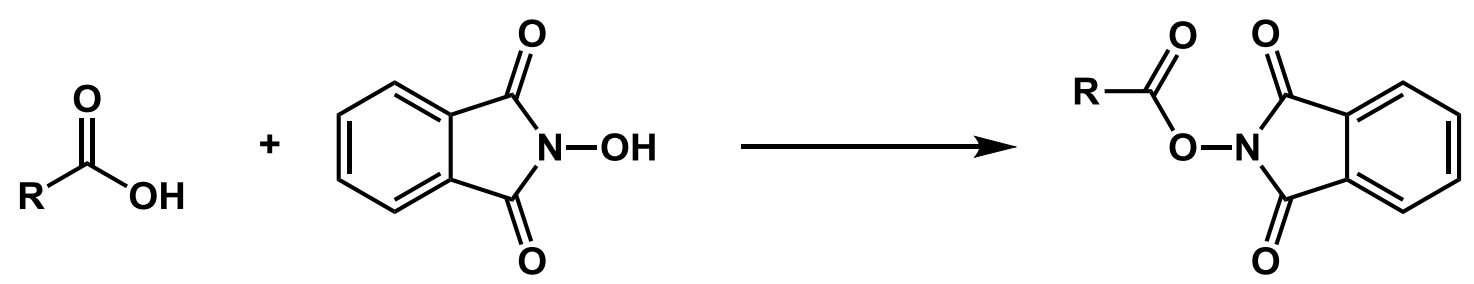

To a round-bottom flask equipped with a stir bar were added N-hydroxypyrrolidone (NHP) (3.26 g, 20 mmol), 4-dimethylaminopyridine (DMAP) (244 mg, 2 mmol), and dichloromethane (DCM) (50 mL). The carboxylic acid (20 mmol, 1.0 equiv) was then added to the mixture. Under stirring, N,N'-diisopropylcarbodiimide (DIC) (3.4 mL, 22 mmol, 1.1 equiv) was added dropwise, resulting in rapid formation of a precipitate. The reaction mixture was stirred at room temperature for 1 h, after which it was filtered through a short silica gel pad. The collected solid was thoroughly washed with DCM. The combined filtrates were concentrated under reduced pressure, and the crude product was further purified either by recrystallization (petroleum ether/ethyl acetate) or by flash column chromatography on silica gel.

General procedure:

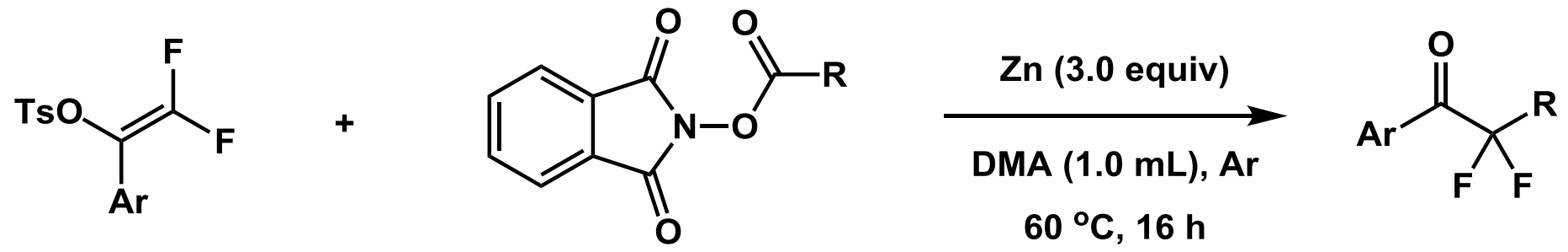

1 2 3

In an argon fulfilled glovebox, an oven-dried 15 ml vial was charged with a magnetic stirring bar, *gem*-difluorovinyl tosylates **1** (0.2 mmol, 1.0 equiv.), N-(acyloxy)phthalimide **2** (0.6 mmol, 3.0 equiv.), Zn (0.6 mmol, 3.0 equiv.), followed by addition of DMA (1.0 mL). The vial was sealed and removed out of the glovebox and heated to 60 °C using heating mantle with 600-700 rpm stirring speed. After 16 h, the vial was cooled to room temperature. The mixture was passed through a short silica gel pad with EtOAc. The filtrate was concentrated and the residue was purified by flash column chromatography to give the desired product **3**.

Gram scale synthesis of 3s

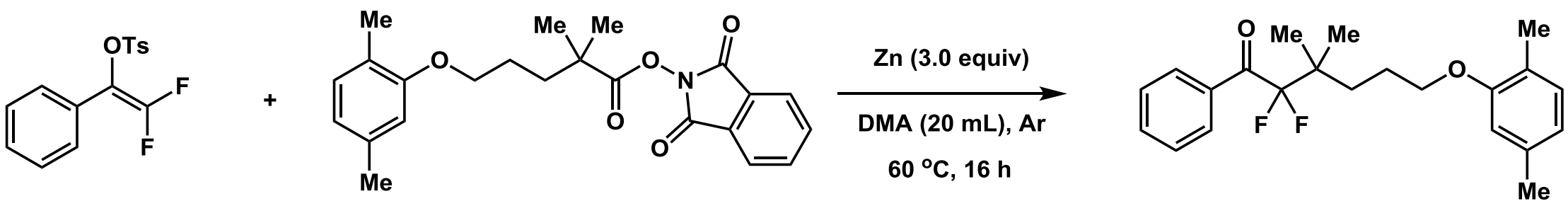

1a 2s 3s, 91%

In an argon fulfilled glovebox, an oven-dried 100 ml Schlenk tube was charged with a magnetic stirring bar, *gem*-difluorovinyl tosylates **1a** (1g, 3.23 mmol, 1.0 equiv.), N-(acyloxy)phthalimide **2s** (3.83g, 9.69 mmol, 3.0 equiv.), Zn (630 mg, 9.69 mmol, 3.0 equiv.), followed by addition of DMA (20 mL). The tube was sealed and removed out of the glovebox and heated to 60 °C using heating mantle with 600-700 rpm stirring speed. After 16 h, the vial was cooled to room temperature. The mixture was passed through a short silica gel pad with EtOAc. The filtrate was concentrated and the residue was purified by flash column chromatography to give the desired product **3s**.

Synthetic transformations of 3s

1) Preparation of compound 4a:

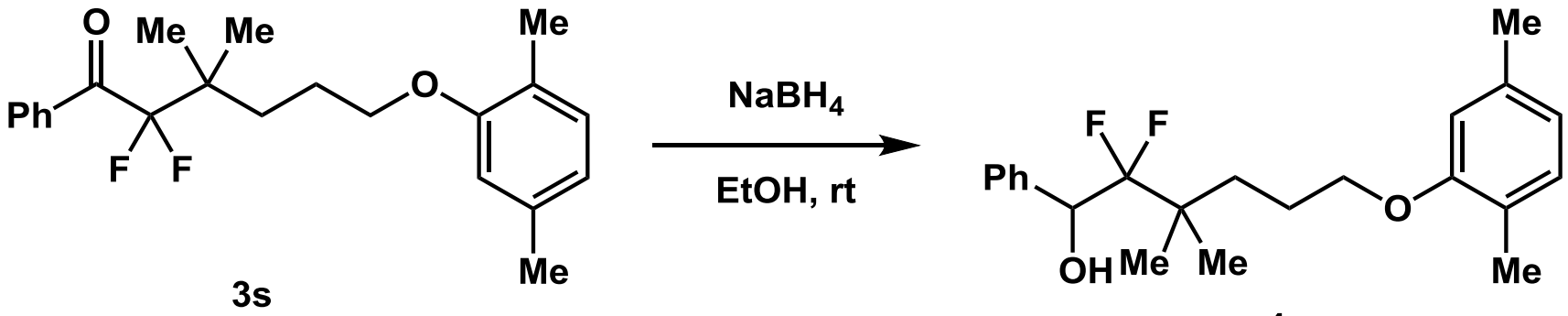

To a solution of compound **3s** (0.2 mmol, 72 mg, 1.0 equiv.) in EtOH (2 mL), $NaBH_4$ (23 mg, 0.6 mmol, 3 equiv.) was added in one portion at room temperature. The resulting mixture was stirred at room temperature for 1 hour. After complete conversion, the reaction was quenched by NH_4Cl (saturated aq.), and extracted with ethyl acetate. The combined organic layers were dried over anhydrous Na_2SO_4 and evaporated under reduced pressure. The desired product **4a** was then purified by chromatography on silica gel.

2) Preparation of compound 4b:

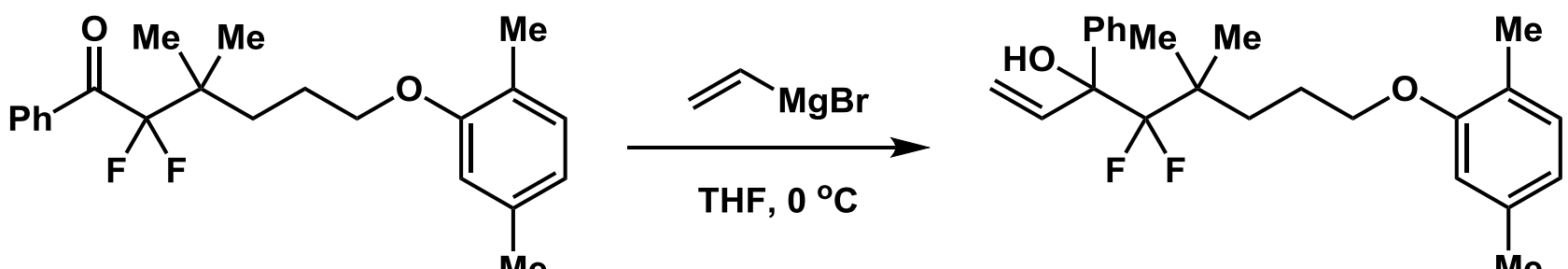

To solution of **3s** (0.2 mmol, 72 mg, 1.0 equiv.) in anhydrous THF, vinylmagnesium bromide (0.4 mL, 1.0 mol/L in THF, 4 equiv.) was added dropwise under argon at 0 °C. The reaction was then stirred at 0 °C for 4 hours and was quenched with NH_4Cl (saturated aq.), and extracted with ethyl acetate. The combined organics were washed with brine, dried over anhydrous Na_2SO_4 and evaporated under reduced pressure. The crude residue was purified by silica-gel column chromatography to afford desired the product **4b**.

3) Preparation of compound 4c:

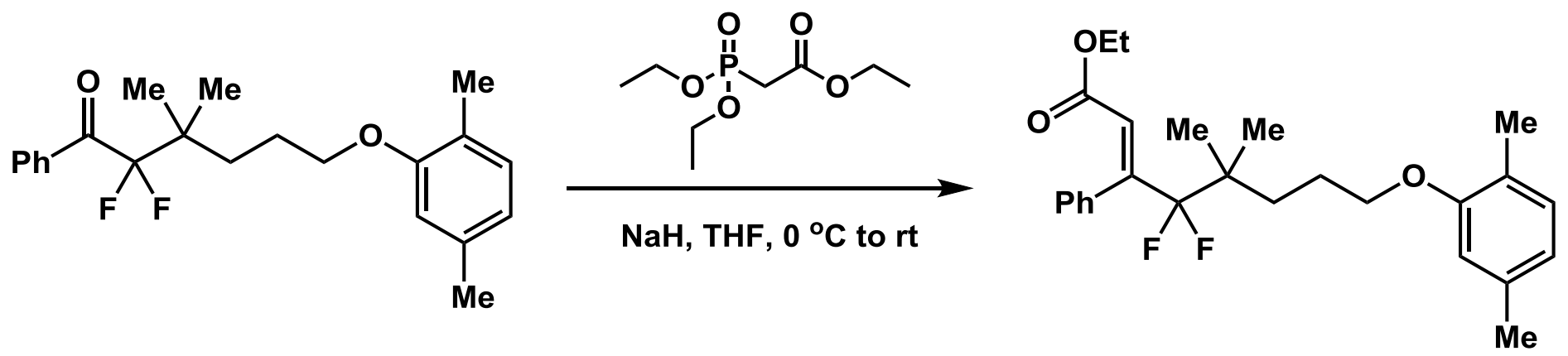

3s 4c

To a solution of ethyl 2-(diethoxyphosphoryl)acetate (54 mg, 0.24 mmol, 1.2 equiv.) in THF, a solution of NaH (16 mg, 0.4 mmol, 60% in mineral oil) was added dropwise. The mixture was stirred for 30 min after which **3s** (0.2 mmol, 72 mg, 1.0 equiv.) was added. The reaction mixture was stirred over night at room temperature. After completion of the reaction, the mixture was quenched by water and then extracted with ethyl acetate. The combined organics were washed with brine, dried over anhydrous Na_2SO_4 and evaporated under reduced pressure. The crude residue was purified by silica-gel column chromatography to afford desired the product **4c**.

HRMS of compound 5

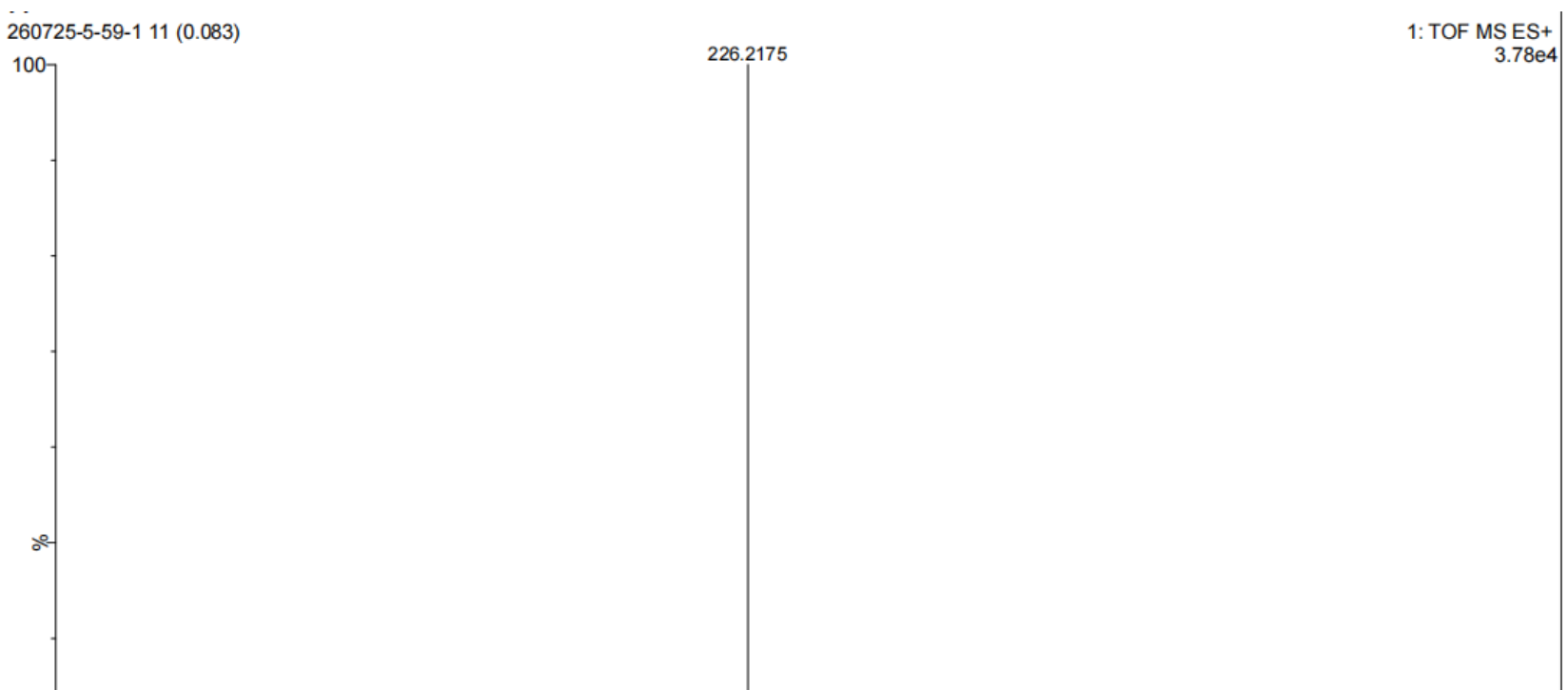

HRMS of compound 6

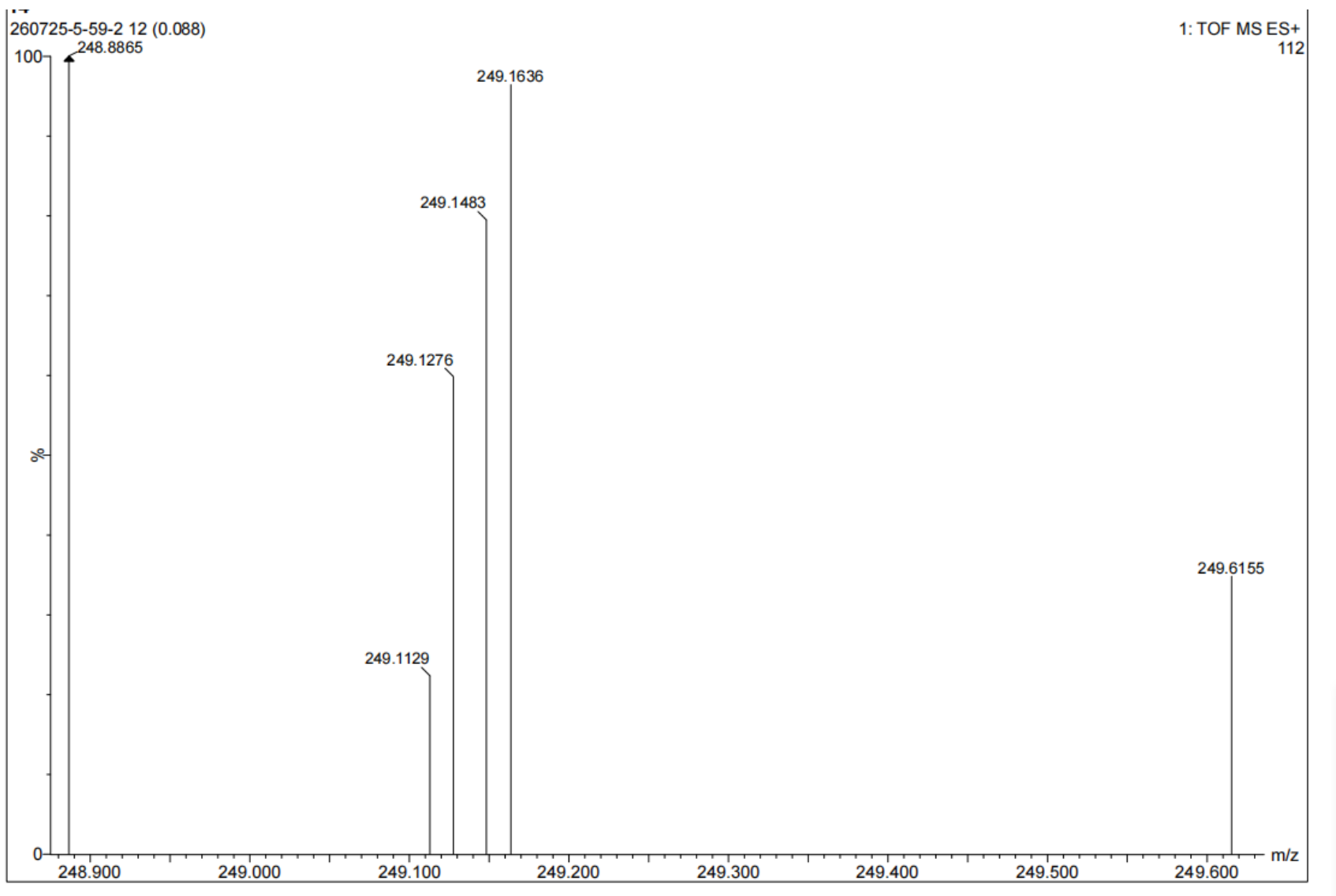

260725-5-59-2 12 (0.088)
1: TOF MS ES+
112
100
248.8865
249.1636
249.1483
249.1276
%
249.6155
249.1129
0
248.900
249.000
249.100
249.200
249.300
249.400
249.500
249.600
m/z

2-cyclohexyl-2,2-difluoro-1-phenylethan-1-one (3a)

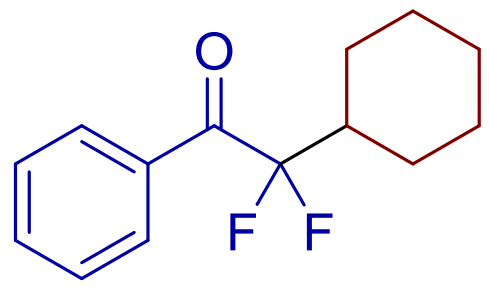

The product (39.1 mg, 82% yield) as colorless oil was purified with silica gel chromatography (petroleum ether/ ethyl acetate = 20/1). **^{1}H NMR** (400 MHz, Chloroform-*d*) δ 8.00 (d, *J* = 7.6 Hz, 2H), 7.57-7.53 (m, 1H), 7.42 (t, *J* = 7.6 Hz, 2H), 3.25-2.09 (m, 1H), 1.77-1.72 (m, 4H), 1.63-1.61 (m, 1H), 1.27-1.09 (m, 5H). **^{19}F NMR** (376 MHz, Chloroform-*d*) δ -108.51 (s, 2F). **^{13}C NMR** (101 MHz, Chloroform-*d*) δ 190.4 (t, *J* = 30.3), 134.1, 132.9 (t, *J* = 2.0 Hz), 130.0 (t, *J* = 4.0 Hz), 128.7, 120.3 (t, *J* = 255.5 Hz), 42.2 (t, *J* = 21.2 Hz), 25.9, 25.5, 24.8 (t, *J* = 4.0 Hz). **HRMS** (EI-TOF) m/z: $[M+Na]^+$ Calcd. for $C_{14}H_{16}F_2NaO$ 261.1061; Found 261.1063.

^{1}H NMR spectrum (400 MHz, $CDCl_3$) of compound **3a**

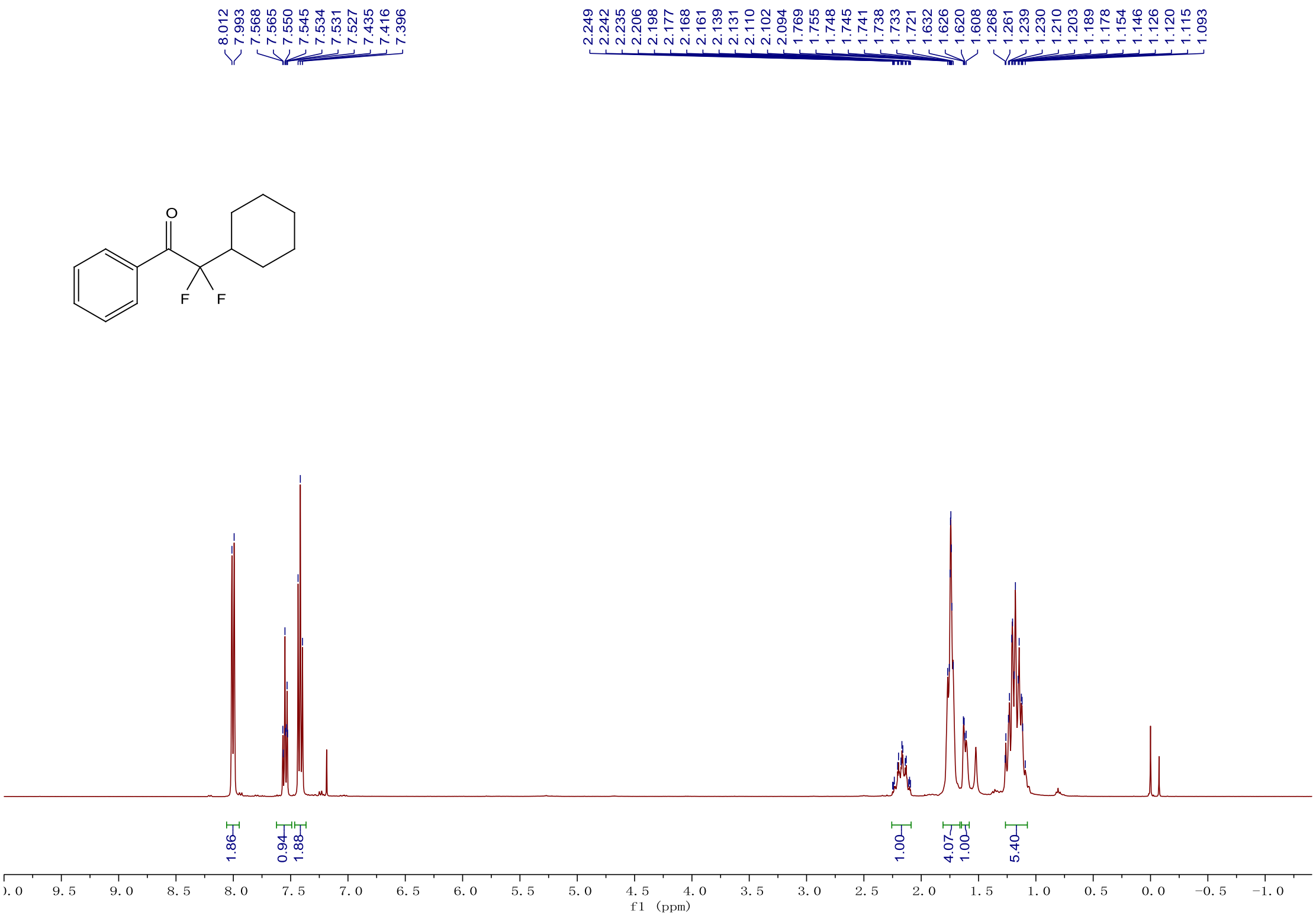

^{19}**F NMR** spectrum (376 MHz, $CDCl_3$) of compound **3a**

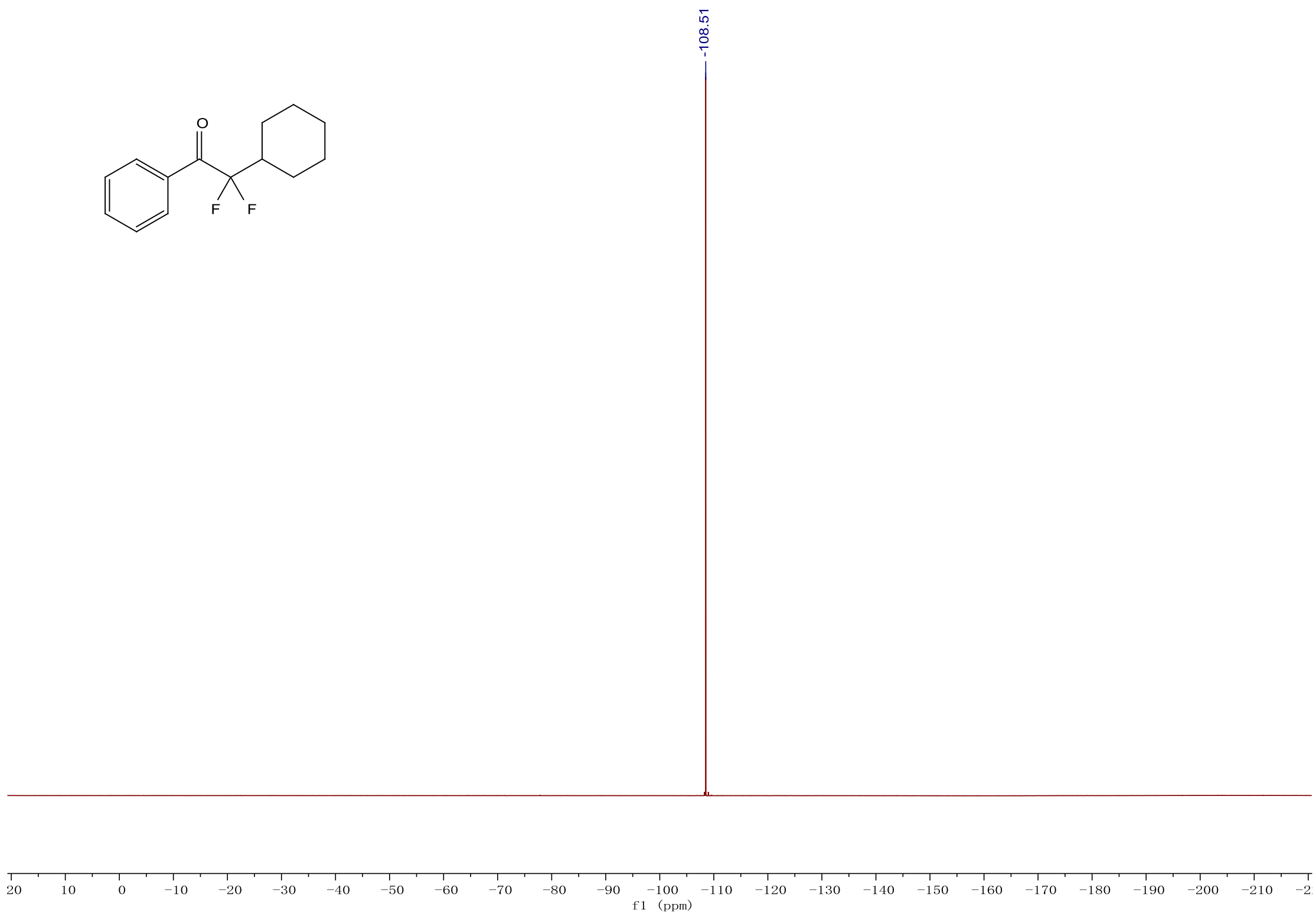

^{13}**C NMR** spectrum (101 MHz, $CDCl_3$) of compound **3a**

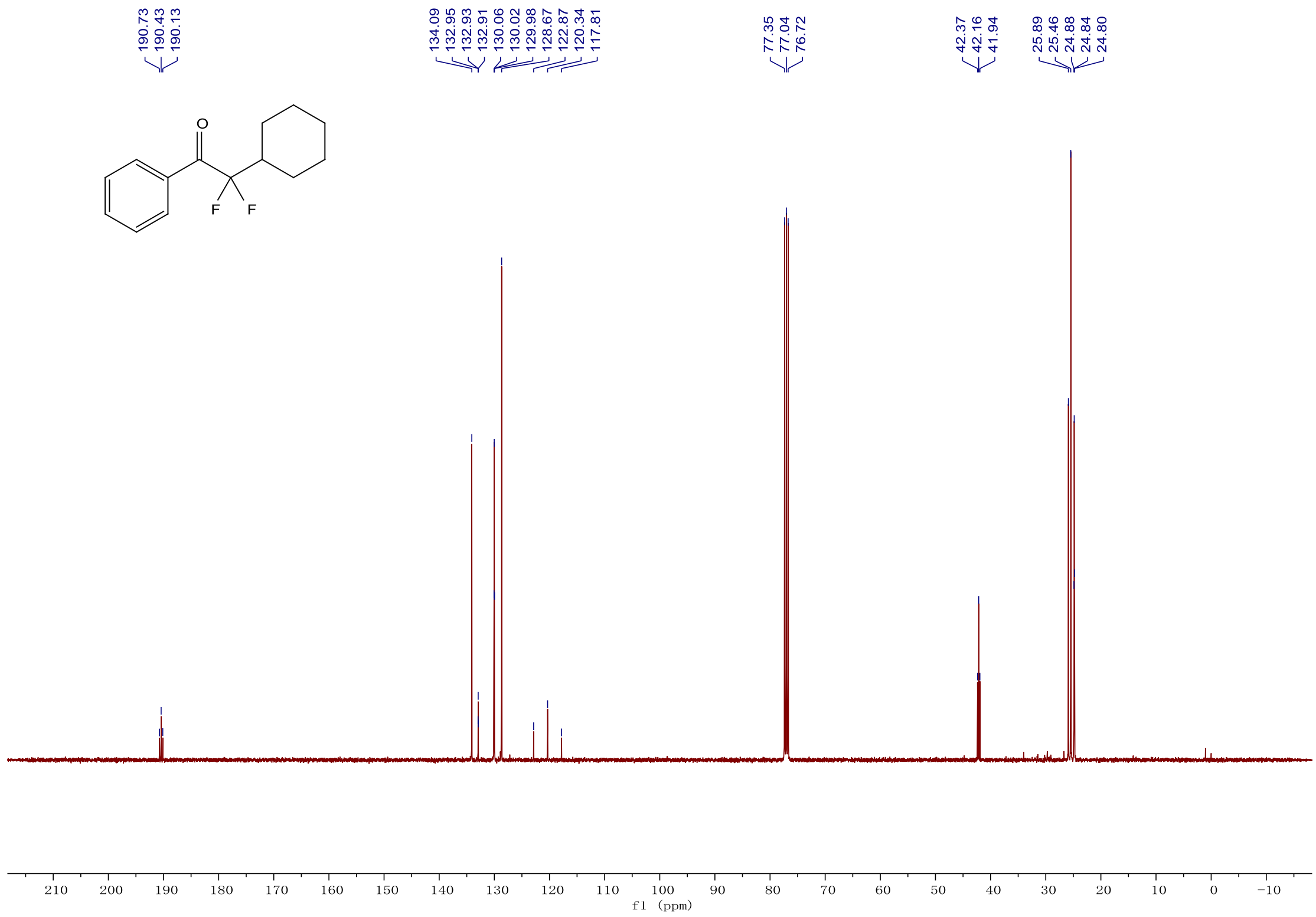

2,2-difluoro-1-phenylhexan-1-one (3b)

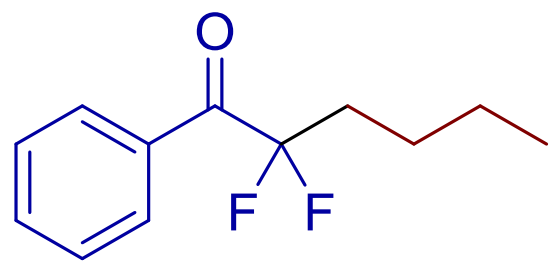

The product (29.7 mg, 70% yield) as colorless oil was purified with silica gel chromatography (petroleum ether/ ethyl acetate = 20/1). ^{1}**H NMR** (400 MHz, Chloroform-*d*) δ 8.11 (d, *J* = 7.2 Hz, 2H), 7.65-7.61 (m, 1H), 7.49 (t, *J* = 8.0 Hz, 2H), 2.25-2.09 (m, 2H), 1.57-1.49 (m, 2H), 1.45-1.36 (m, 2H), 0.92 (t, *J* = 7.2 Hz, 3H). ^{19}**F NMR** (376 MHz, Chloroform-*d*) δ -100.22 (s, 2F). ^{13}**C NMR** (101 MHz, Chloroform-*d*) δ 189.70 (t, *J* = 31.3 Hz), 134.2, 132.2 (t, *J* = 3.0 Hz), 130.1 (t, *J* = 4.0 Hz), 128.7, 119.9 (t, *J* = 253.5 Hz), 33.8 (t, *J* = 23.2 Hz), 23.4 (t, *J* = 4.0 Hz), 22.5, 13.8. **HRMS** (EI-TOF) m/z: $[M+H]^+$ Calcd. for $C_{12}H_{15}F_2O$ 213.1085; Found 213.1092.

^{1}**H NMR** spectrum (400 MHz, $CDCl_3$) of compound **3b**

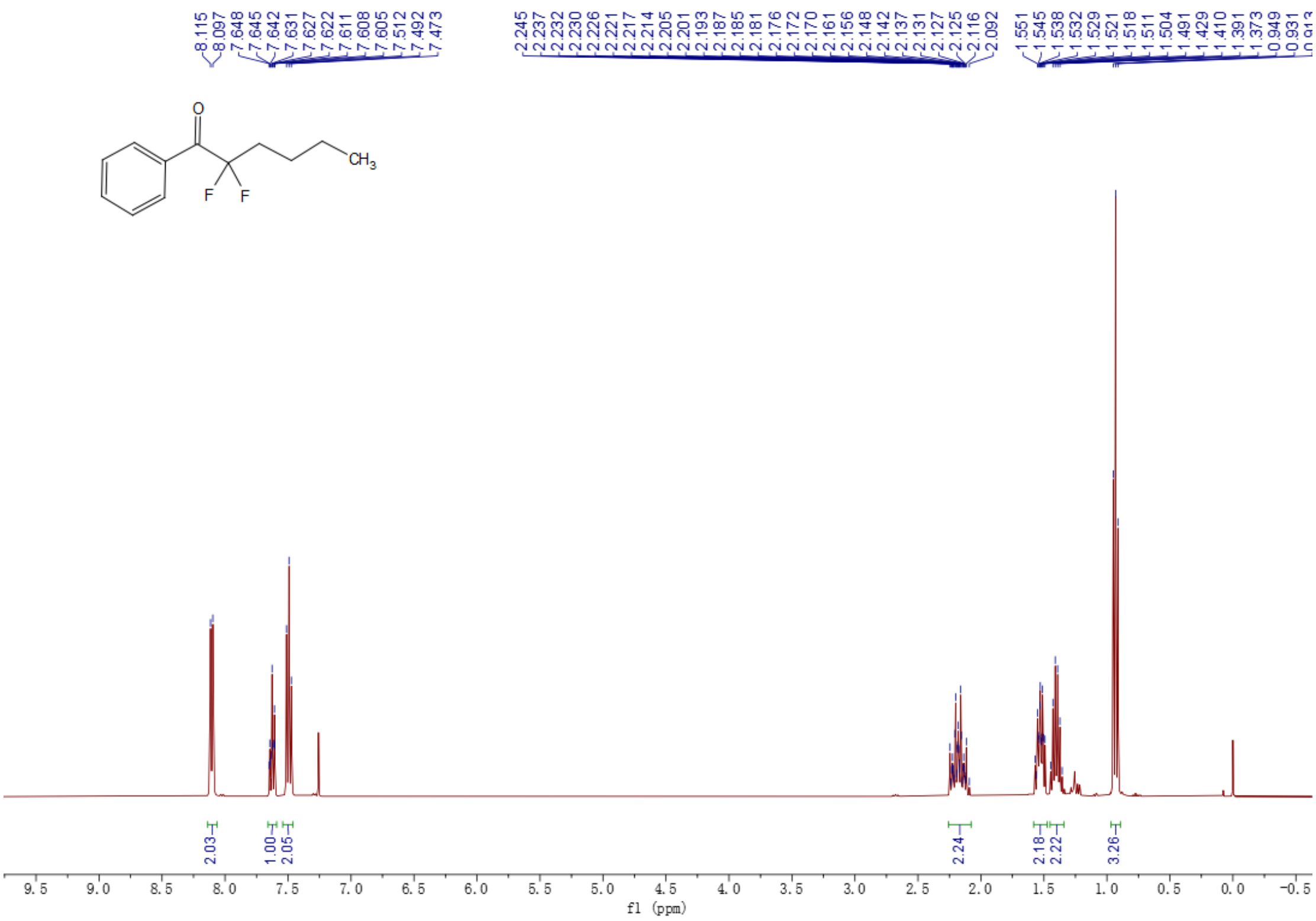

^{19}**F NMR** spectrum (376 MHz, $CDCl_3$) of compound **3b**

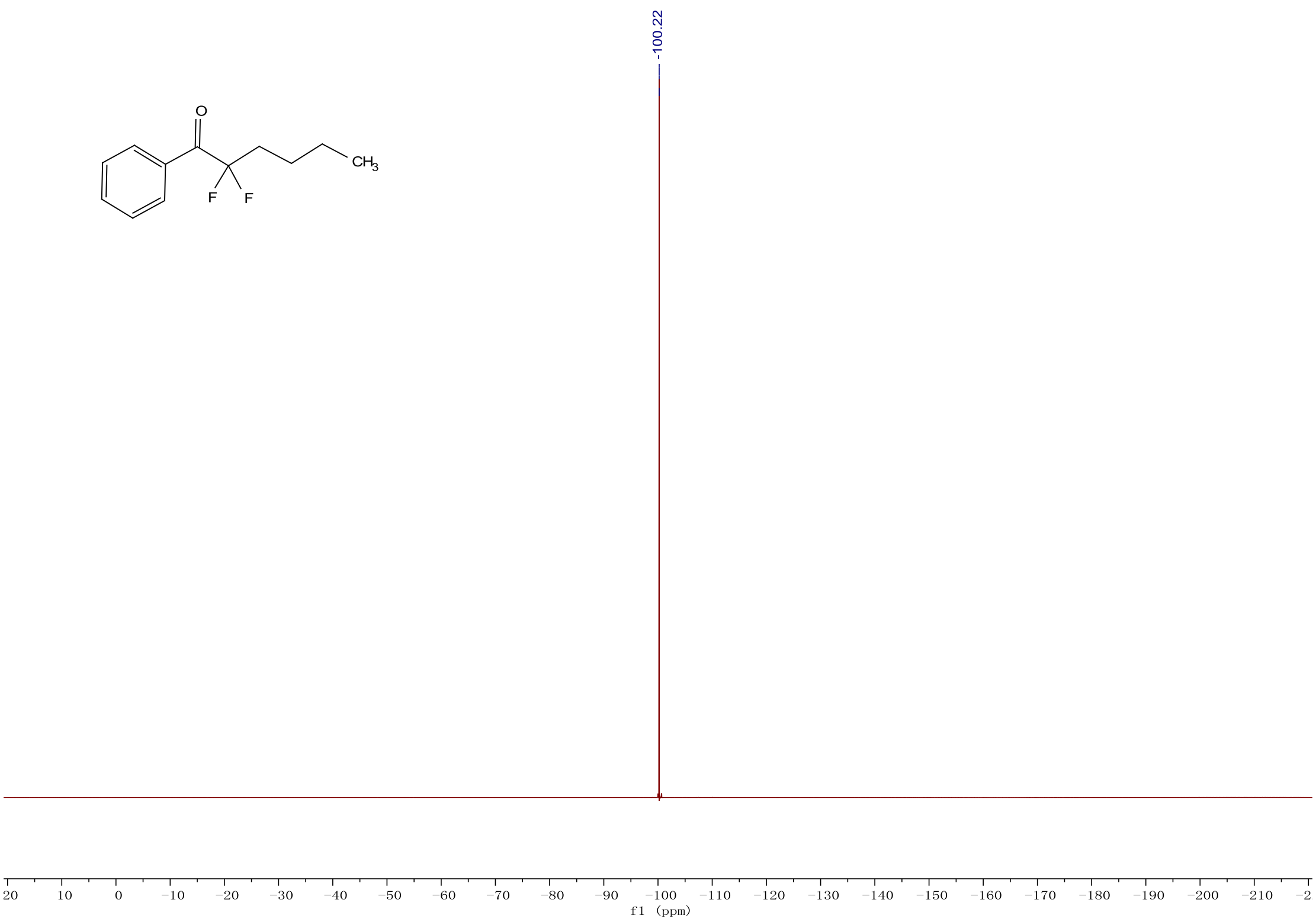

^{13}C NMR spectrum (101 MHz, $CDCl_3$) of compound **3b**

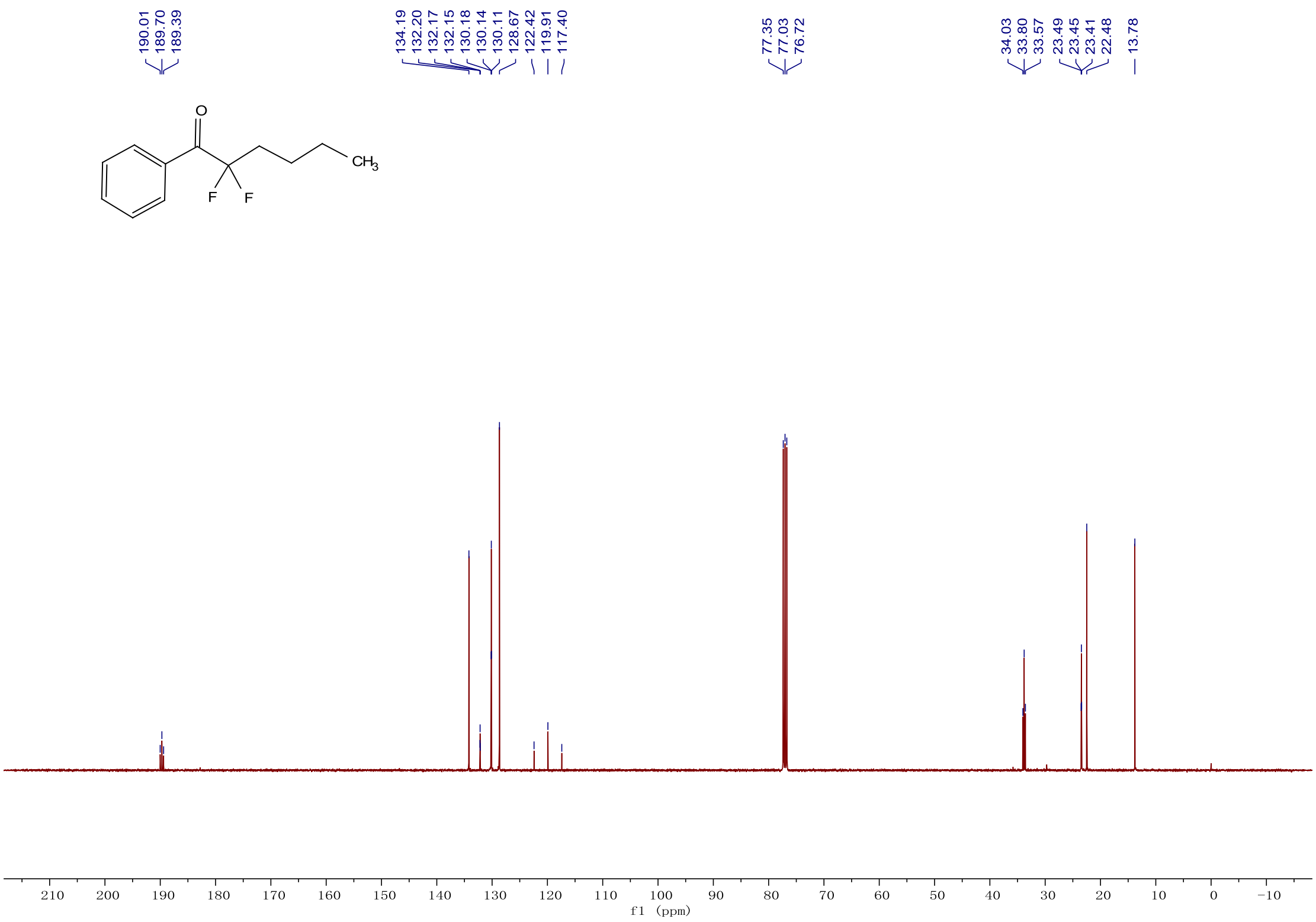

2,2-difluoro-1,5-diphenylpentan-1-one (3c)

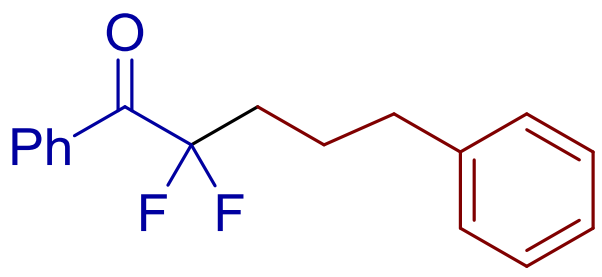

The product (36.3 mg, 66% yield) as colorless oil was purified with silica gel chromatography (petroleum ether/ ethyl acetate = 20/1). ^{1}**H NMR** (400 MHz, Chloroform-*d*) δ 8.09 (d, *J* = 7.6 Hz, 2H), 7.64-7.59 (m, 1H), 7.48 (t, *J* = 6.8 Hz, 2H), 7.30-7.26 (m, 2H), 7.21-7.17 (m, 3H), 2.71 (t, *J* = 7.6 Hz, 2H), 2.27-2.14 (m, 2H), 1.94-1.86 (m, 2H).^{19}**F NMR** (376 MHz, Chloroform-*d*) δ -100.00 (s, 2F). ^{13}**C NMR** (101 MHz, Chloroform-*d*) δ 189.5 (t, *J* = 31.5 Hz), 141.2, 134.3, 132.1 (t, *J* = 2.6 Hz), 130.2 (t, *J* = 3.3 Hz), 128.7, 128.5, 128.4, 126.1, 119.8 (t, *J* = 253.6 Hz), 35.3, 33.4 (t, *J* = 22.9 Hz), 23.1 (t, *J* = 4.3 Hz). **HRMS** (EI-TOF) m/z: $[M]^+$ Calcd. for $C_{17}H_{16}F_2NaO$ 297.1061; Found 297.1066.

^{1}**H NMR** spectrum (400 MHz, $CDCl_3$) of compound **3c**

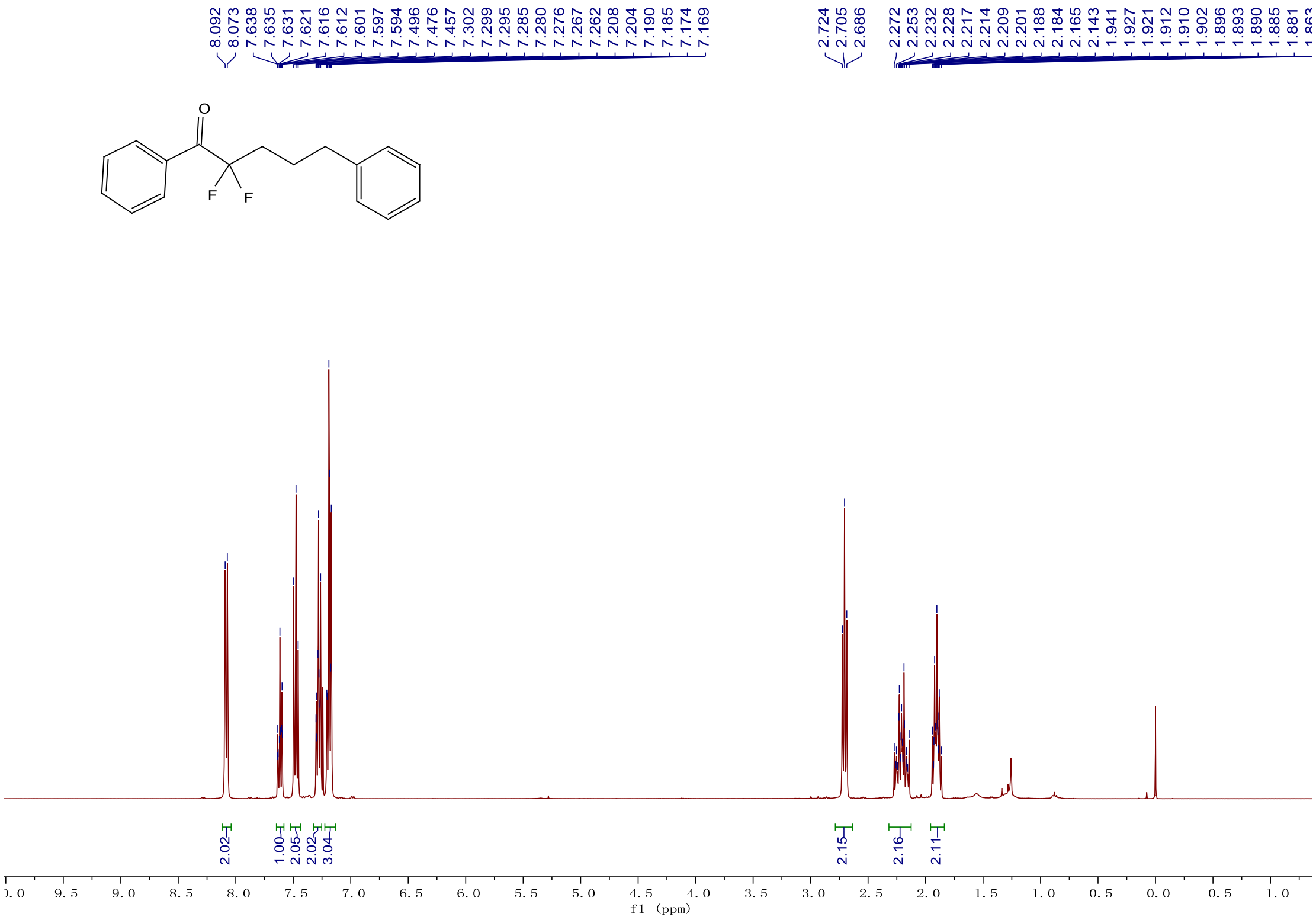

^{19}**F NMR** spectrum (376 MHz, $CDCl_3$) of compound **3c**

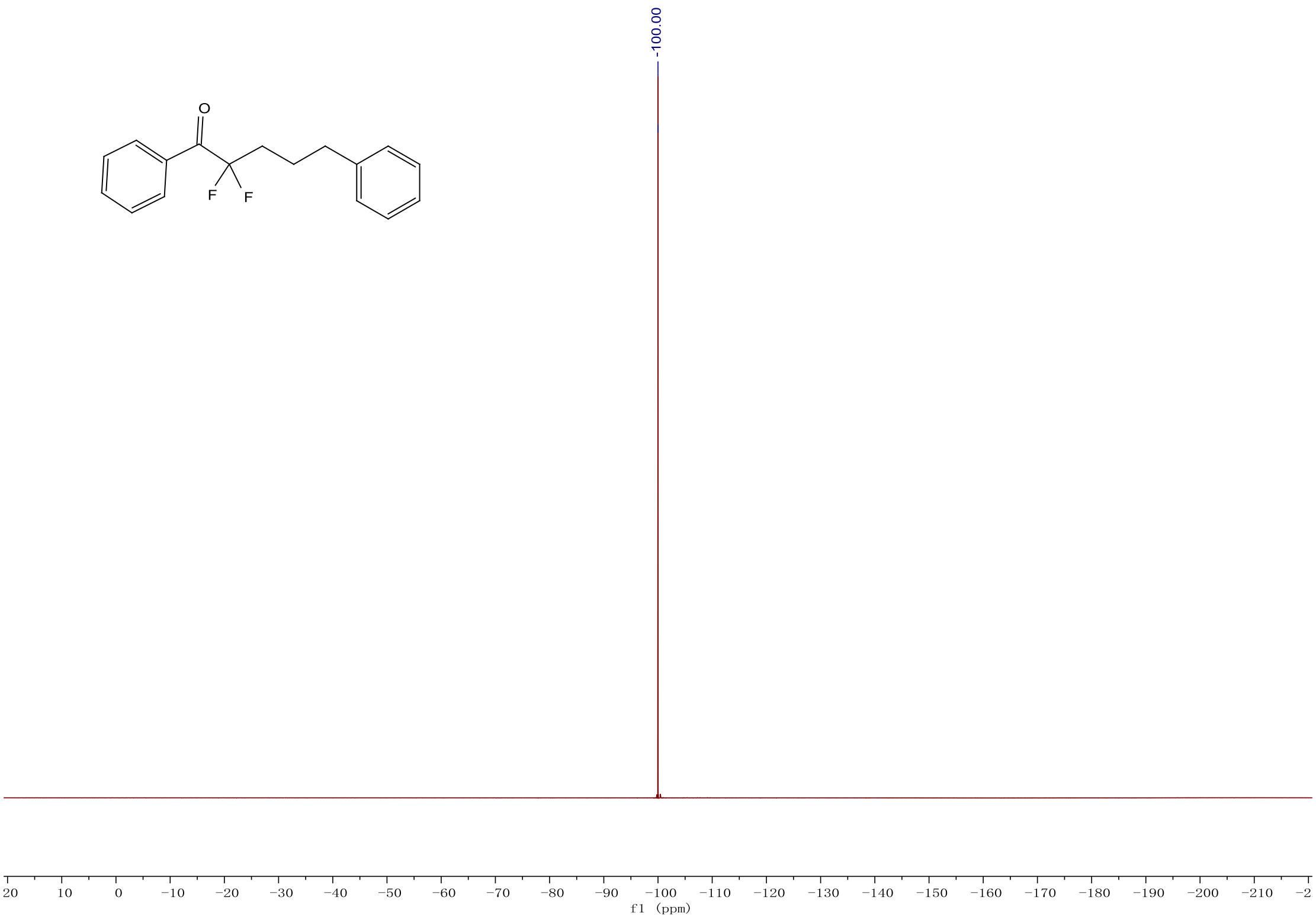

^{13}**C NMR** spectrum (101 MHz, $CDCl_3$) of compound **3c**

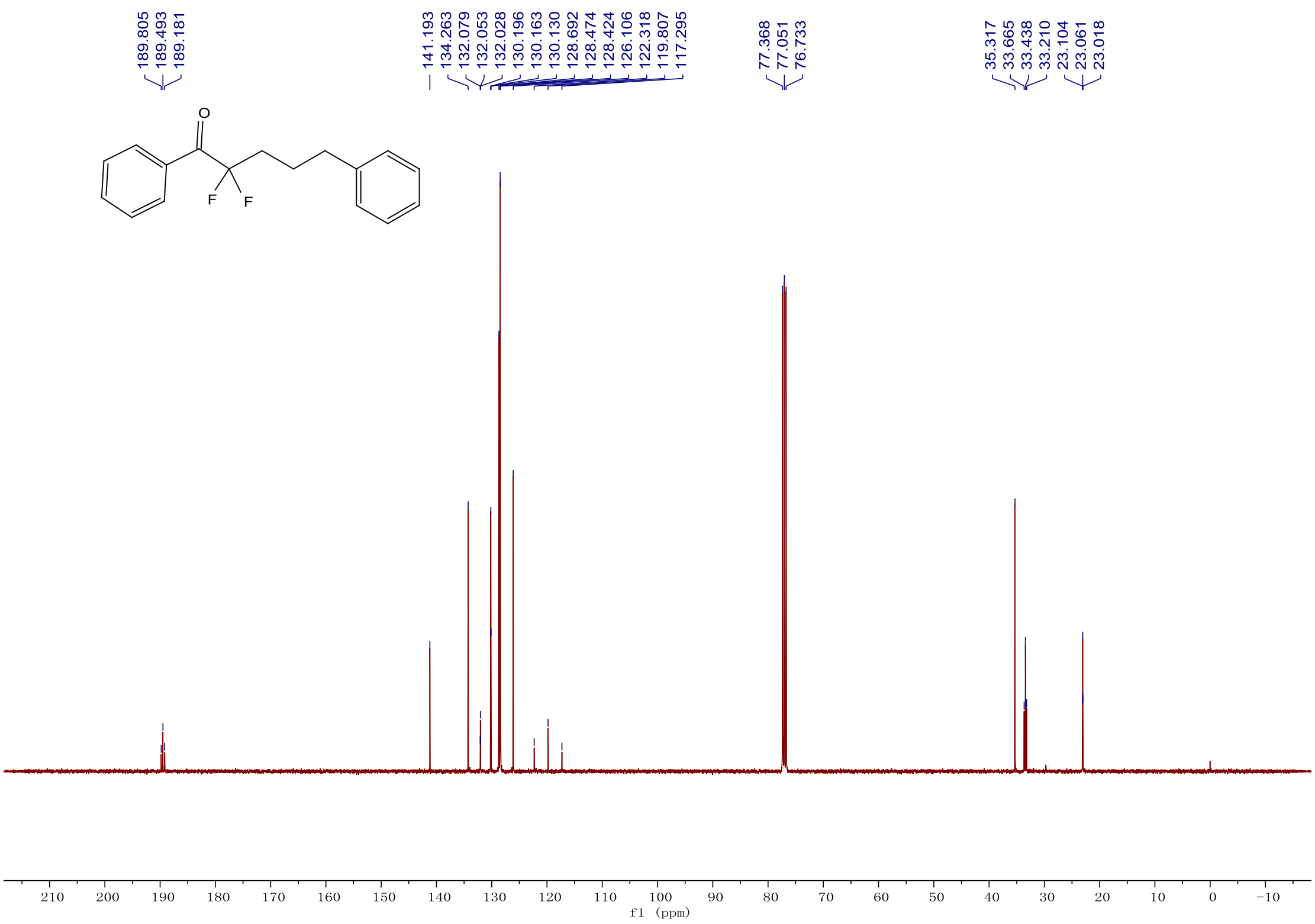

2,2-difluoro-1-phenylnonadec-10-en-1-one (3d)

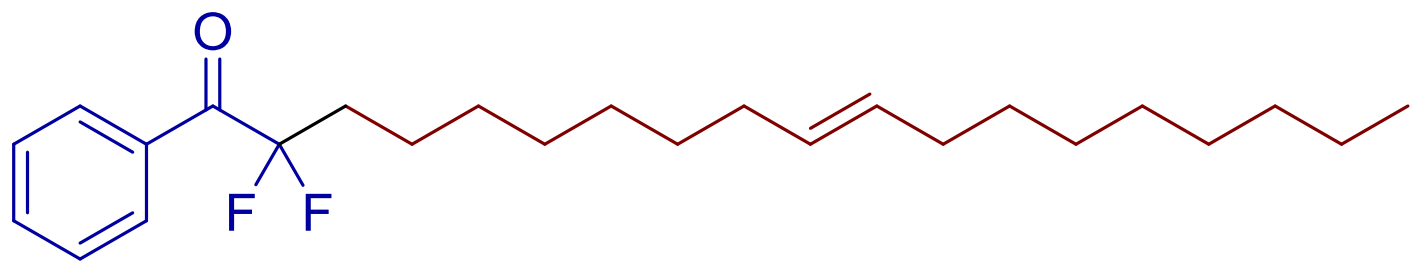

The product (43.1 mg, 55% yield) as colorless oil was purified with silica gel chromatography (petroleum ether/ ethyl acetate = 20/1). **^{1}H NMR** (400 MHz, Chloroform-*d*) δ 8.10 (d, *J* = 8.4 Hz, 2H), 7.64-7.61 (m, 1H), 7.49 (t, *J* = 8.0 Hz, 2H), 5.39-5.32 (m, 2H), 2.32-2.10 (m, 2H), 2.03-1.95 (m, 4H), 1.58-1.50 (m, 2H), 1.38-1.26 (m, 20H), 0.88 (t, *J* = 6.4 Hz, 3H). **^{19}F NMR** (376 MHz, Chloroform-*d*) δ -100.20 (s, 2F). **^{13}C NMR** (101 MHz, Chloroform-*d*) δ 189.7 (t, *J* = 31.3 Hz), 134.2, 132.2 (t, *J* = 2.0 Hz), 130.1 (t, *J* = 3.0 Hz), 130.0, 129.7, 128.7, 119.9 (t, *J* = 253.5 Hz), 34.1 (t, *J* = 23.2 Hz), 31.9, 29.8, 29.7, 29.5, 29.34, 29.32, 29.2, 29.1, 27.2 (d, *J* = 8.1 Hz), 22.7, 21.4 (t, *J* = 4.0 Hz), 14.1. **HRMS** (EI-TOF) m/z: [M+Na]$^{+}$ Calcd. for $C_{25}H_{38}F_2NaO$ 415.2783; Found 415.2781.

^{1}H NMR spectrum (400 MHz, $CDCl_3$) of compound **3d**

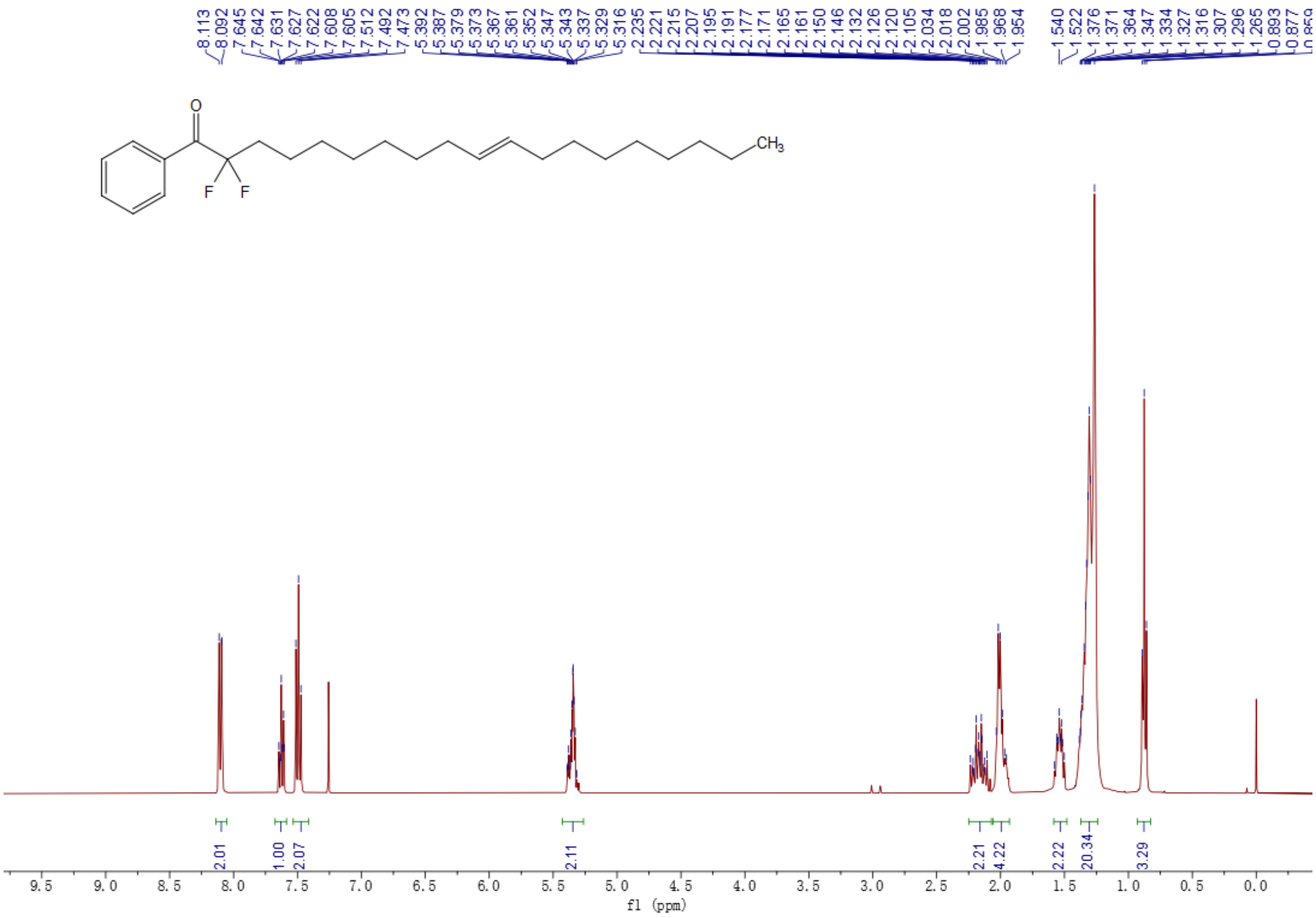

^{19}F NMR spectrum (376 MHz, $CDCl_3$) of compound **3d**

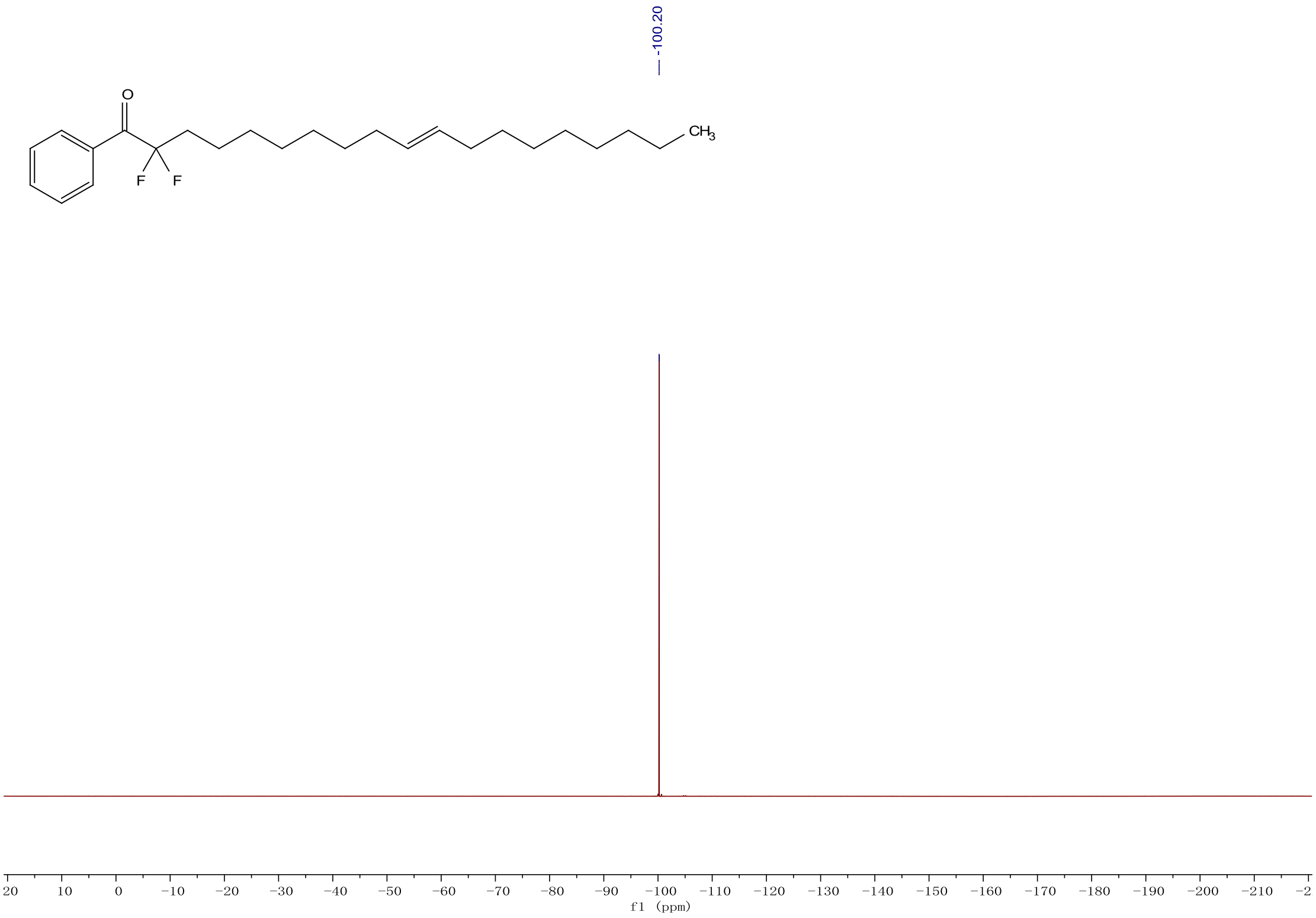

^{13}C NMR spectrum (101 MHz, $CDCl_3$) of compound **3d**

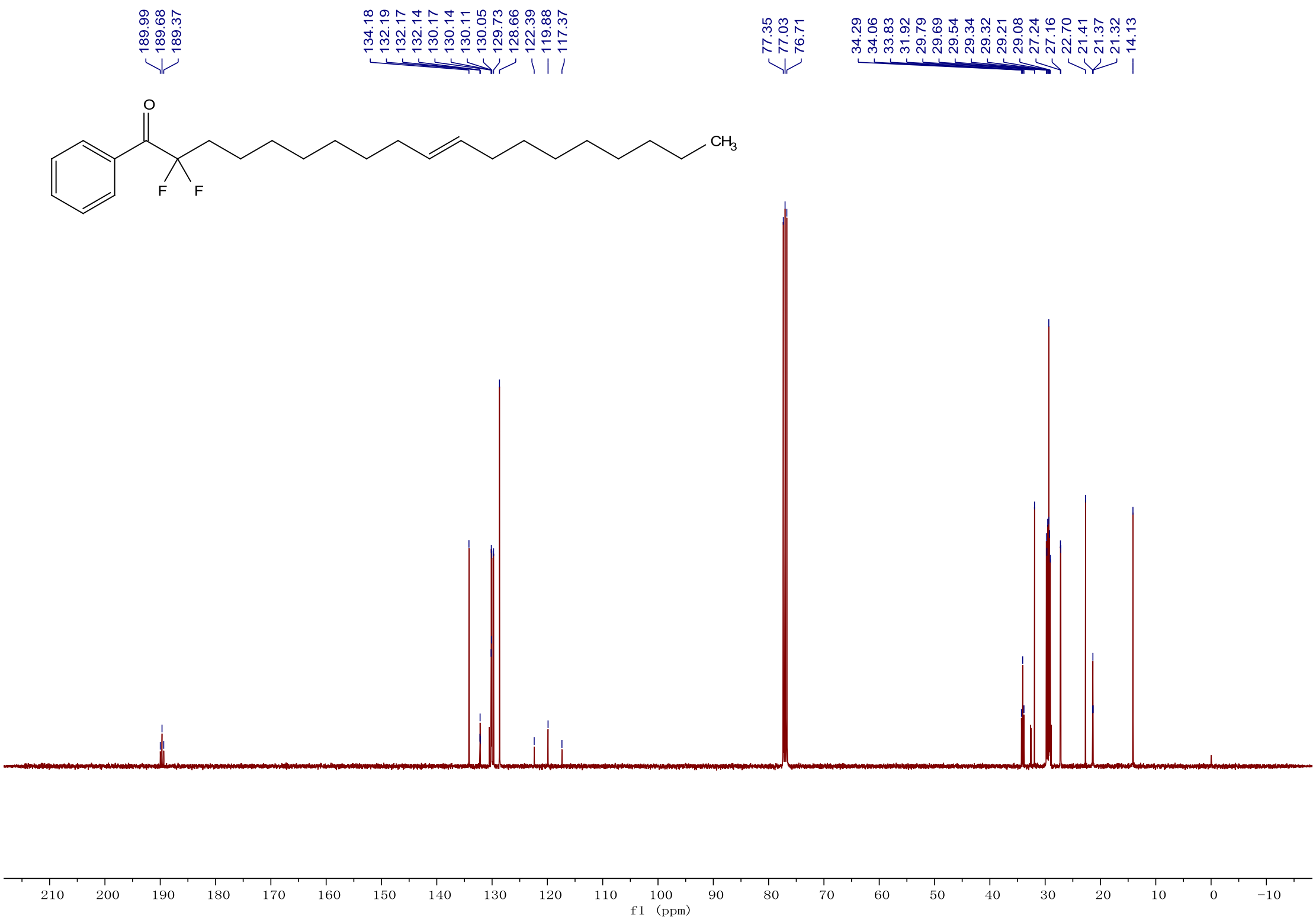

7-bromo-2,2-difluoro-1-phenylheptan-1-one (3e)

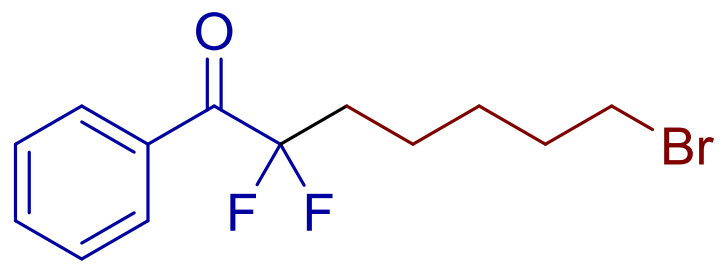

The product (36.7 mg, 60% yield) as colorless oil was purified with silica gel chromatography (petroleum ether/ ethyl acetate = 20/1). **^{1}H NMR** (400 MHz, Chloroform-*d*) δ 8.11 (d, *J* = 8.0 Hz, 2H), 7.66-61 (m, 1H), 7.50 (t, *J* = 8.0 Hz, 2H), 3.41 (t, *J* = 8.8 Hz, 2H), 2.26-2.14 (m, 2H), 1.93-1.86 (m, 2H), 1.63-1.50 (m, 4H).**^{19}F NMR** (376 MHz, Chloroform-*d*) δ -100.10 (s, 2F). **^{13}C NMR** (101 MHz, Chloroform-*d*) δ 189.4 (t, *J* = 31.3 Hz), 134.3, 132.0 (t, *J* = 2.0 Hz), 130.2 (t, *J* = 3.0 Hz), 128.7, 119.7 (t, *J* = 253.5 Hz), 33.8 (t, *J* = 23.2 Hz), 33.4, 32.4, 27.8, 20.7 (t, *J* = 4.0 Hz). **HRMS** (EI-TOF) m/z: $[M+Na]^+$ Calcd. for $C_{13}H_{15}BrF_2NaO$ 327.0167; Found 327.0169.

^{1}H NMR spectrum (400 MHz, $CDCl_3$) of compound **3e**

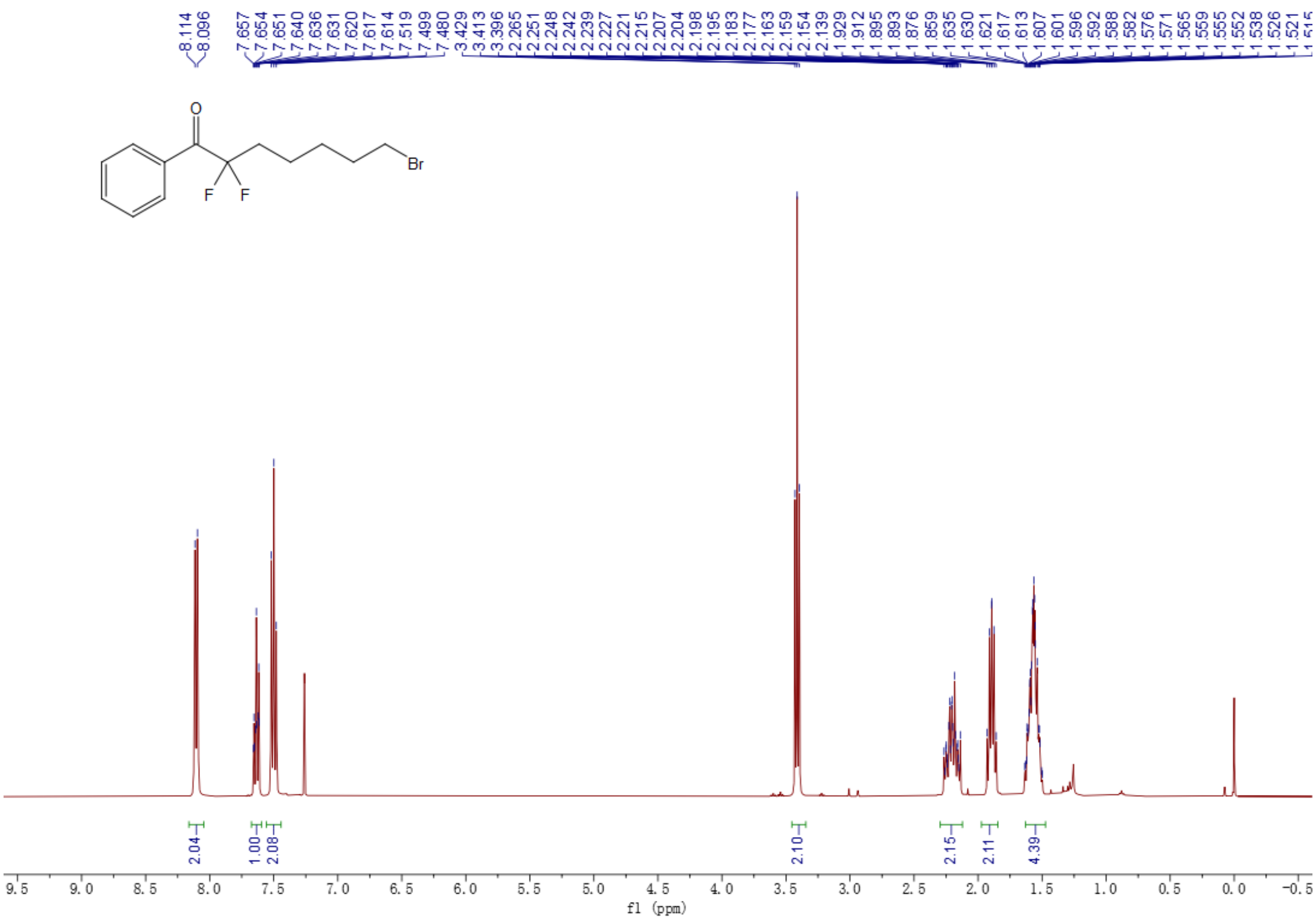

^{19}F NMR spectrum (376 MHz, $CDCl_3$) of compound **3e**

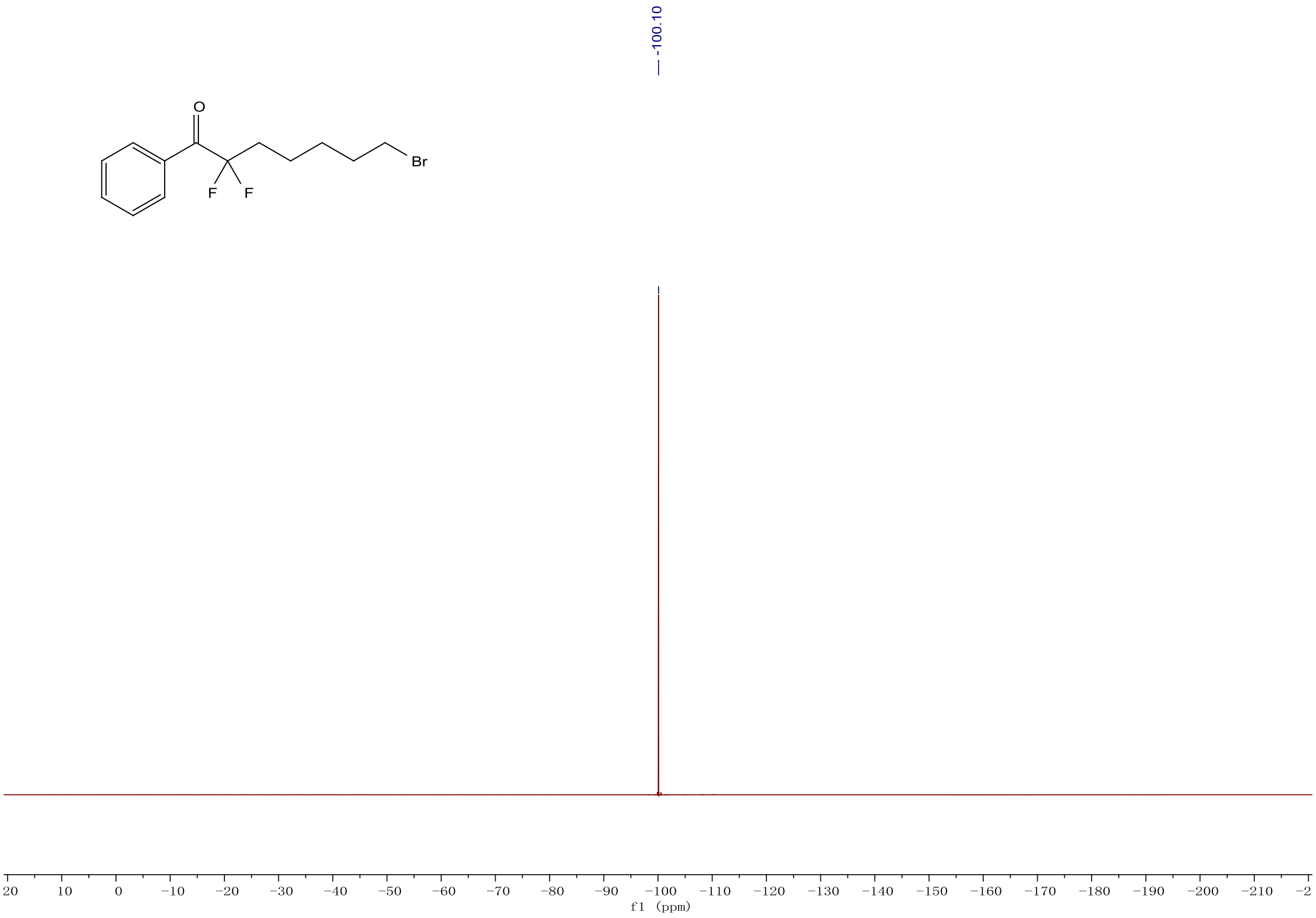

^{13}C NMR spectrum (101 MHz, $CDCl_3$) of compound **3e**

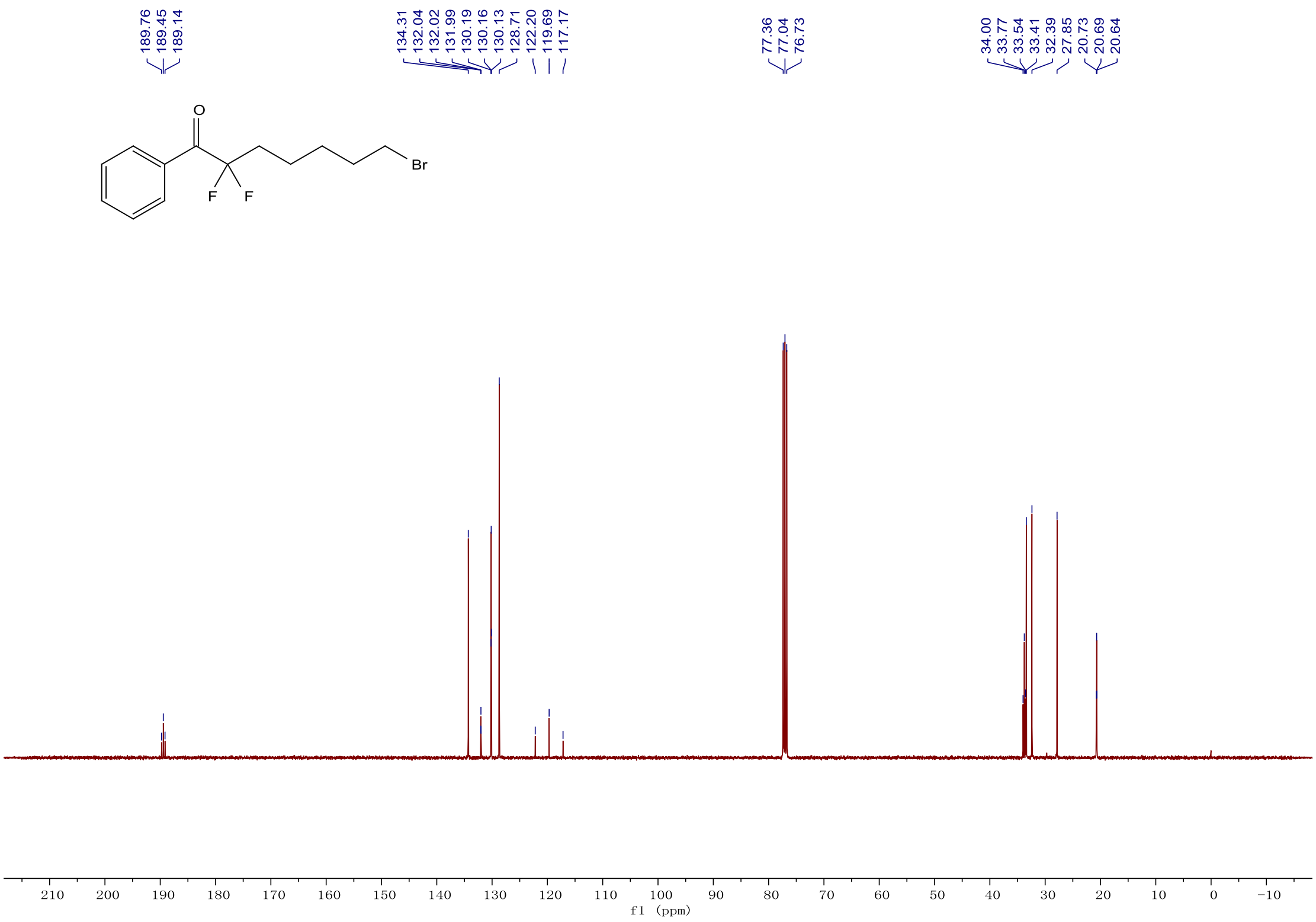

tert-butyl (2,2-difluoro-3-oxo-3-phenylpropyl)carbamate (3f)

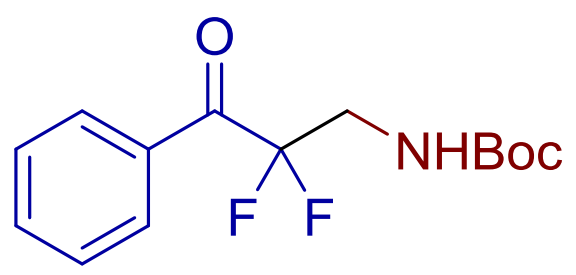

The product (38.8 mg, 68% yield) as colorless oil was purified with silica gel chromatography (petroleum ether/ ethyl acetate = 10/1). **^{1}H NMR** (400 MHz, Chloroform-*d*) δ 8.12 (d, *J* = 8.0 Hz, 2H), 7.65 (t, *J* = 8.0 Hz, 1H), 7.51 (t, *J* = 8.0 Hz, 2H), 4.99 (s, 1H), 3.96-3.88 (m, 2H), 1.43 (s, 9H). **^{19}F NMR** (376 MHz, Chloroform-*d*) δ -150.71 (s, 2F). **^{13}C NMR** (101 MHz, Chloroform-*d*) δ 188.9 (t, *J* = 31.3 Hz), 155.6, 134.7, 131.6 (t, *J* = 3.0 Hz), 130.0 (t, *J* = 3.0 Hz), 128.8, 116.5 (t, *J* = 257.5 Hz), 80.2, 43.2 (t, *J* = 27.3 Hz), 28.2. **HRMS** (EI-TOF) m/z: $[M+Na]^+$ Calcd. for $C_{14}H_{17}F_2NNaO_3$ 308.1069; Found 308.1078.

^{1}H NMR spectrum (400 MHz, $CDCl_3$) of compound **3f**

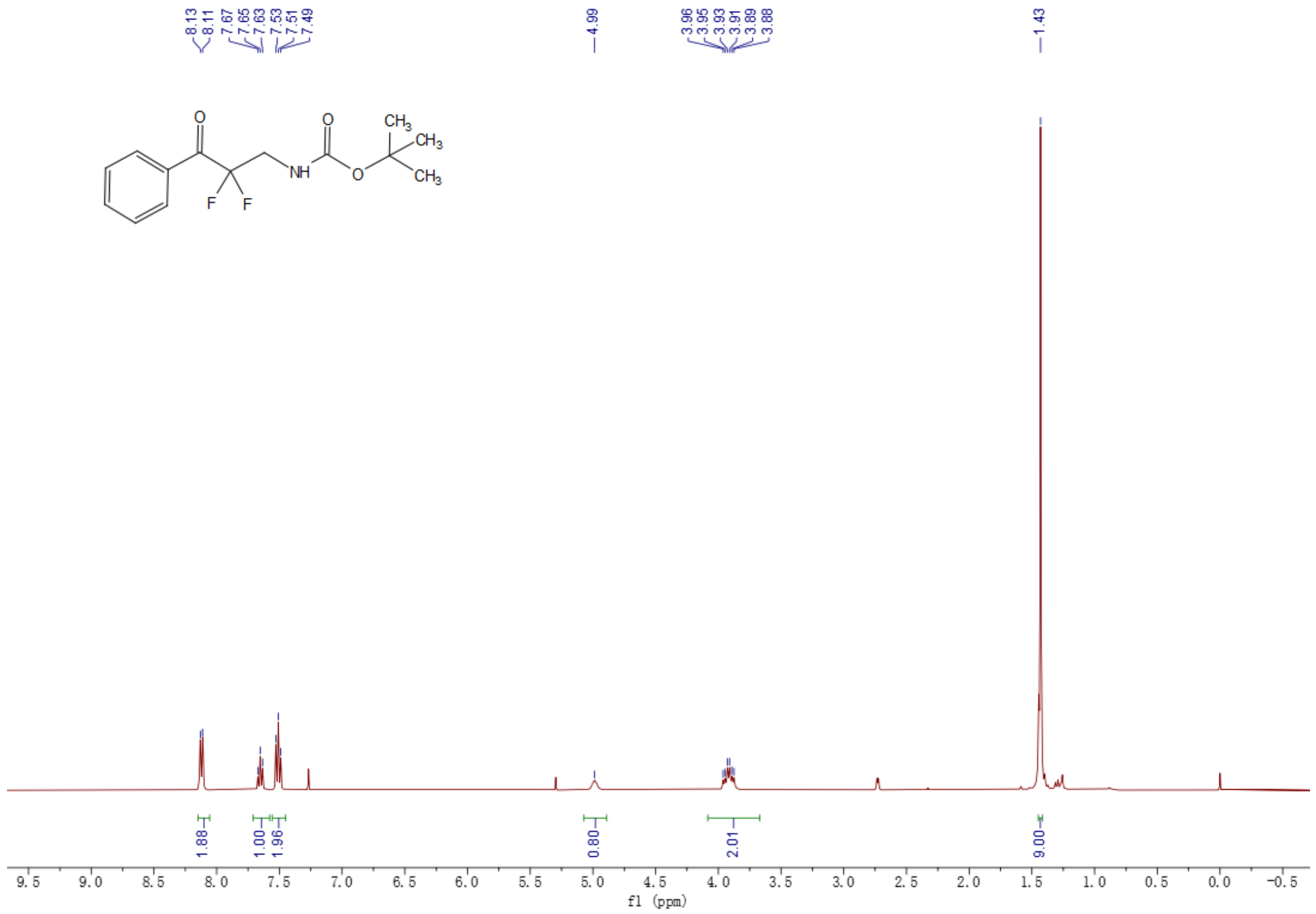

^{19}F NMR spectrum (376 MHz, $CDCl_3$) of compound **3f**

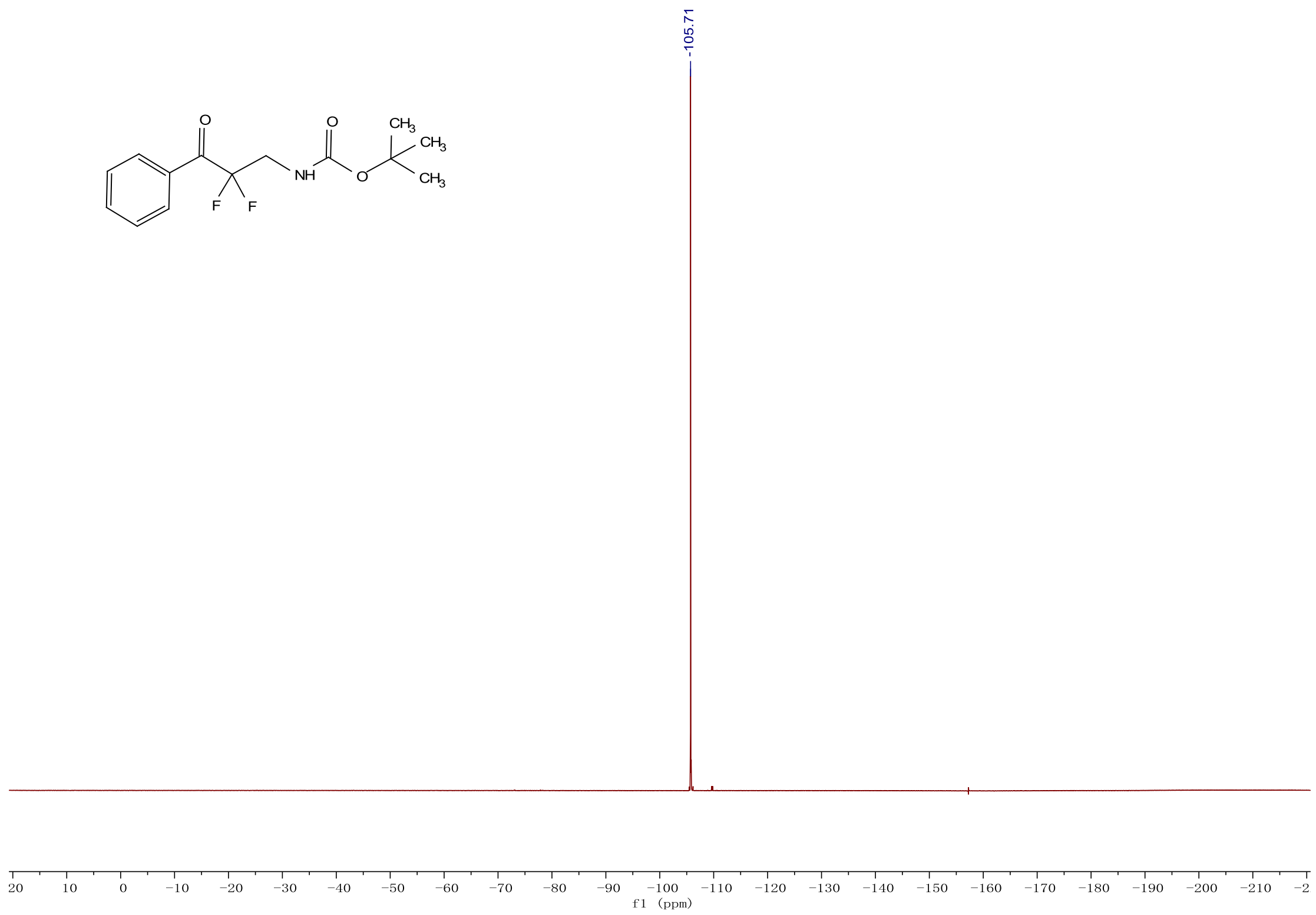

^{13}C NMR spectrum (101 MHz, $CDCl_3$) of compound **3f**

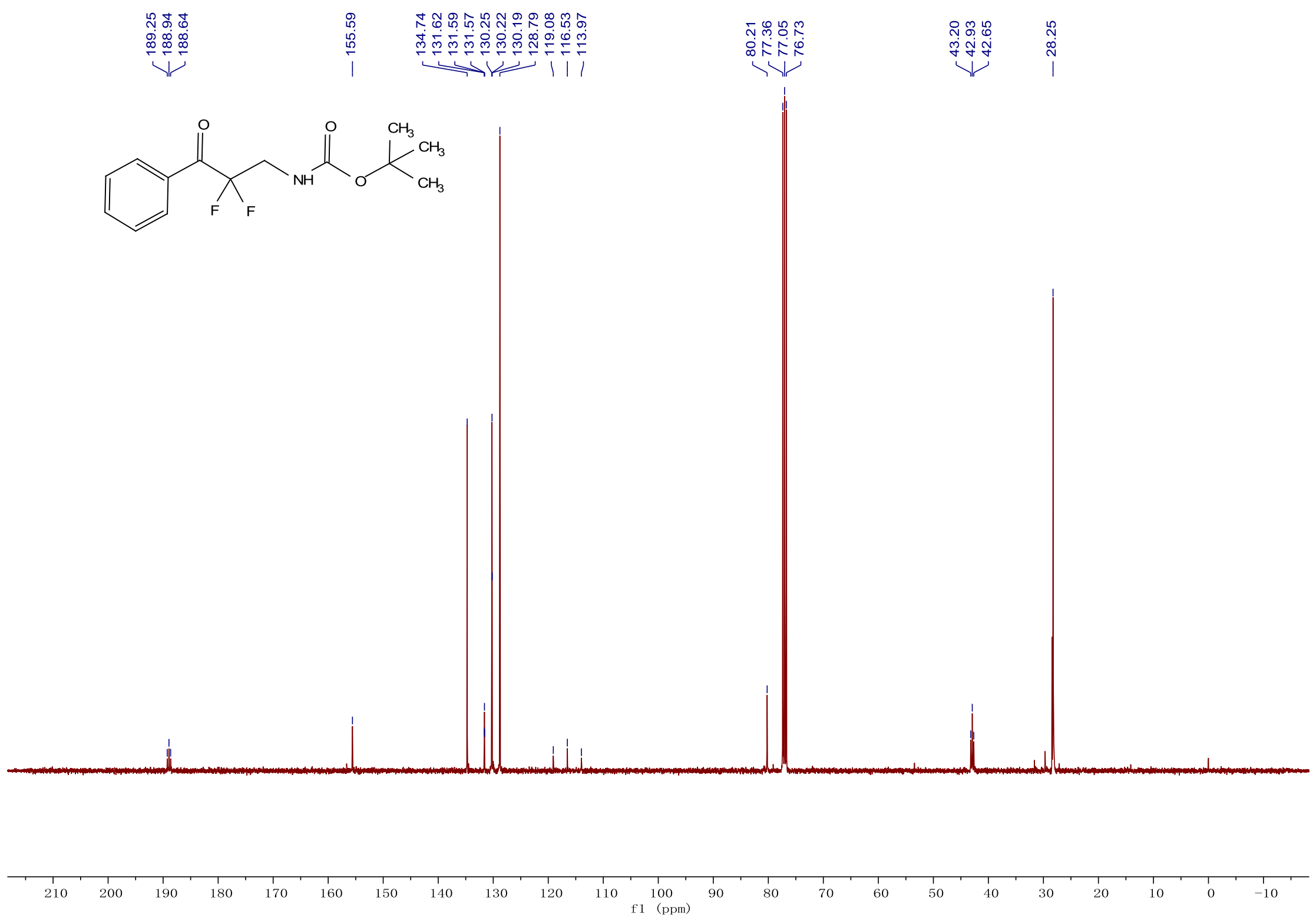

2-cyclobutyl-2,2-difluoro-1-phenylethan-1-one (3g)

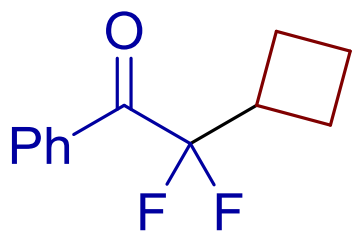

The product (29.1 mg, 69% yield) as colorless oil was purified with silica gel chromatography (petroleum ether/ ethyl acetate = 20/1). **^{1}H NMR** (400 MHz, Chloroform-*d*) δ 8.10 (d, *J* = 7.2 Hz, 2H), 7.64-7.60 (m, 1H), 7.49 (t, *J* =7.6 Hz, 2H), 3.26-3.09 (m, 1H), 2.28-2.18 (m, 2H), 2.14-2.06 (m, 2H), 2.03-1.96 (m, 1H), 1.94-1.86 (m, 1H). **^{19}F NMR** (376 MHz, Chloroform-*d*) δ -108.67 (s, 2F). **^{13}C NMR** (101 MHz, Chloroform-*d*) δ 189.5 (t, *J* = 31.3 Hz), 134.2, 132.4 (t, *J* = 3.0 Hz), 130.1 (t, *J* = 3.0 Hz), 128.6, 119.2 (t, *J* = 252.5 Hz), 37.4 (t, *J* = 24.2 Hz), 21.4 (t, *J* = 5.0 Hz), 17.9. **HRMS** (EI-TOF) m/z: $[M+Na]^+$ Calcd. for $C_{12}H_{12}F_2ONa$ 233.0748; Found 233.0748.

^{1}H NMR spectrum (400 MHz, $CDCl_3$) of compound **3g**

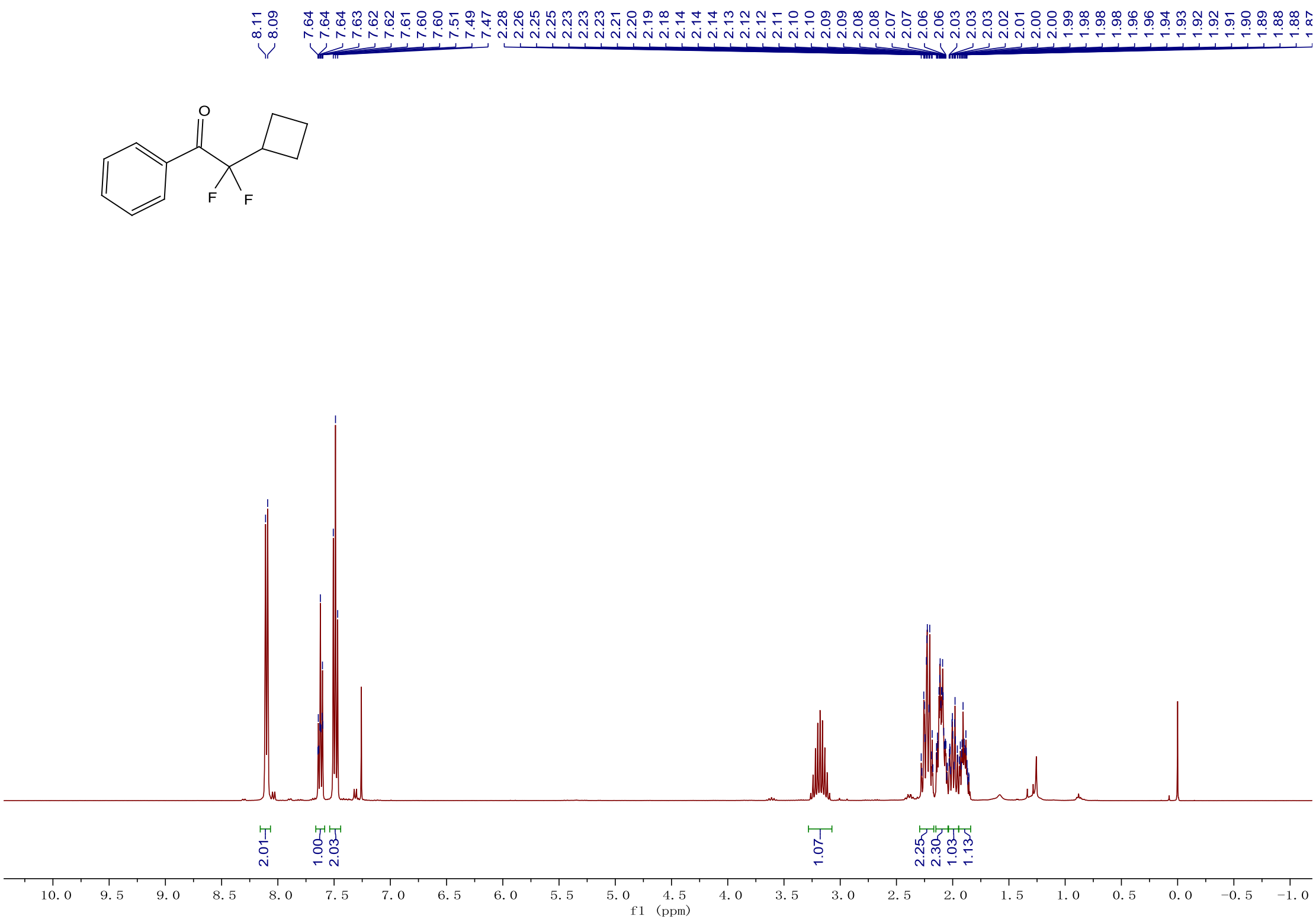

^{19}F NMR spectrum (376 MHz, $CDCl_3$) of compound **3g**

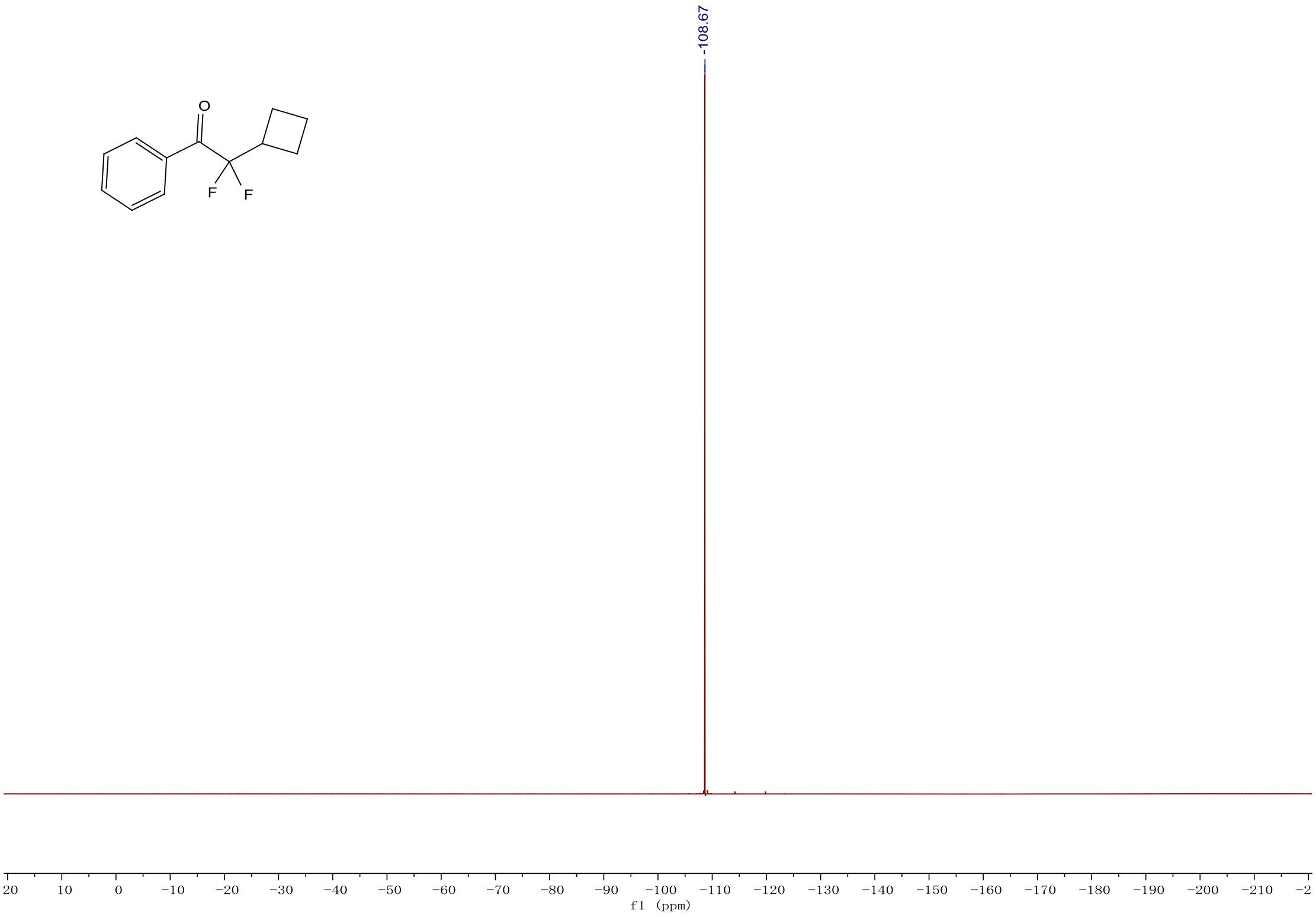

^{13}C NMR spectrum (101 MHz, $CDCl_3$) of compound **3g**

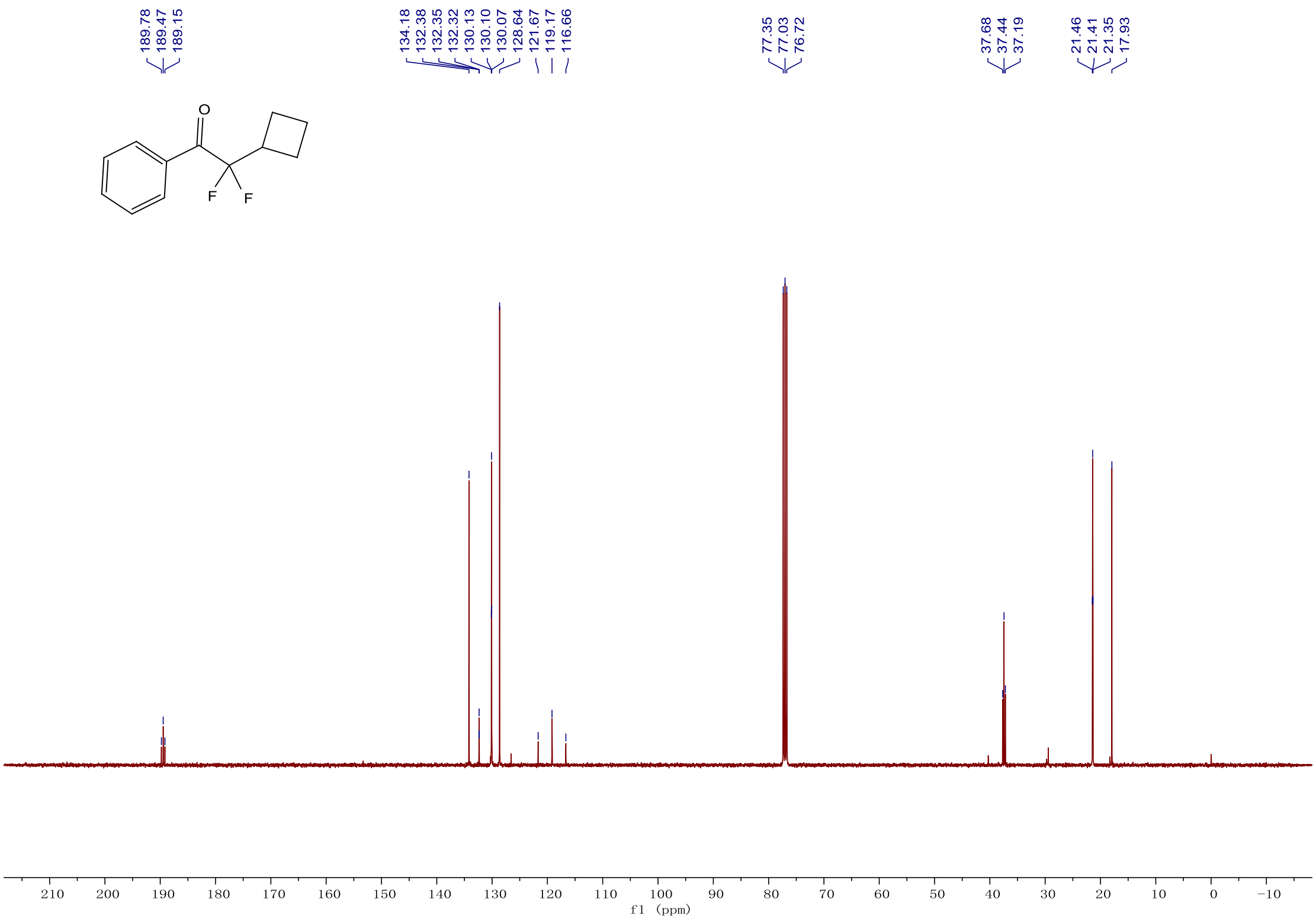

2-cyclopentyl-2,2-difluoro-1-phenylethan-1-one (3h)

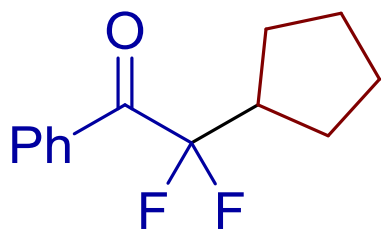

The product (29.2 mg, 65% yield) as colorless oil was purified with silica gel chromatography (petroleum ether/ ethyl acetate = 20/1). **^{1}H NMR** (400 MHz, Chloroform-*d*) δ 8.10 (d, *J* = 8.4 Hz, 2H), 7.62 (t, *J* = 7.6 Hz, 1H), 7.49 (t, *J* = 8.0 Hz, 2H), 2.88-2.71 (m, 1H), 1.86-1.79 (m, 2H), 1.73-1.56 (m, 6H). **^{19}F NMR** (376 MHz, Chloroform-*d*) δ -106.03 (s, 2F). **^{13}C NMR** (101 MHz, Chloroform-*d*) δ 191.3 (t, *J* = 31.3 Hz), 134.9 (t, *J* = 2.0 Hz), 133.7, 130.2 (t, *J* = 4.0 Hz), 128.5, 122.3 (t, *J* = 259.6 Hz), 40.9 (t, *J* = 20.2 Hz), 30.0 (t, *J* = 4.0 Hz), 25.7, 21.0, 16.7 (t, *J* = 4.0Hz). **HRMS** (EI-TOF) m/z: [M+Na]$^+$ Calcd. for $C_{13}H_{14}F_2ONa$ 247.0905; Found 247.0904.

^{1}H NMR spectrum (400 MHz, $CDCl_3$) of compound **3h**

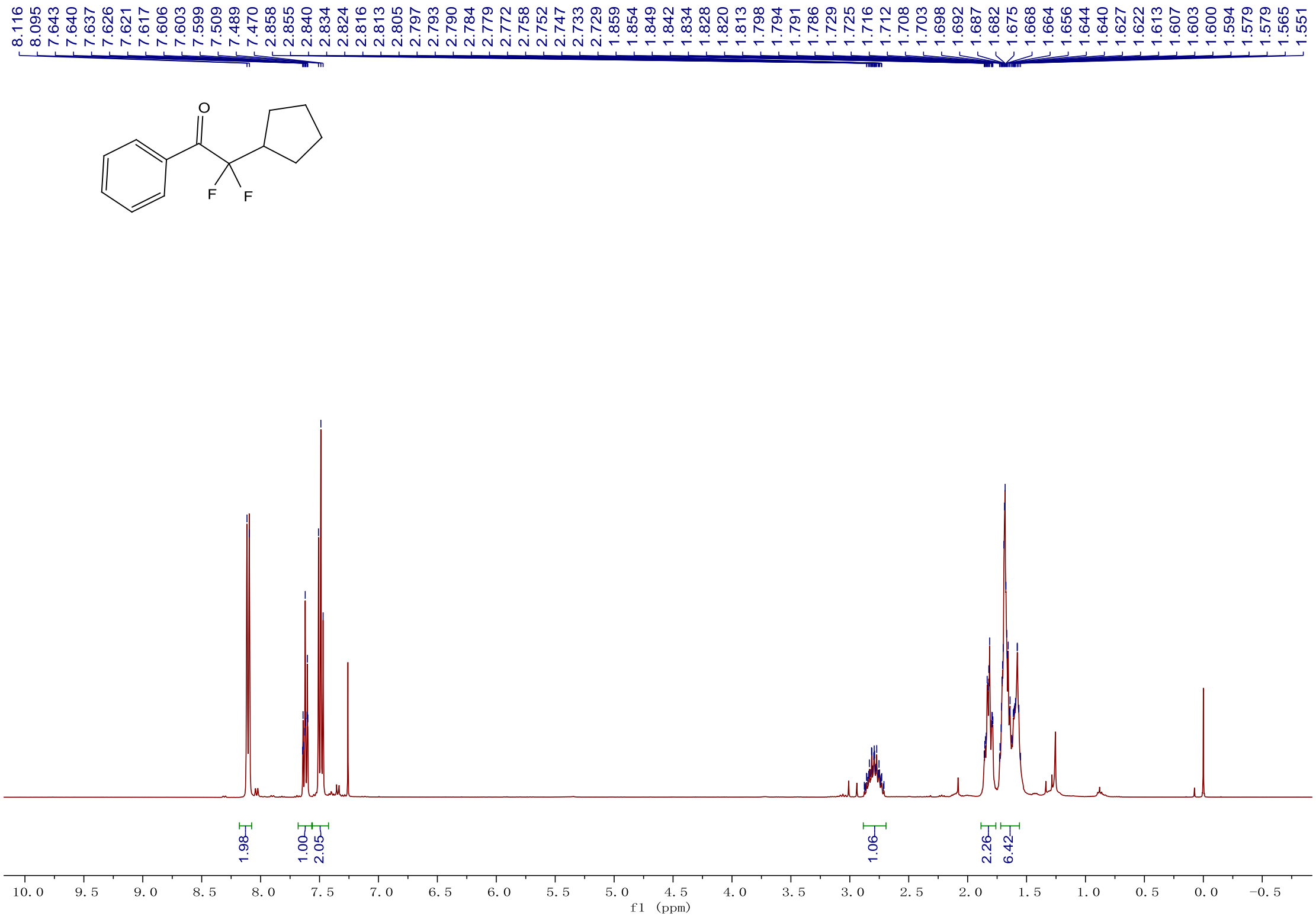

^{19}**F NMR** spectrum (376 MHz, $CDCl_3$) of compound **3h**

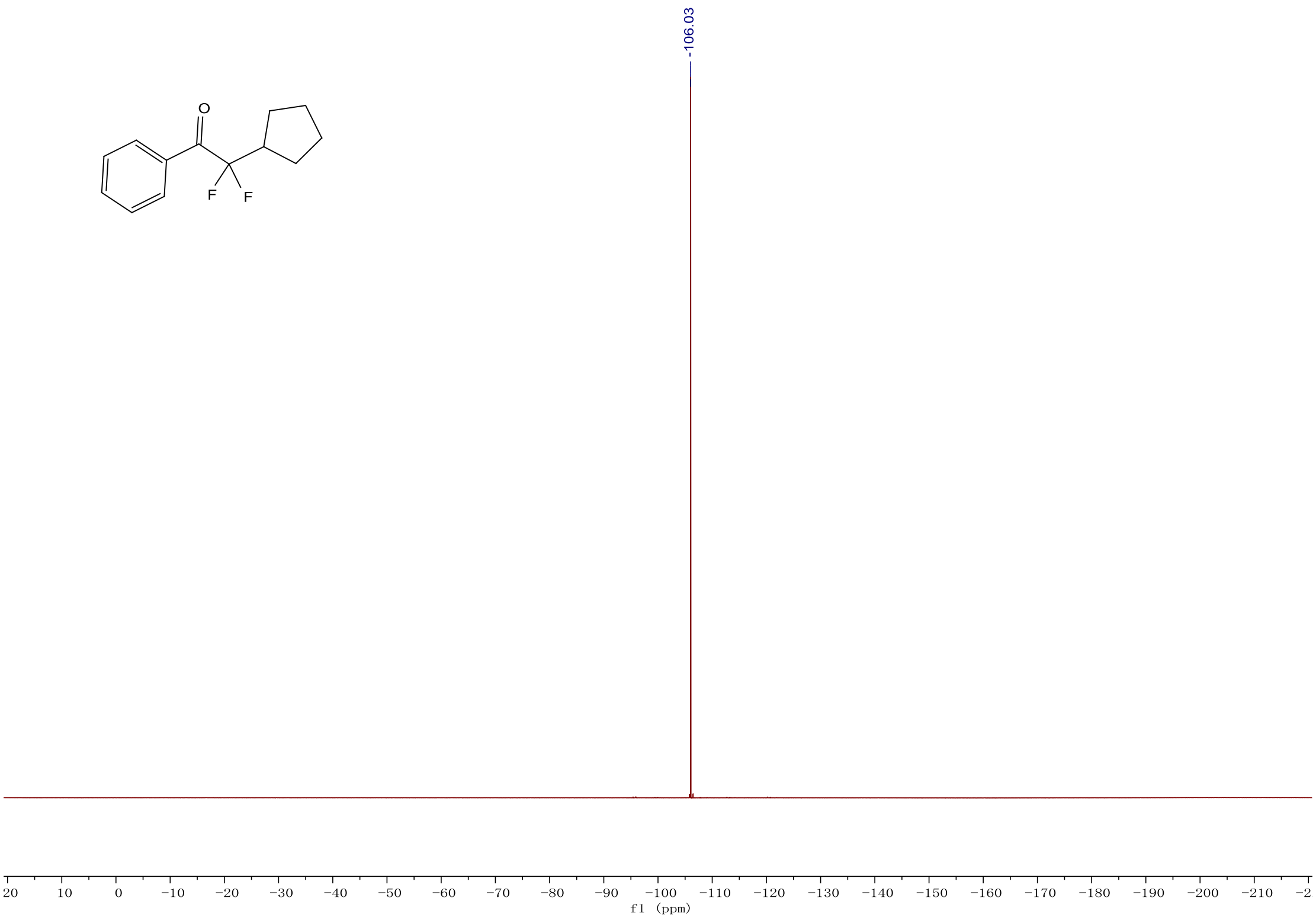

^{13}**C NMR** spectrum (101 MHz, $CDCl_3$) of compound **3h**

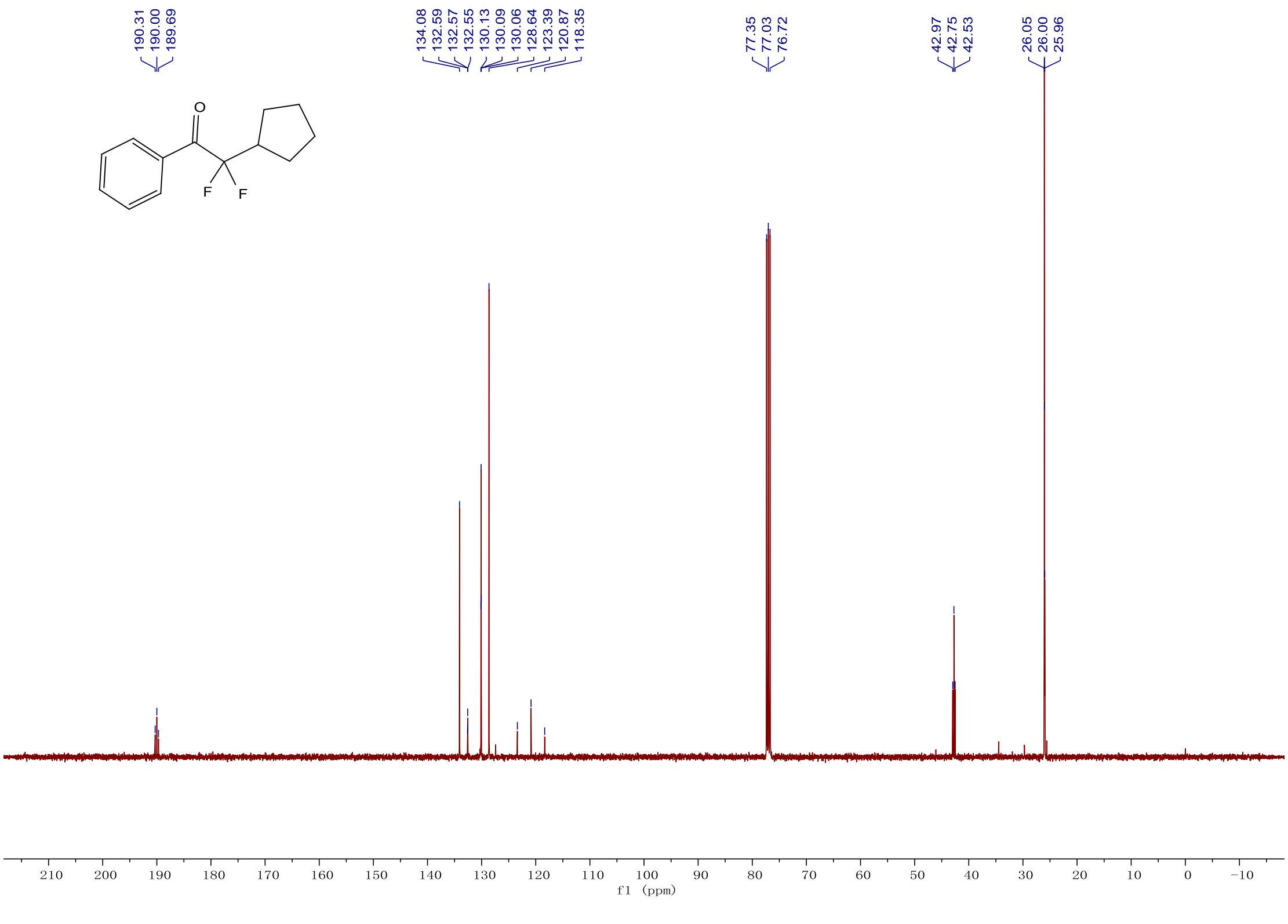

2-(2,3-dihydro-1*H*-inden-2-yl)-2,2-difluoro-1-phenylethan-1-one (3i)

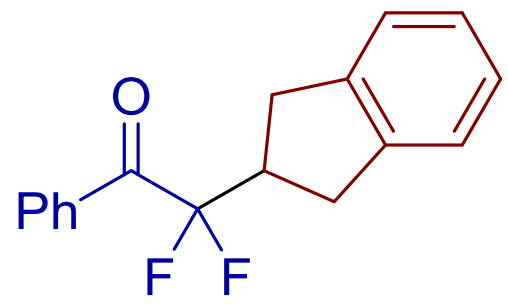

The product (27.7 mg, 51% yield) as white solid was purified with silica gel chromatography (petroleum ether/ ethyl acetate = 20/1). ^{1}**H NMR** (400 MHz, Chloroform-*d*) δ 8.15 (d, *J* = 7.6 Hz, 2H), 7.64 (t, *J* = 7.2 Hz, 1H), 7.50 (t, *J* = 7.6 Hz, 2H), 7.22-7.15 (m, 4H), 3.52-3.34 (m, 1H), 3.22-3.12 (m, 4H).^{19}**F NMR** (376 MHz, Chloroform-*d*) δ -105.40 (s, 2F). ^{13}**C NMR** (101 MHz, Chloroform-*d*) δ 189.4 (t, *J* = 32.3 Hz), 141.3, 134.3, 132.3 (t, *J* = 3.0 Hz), 130.2 (t, *J* = 4.0 Hz), 128.7, 126.7, 124.4, 120.3 (t, *J* = 255.5 Hz), 42.5 (t, *J* = 22.2 Hz), 32.8 (t, *J* = 5.0 Hz). **HRMS** (EI-TOF) m/z: $[M+Na]^+$ Calcd. for $C_{17}H_{14}F_2NaO$ 295.0905; Found 295.0914.

^{1}**H NMR** spectrum (400 MHz, $CDCl_3$) of compound **3i**

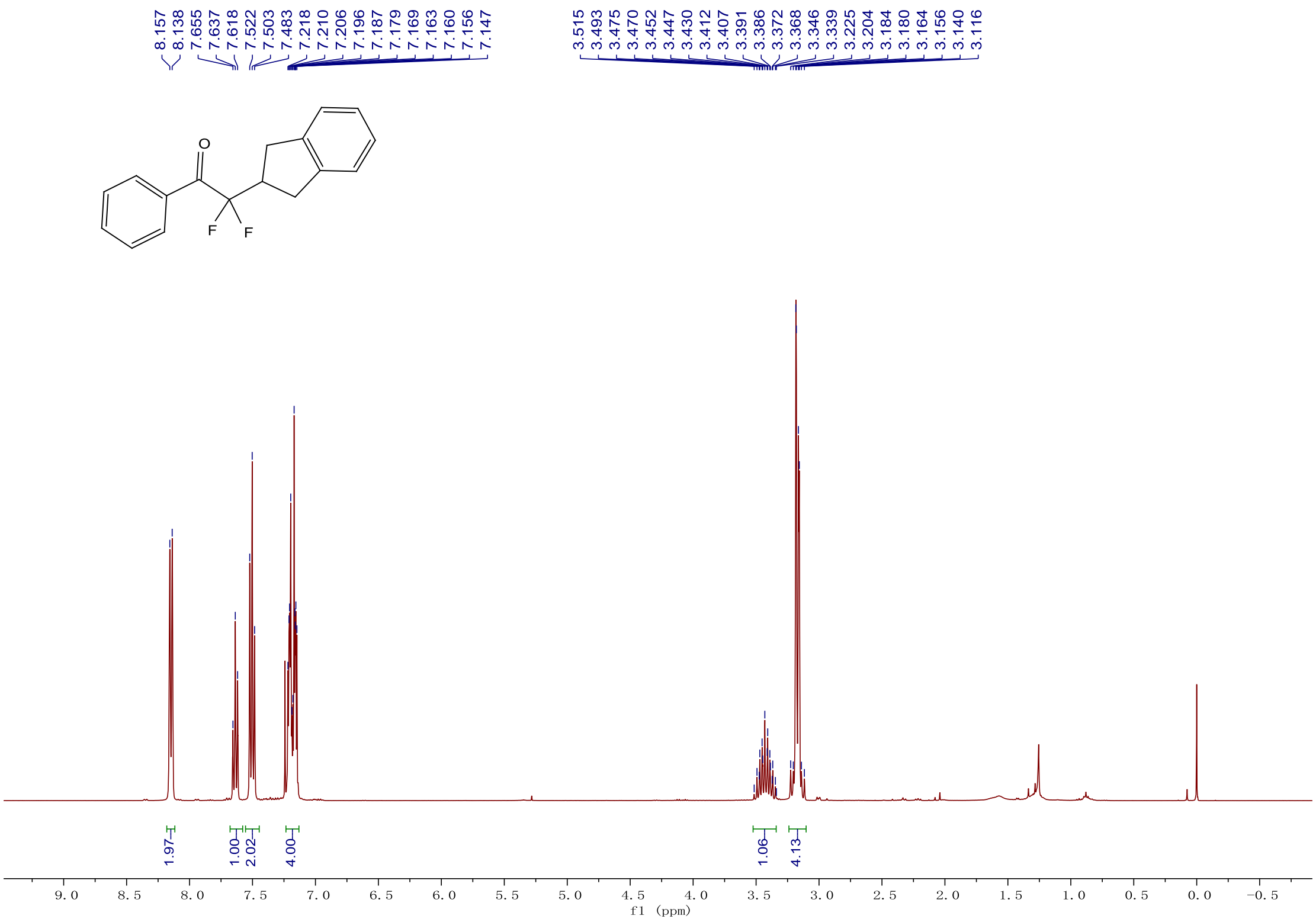

^{19}F NMR spectrum (376 MHz, $CDCl_3$) of compound **3i**

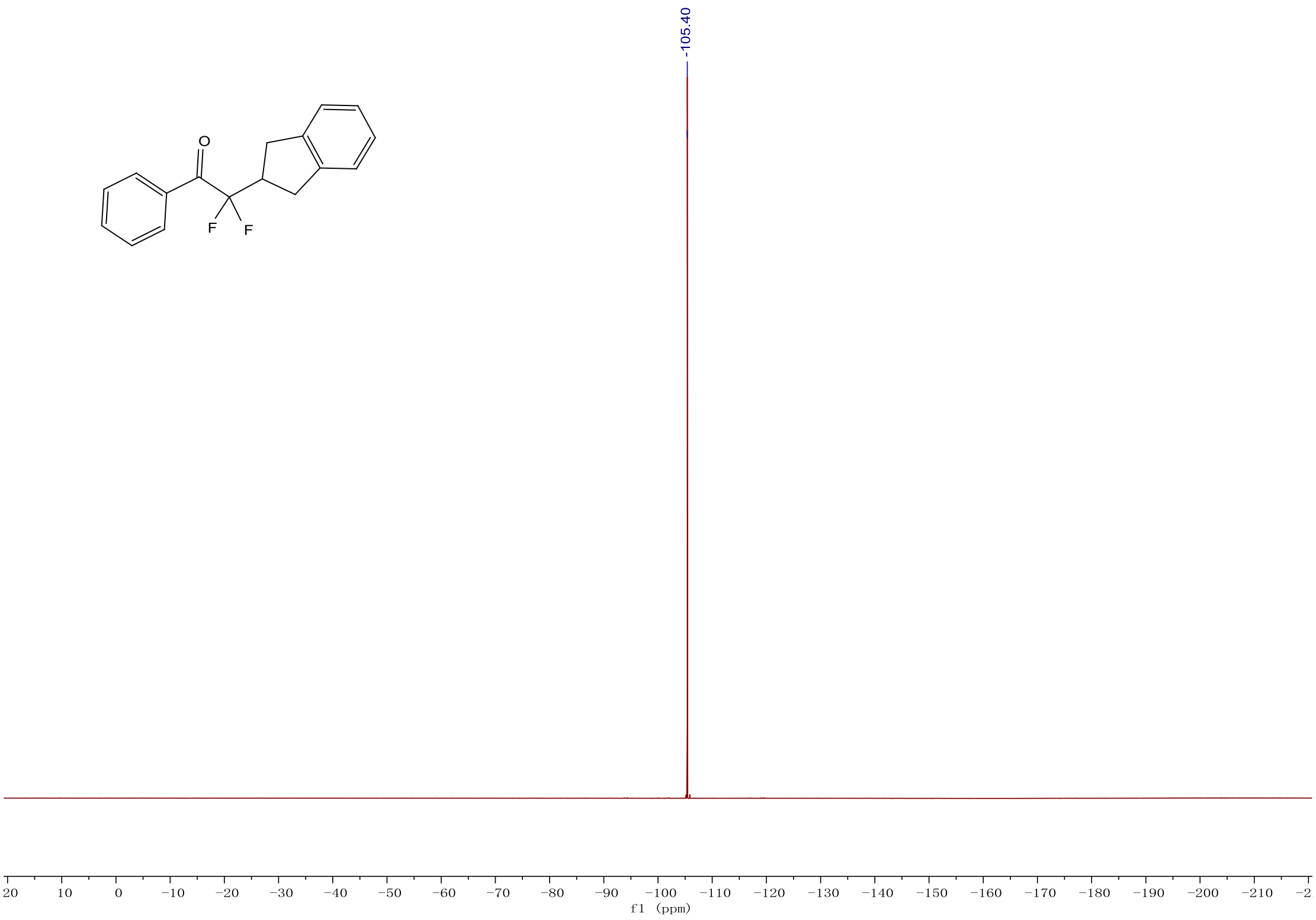

^{13}C NMR spectrum (101 MHz, $CDCl_3$) of compound **3i**

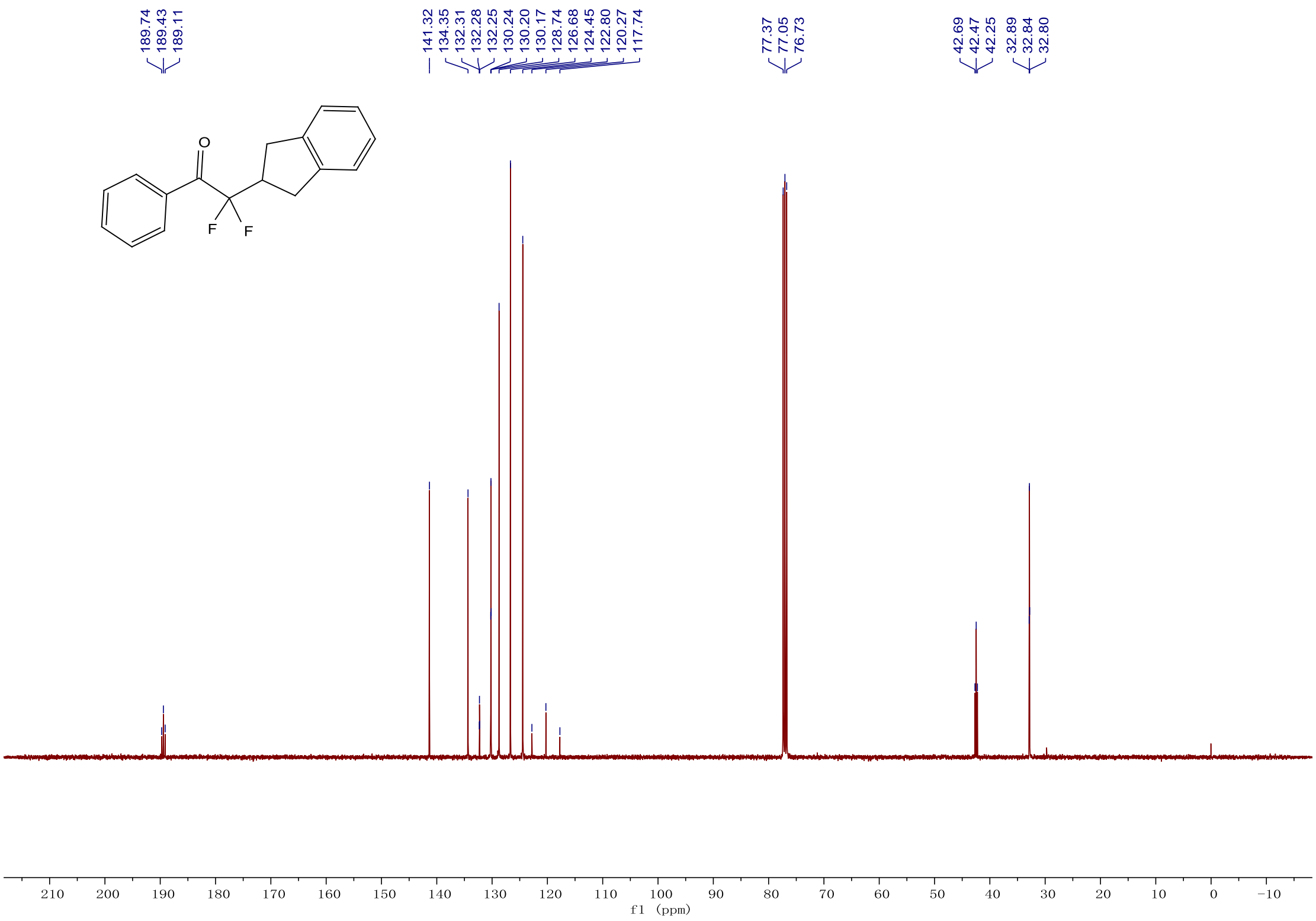

2-((4-(tert-butyl)phenyl)thio)-2,2-difluoro-1-phenylethan-1-one (3j)

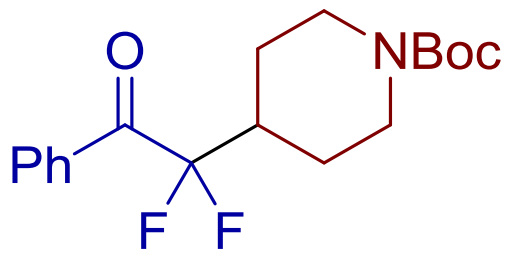

The product (46.1 mg, 68% yield) as white solid was purified with silica gel chromatography (petroleum ether/ ethyl acetate = 10/1). **^{1}H NMR** (400 MHz, Chloroform-*d*) δ 8.09 (d, *J* = 7.6 Hz, 2H), 7.67-7.62 (m, 1H), 7.50 (t, *J* = 8.0 Hz, 2H), 4.21 (s, 2H), 2.70 (t, *J* = 12.4 Hz, 2H), 2.53-2.38 (m, 1H), 1.79 (d, *J* = 13.2 Hz, 2H), 1.56-1.48 (m, 2H), 1.46 (s, 9H). **^{19}F NMR** (376 MHz, Chloroform-*d*) δ -97.46 (dd, *J* = 3976.8, *J* = 236.9, 2F), -106.90 (d, *J* = 2.6, 2F). **^{13}C NMR** (101 MHz, Chloroform-*d*) δ 189.6 (t, *J* = 30.3 Hz), 154.6, 134.4, 132.5 (t, *J* = 3.0 Hz), 130.1 (t, *J* = 4.0 Hz), 128.8, 119.5 (t, *J* = 256.5 Hz), 79.7, 43.1, 40.3 (t, *J* = 22.2 Hz), 28.4, 24.3. **HRMS** (EI-TOF) m/z: $[M+Na]^+$ Calcd. for $C_{18}H_{23}F_2NaO_3$ 362.1538; Found 362.1547.

^{1}H NMR spectrum (400 MHz, $CDCl_3$) of compound **3j**

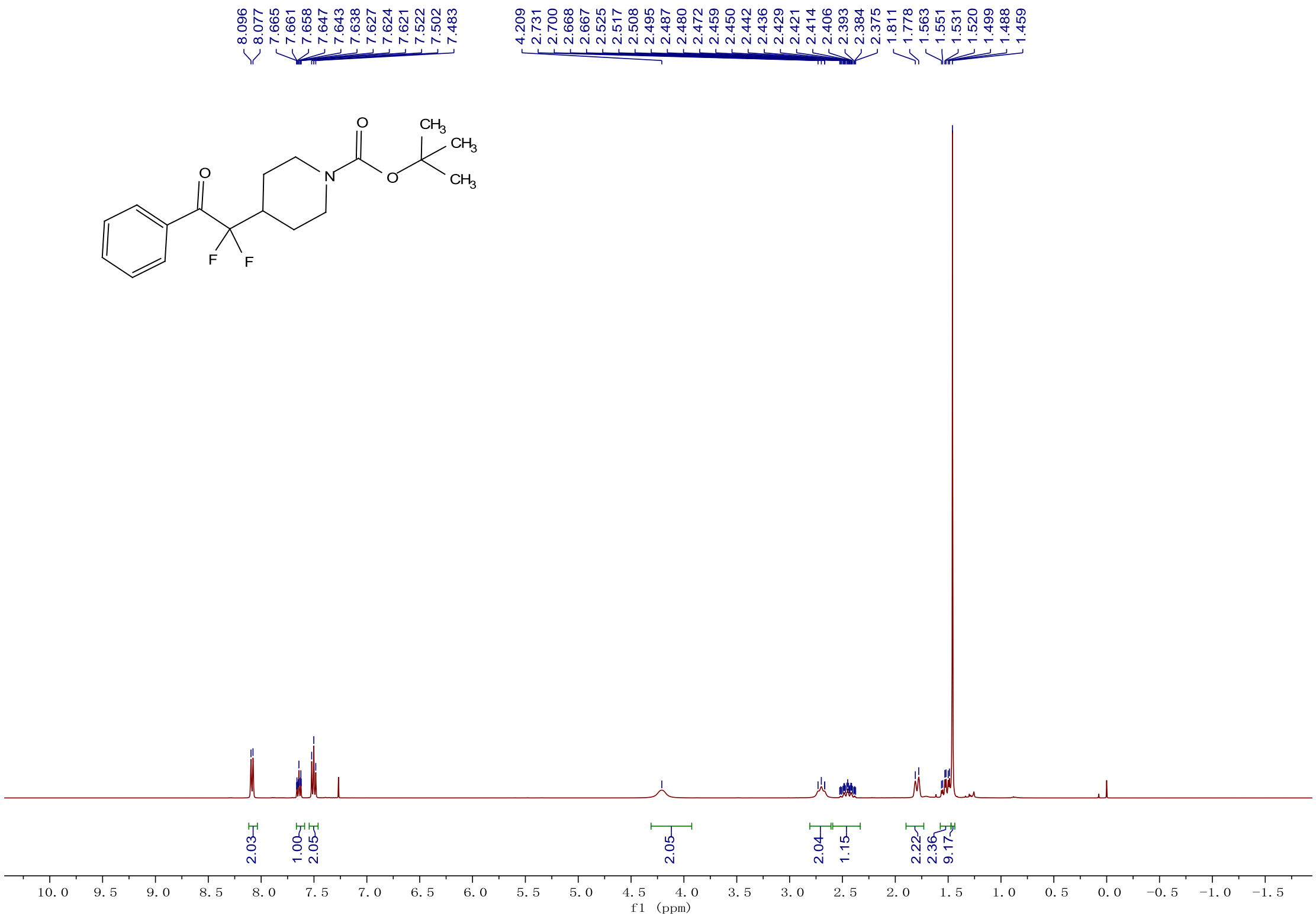

^{19}F NMR spectrum (376 MHz, $CDCl_3$) of compound **3j**

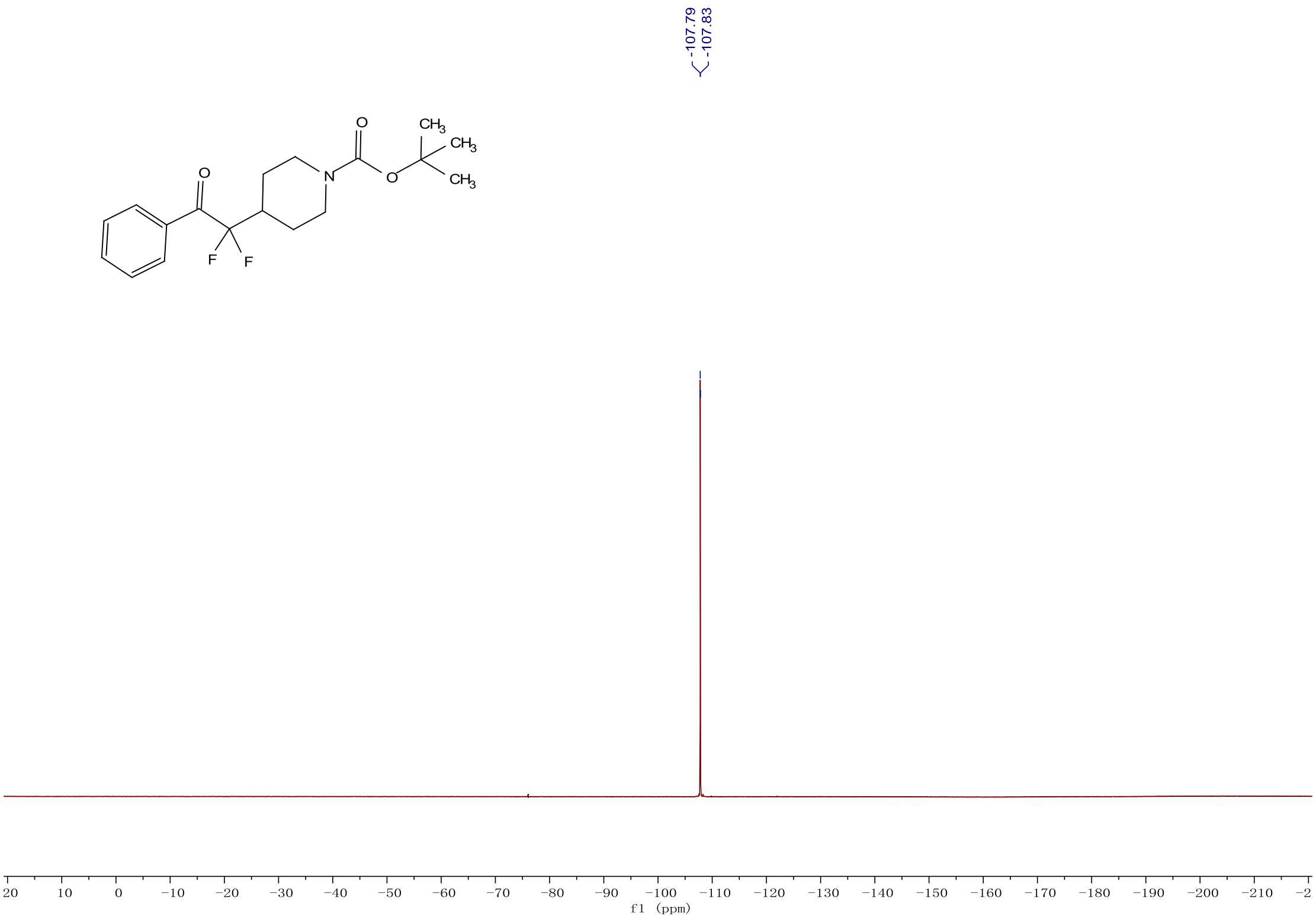

^{13}C NMR spectrum (101 MHz, $CDCl_3$) of compound **3j**

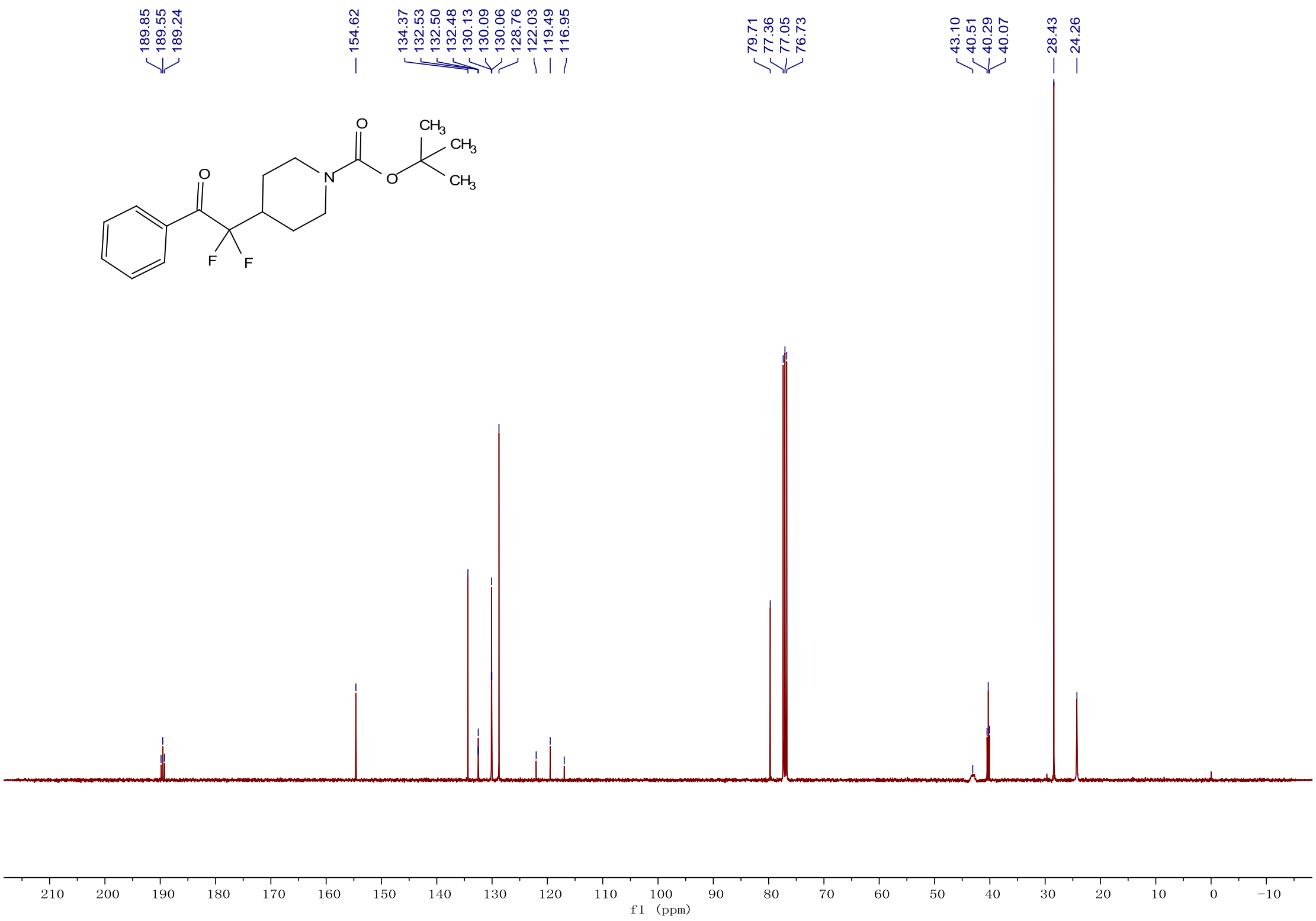

2-(4,4-difluorocyclohexyl)-2,2-difluoro-1-phenylethan-1-one (3k)

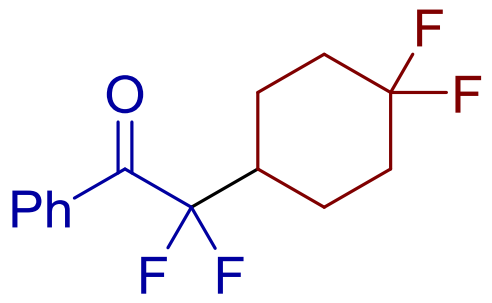

The product (35.7 mg, 65% yield) as white solid was purified with silica gel chromatography (petroleum ether/ ethyl acetate = 20/1). **^{1}H NMR** (400 MHz, Chloroform-*d*) δ 8.09 (dd, *J* = 8.8 Hz, *J* =0.8 Hz, 2H), 7.67-7.62 (m, 1H), 7.52-7.48 (m, 2H), 2.45-2.34 (m, 1H), 2.32-2.16 (m, 2H), 1.97-1.93 (m, 2H), 1.83-1.62 (m, 4H). **^{19}F NMR** (376 MHz, Chloroform-*d*) δ -77.42 (d, *J* = 11.28 Hz, 2F). **^{13}C NMR** (101 MHz, Chloroform-*d*) δ 189.4 (t, *J* = 31.3 Hz), 134.4, 132.4 (t, *J* = 2.0 Hz), 130.1 (t, *J* = 3.0 Hz), 128.8, 122.6 (t, *J* = 243.4 Hz), 119.6 (td, *J* = 256.5 Hz, *J* = 2.0 Hz), 39.72 (td, *J* = 22.2 Hz, *J* = 2.0 Hz), 32.77 (dd, *J*= 23.2 Hz), 21.53-21.34 (m). **HRMS** (EI-TOF) m/z: $[M+Na]^{+}$ Calcd. for $C_{14}H_{14}F_{4}NaO$ 297.0873; Found 297.0879.

^{1}H NMR spectrum (400 MHz, $CDCl_3$) of compound **3k**

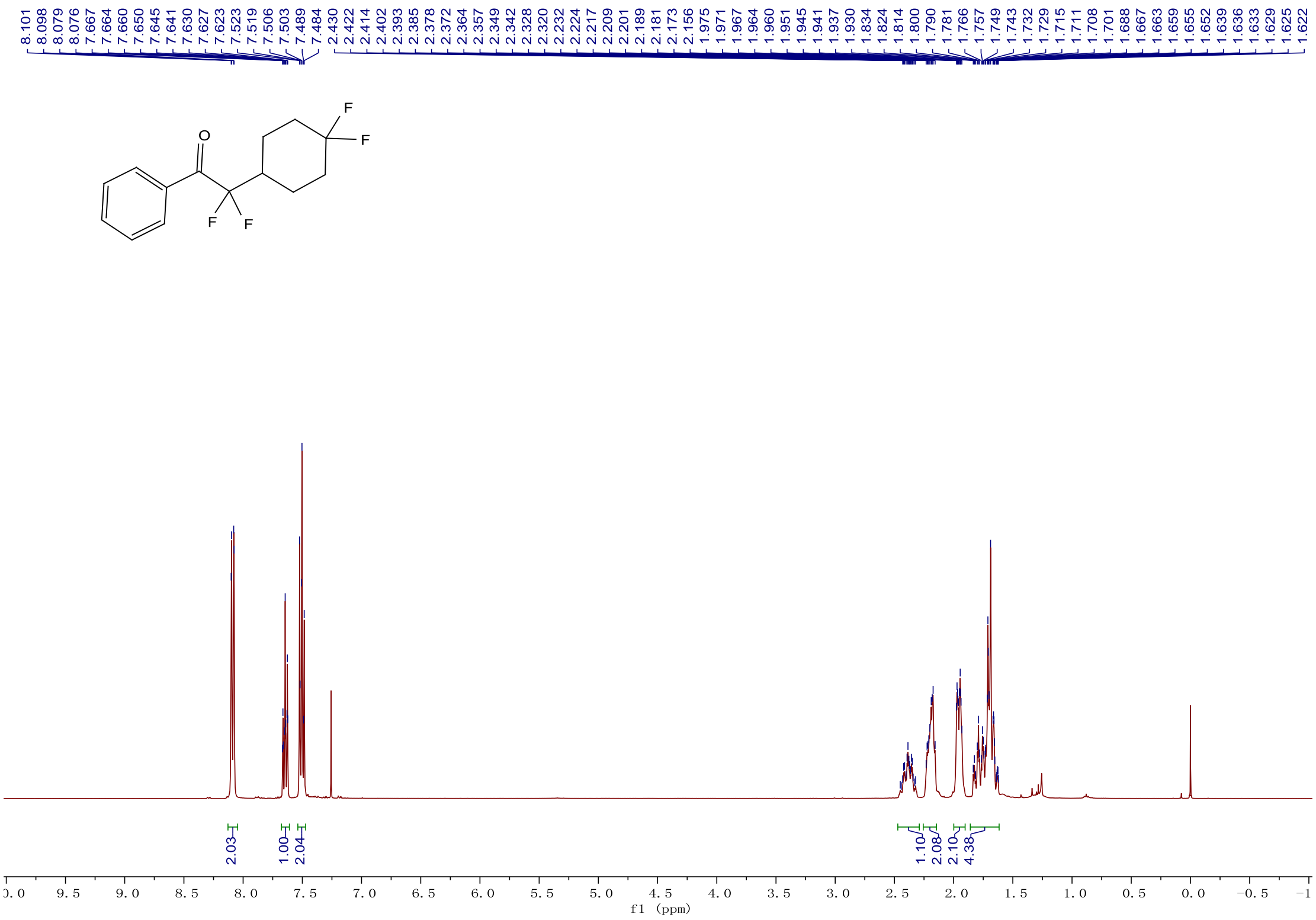

^{19}F NMR spectrum (376 MHz, $CDCl_3$) of compound **3k**

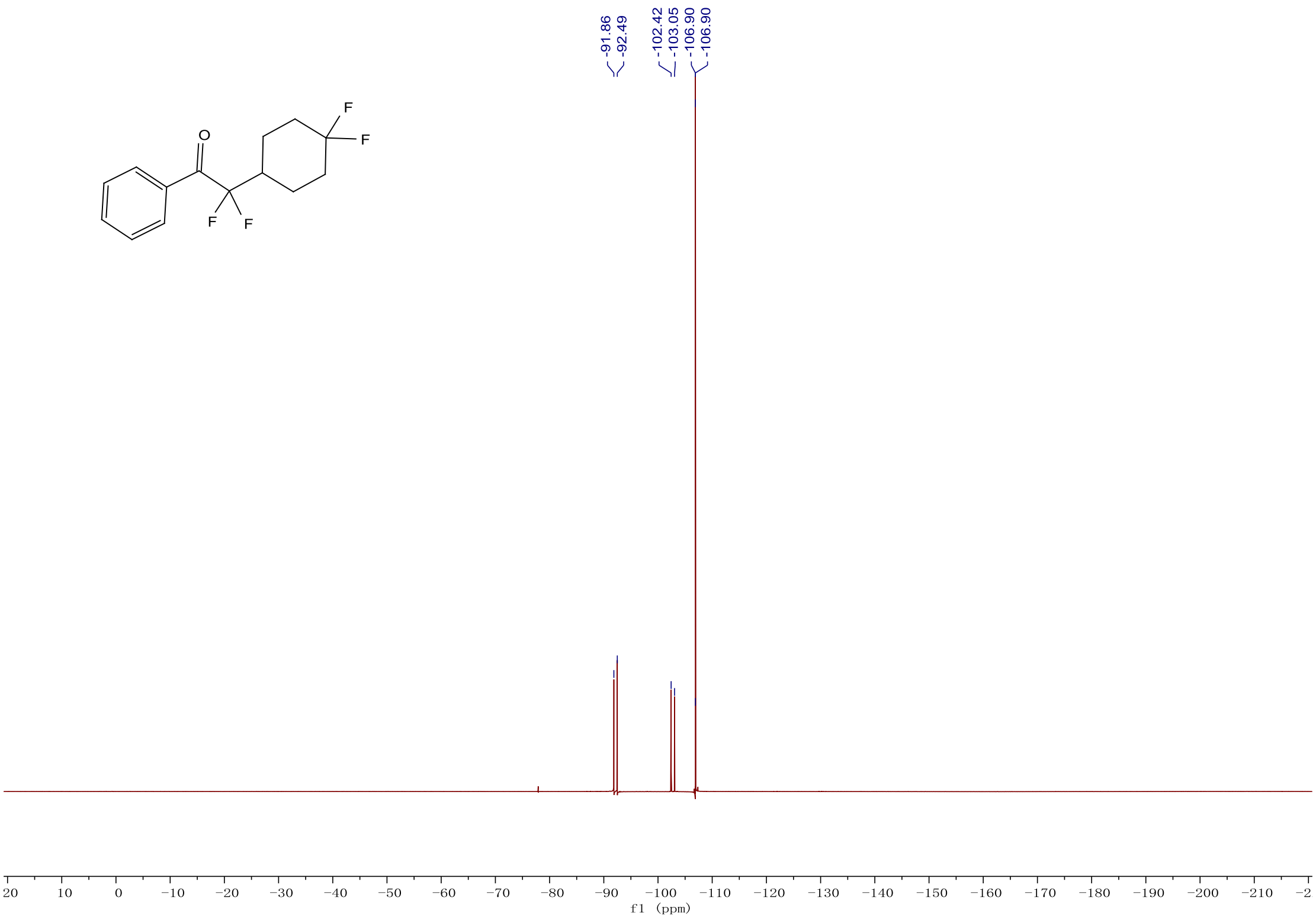

^{13}C NMR spectrum (101 MHz, $CDCl_3$) of compound **3k**

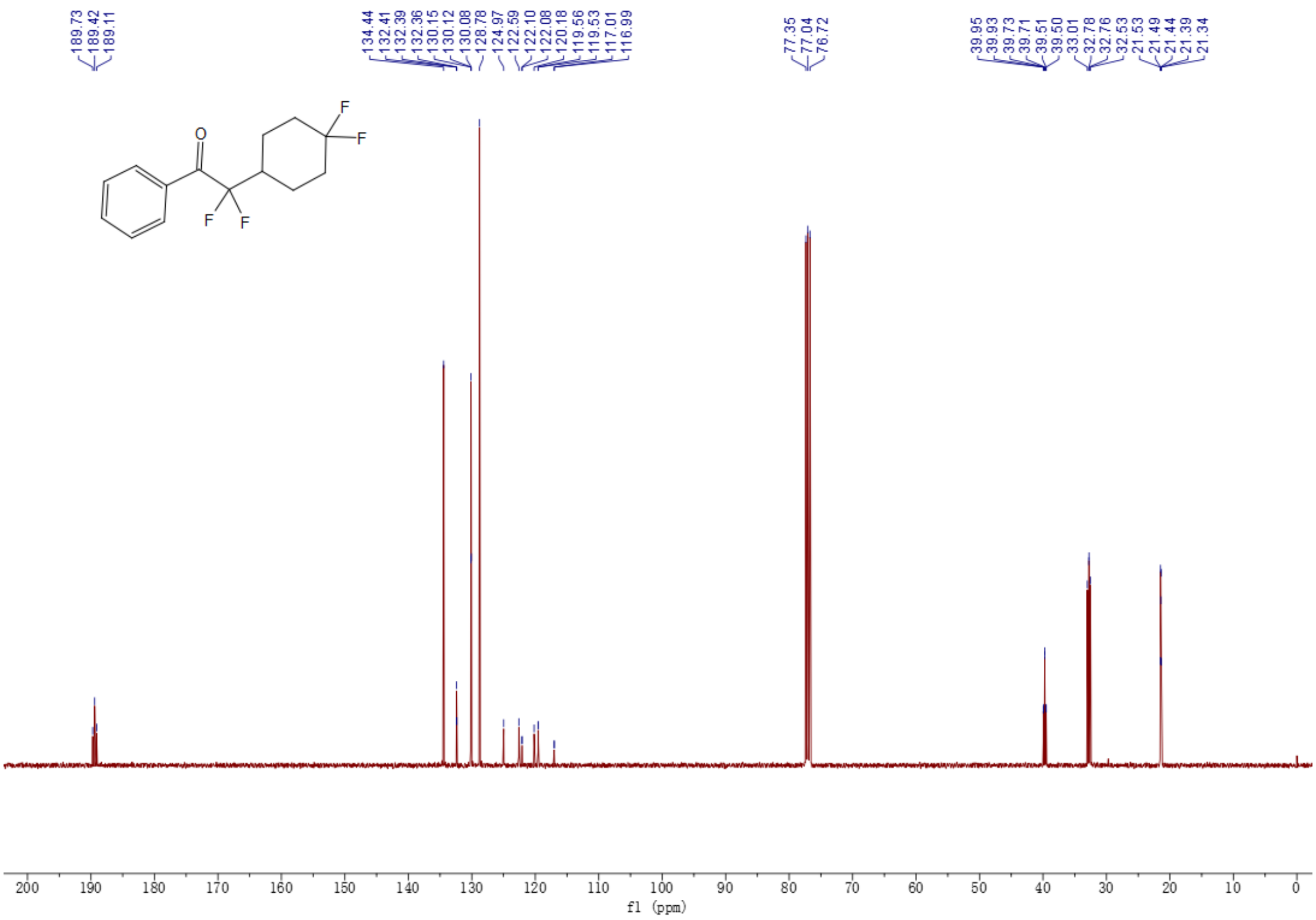

2-cycloheptyl-2,2-difluoro-1-phenylethan-1-one (3l)

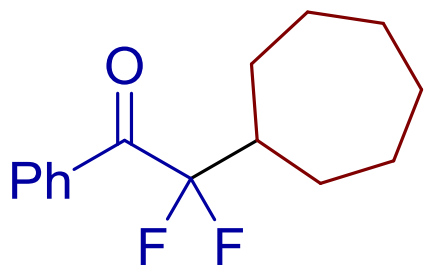

The product (35.3 mg, 70% yield) as colorless oil was purified with silica gel chromatography (petroleum ether/ ethyl acetate = 20/1). **^{1}H NMR** (400 MHz, Chloroform-*d*) δ 8.07 (d, *J* = 8.0 Hz, 2H), 7.62 (t, *J* = 7.2 Hz, 1H), 7.49 (t, *J* = 7.6 Hz, 2H), 2.50-2.35 (m, 1H), 1.88-1.82 (m, 2H), 1.77-1.72 (m, 2H), 1.58-1.43 (m, 8H).**^{19}F NMR** (376 MHz, Chloroform-*d*) δ -106.89 (s, 2F). **^{13}C NMR** (101 MHz, Chloroform-*d*) δ 190.5 (t, *J* = 30.3 Hz), 134.0,133.0 (t, *J* = 2.0 Hz), 130.0 (t, *J* = 4.0 Hz), 128.7, 121.1 (t, *J* = 256.5 Hz), 43.3 (t, *J* = 20.2 Hz), 28.3, 26.5, 26.4(d, *J* = 4.0 Hz). **HRMS** (EI-TOF) m/z: $[M+Na]^+$ Calcd. for $C_{15}H_{18}F_2NaO$ 275.1218; Found 275.1218

^{1}H NMR spectrum (400 MHz, $CDCl_3$) of compound **3l**

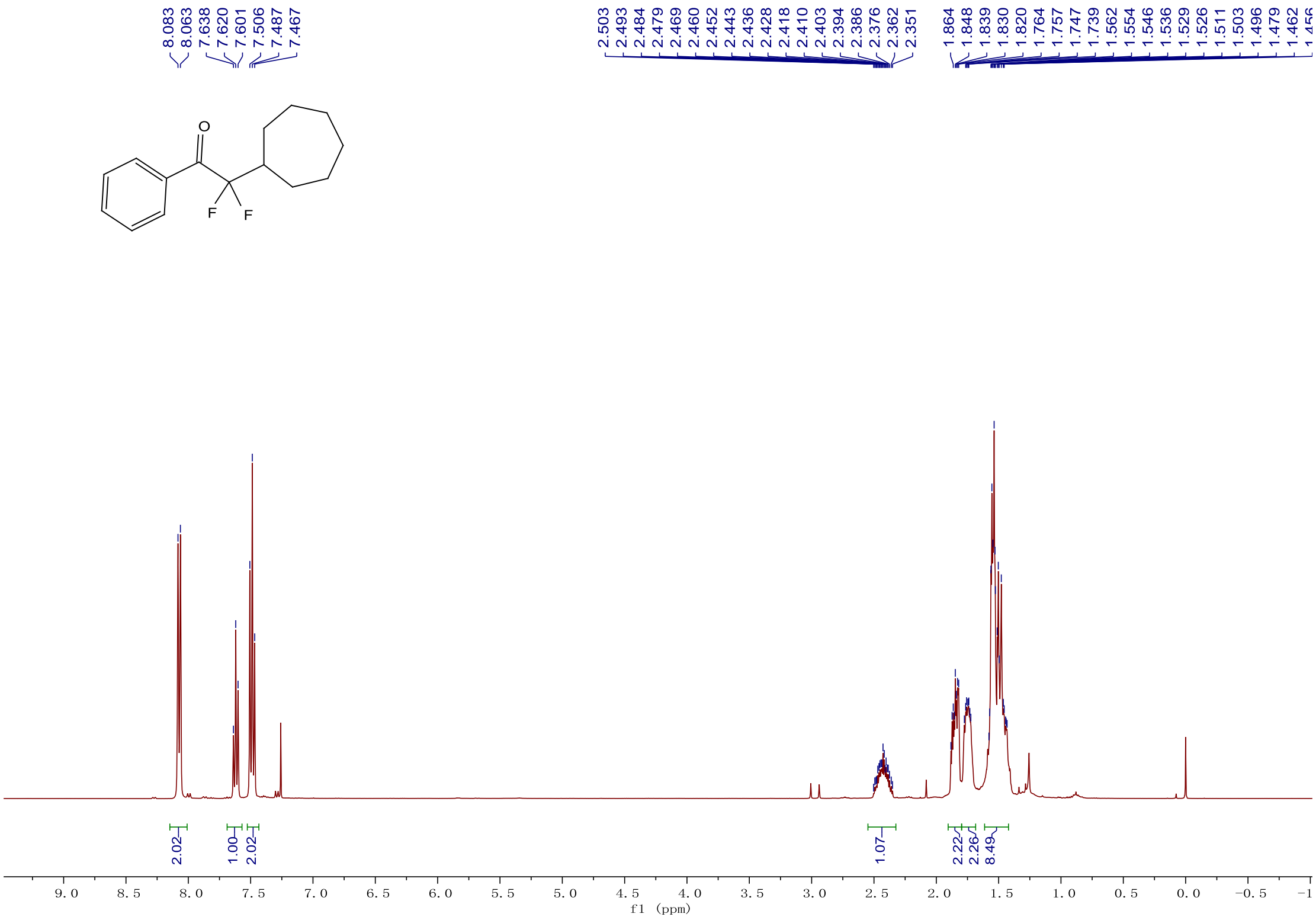

^{19}F NMR spectrum (376 MHz, $CDCl_3$) of compound **3l**

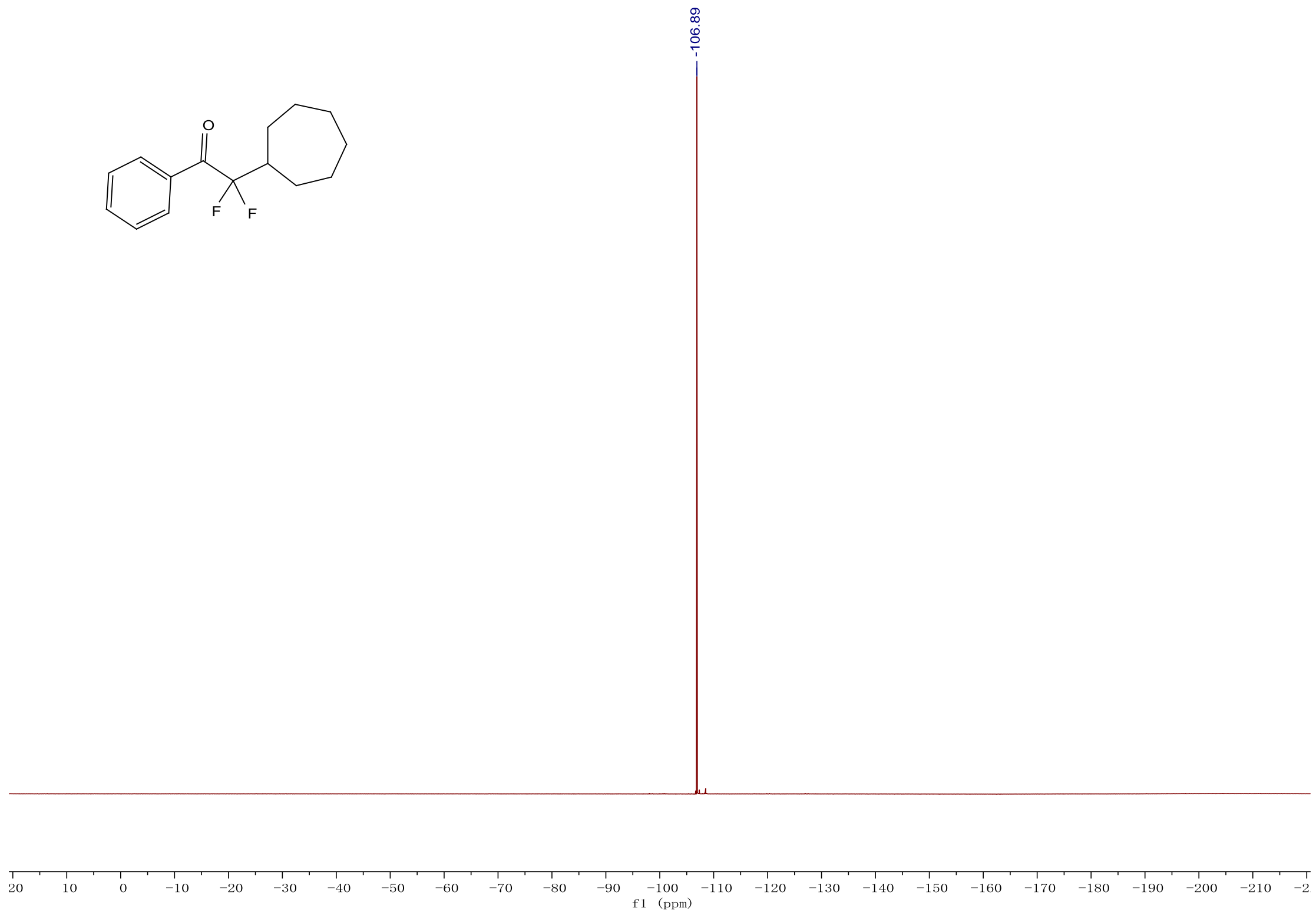

^{13}C NMR spectrum (101 MHz, $CDCl_3$) of compound **3l**

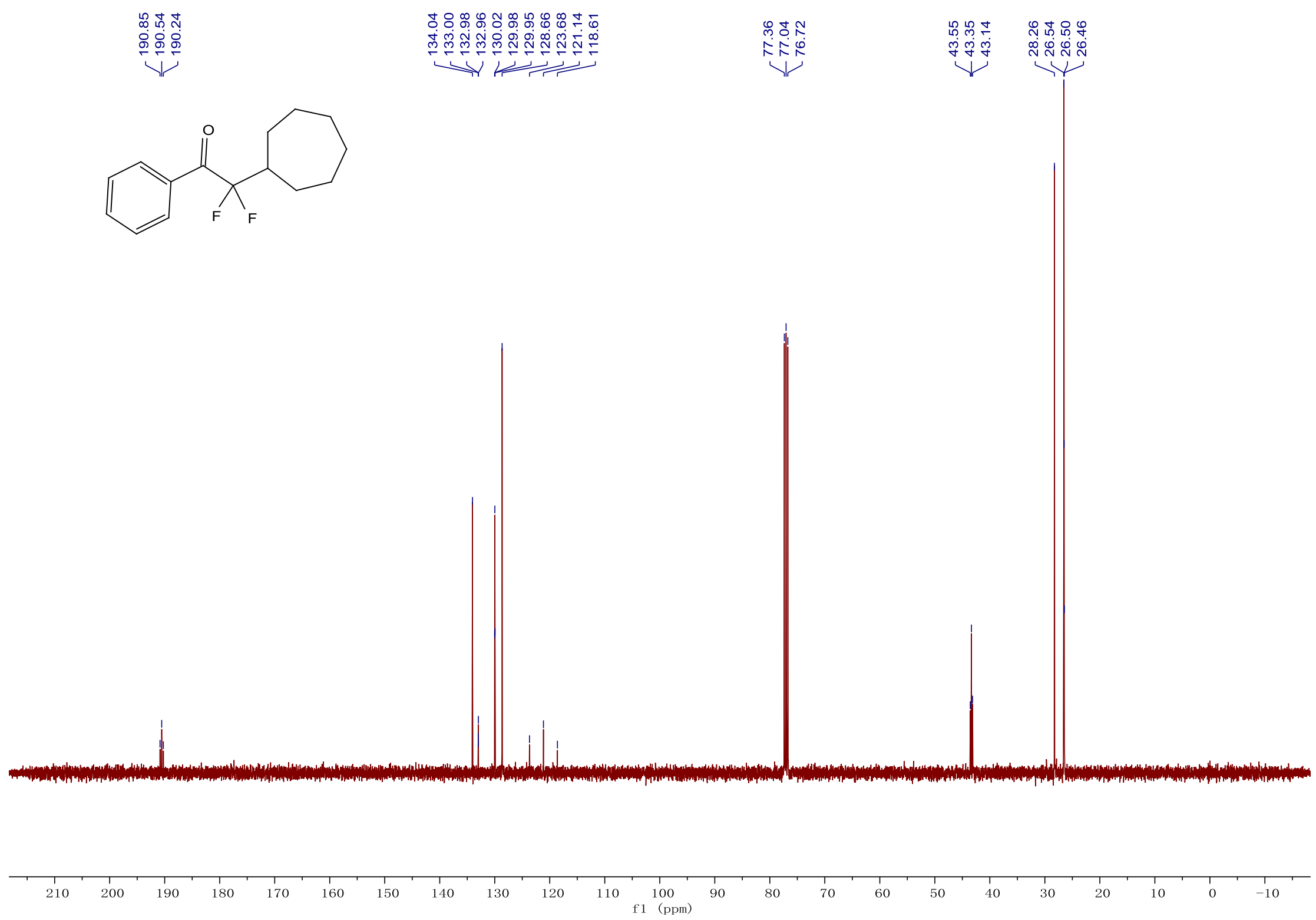

***tert*-butyl (3,3-difluoro-4-oxo-4-phenylbutan-2-yl)carbamate (3m)**

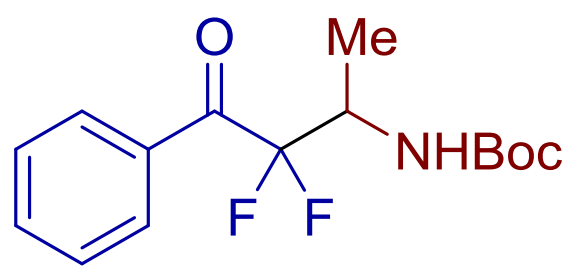

The product (35.3 mg, 59% yield) as white solid was purified with silica gel chromatography (petroleum ether/ ethyl acetate = 10/1). **^{1}H NMR** (400 MHz, Chloroform-*d*) δ 8.10 (d, *J* = 8.0 Hz, 2H), 7.67-7.62 (m, 1H), 7.50 (t, *J* = 8.0 Hz, 2H), 4.85 (d, *J* = 8.0 Hz, 1H), 4.63-4.50 (m, 1H), 1.40 (s, 9H), 1.31 (d, *J* = 8.0 Hz, 3H). **^{19}F NMR** (376 MHz, Chloroform-*d*) δ -108.9-110.5 (m, 2F). **^{13}C NMR** (101 MHz, Chloroform-*d*) δ 189.0 (t, *J* = 29.3 Hz), 154.9, 134.5, 132.3 (t, *J* = 2.0 Hz), 130.0 (t, *J* = 3.0 Hz), 128.8, 117.4 (t, *J* = 258.6 Hz), 80.1, 49.2 (t, *J* = 25.2 Hz), 28.2, 14.7 (t, *J* = 3.0 Hz). **HRMS** (EI-TOF) m/z: $[M+Na]^+$ Calcd. for $C_{15}H_{19}F_2NNaO_3$ 322.1225; Found 322.1233.

^{1}H NMR spectrum (400 MHz, $CDCl_3$) of compound **3m**

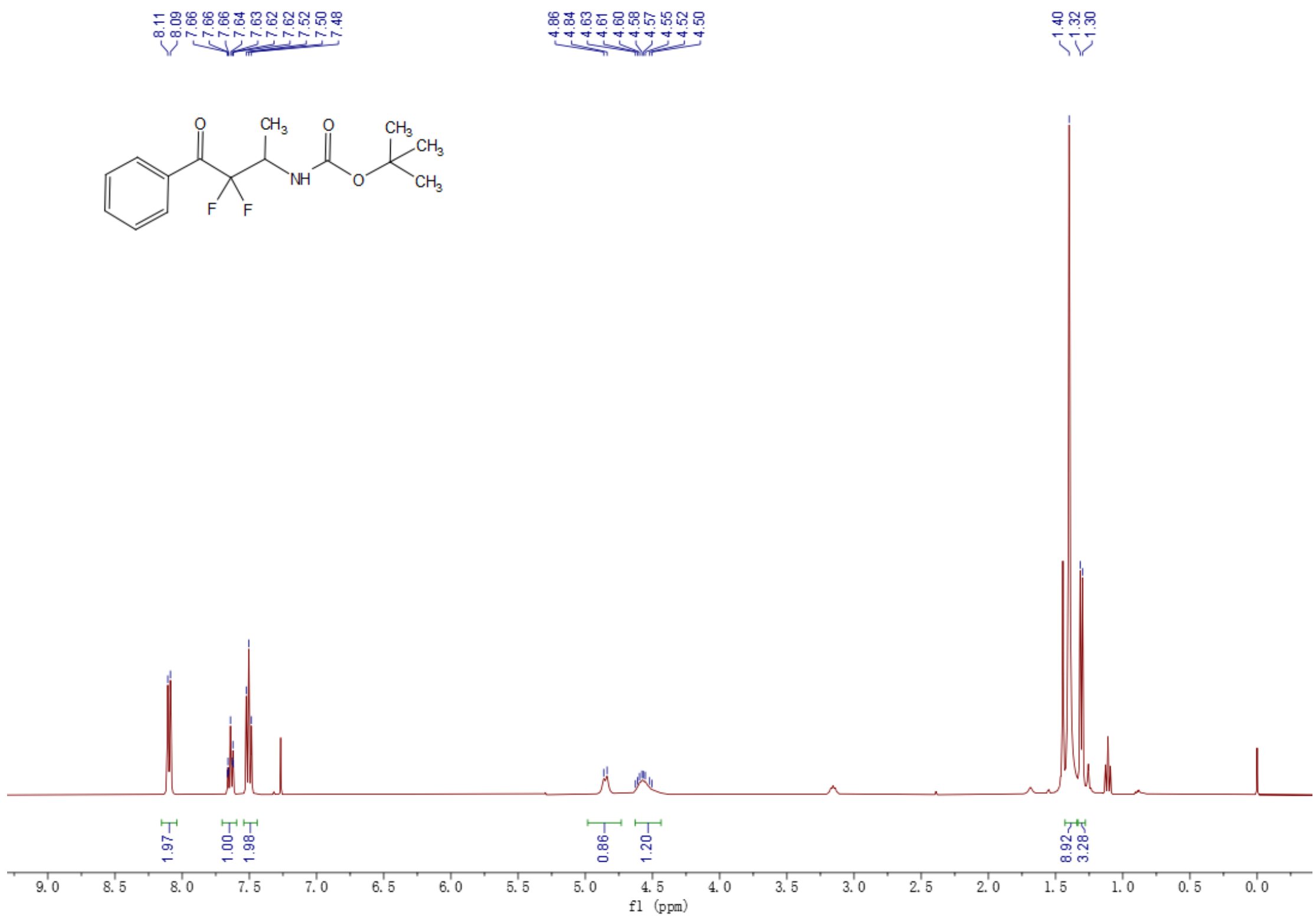

^{19}**F NMR** spectrum (376 MHz, $CDCl_3$) of compound **3m**

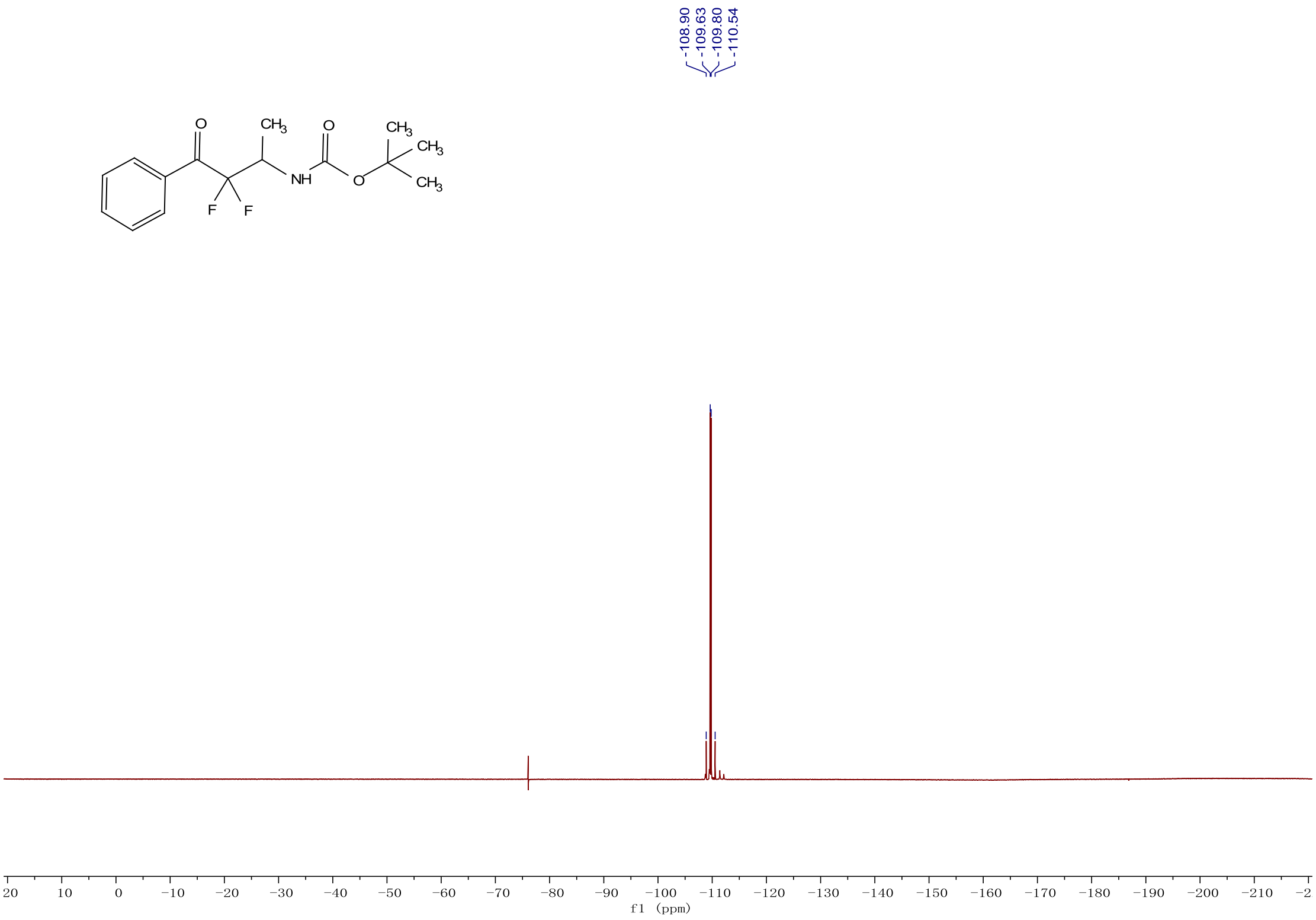

^{13}**C NMR** spectrum (101 MHz, $CDCl_3$) of compound **3m**

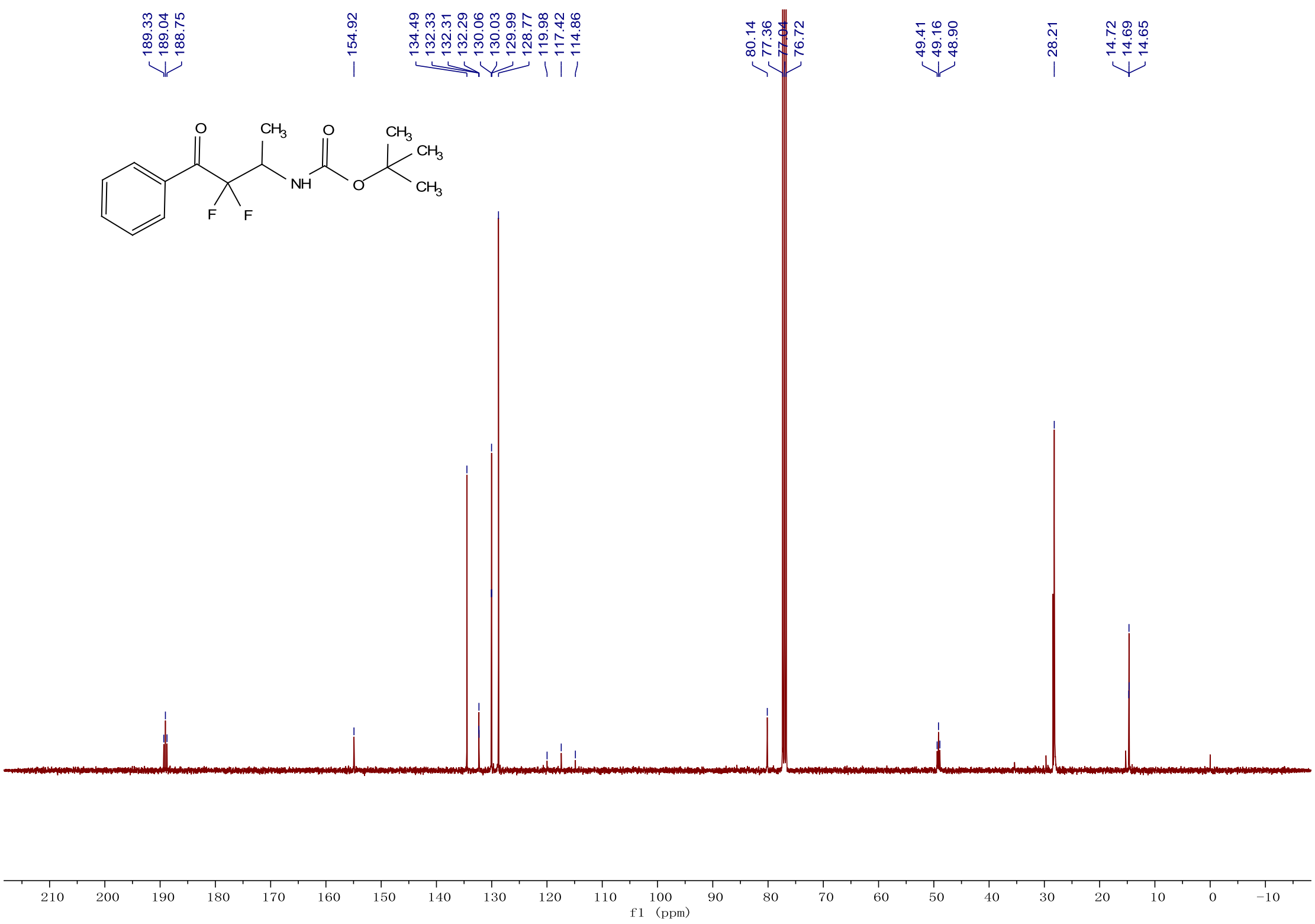

2,2-difluoro-3,3-dimethyl-1-phenylbutan-1-one (3n)

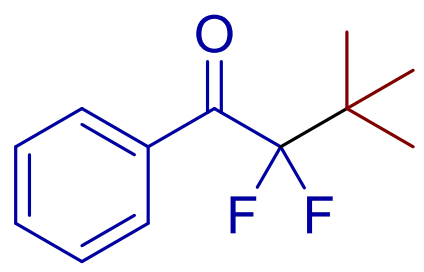

The product (28.9 mg, 68% yield) as colorless oil was purified with silica gel chromatography (petroleum ether/ ethyl acetate = 20/1).**^{1}H NMR** (400 MHz, Chloroform-*d*) δ 8.08 (d, *J* = 7.6 Hz, 2H), 7.62 – 7.58 (m, 1H), 7.47 (t, *J* = 8.4 Hz, 2H), 1.15 (s, 9H). **^{19}F NMR** (376 MHz, Chloroform-*d*) δ -108.76 (s, 2F). **^{13}C NMR** (101 MHz, Chloroform-*d*) δ 190.8 (t, *J* = 31.3 Hz), 134.5 (t, *J* = 3.0 Hz), 133.8, 130.2 (t, *J* = 5.0 Hz), 128.5, 121.9 (t, *J* = 259.6 Hz), 37.8 (t, *J* = 21.2 Hz), 24.1 (t, *J* = 4.0 Hz). **HRMS** (EI-TOF) m/z: $[M+Na]^+$ Calcd. for $C_{12}H_{14}F_2NaO$ 235.0905; Found 235.0909.

^{1}H NMR spectrum (400 MHz, $CDCl_3$) of compound **3n**

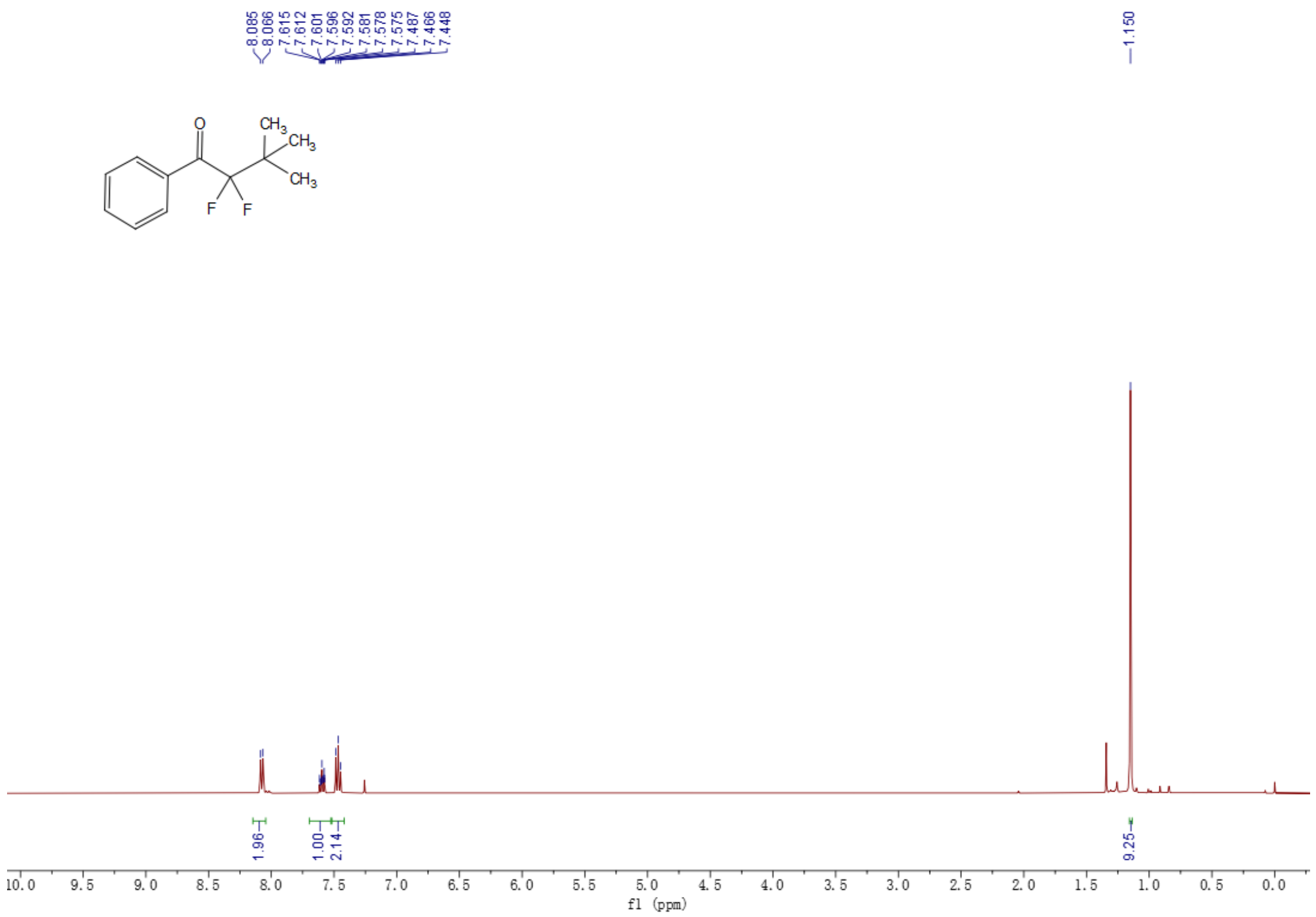

^{19}F NMR spectrum (376 MHz, $CDCl_3$) of compound **3n**

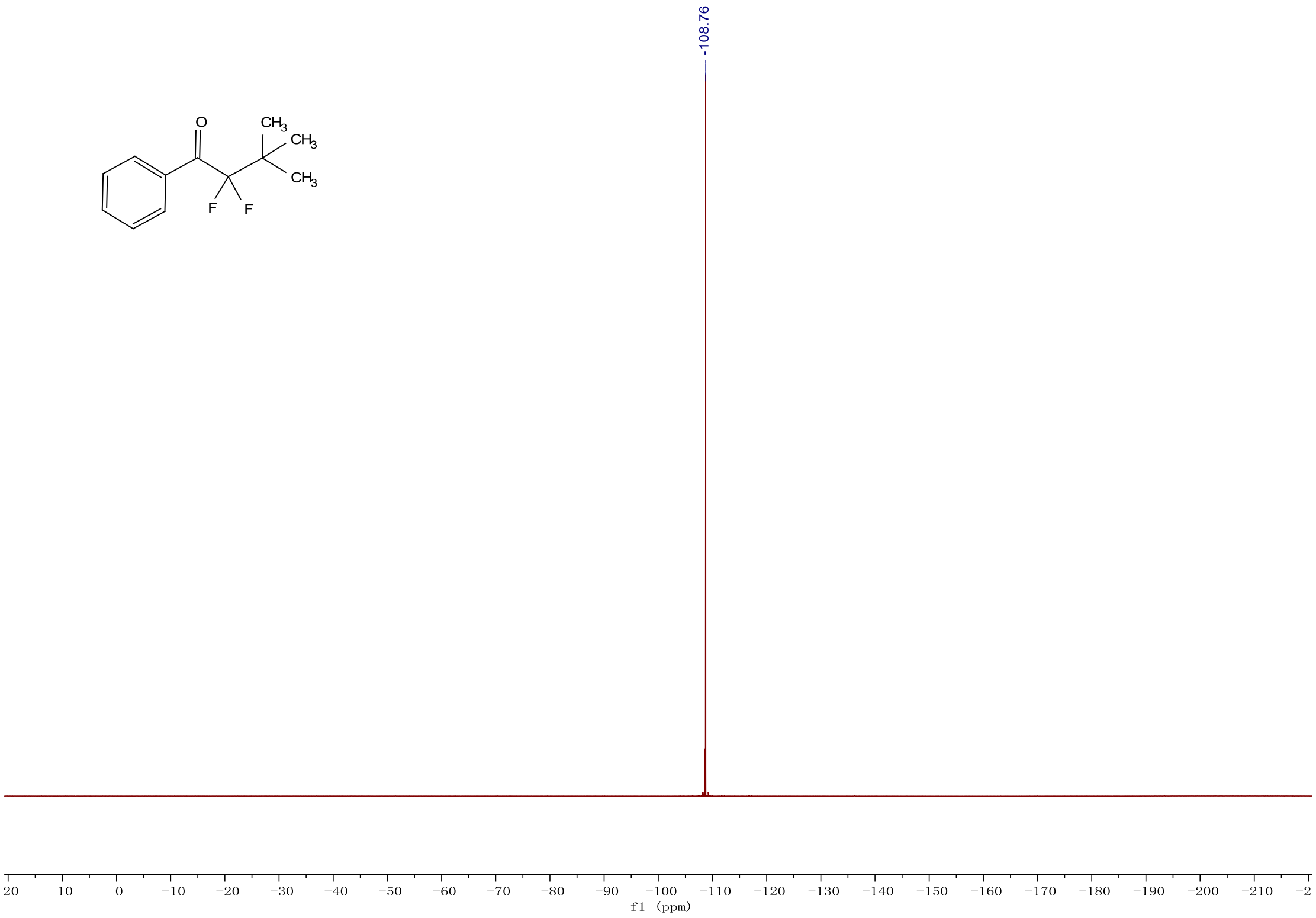

^{13}C NMR spectrum (101 MHz, $CDCl_3$) of compound **3n**

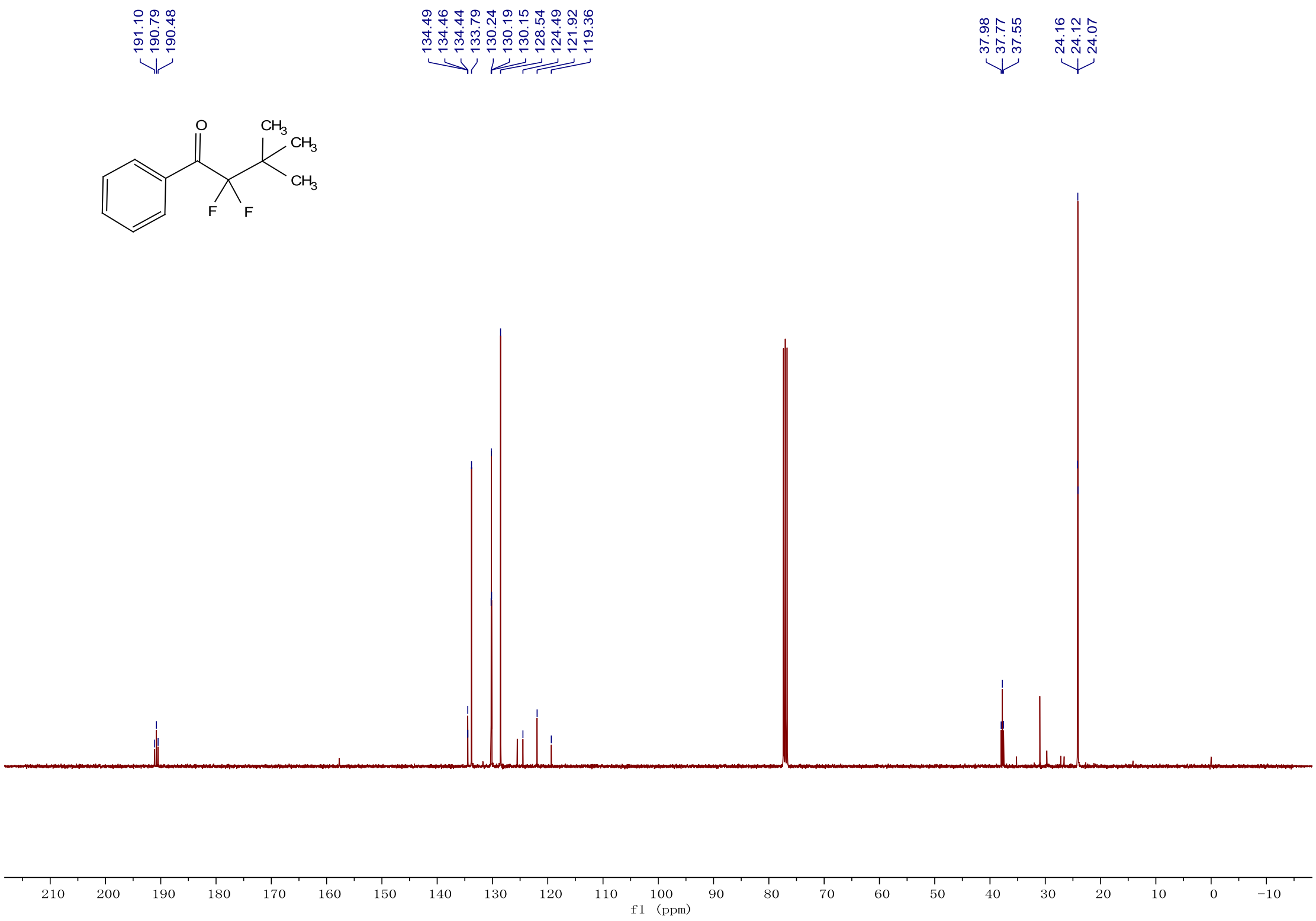

2,2-difluoro-2-(1-methylcyclohexyl)-1-phenylethan-1-one (3o)

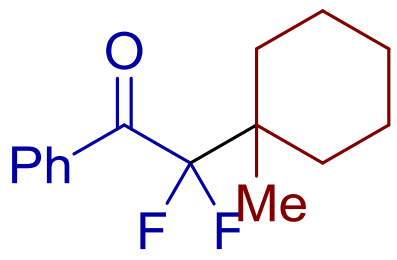

The product (38.8 mg, 77% yield) as colorless oil was purified with silica gel chromatography (petroleum ether/ ethyl acetate = 20/1). **^{1}H NMR** (400 MHz, Chloroform-*d*) δ 8.07 (d, *J* = 8.0 Hz, 2H), 7.59 (t, *J* =6.4 Hz, 1H), 7.46 (t, *J* =8.0 Hz, 2H), 2.88-2.71 (m, 1H), 1.66-1.54 (m, 8H),1.46-1.37 (m, 2H), 1.15 (s, 3H).**^{19}F NMR** (376 MHz, Chloroform-*d*) δ -110.7 (s, 2F). **^{13}C NMR** (101 MHz, Chloroform-*d*) δ 190.0 (t, *J* = 31.3 Hz), 134.1, 132.6 (t, *J* = 2.0 Hz), 130.1 (t, *J* = 4.0 Hz), 120.9 (t, *J* = 254.5 Hz), 42.7 (t, *J* = 22.2 Hz), 26.1, 26.0 (d, *J* = 4.0 Hz). **HRMS** (EI-TOF) m/z: $[M+Na]^+$ Calcd. for $C_{15}H_{18}F_2NaO$ 275.1218; Found 275.1222.

^{1}H NMR spectrum (400 MHz, $CDCl_3$) of compound **3o**

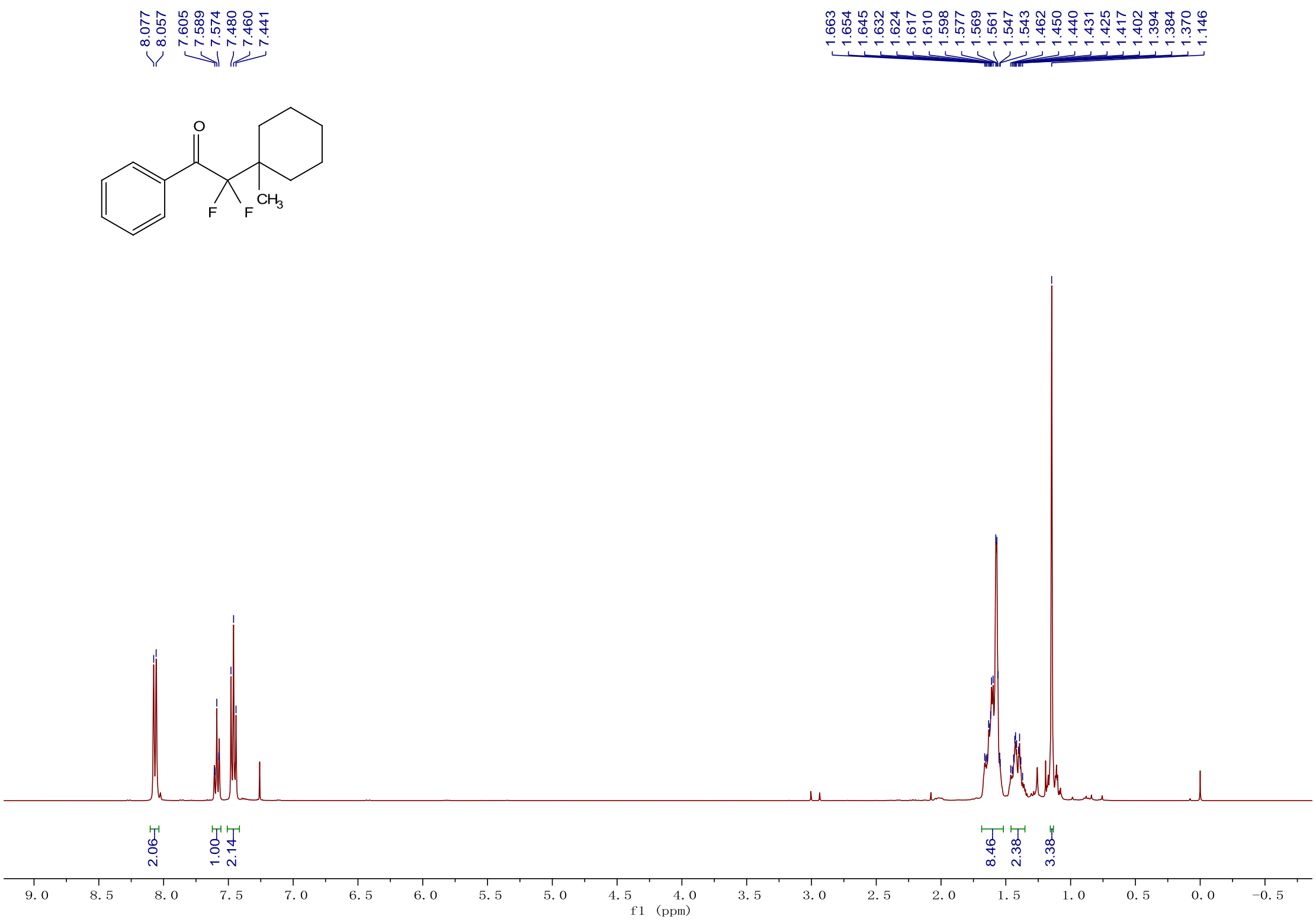

[19]**F NMR** spectrum (376 MHz, $CDCl_3$) of compound **3o**

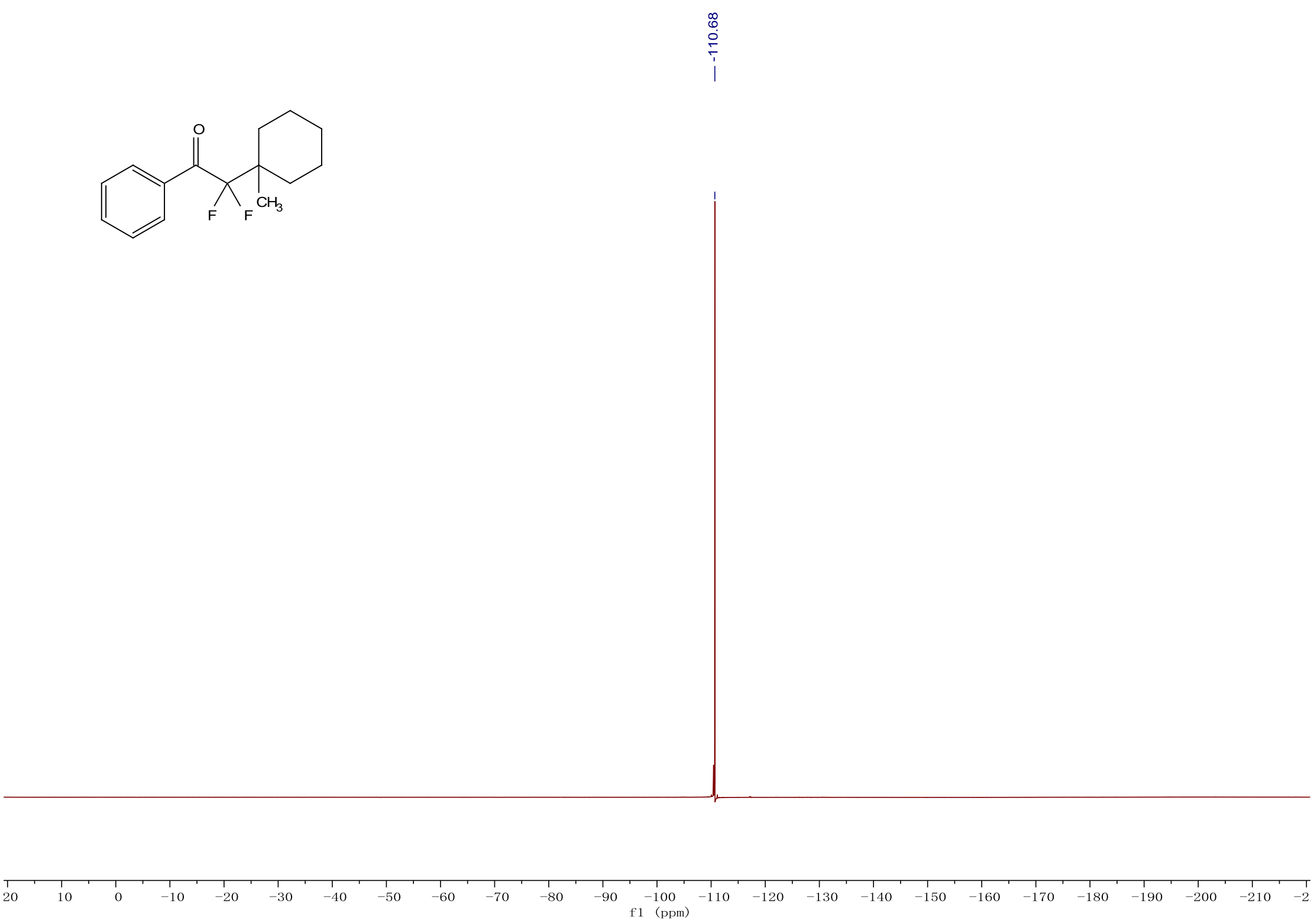

[13]**C NMR** spectrum (101 MHz, $CDCl_3$) of compound **3o**

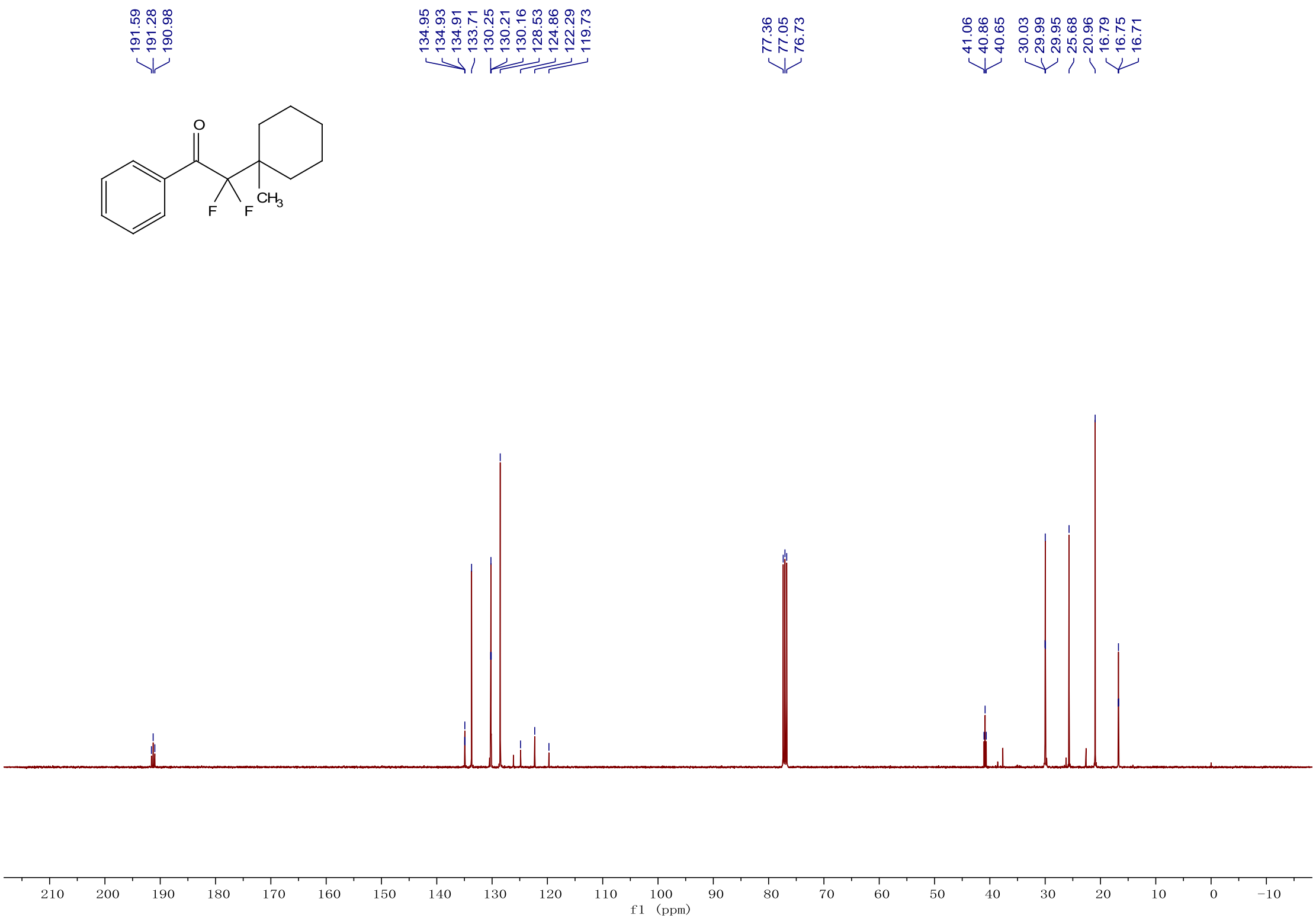

2-(adamantan-1-yl)-2,2-difluoro-1-phenylethan-1-one (3p)

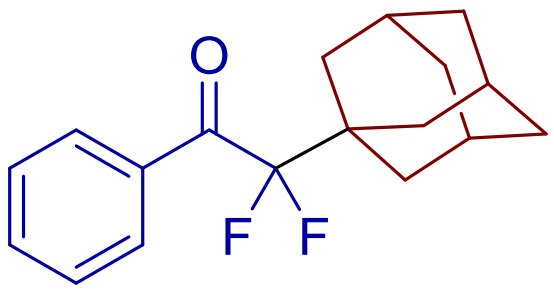

The product (45.9 mg, 79% yield) as white solid was purified with silica gel chromatography (petroleum ether/ ethyl acetate = 20/1). **^{1}H NMR** (400 MHz, Chloroform-*d*) δ 8.06 (d, *J* = 7.6 Hz, 2H), 7.61-7.57 (m, 1H), 7.48-7.44 (m, 2H), 2.01 (s, 3H), 1.80 (d, *J* = 2.8 Hz, 6H), 1.72-1.62 (m, 6H). **^{19}F NMR** (376 MHz, Chloroform-*d*) δ -113.36 (s, 2F). **^{13}C NMR** (101 MHz, Chloroform-*d*) δ 190.3 (t, *J* = 29.3 Hz), 134.9 (t, *J* = 2.0 Hz), 133.8, 130.3 (t, *J* = 4.0 Hz), 128.5, 121.5 (t, *J* = 257.5 Hz), 39.4 (t, J = 21.2 Hz), 36.4, 35.1 (t, *J* = 4.0 Hz), 27.6. **HRMS** (EI-TOF) m/z: $[M+Na]^+$ Calcd. for $C_{18}H_{20}F_2NaO$ 313.1374; Found 313.1384.

^{1}H NMR spectrum (400 MHz, $CDCl_3$) of compound **3p**

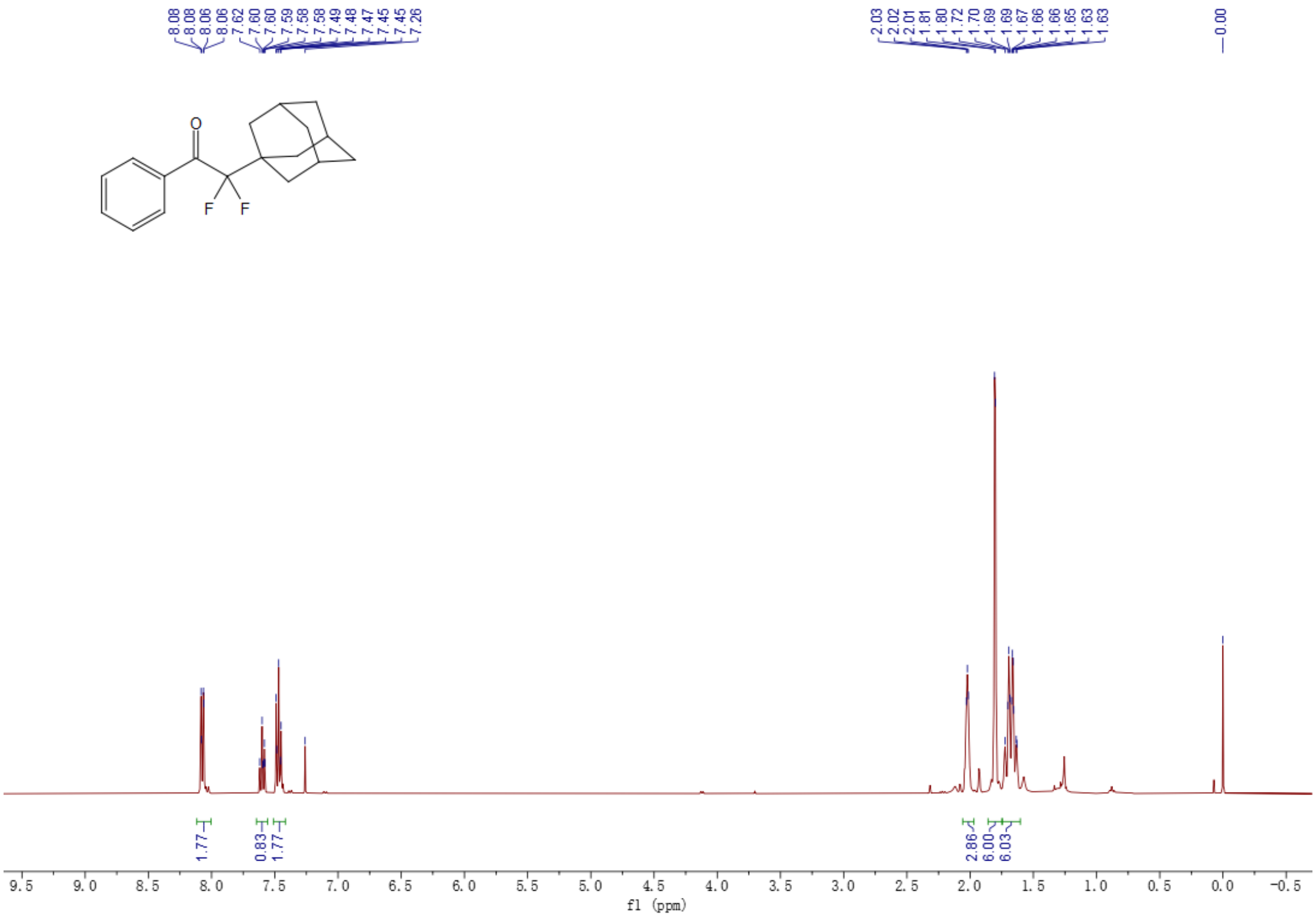

^{19}F NMR spectrum (376 MHz, $CDCl_3$) of compound **3p**

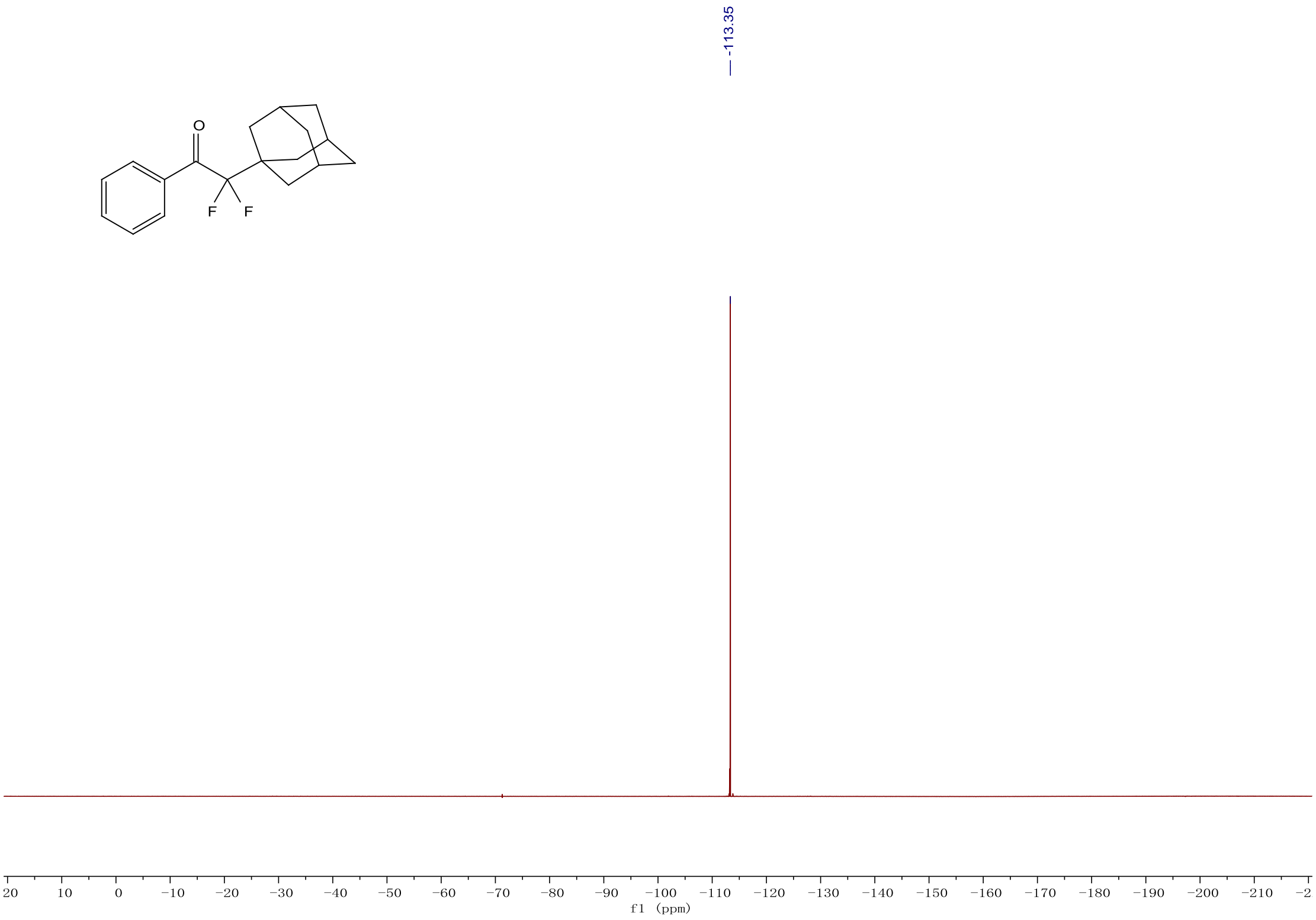

^{13}C NMR spectrum (101 MHz, $CDCl_3$) of compound **3p**

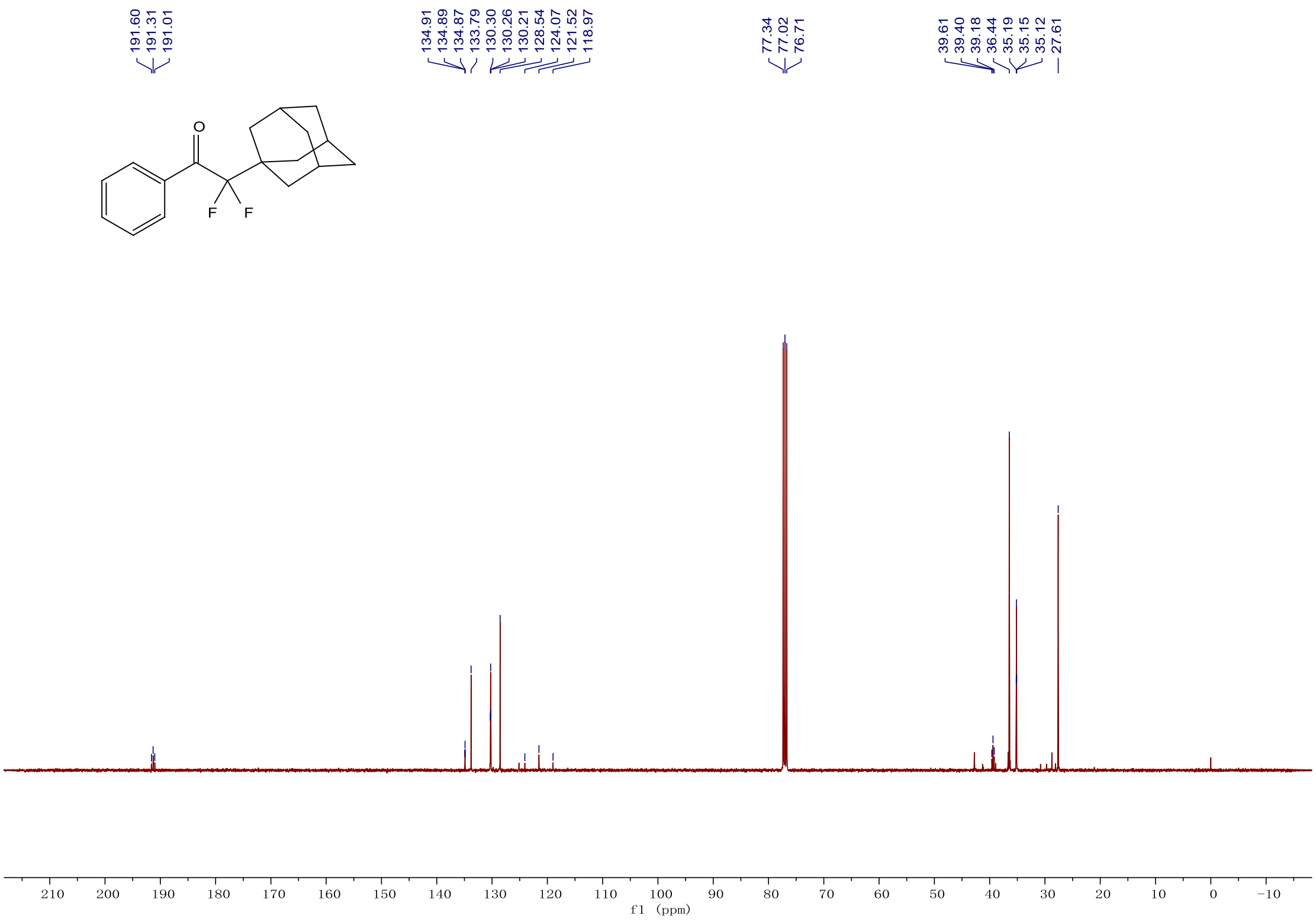

methyl 4-(1,1-difluoro-2-oxo-2-phenylethyl)bicyclo[2.2.2]octane-1-carboxylate (3q)

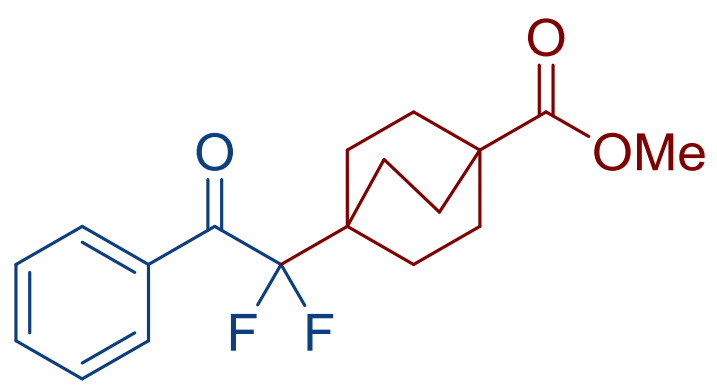

The product (47.1 mg, 73% yield) as white solid was purified with silica gel chromatography (petroleum ether/ ethyl acetate = 10/1). **^{1}H NMR** (400 MHz, Chloroform-*d*) δ 8.05 (d, *J* = 7.6 Hz, 2H), 7.63-7.57 (m, 1H), 7.47 (t, *J* = 8.0 Hz, 2H), 3.64 (s, 3H), 1.82-1.75 (m, 12H). **^{19}F NMR** (376 MHz, Chloroform-*d*) δ -108.32 (s, 2F). **^{13}C NMR** (101 MHz, Chloroform-*d*) δ 190.6 (t, *J* = 28.3), 177.7, 134.3 (t, *J* = 2.0 Hz), 134.0, 130.2 (t, *J* = 4.0 Hz), 128.6, 121.4 (t, *J* = 256.5 Hz), 51.8, 38.6, 38.2 (t, *J* = 21.2 Hz), 27.3, 24.1 (t, *J* = 4.0 Hz). **HRMS** (EI-TOF) m/z: $[M+Na]^+$ Calcd. for $C_{18}H_{20}F_2NaO_3$ 345.1273; Found 345.1280.

^{1}H NMR spectrum (400 MHz, $CDCl_3$) of compound **3q**

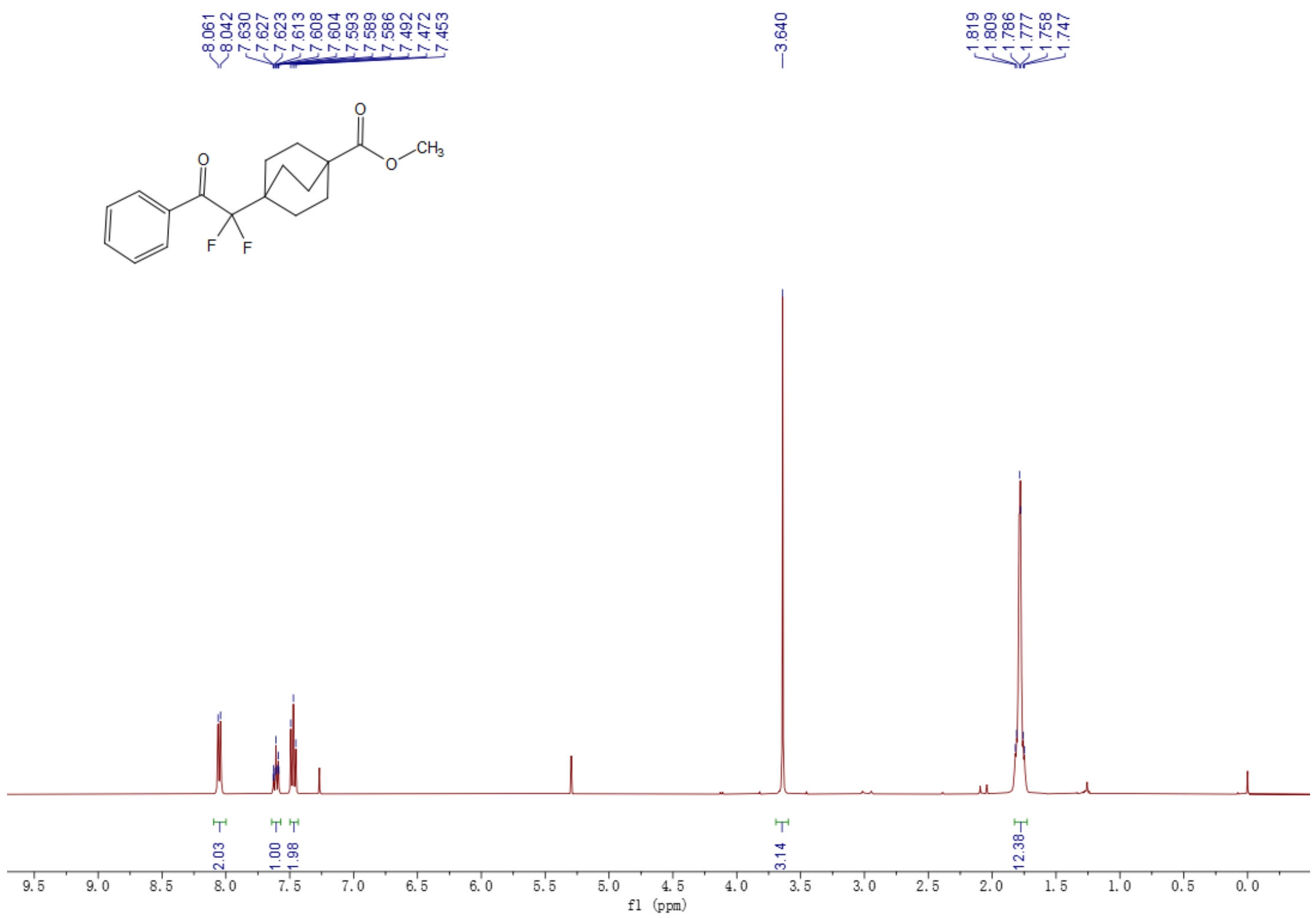

^{19}F NMR spectrum (376 MHz, $CDCl_3$) of compound **3q**

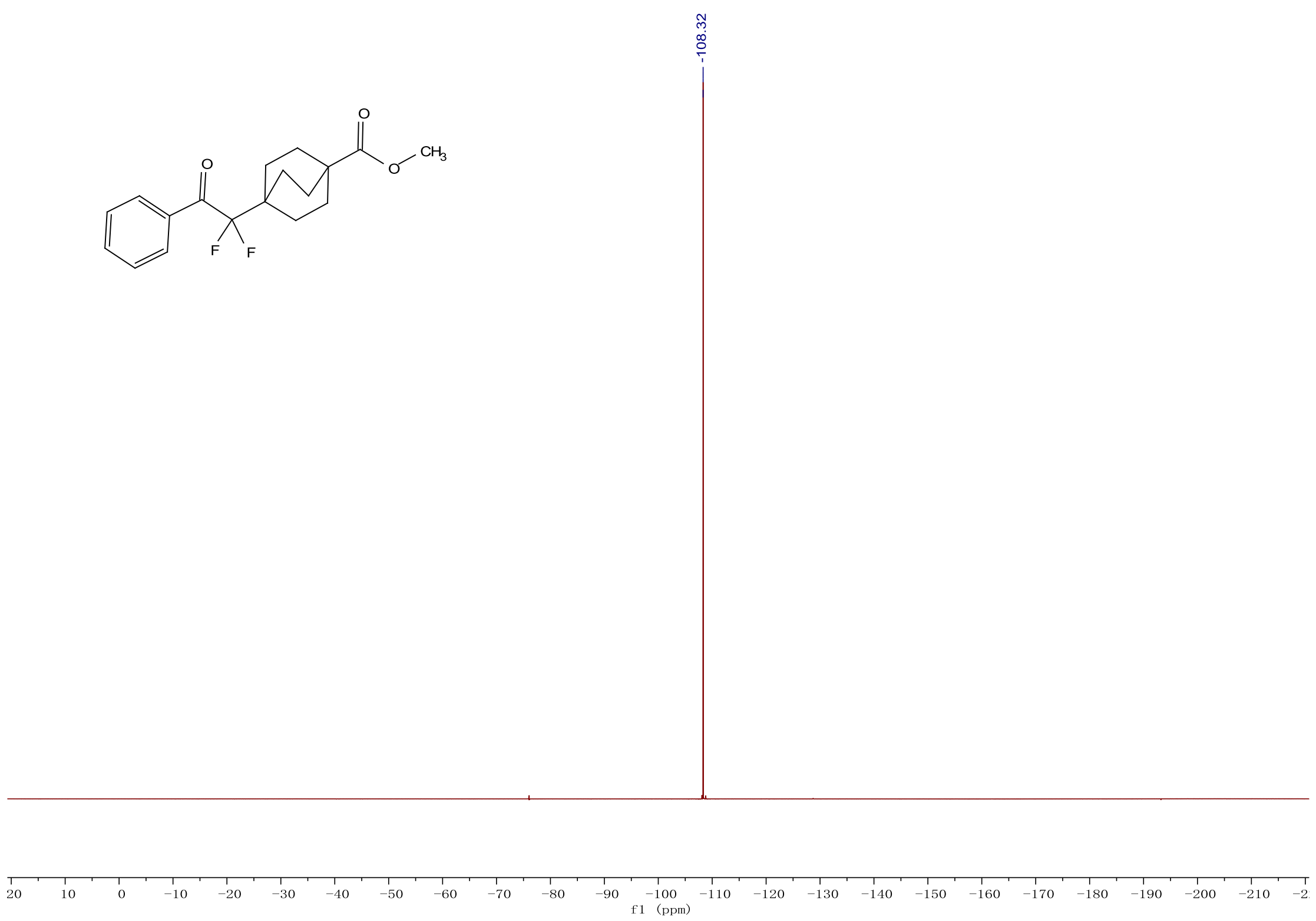

^{13}C NMR spectrum (101 MHz, $CDCl_3$) of compound **3q**

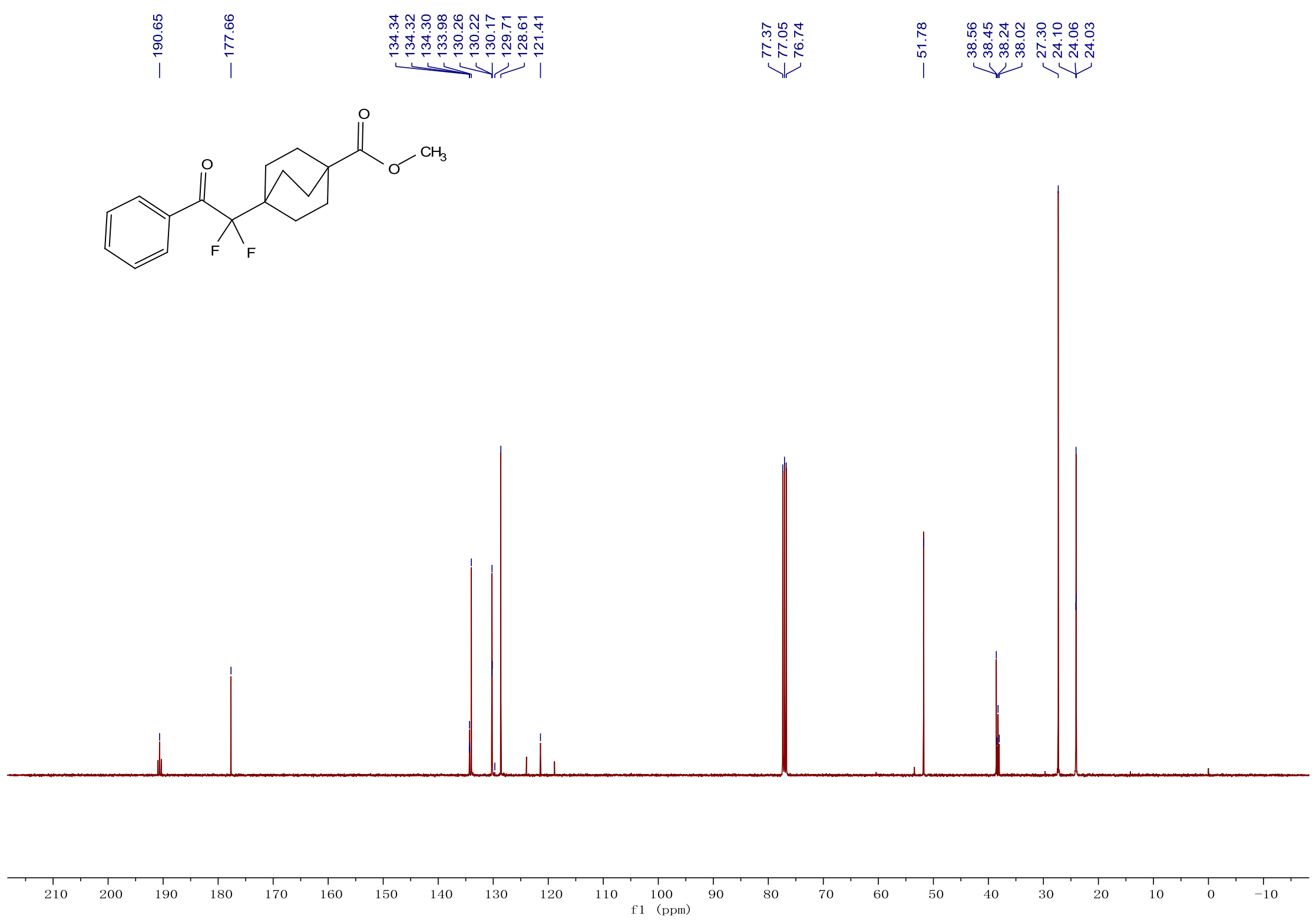

3-(4-chlorophenoxy)-2,2-difluoro-3-methyl-1-phenylbutan-1-one (3r)

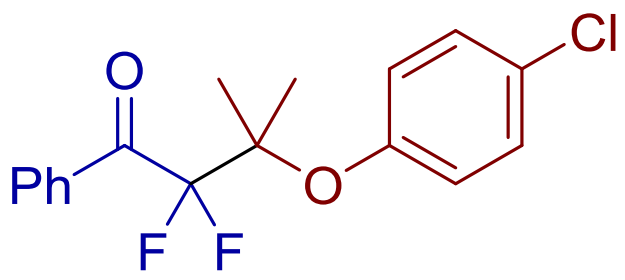

The product (48.7 mg, 75% yield) as colorless oil was purified with silica gel chromatography (petroleum ether/ ethyl acetate = 20/1). **^{1}H NMR** (400 MHz, Chloroform-*d*) δ 8.16 (d, *J* = 7.6 Hz, 2H), 7.60-7.56 (m, 1H), 7.47-7.43 (m, 2H), 7.20-7.18 (m,2H), 6.82-6.78 (m, 2H), 1.48 (s, 6H).**^{19}F NMR** (376 MHz, Chloroform-*d*) δ -110.88 (s, 2F). **^{13}C NMR** (101 MHz, Chloroform-*d*) δ 191.5 (t, *J* = 29.3 Hz), 151.7,134.4 (t, *J* = 2.0 Hz), 133.8, 130.7 (t, *J* = 4.0 Hz), 129.9, 129.2, 128.3, 125.1, 118.0 (t, *J* = 260.6 Hz), 82.4 (t, *J* = 26.3 Hz), 20.9. **HRMS** (EI-TOF) m/z: [M+Na]$^{+}$ Calcd. for $C_{17}H_{15}ClF_{2}NaO_{2}$ 347.0621; Found 347.0628.

^{1}H NMR spectrum (400 MHz, $CDCl_3$) of compound **3r**

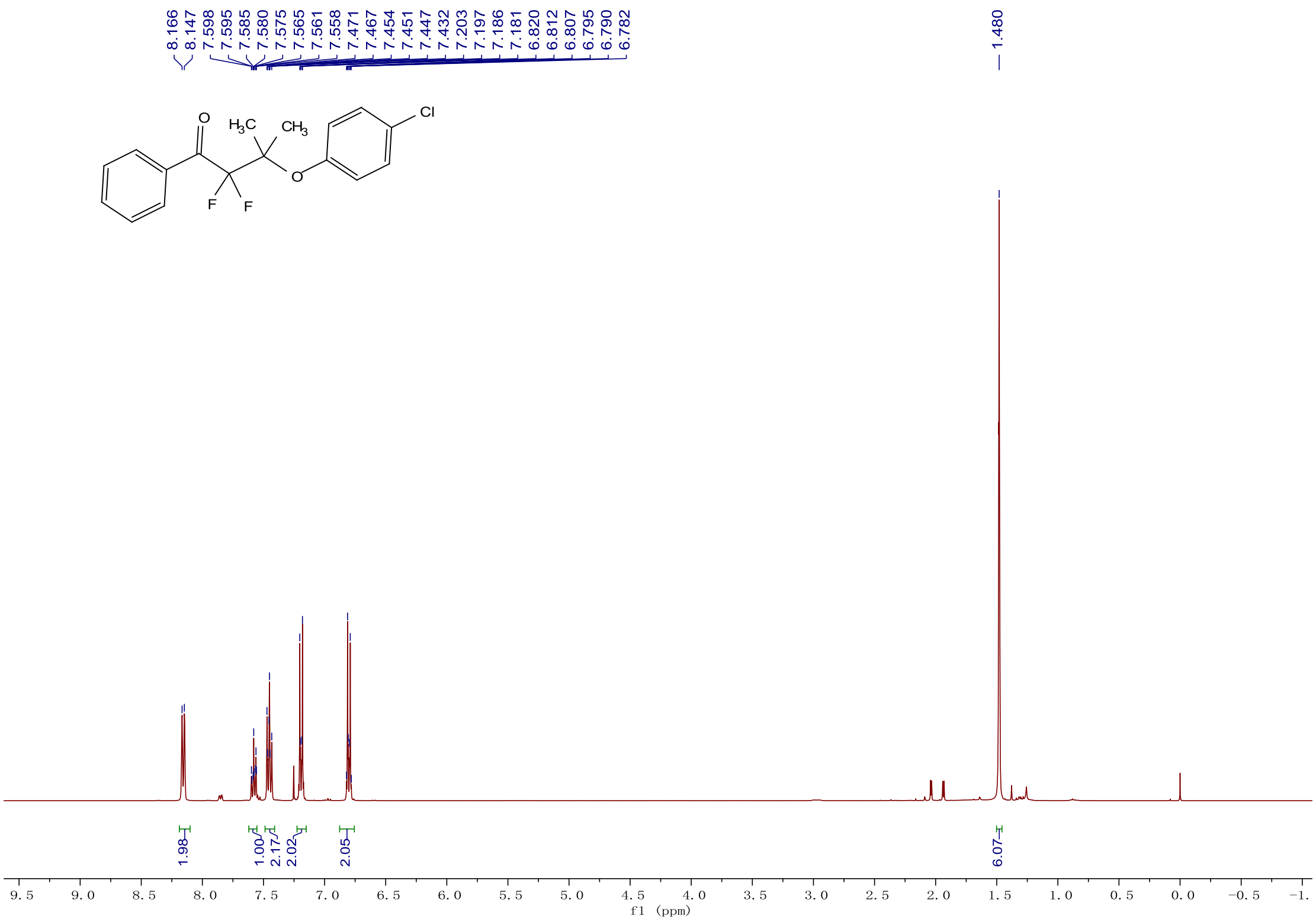

^{19}F NMR spectrum (376 MHz, $CDCl_3$) of compound **3r**

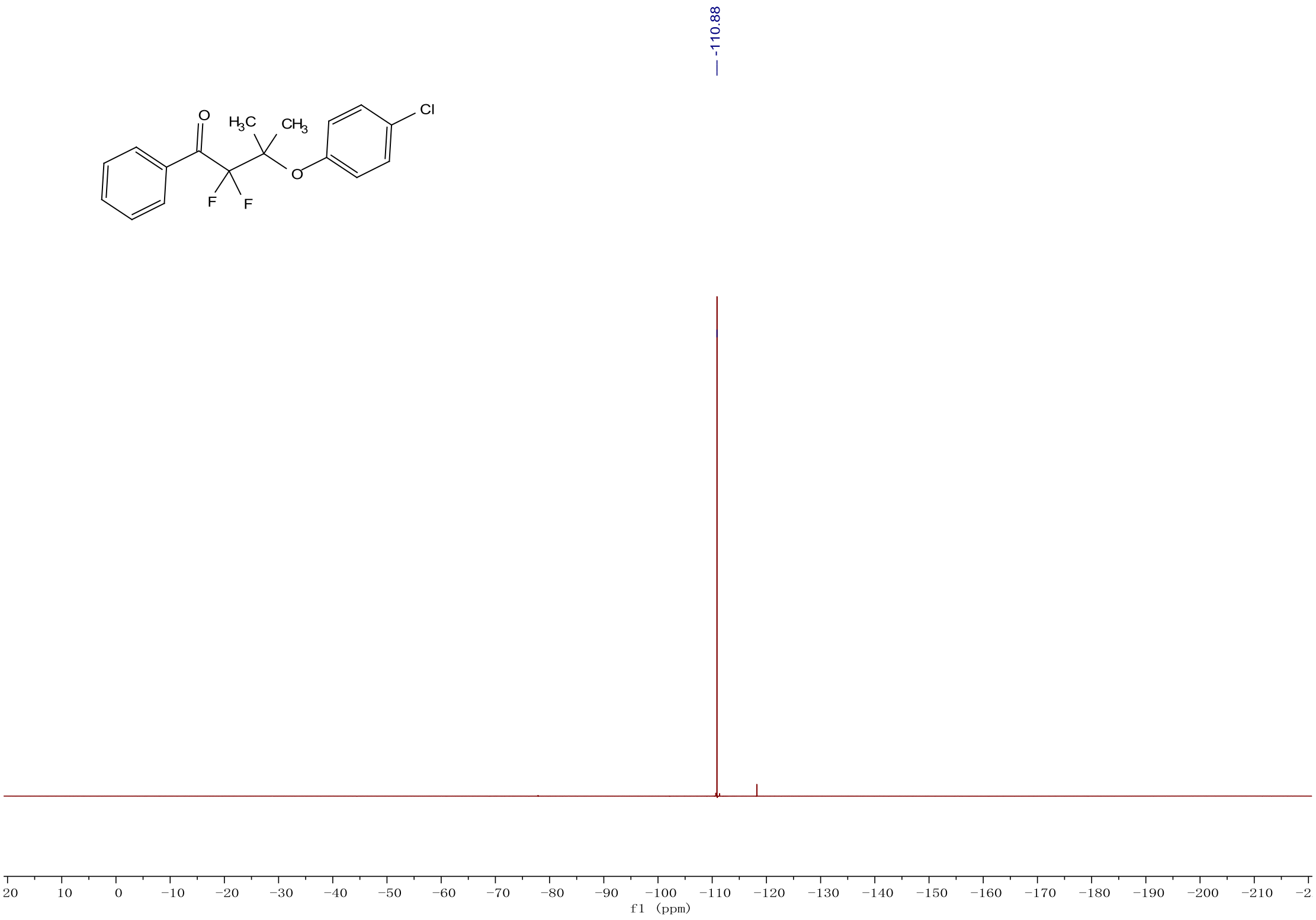

^{13}C NMR spectrum (101 MHz, $CDCl_3$) of compound **3r**

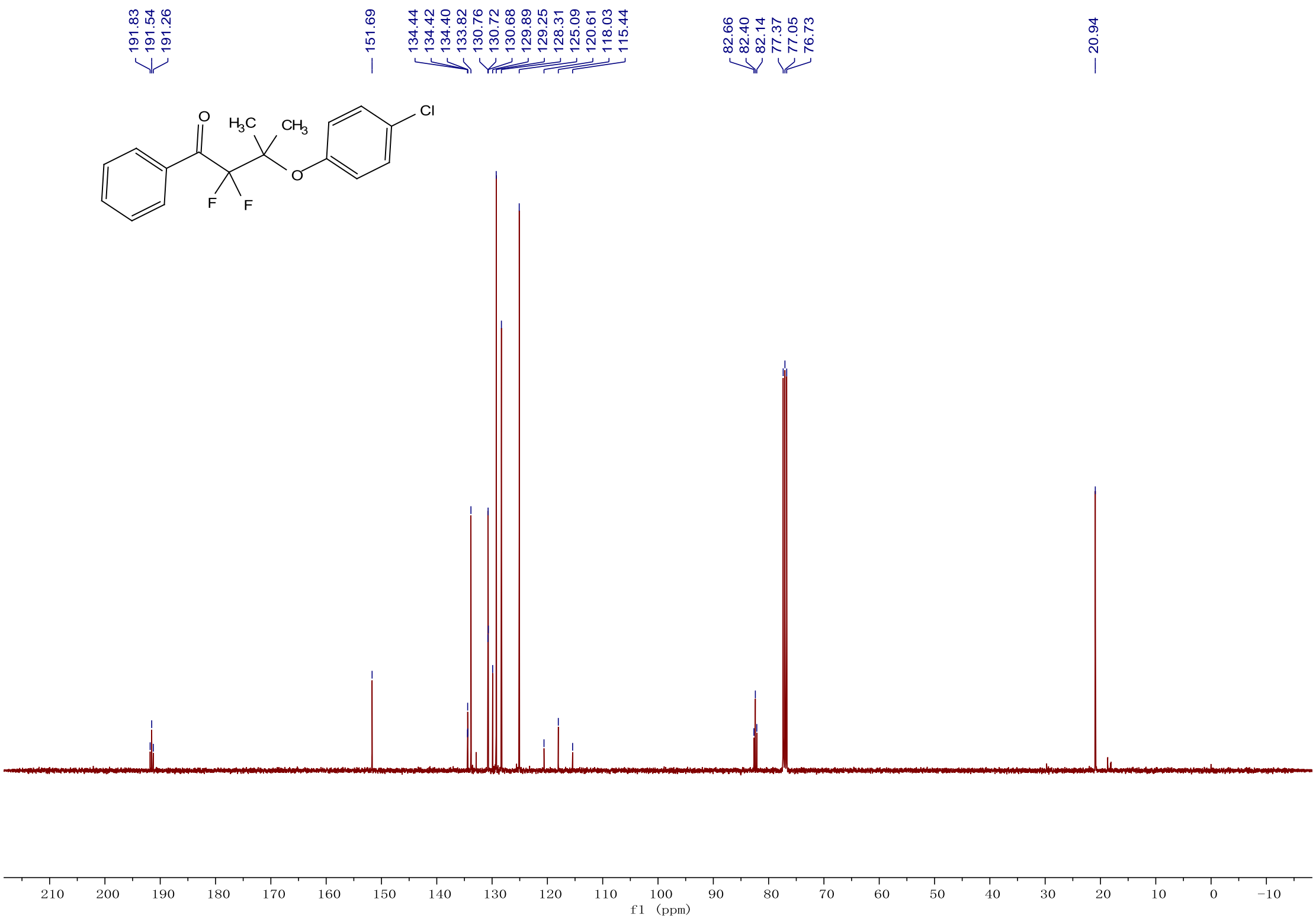

6-(2,5-dimethylphenoxy)-2,2-difluoro-3,3-dimethyl-1-phenylhexan-1-one (3s)

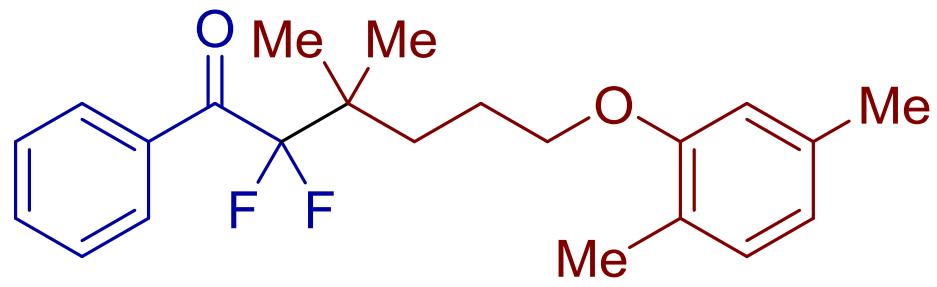

The product (62.7 mg, 87% yield) as colorless oil was purified with silica gel chromatography (petroleum ether/ ethyl acetate = 20/1). **^{1}H NMR** (400 MHz, Chloroform-*d*) δ 8.07 (d, *J* = 7.6 Hz, 2H), 7.60-7.56 (m, 1H), 7.45 (t, *J* = 8.0 Hz, 2H), 6.99 (d, *J* = 7.6 Hz, 1H), 6.65 (d, *J* = 7.6 Hz, 1H), 6.60 (s, 1H), 3.90 (t, *J* = 6.0 Hz, 2H) 2.30 (s, 3H), 2.15 (s, 3H), 1.88-1.79 (m, 2H), 1.74-1.70 (m, 2H), 1.16 (s, 6H).**^{19}F NMR** (376 MHz, Chloroform-*d*) δ -107.06 (s, 2F). **^{13}C NMR** (101 MHz, Chloroform-*d*) δ 190.9 (t, *J* = 31.3 Hz), 157.0, 136.5, 134.6 (t, *J* = 3.0 Hz), 133.8, 130.3, 130.2 (t, *J* = 25.2 Hz), 128.6, 123.6, 122.1 (t, *J* = 260.6 Hz), 120.8, 112.0, 68.1, 40.6 (t, *J* = 21.2 Hz), 32.7 (t, *J* = 4.0 Hz), 24.1 (t, *J* = 2.0 Hz), 21.4, 21.2 (t, *J* = 4.0 Hz), 15.8. **HRMS** (EI-TOF) m/z: $[M+Na]^+$ Calcd. for $C_{22}H_{26}F_2NaO_2$ 383.1793; Found 383.1806.

^{1}H NMR spectrum (400 MHz, $CDCl_3$) of compound **3s**

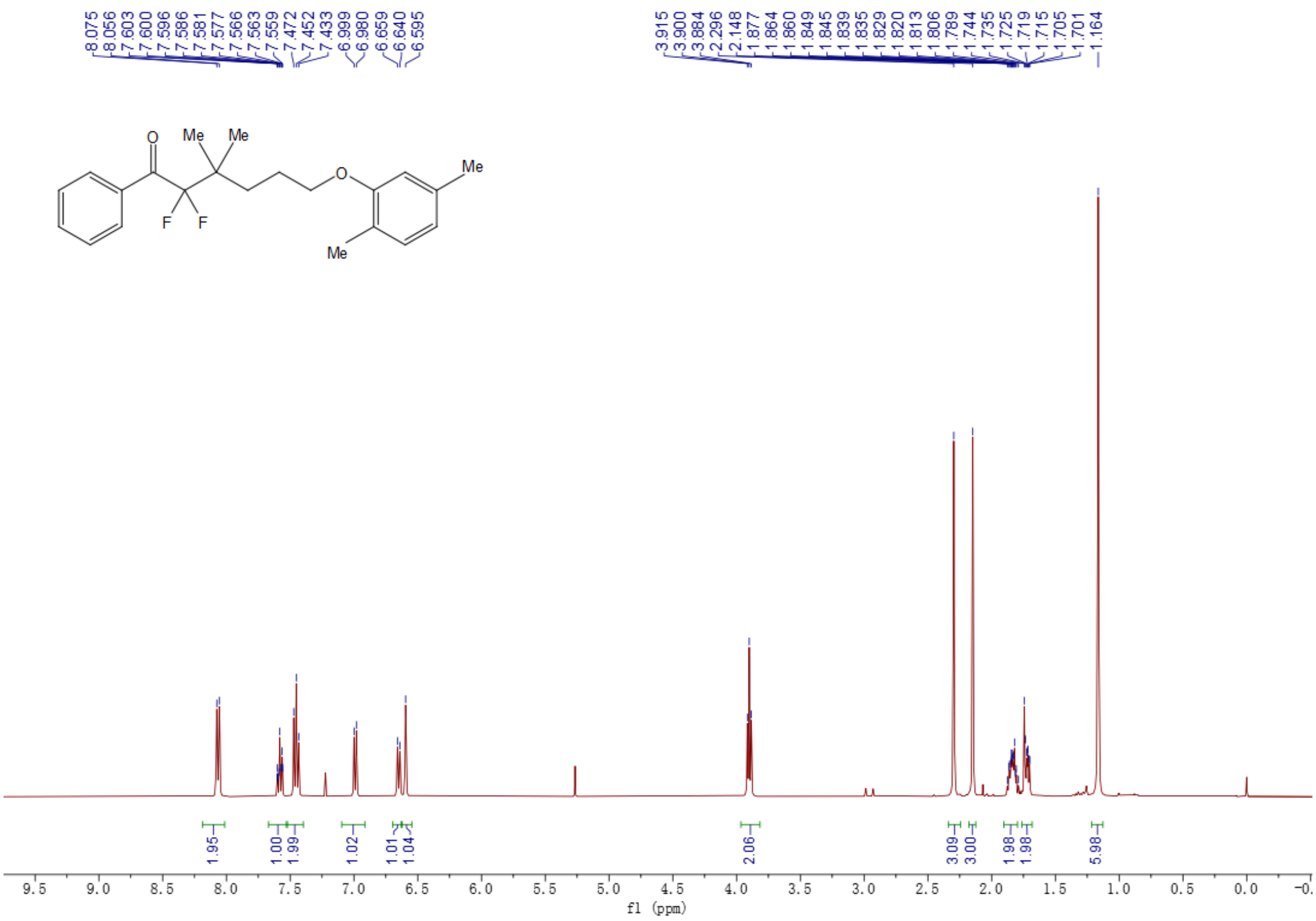

^{19}F NMR spectrum (376 MHz, $CDCl_3$) of compound **3s**

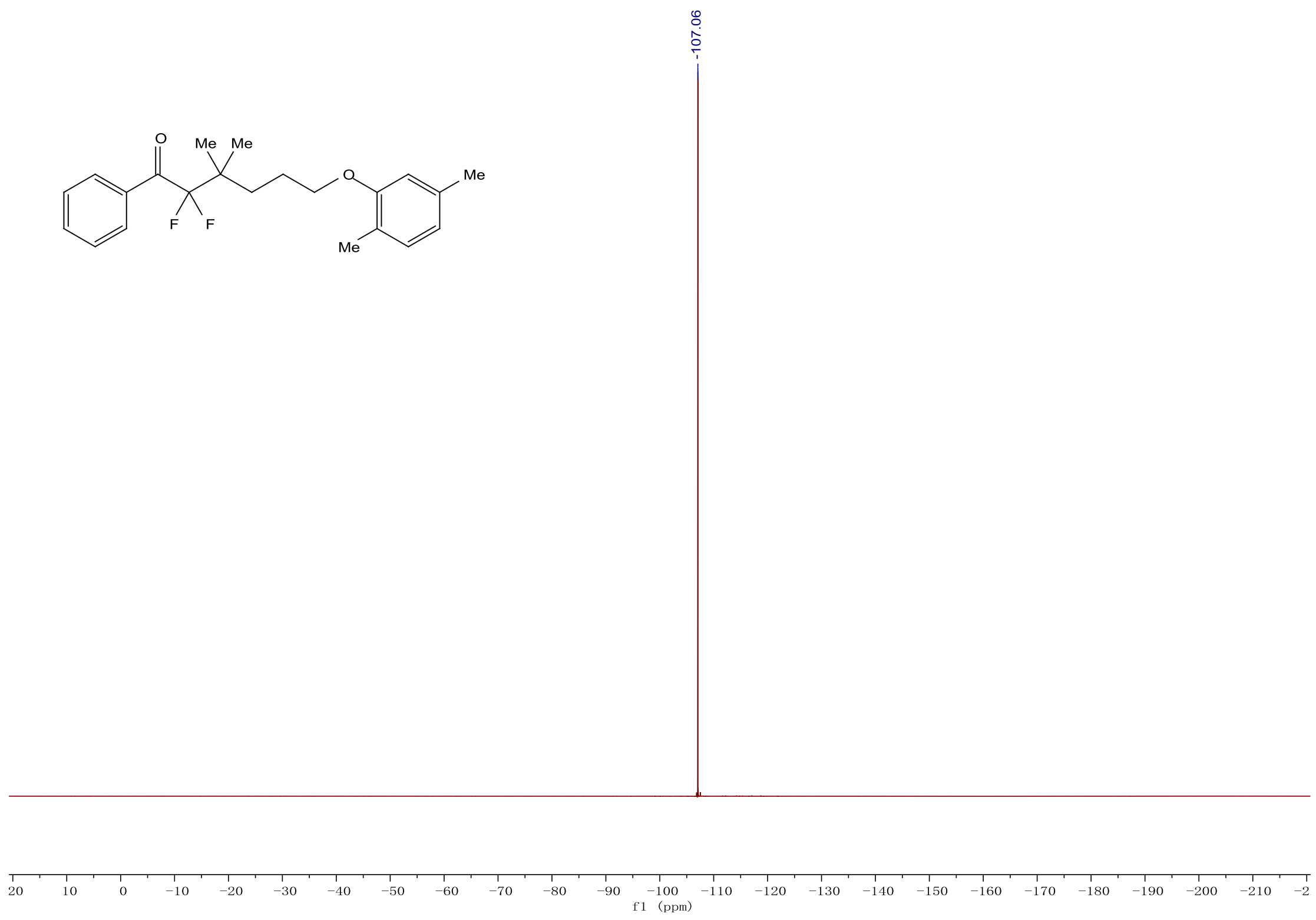

^{13}C NMR spectrum (101 MHz, $CDCl_3$) of compound **3s**

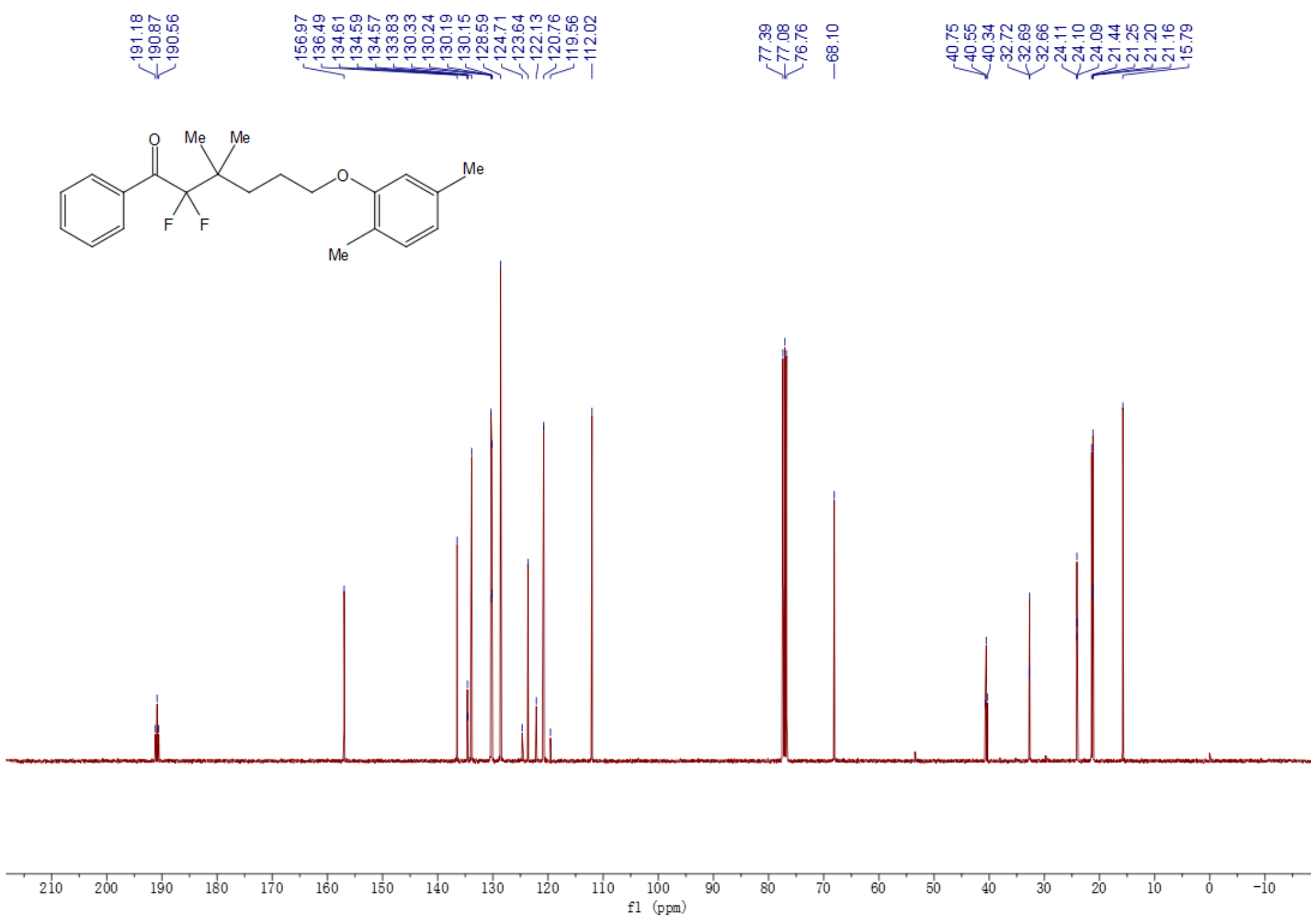

2-cyclohexyl-1-(3,4-dimethylphenyl)-2,2-difluoroethan-1-one (3t)

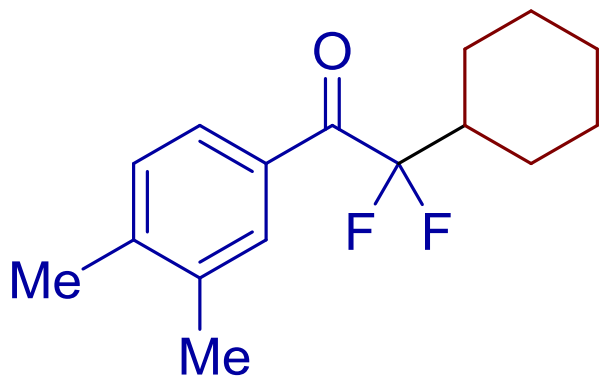

The product (40.0 mg, 75% yield) as colorless oil was purified with silica gel chromatography (petroleum ether/ ethyl acetate = 20/1). **^{1}H NMR** (400 MHz, Chloroform-*d*) δ 7.84-7.81 (m, 2H), 7.23 (d, *J* = 8.0 Hz, 1H), 2.33 (s, 3H), 2.32 (s, 3H), 2.26-2.18 (m, 1H), 1.83-1.78 (m, 4H), 1.70-1.67 (m, 1H),1.33-1.16 (m, 5H).**^{19}F NMR** (376 MHz, Chloroform-*d*) δ -108.53 (s, 2F). **^{13}C NMR** (101 MHz, Chloroform-*d*) δ 190.2 (t, *J* = 30.3 Hz), 144.0, 137.2, 131.0 (t, *J* = 3.0 Hz), 130.8 (t, *J* = 2.0 Hz), 129.9, 127.8 (t, *J* = 4.0 Hz), 120.4 (t, *J* = 255.5 Hz), 42.3 (t, *J* = 21.2 Hz), 25.9, 25.5, 24.9 (t, *J* = 4.0 Hz), 20.1, 19.8. **HRMS** (EI-TOF) m/z: $[M+Na]^+$ Calcd. for $C_{16}H_{20}F_2NaO$ 289.1374; Found 289.1383.

^{1}H NMR spectrum (400 MHz, $CDCl_3$) of compound **3t**

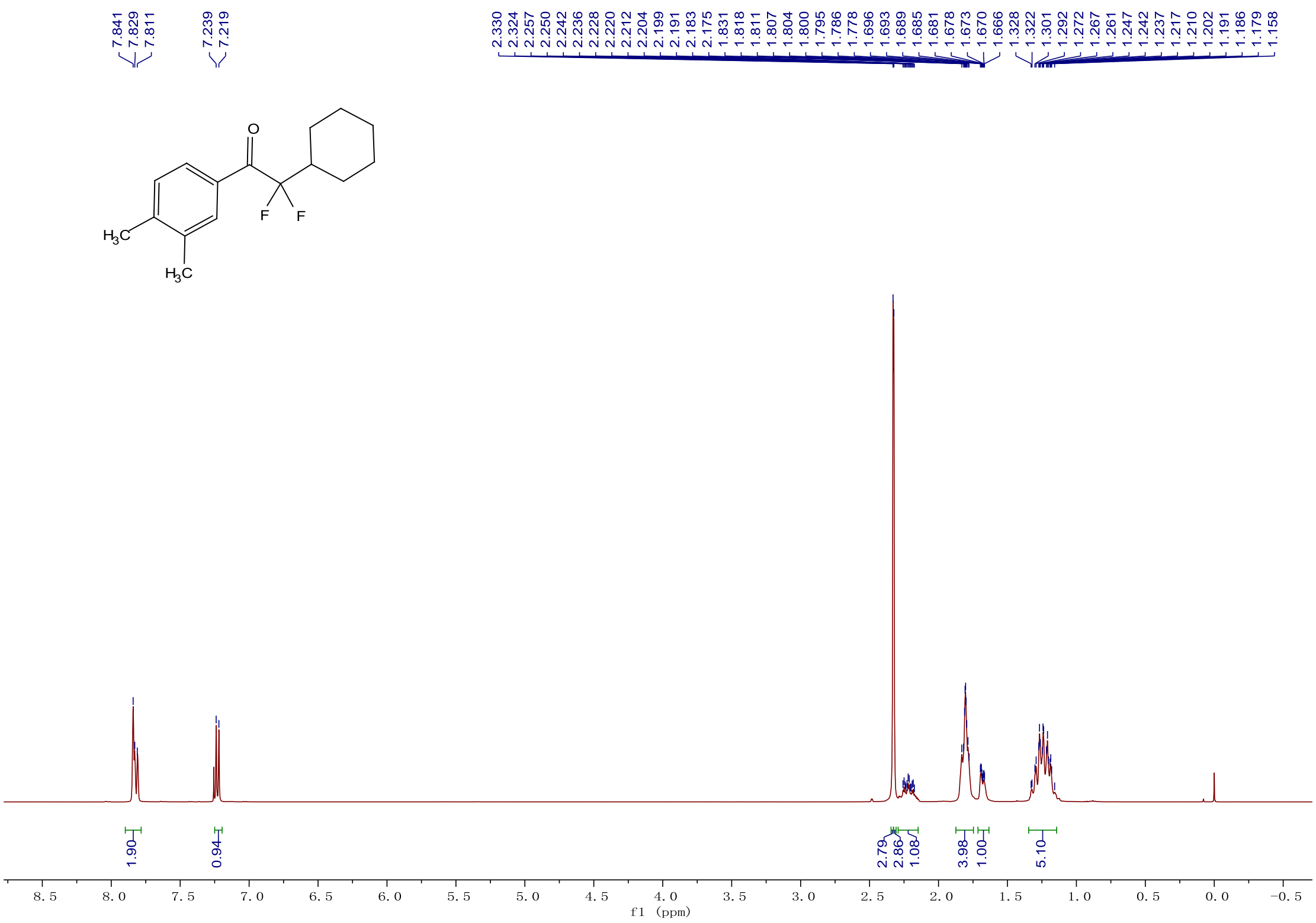

^{19}F NMR spectrum (376 MHz, $CDCl_3$) of compound **3t**

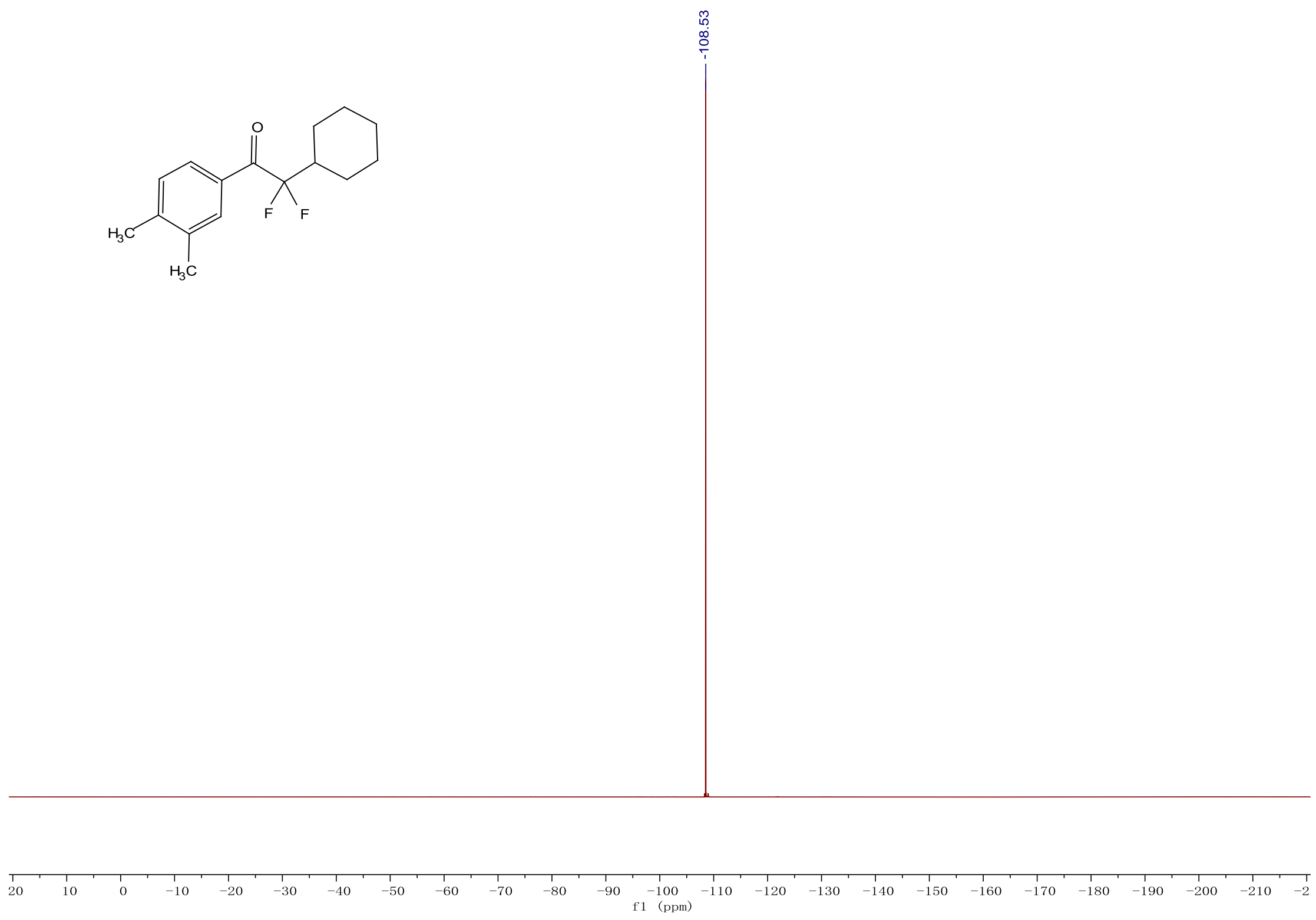

^{13}**C NMR** spectrum (101 MHz, $CDCl_3$) of compound **3t**

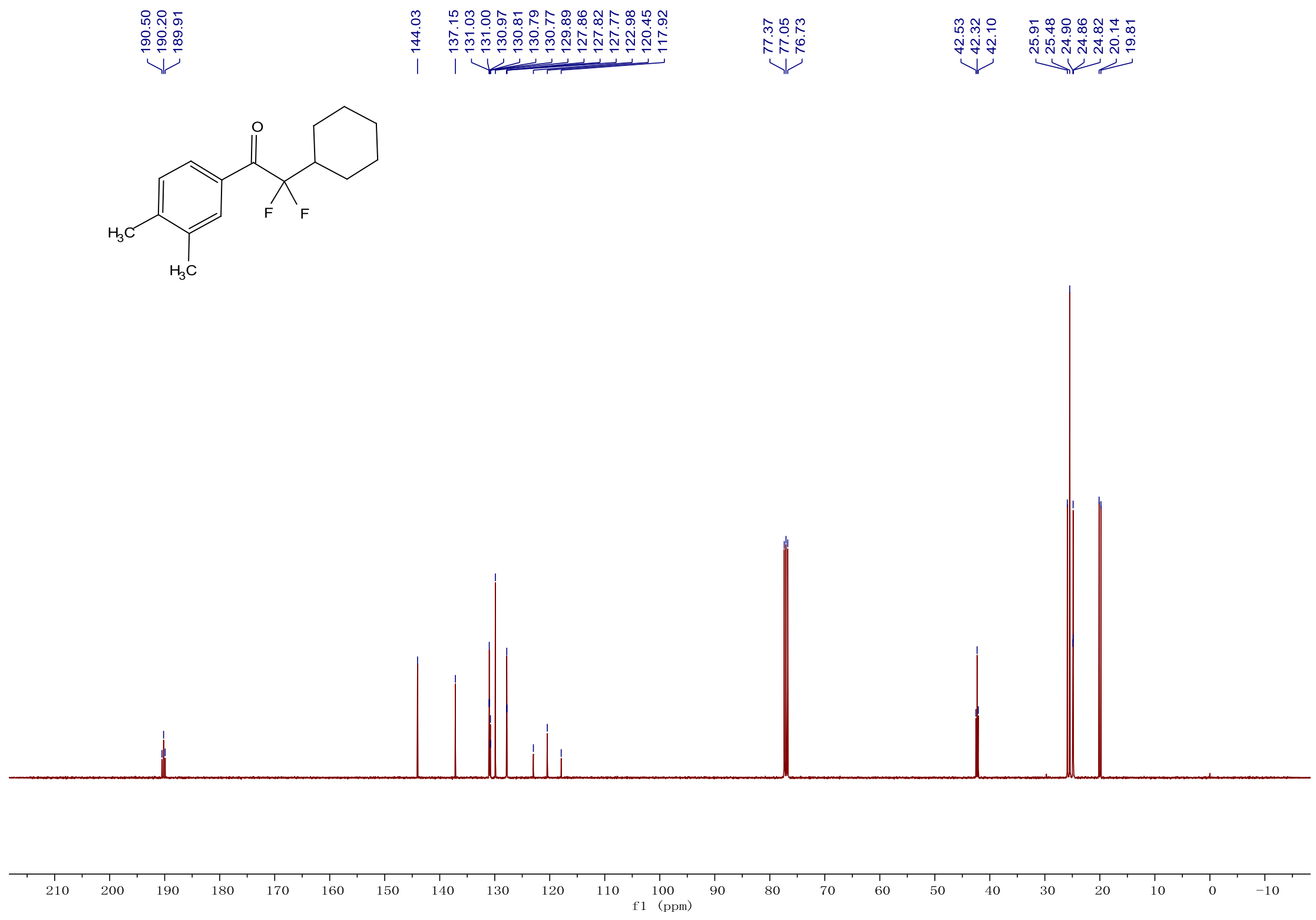

1-([1,1'-biphenyl]-4-yl)-2-cyclohexyl-2,2-difluoroethan-1-one (3u)

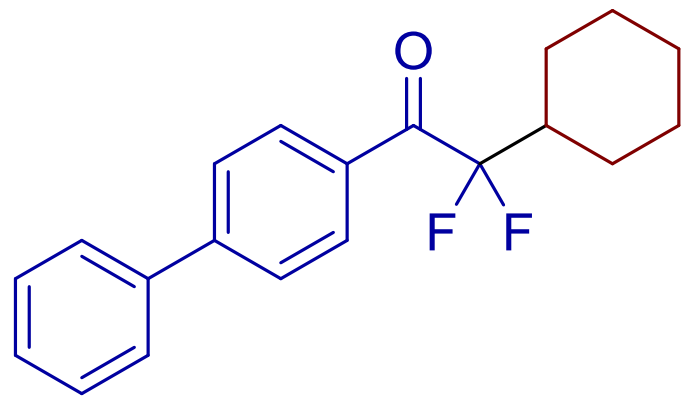

The product (45.3 mg, 72% yield) as white solid was purified with silica gel chromatography (petroleum ether/ ethyl acetate = 20/1). **^{1}H NMR** (400 MHz, Chloroform-*d*) δ 8.16 (d, *J* = 8.4 Hz, 2H), 7.70 (d, *J* = 8.4 Hz, 2H), 7.64-7.62 (m, 2H), 7.50-7.46 (m, 2H), 7.43-7.39 (m, 1H), 2.34-2.20 (m, 1H), 1.87-1.80 (m,4H), 1.71-1.68 (m, 1H), 1.36-1.16 (m, 5H). **^{19}F NMR** (376 MHz, Chloroform-*d*) δ -108.40 (s, 2F). **^{13}C NMR** (101 MHz, Chloroform-*d*) δ 190.0 (t, *J* = 30.3 Hz), 146.8, 139.6, 131.6 (t, *J* = 2.0 Hz), 130.7 (t, *J* = 3.0 Hz), 129.1, 128.5, 127.3, 127.29, 120.45 (t, *J* = 255.5 Hz), 42.2 (t, *J* = 22.2 Hz), 25.9, 25.5, 24.9 (t, *J* = 4.0 Hz). **HRMS** (EI-TOF) m/z: $[M+Na]^+$ Calcd. for $C_{20}H_{20}F_2NaO$ 337.1374; Found 337.1377.

^{1}H NMR spectrum (400 MHz, $CDCl_3$) of compound **3u**

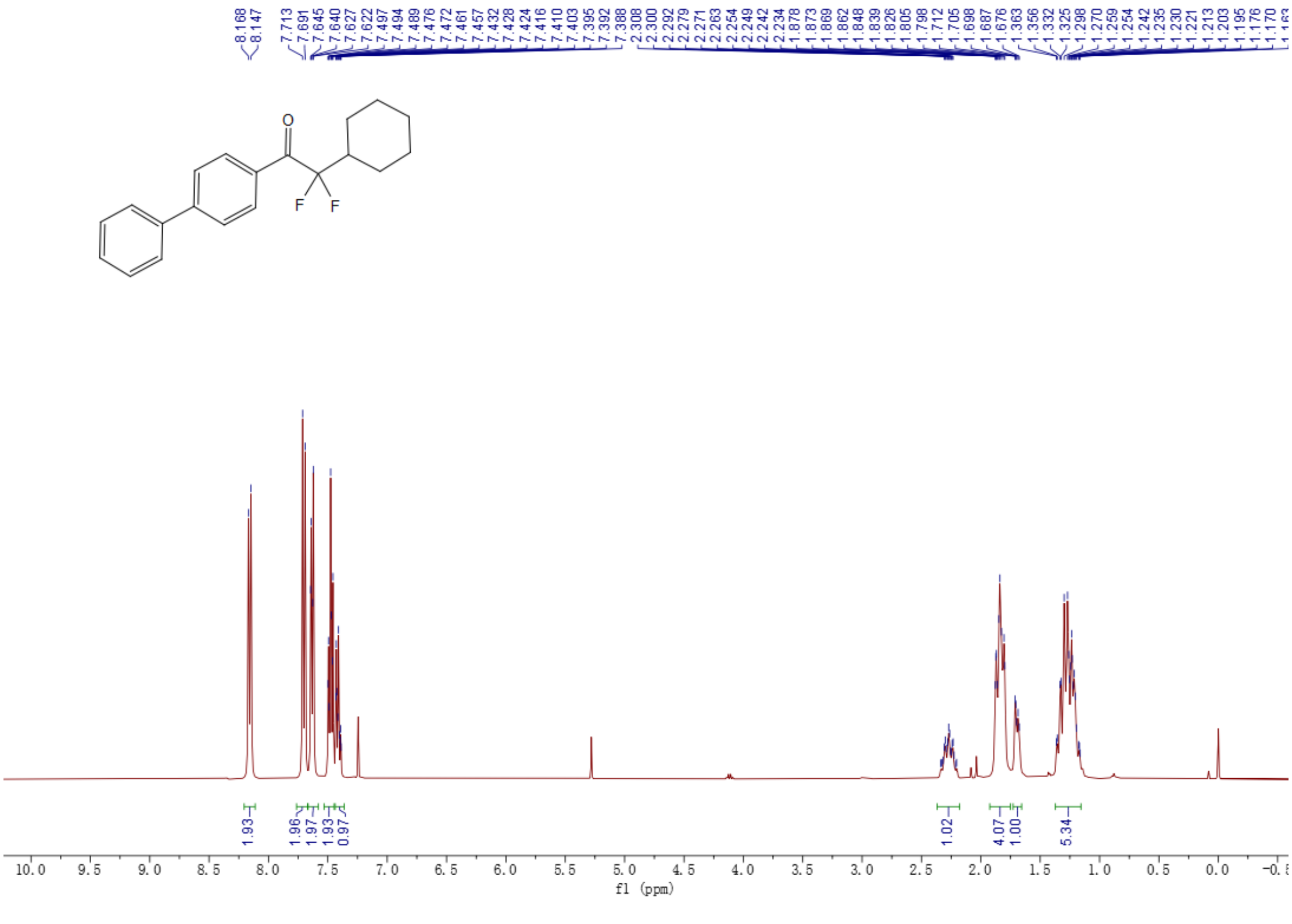

^{19}F NMR spectrum (376 MHz, $CDCl_3$) of compound **3u**

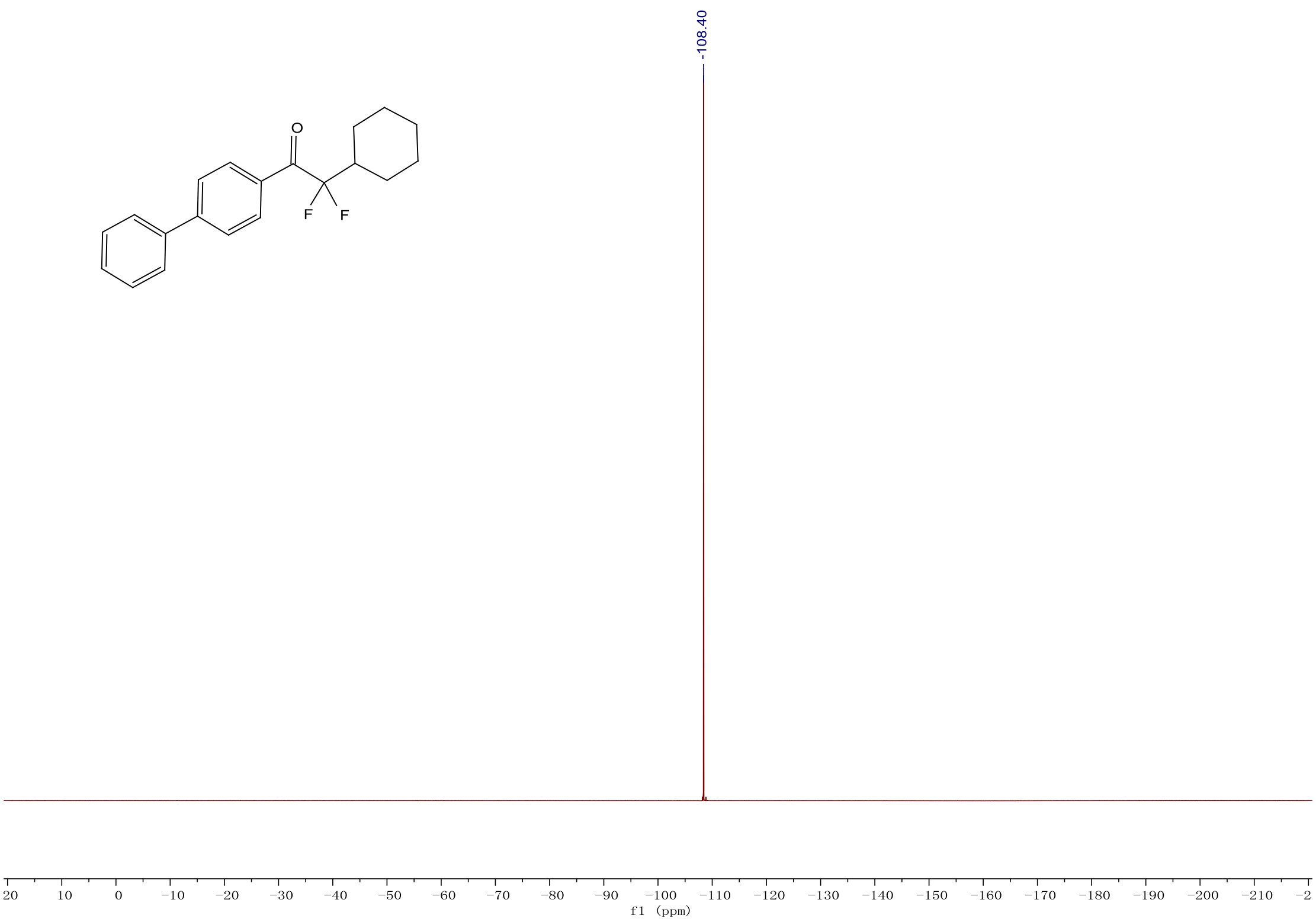

^{13}C NMR spectrum (101 MHz, $CDCl_3$) of compound **3u**

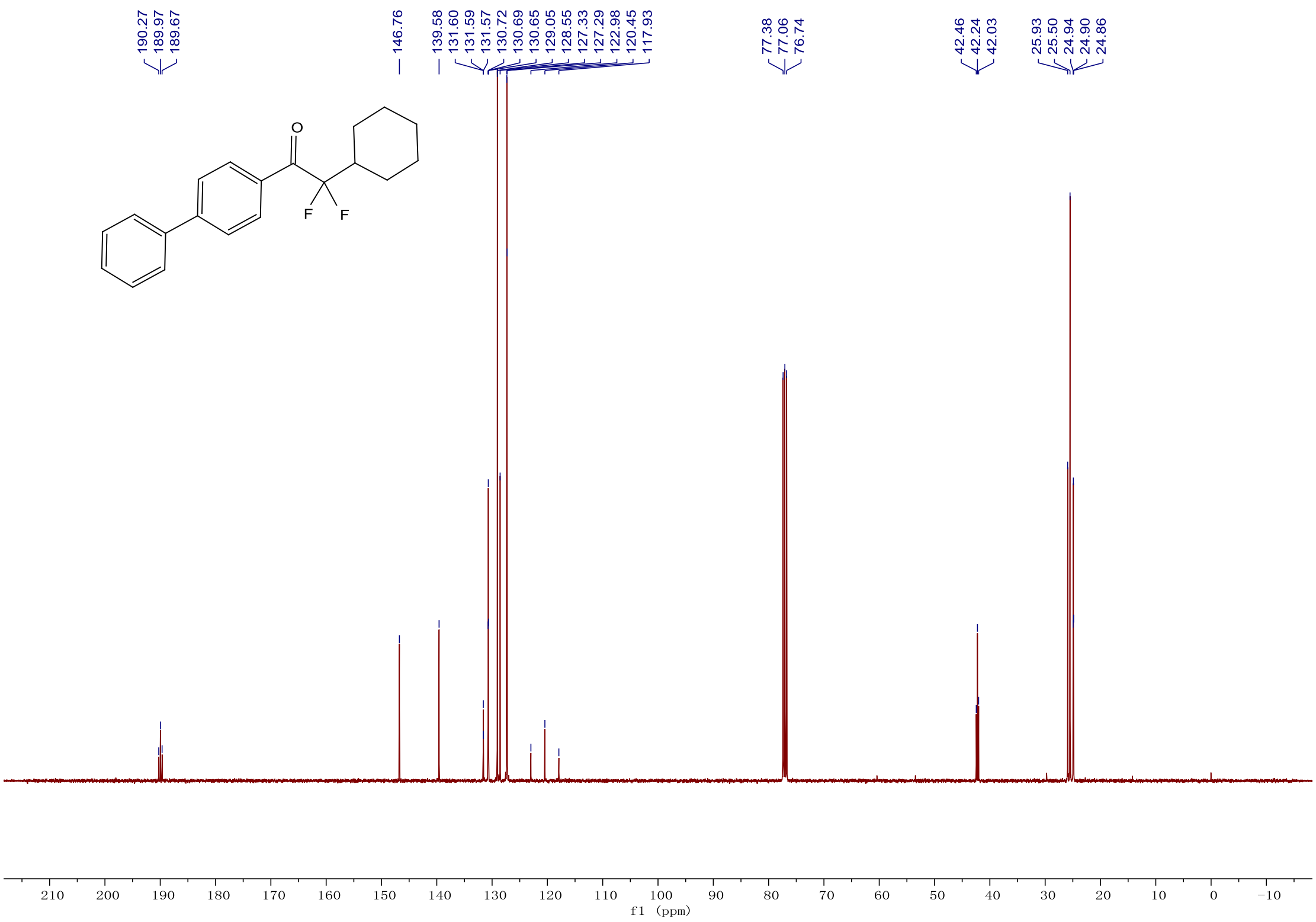

2-cyclohexyl-2,2-difluoro-1-(naphthalen-2-yl)ethan-1-one (3v)

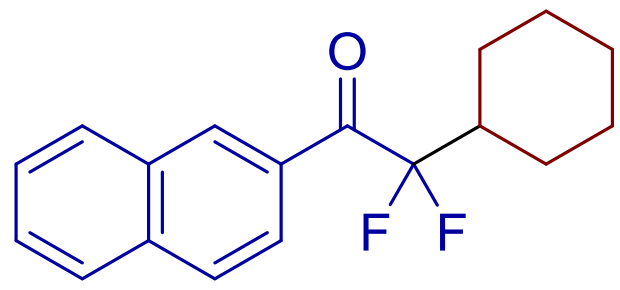

The product (39.2 mg, 68% yield) as light yellow oil was purified with silica gel chromatography (petroleum ether/ ethyl acetate = 20/1). **^{1}H NMR** (400 MHz, Chloroform-*d*) δ 8.67 (s, 1H), 8.08 (d, *J* = 2.0 Hz, 1H), 8.00 (d, *J* = 8.0 Hz, 1H), 7.92-7.87 (m, 2H), 2.35-2.24 (m, 1H), 1.90-1.81 (m, 4H), 1.71-1.68 (m, 1H),1.38-1.17 (m, 5H).**^{19}F NMR** (376 MHz, Chloroform-*d*) δ -107.98 (s, 2F). **^{13}C NMR** (101 MHz, Chloroform-*d*) δ 190.3 (t, *J* = 30.3 Hz), 135.9, 132.5 (t, *J* = 5.0 Hz), 132.3, 130.2, 130.1, 129.3, 128.5, 127.8, 127.0, 124.9 (t, *J* = 3.0 Hz), 120.6 (t, *J* = 255.5 Hz), 42.3 (t, *J* = 22.2 Hz), 25.9, 25.5, 24.9 (t, *J* = 4.0 Hz). **HRMS** (EI-TOF) m/z: $[M+Na]^+$ Calcd. for $C_{18}H_{18}F_2NaO$ 311.1218; Found 311.1228.

^{1}H NMR spectrum (400 MHz, $CDCl_3$) of compound **3v**

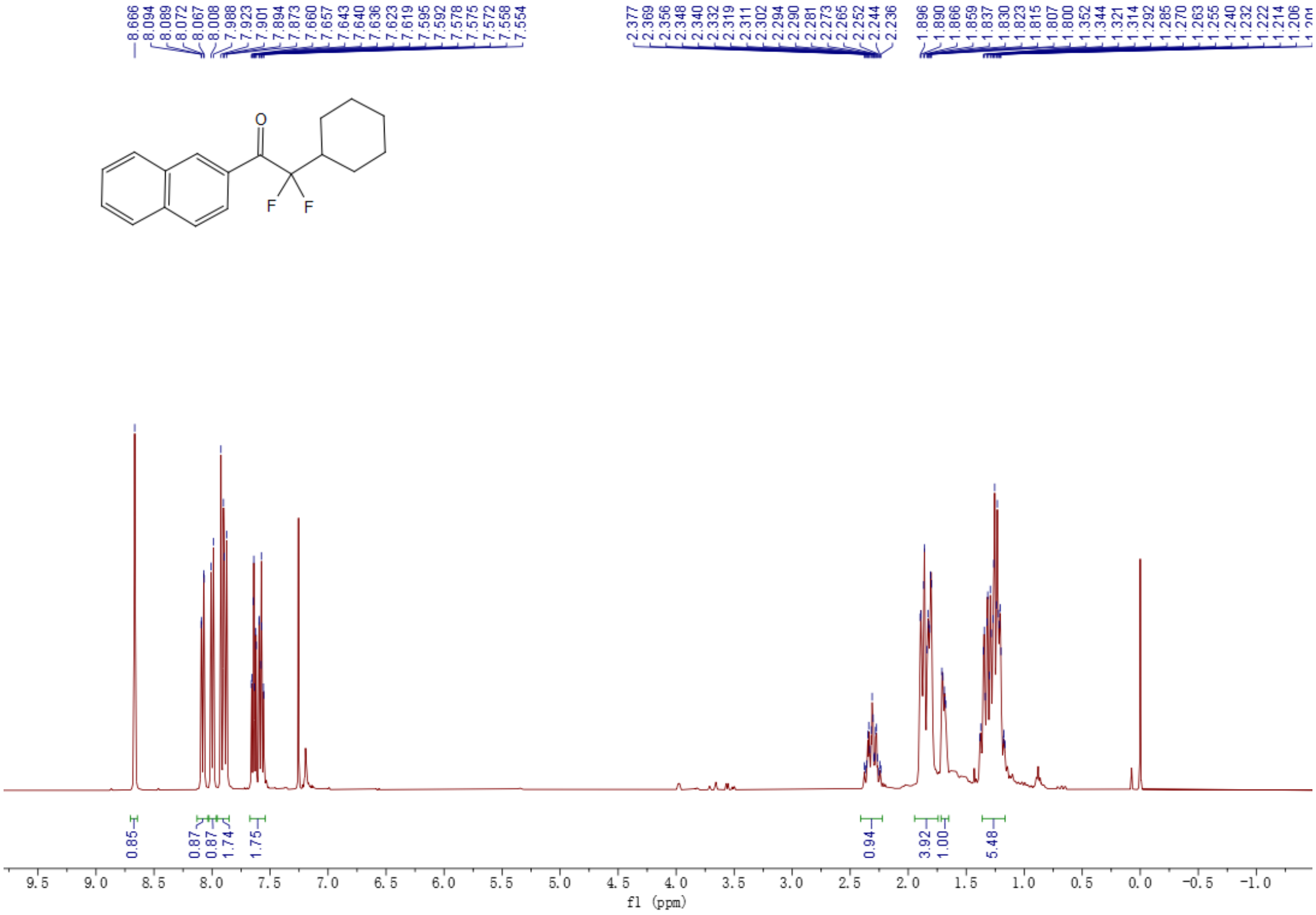

^{19}F NMR spectrum (376 MHz, $CDCl_3$) of compound **3v**

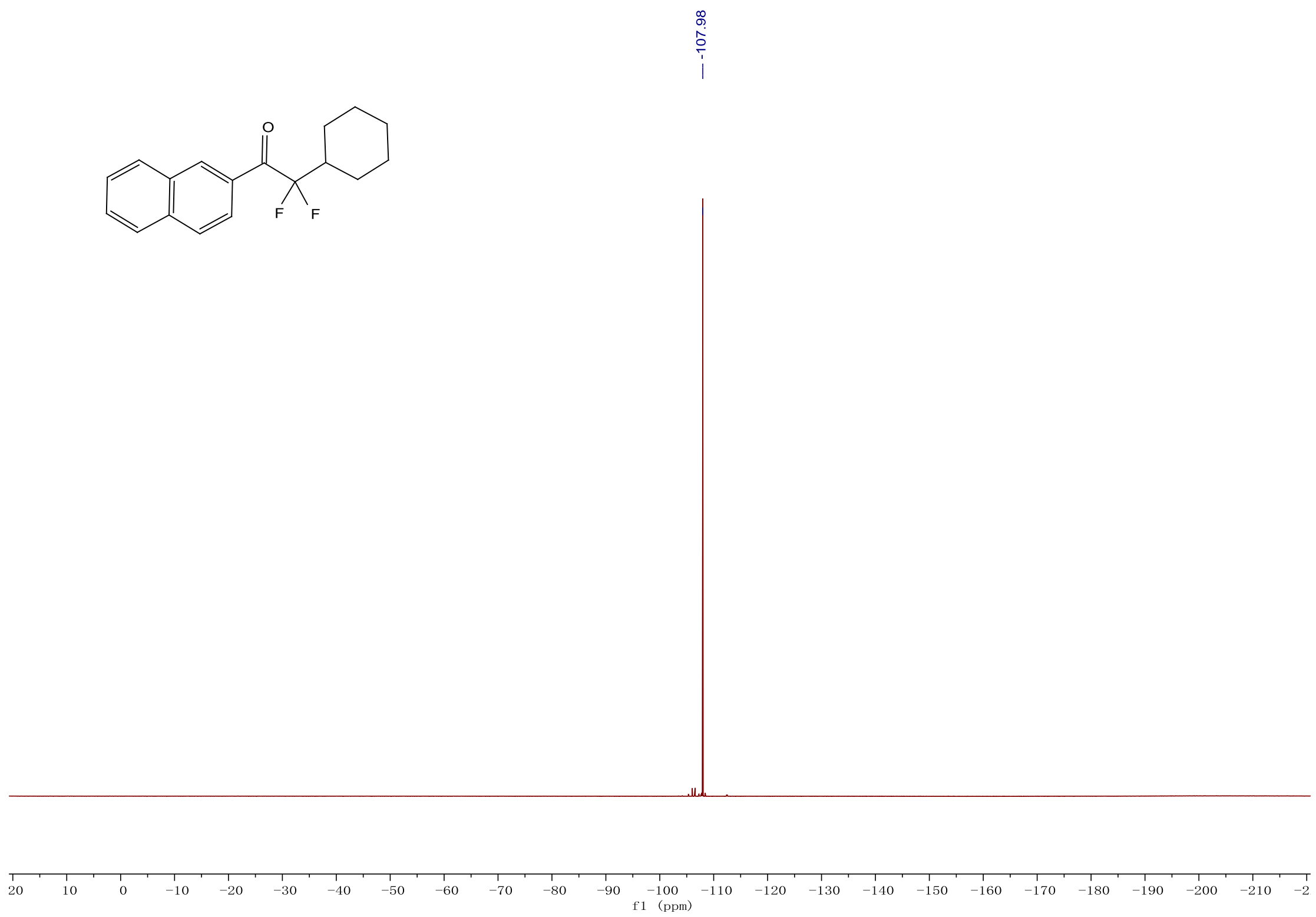

^{13}**C NMR** spectrum (101 MHz, $CDCl_3$) of compound **3v**

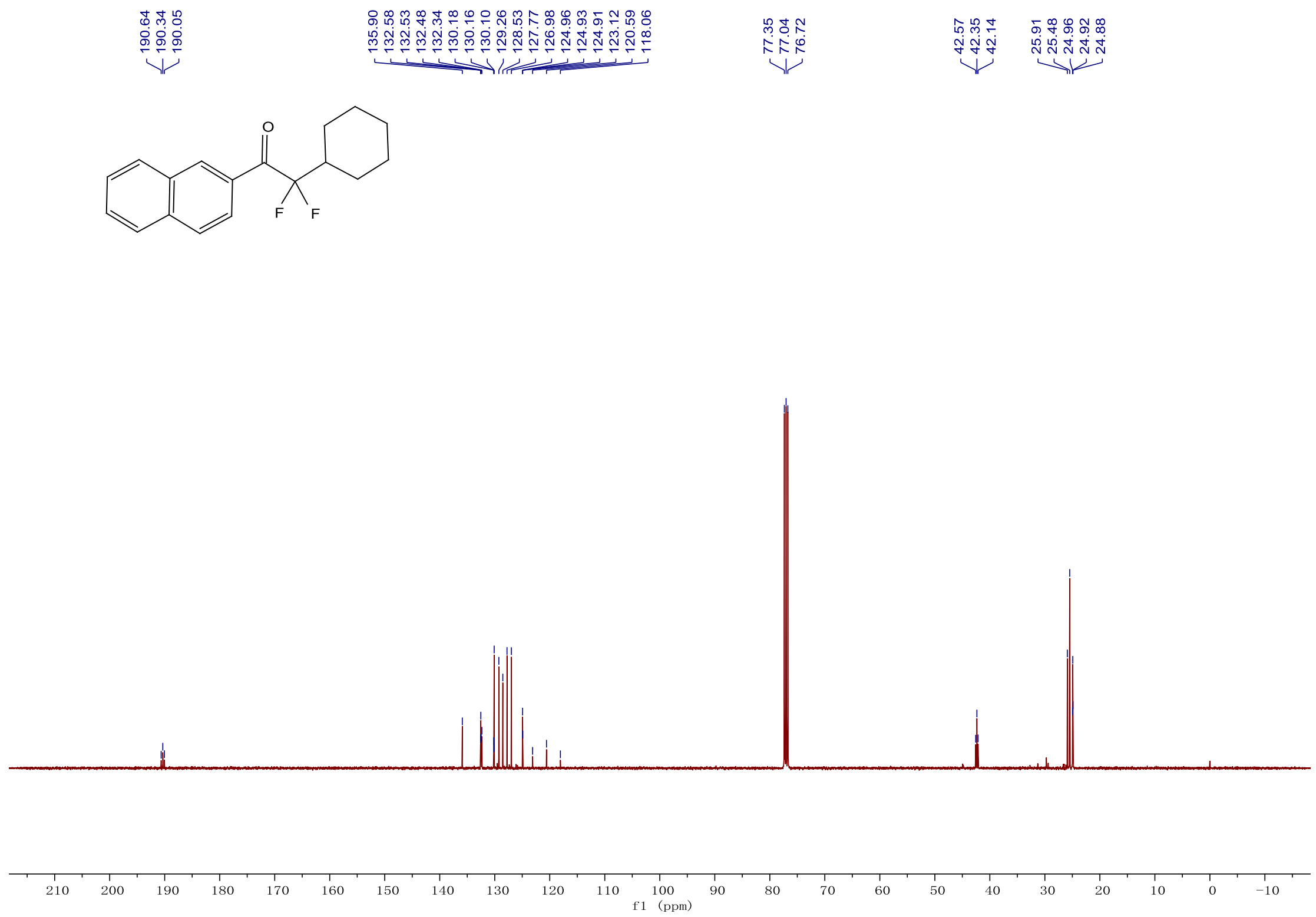

2-cyclohexyl-2,2-difluoro-1-(4-fluorophenyl)ethan-1-one (3w)

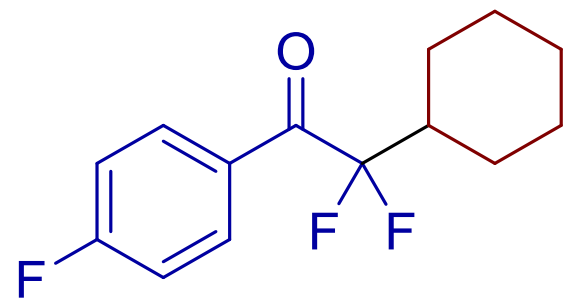

The product (30.3 mg, 59% yield) as colorless oil was purified with silica gel chromatography (petroleum ether/ ethyl acetate = 20/1). **^{1}H NMR** (400 MHz, Chloroform-*d*) δ 8.15-8.11 (m, 2H), 7.19-7.14 (m, 2H), 2.29-2.16 (m, 1H), 1.83-1.80 (m, 4H), 1.72-1.68 (m, 1H),1.34-1.16 (m, 5H).**^{19}F NMR** (376 MHz, Chloroform-*d*) δ -102.75 (s, 1F), -108.15 (s, 2F). **^{13}C NMR** (101 MHz, Chloroform-*d*) δ 188.8 (t, *J* = 31.3 Hz), 167.5, 165.0, 132.0 (dd, *J* = 10.1 Hz, *J* = 4.0 Hz), 129.3 (t, *J* = 2.0 Hz), 120.3 (t, *J* = 255.5 Hz), 116.1, 115.8, 42.0 (t, *J* = 21.2 Hz), 25.9, 25.5, 24.8 (t, *J* = 4.0 Hz). **HRMS** (EI-TOF) m/z: $[M+Na]^+$ Calcd. for $C_{14}H_{15}F_3NaO$ 279.0967; Found 279.0969.

^{1}H NMR spectrum (400 MHz, $CDCl_3$) of compound **3w**

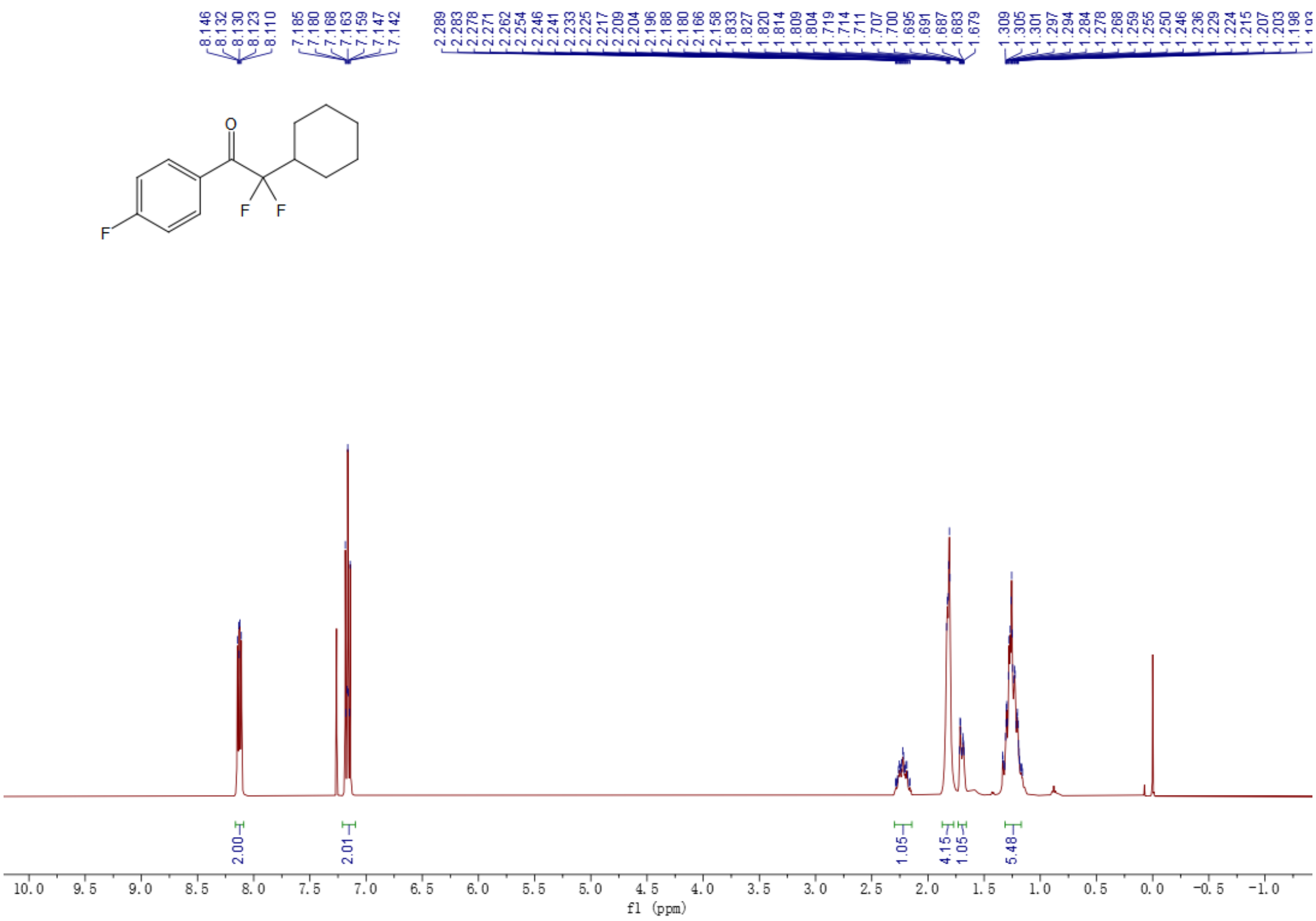

^{19}F NMR spectrum (376 MHz, $CDCl_3$) of compound **3w**

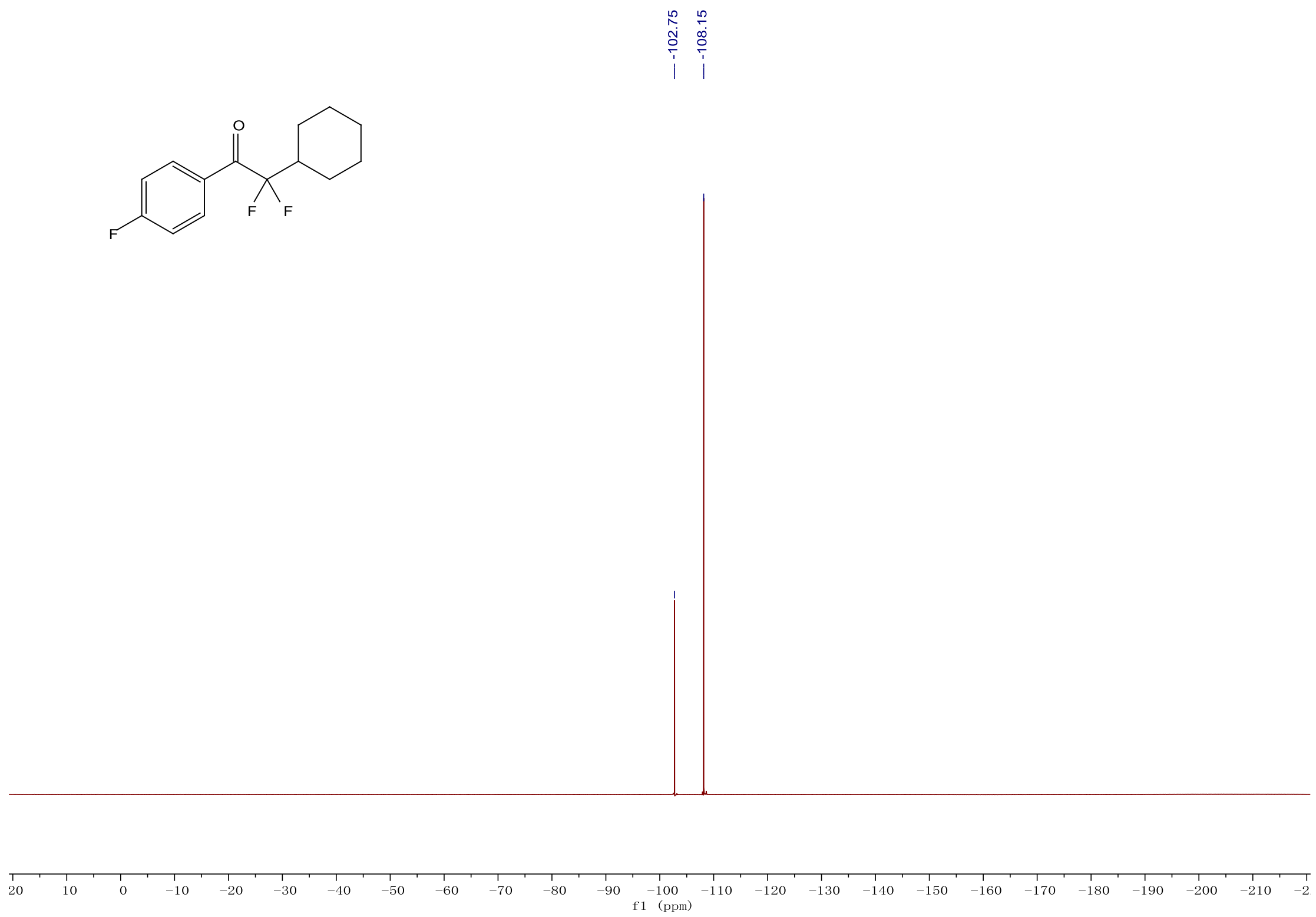

^{13}C NMR spectrum (101 MHz, $CDCl_3$) of compound **3w**

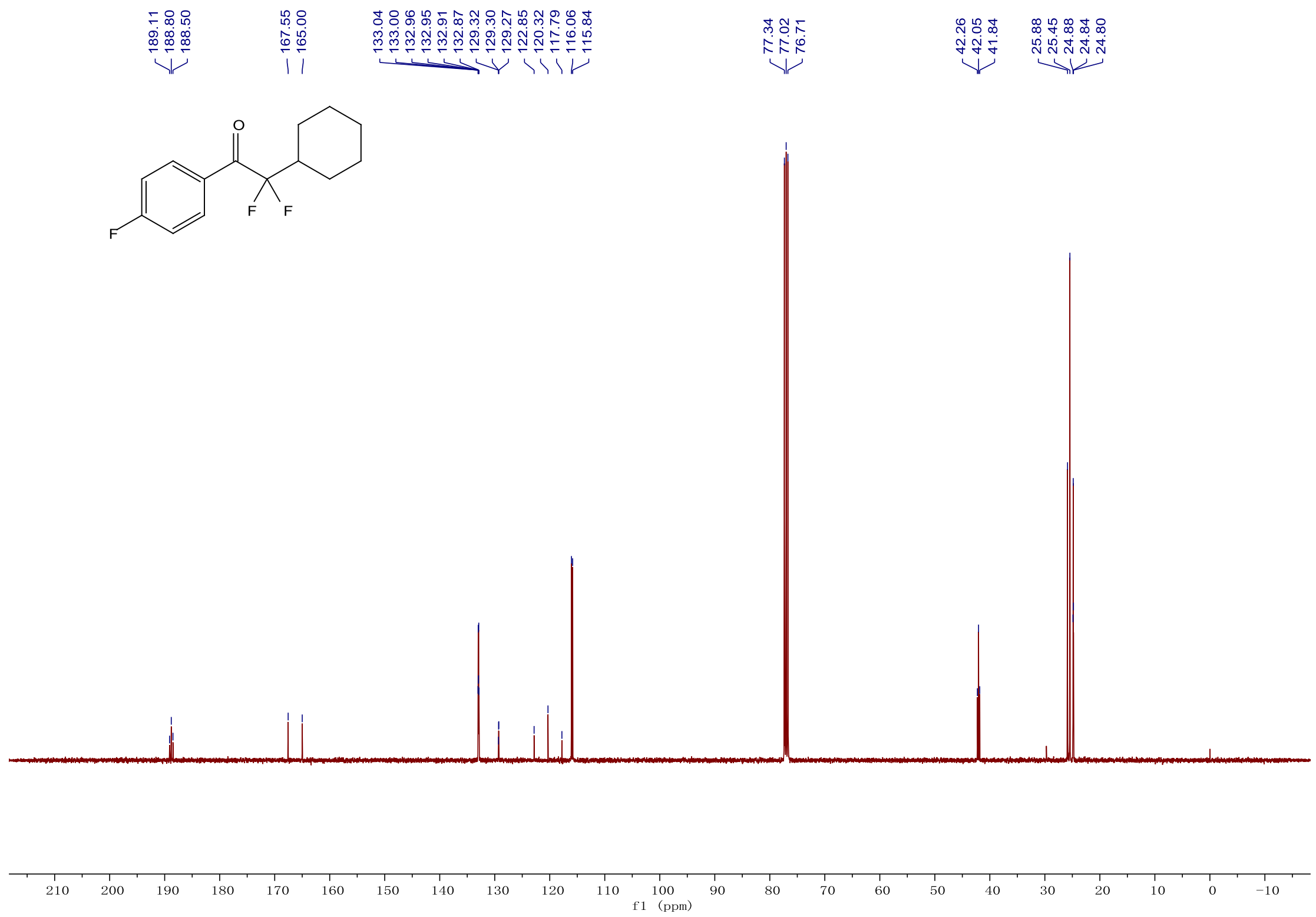

1-(4-chlorophenyl)-2-cyclohexyl-2,2-difluoroethan-1-one (3x)

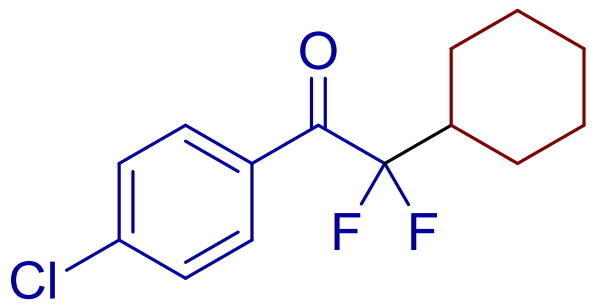

The product (28.8 mg, 53% yield) as colorless oil was purified with silica gel chromatography (petroleum ether/ ethyl acetate = 20/1). **^{1}H NMR** (400 MHz, Chloroform-*d*) δ 8.02 (d, *J* = 8.8 Hz, 2H), 7.48-7.45 (m, 2H), 2.28-2.15 (m, 1H), 1.82-1.80 (m, 4H), 1.71-1.68 (m, 1H),1.33-1.20 (m, 5H).**^{19}F NMR** (376 MHz, Chloroform-*d*) δ -108.30 (s, 2F). **^{13}C NMR** (101 MHz, Chloroform-*d*) δ 189.2 (t, *J* = 31.3 Hz), 140.8, 131.4 (t, *J* = 4.0 Hz), 131.2 (t, *J* = 2.0 Hz), 129.1, 120.3 (t, *J* = 254.5 Hz), 42.0 (t, *J* = 21.2 Hz), 25.9, 25.4, 24.8 (t, *J* = 4.0 Hz). **HRMS** (EI-TOF) m/z: $[M+Na]^+$ Calcd. for $C_{14}H_{15}ClF_2NaO$ 295.0672; Found 295.0674.

^{1}H NMR spectrum (400 MHz, $CDCl_3$) of compound **3x**

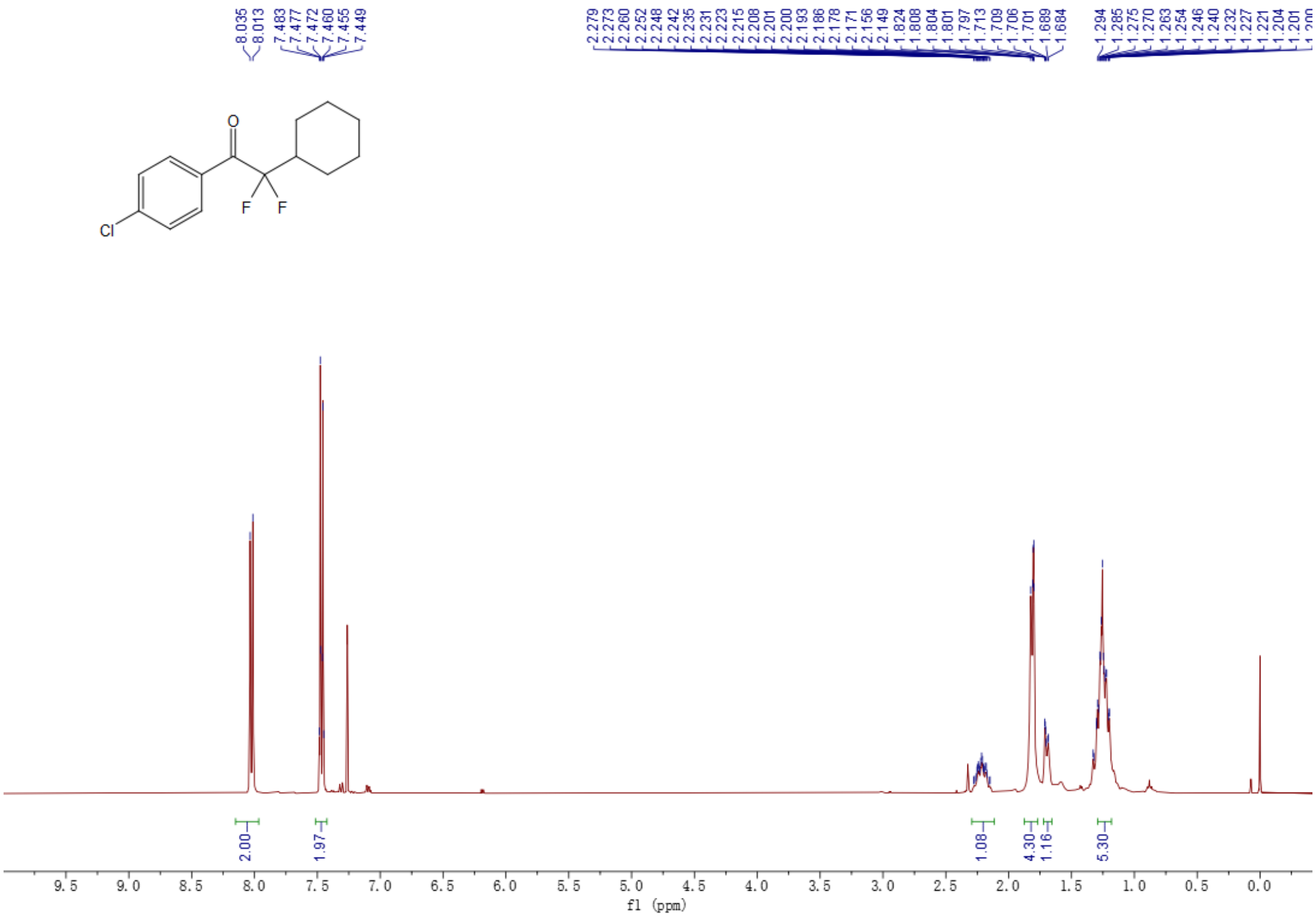

^{19}F NMR spectrum (376 MHz, $CDCl_3$) of compound **3x**

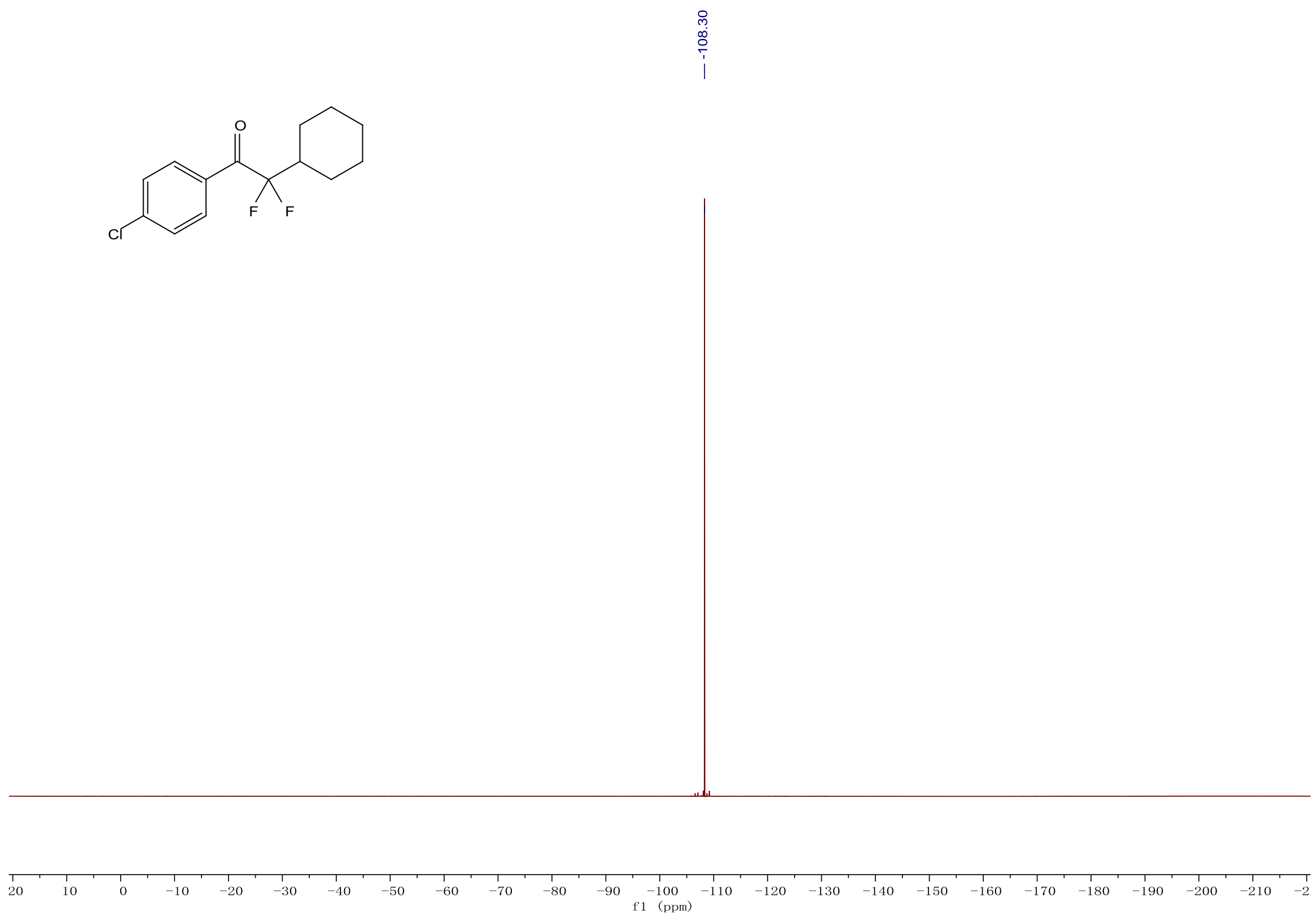

^{13}**C NMR** spectrum (101 MHz, $CDCl_3$) of compound **3x**

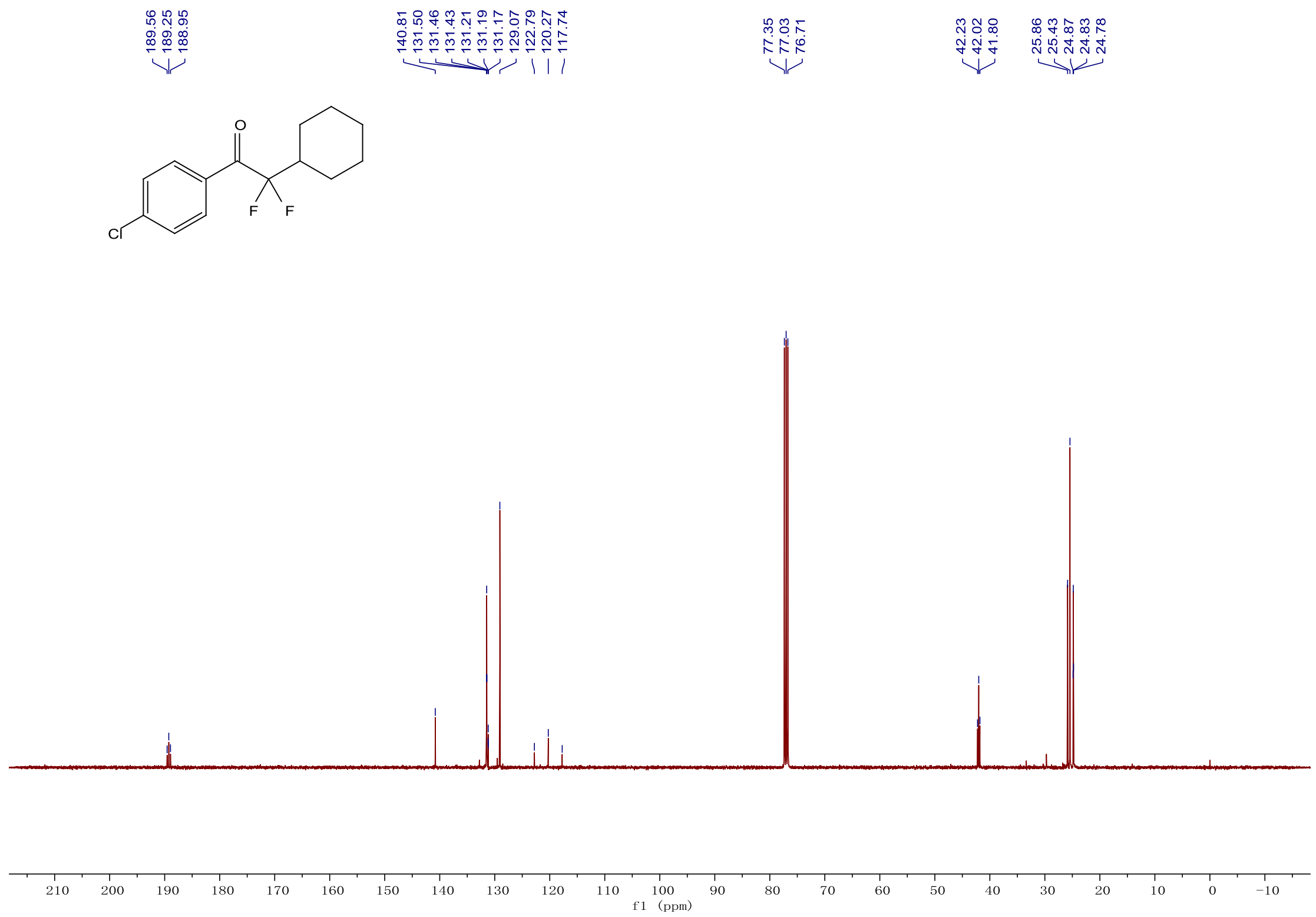

methyl 4-(2-cyclohexyl-2,2-difluoroacetyl)benzoate (3y)

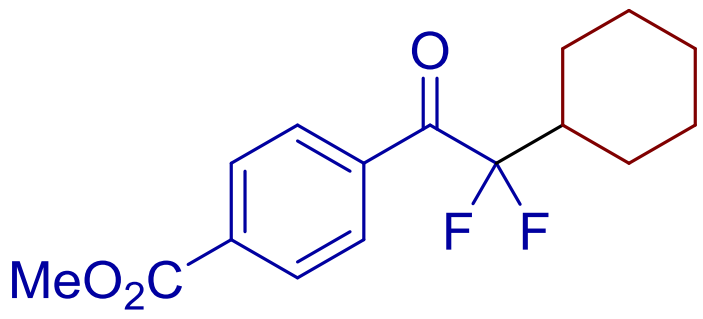

The product (35.5 mg, 60% yield) as white solid was purified with silica gel chromatography (petroleum ether/ ethyl acetate = 10/1). **^{1}H NMR** (400 MHz, Chloroform-*d*) δ 8.16-8.11 (m, 4H), 3.96 (s, 3H), 2.30-2.17 (m, 1H), 1.85-1.81 (m, 4H), 1.72-1.68 (m, 1H), 1.34-1.16 (m, 5H). **^{19}F NMR** (376 MHz, Chloroform-*d*) δ -108.50 (s, 2F). **^{13}C NMR** (101 MHz, Chloroform-*d*) δ 190.1 (t, *J* = 31.3 Hz), 166.0,136.2 (t, *J* = 2.0 Hz), 134.6, 129.9 (t, *J* = 3.0 Hz), 129.7, 120.2(t, *J* = 255.5 Hz), 52.6, 41.9, 25.9, 25.4, 24.8 (t, *J* = 4.0 Hz). **HRMS** (EI-TOF) m/z: $[M+Na]^+$ Calcd. for $C_{16}H_{19}F_2NaO_3$ 319.1116; Found 319.1122.

^{1}H NMR spectrum (400 MHz, $CDCl_3$) of compound **3y**

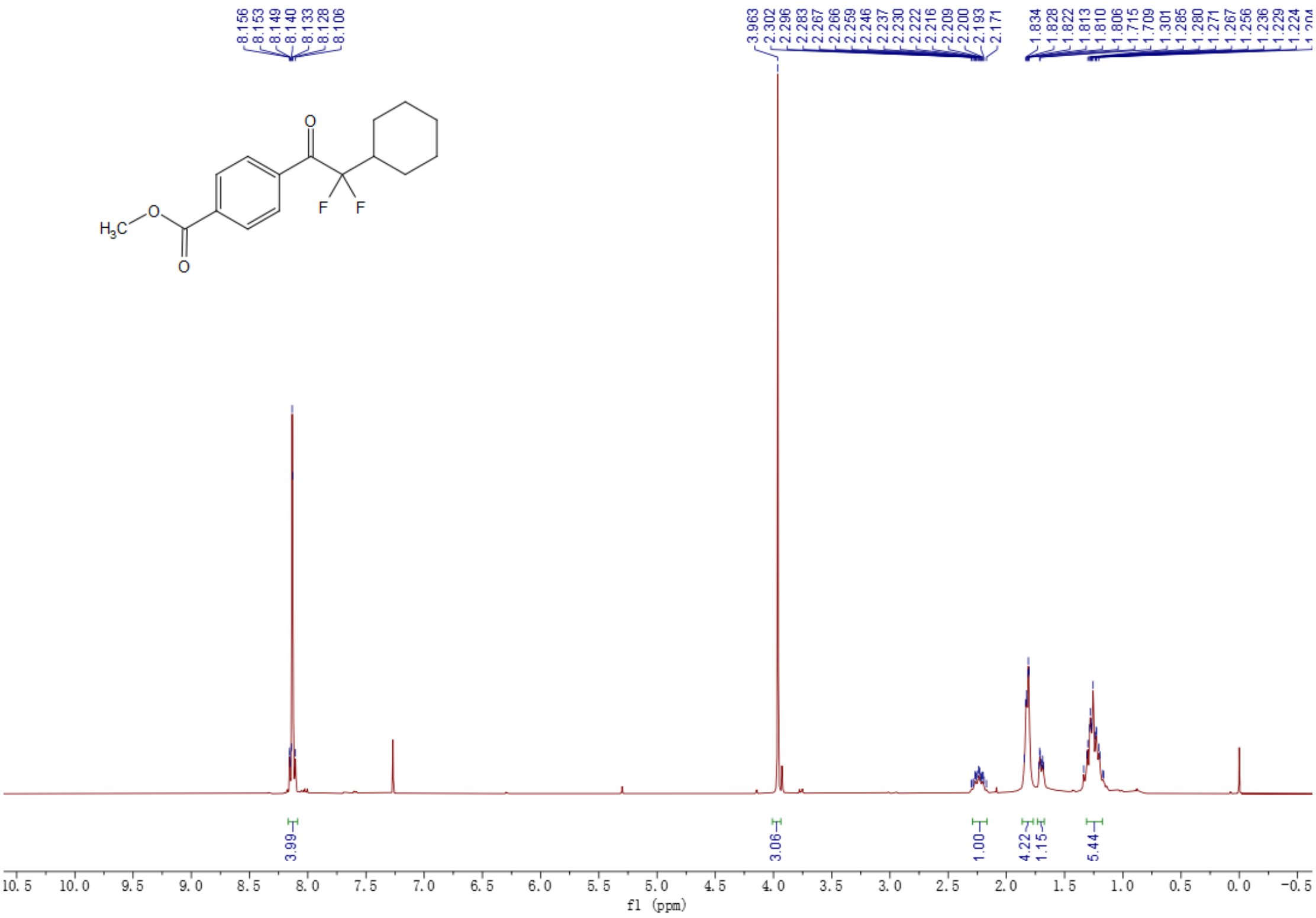

^{19}F NMR spectrum (376 MHz, $CDCl_3$) of compound **3y**

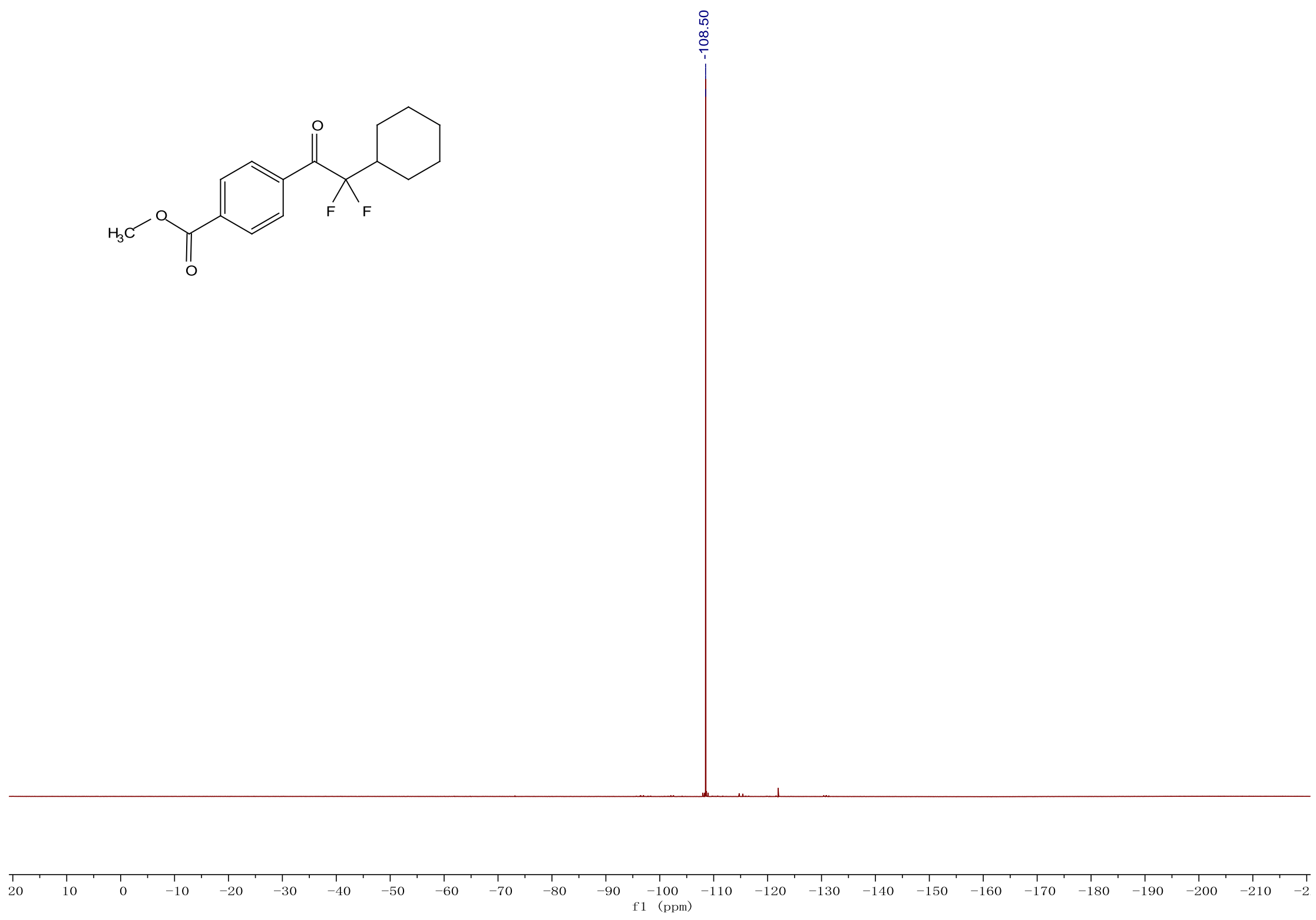

^{13}C NMR spectrum (101 MHz, $CDCl_3$) of compound **3y**

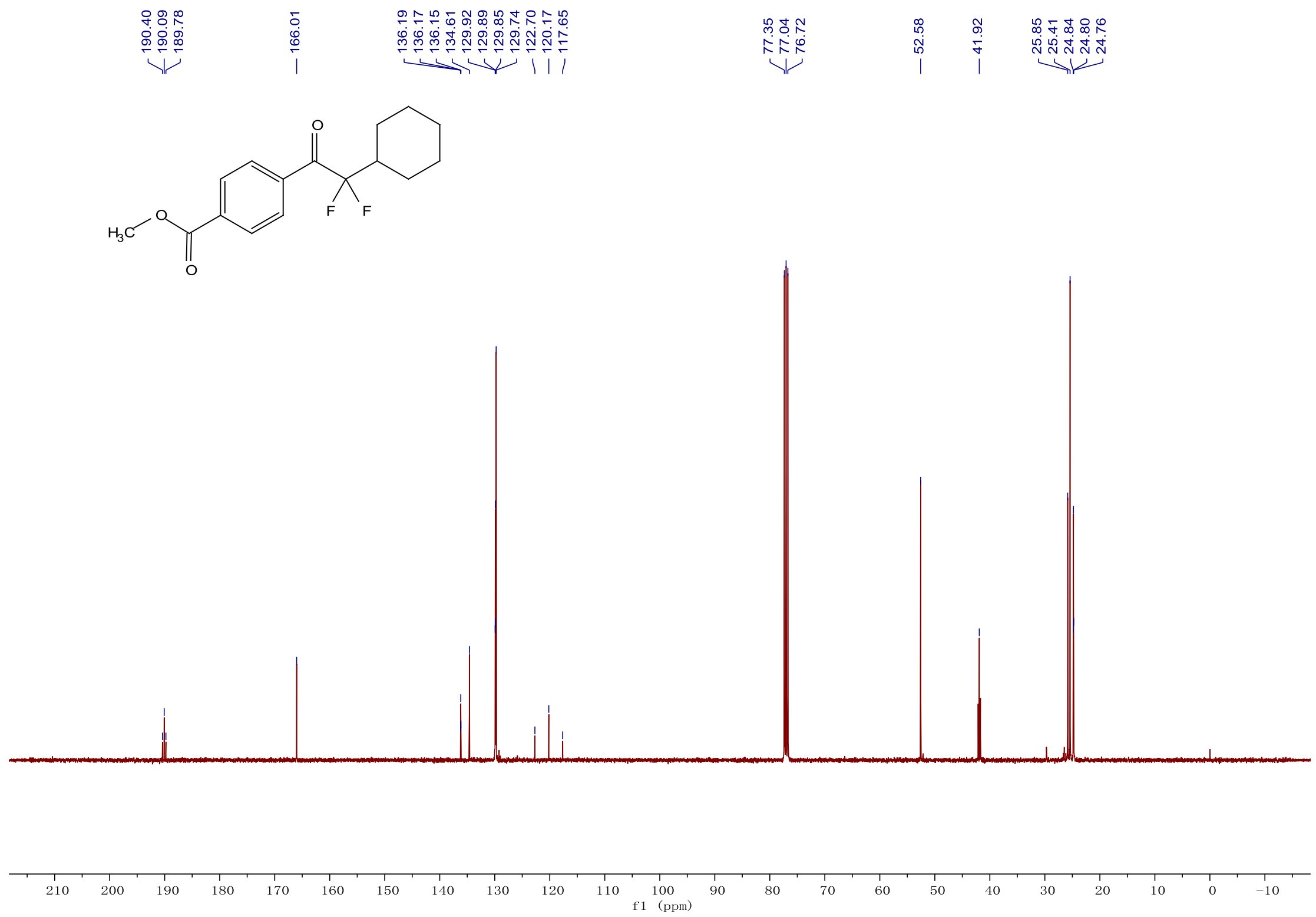

4-(2-cyclohexyl-2,2-difluoroacetyl)phenyl 4-methylbenzenesulfonate (3z)

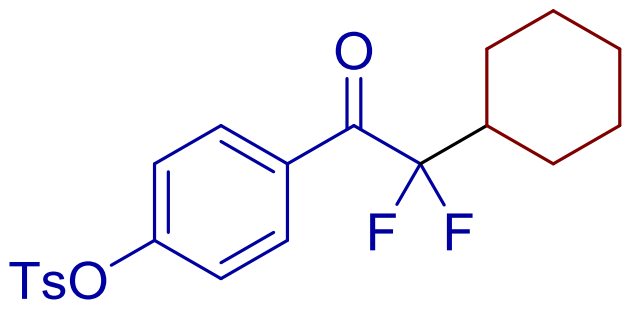

The product (53.9 mg, 66% yield) as white solid was purified with silica gel chromatography (petroleum ether/ ethyl acetate = 10/1). ^{1}**H NMR** (400 MHz, Chloroform-*d*) δ 8.02 (d, *J* = 9.2 Hz, 2H), 7.74 (d, *J* = 8.4 Hz, 2H), 7.34 (d, *J* = 8.4 Hz, 2H), 7.14-7.11 (m, 2H), 2.46 (s, 3H), 2.27-2.14 (m, 1H), 1.82-1.79 (m, 4H), 1.71-1.68 (m, 1H), 1.31-1.18 (m, 5H). ^{19}**F NMR** (376 MHz, Chloroform-*d*) δ -108.16 (s, 2F). ^{13}**C NMR** (101 MHz, Chloroform-*d*) δ 189.0 (t, *J* = 29.3 Hz), 153.6, 145.9, 132.1, 132.0 (t, *J* = 4.0 Hz), 131.4 (t, *J* = 2.0 Hz), 130.0, 128.5, 122.6, 120.2 (t, *J* = 255.5 Hz), 41.9 (t, *J* = 21.2 Hz), 25.8, 25.4, 24.8 (t, *J* = 5.0 Hz), 21.8. **HRMS** (EI-TOF) m/z: $[M+Na]^+$ Calcd. for $C_{21}H_{22}F_2NaO_4S$ 431.1099; Found 431.1098.

^{1}**H NMR** spectrum (400 MHz, $CDCl_3$) of compound **3z**

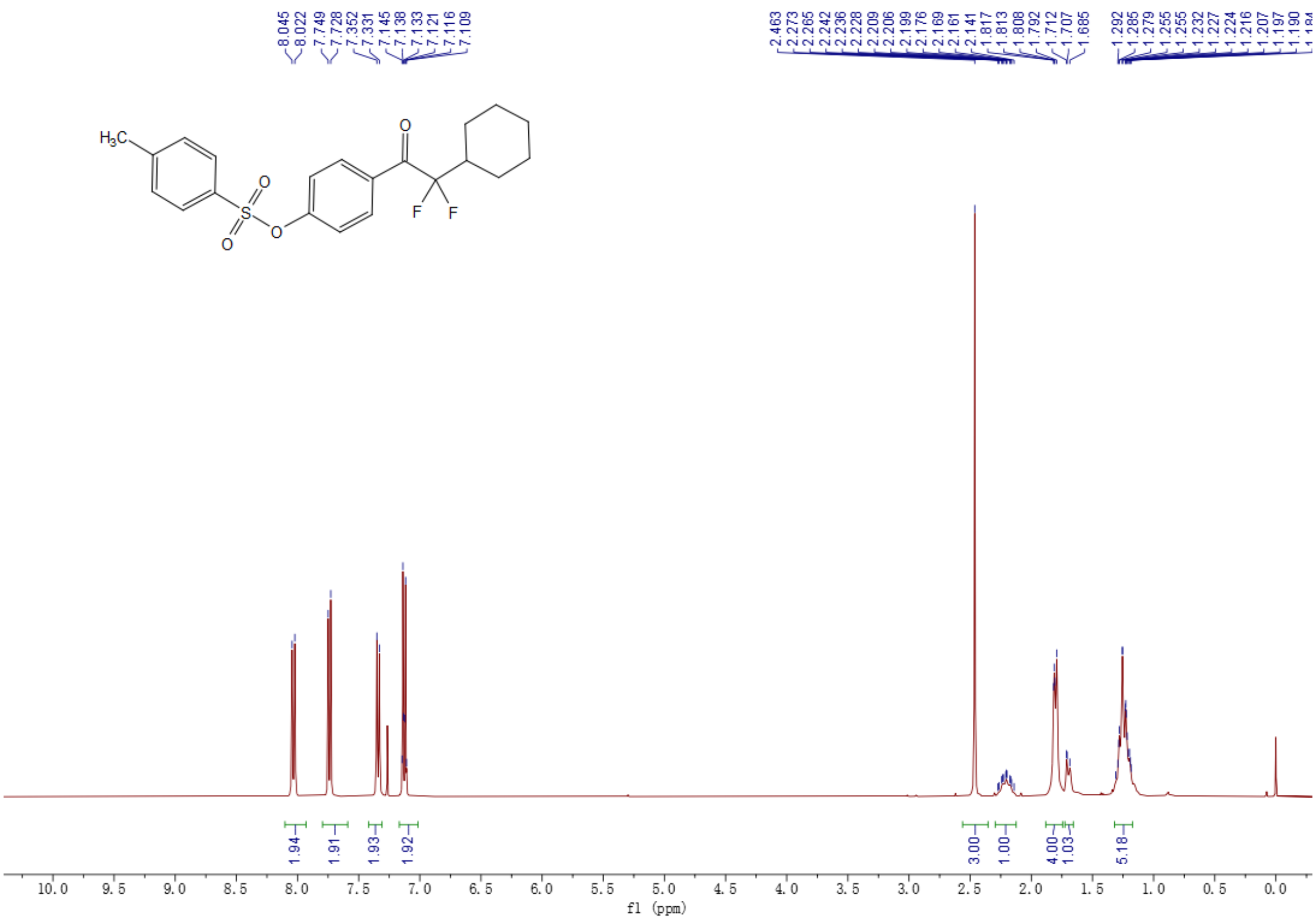

^{19}**F NMR** spectrum (376 MHz, $CDCl_3$) of compound **3z**

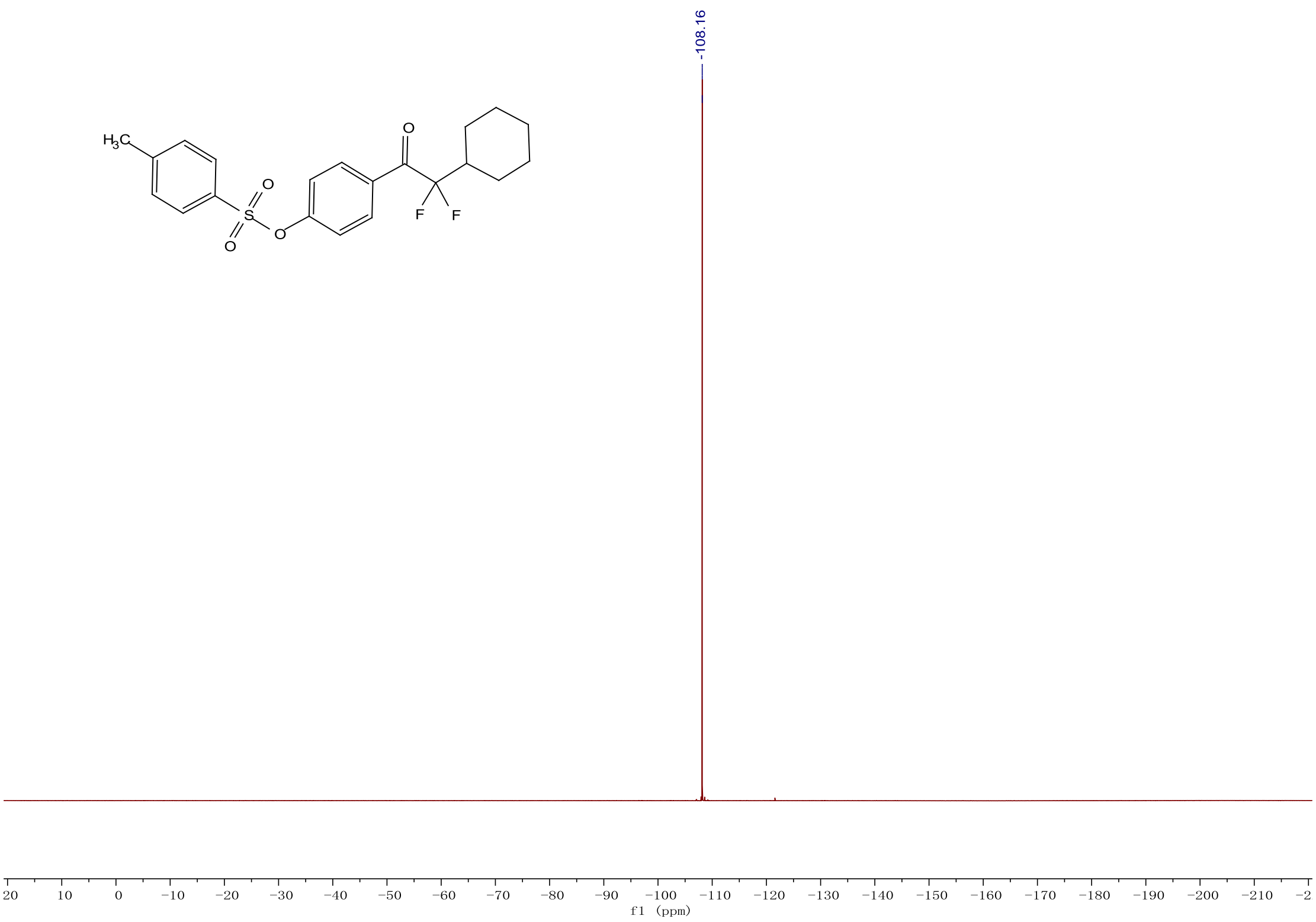

^{13}C NMR spectrum (101 MHz, $CDCl_3$) of compound **3z**

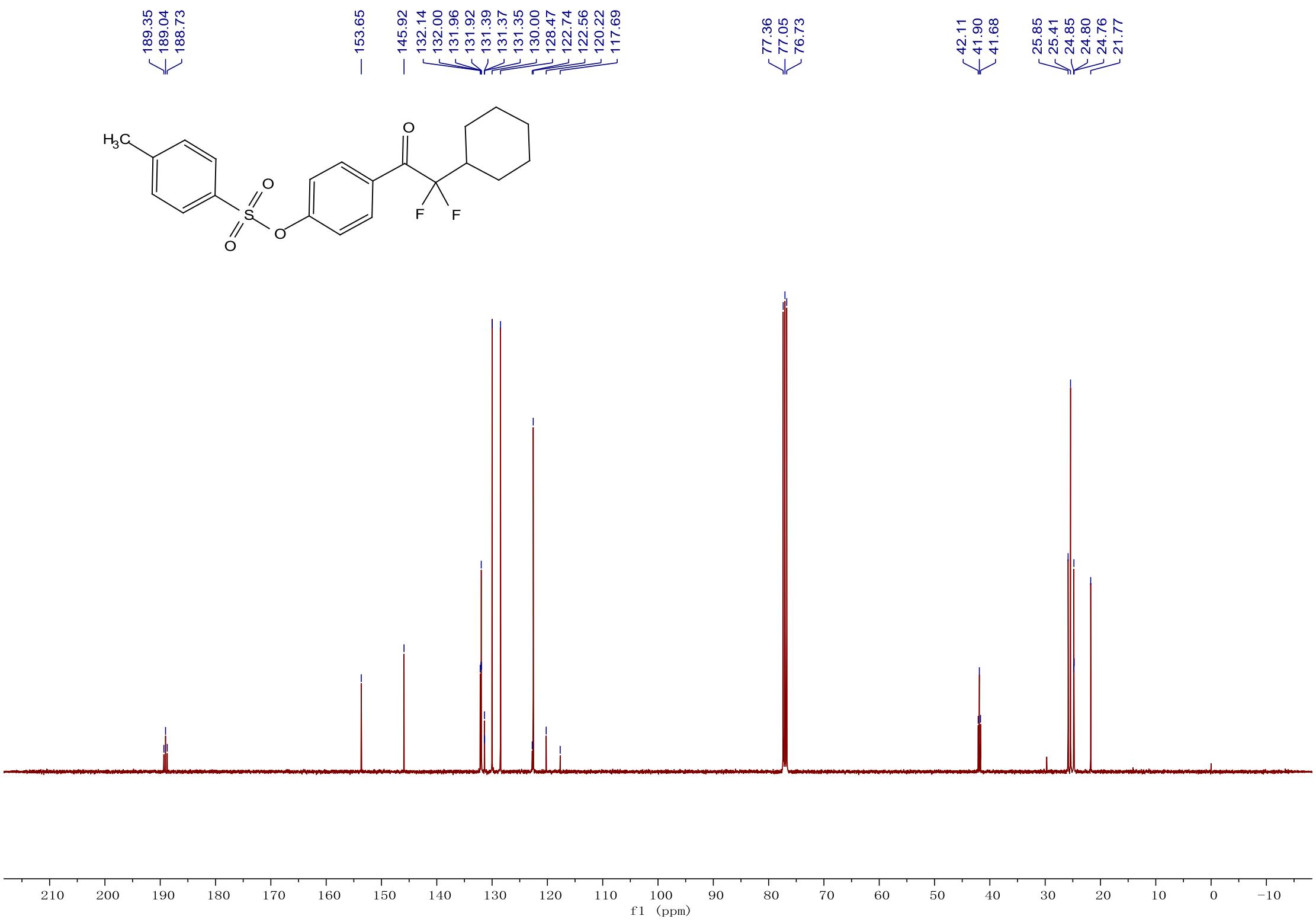

2-cyclohexyl-2,2-difluoro-1-(4-methoxyphenyl)ethan-1-one (3aa)

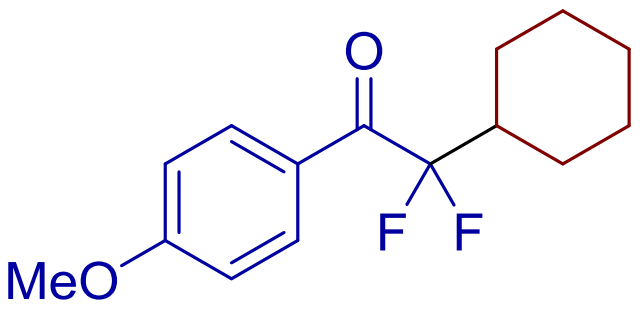

The product (40.8 mg, 76% yield) as colorless oil was purified with silica gel chromatography (petroleum ether/ ethyl acetate = 20/1). **^{1}H NMR** (400 MHz, Chloroform-*d*) δ 8.08 (d, *J* = 9.2 Hz, 2H), 6.95 (d, *J* = 9.2 Hz, 2H), 3.88 (s, 3H), 2.28-2.15 (m, 1H), 1.85-1.79 (m, 4H), 1.70-1.67 (m, 1H), 1.32-1.18 (m, 5H). **^{19}F NMR** (376 MHz, Chloroform-*d*) δ -108.24 (s, 2F). **^{13}C NMR** (101 MHz, Chloroform-*d*) δ 188.7 (t, *J* = 29.3 Hz), 164.3, 132.6 (t, *J* = 4.0 Hz), 125.8 (t, *J* = 2.0 Hz), 120.6 (t, *J* = 254.5 Hz), 113.9, 55.5, 42.4 (t, *J* = 22.2 Hz), 25.9, 25.5, 24.9 (t, *J* = 4.0 Hz). **HRMS** (EI-TOF) m/z: $[M+Na]^+$ Calcd. for $C_{15}H_{18}F_2NaO_2$ 291.1167; Found 291.1171.

^{1}H NMR spectrum (400 MHz, $CDCl_3$) of compound **3aa**

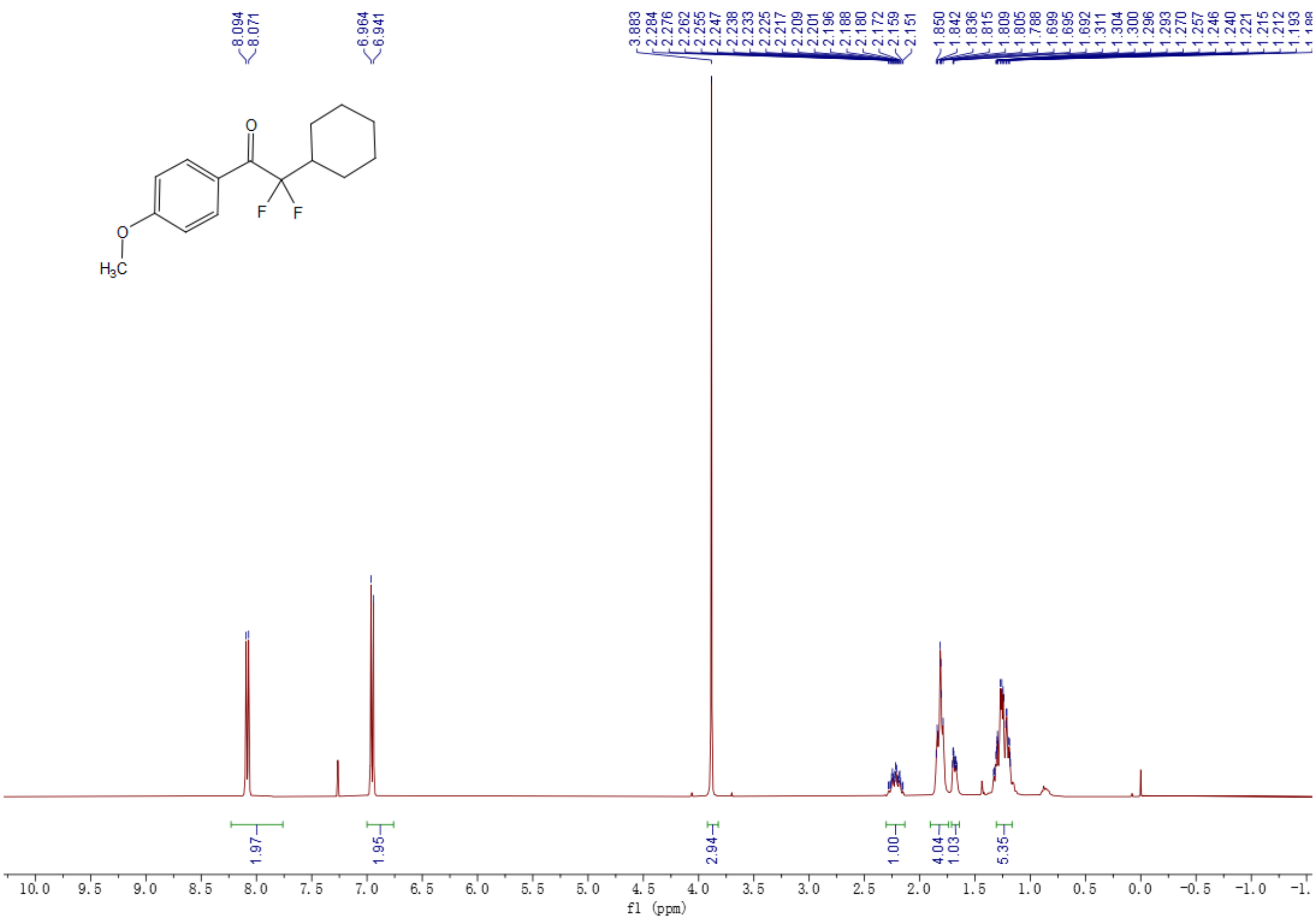

^{19}F NMR spectrum (376 MHz, $CDCl_3$) of compound **3aa**

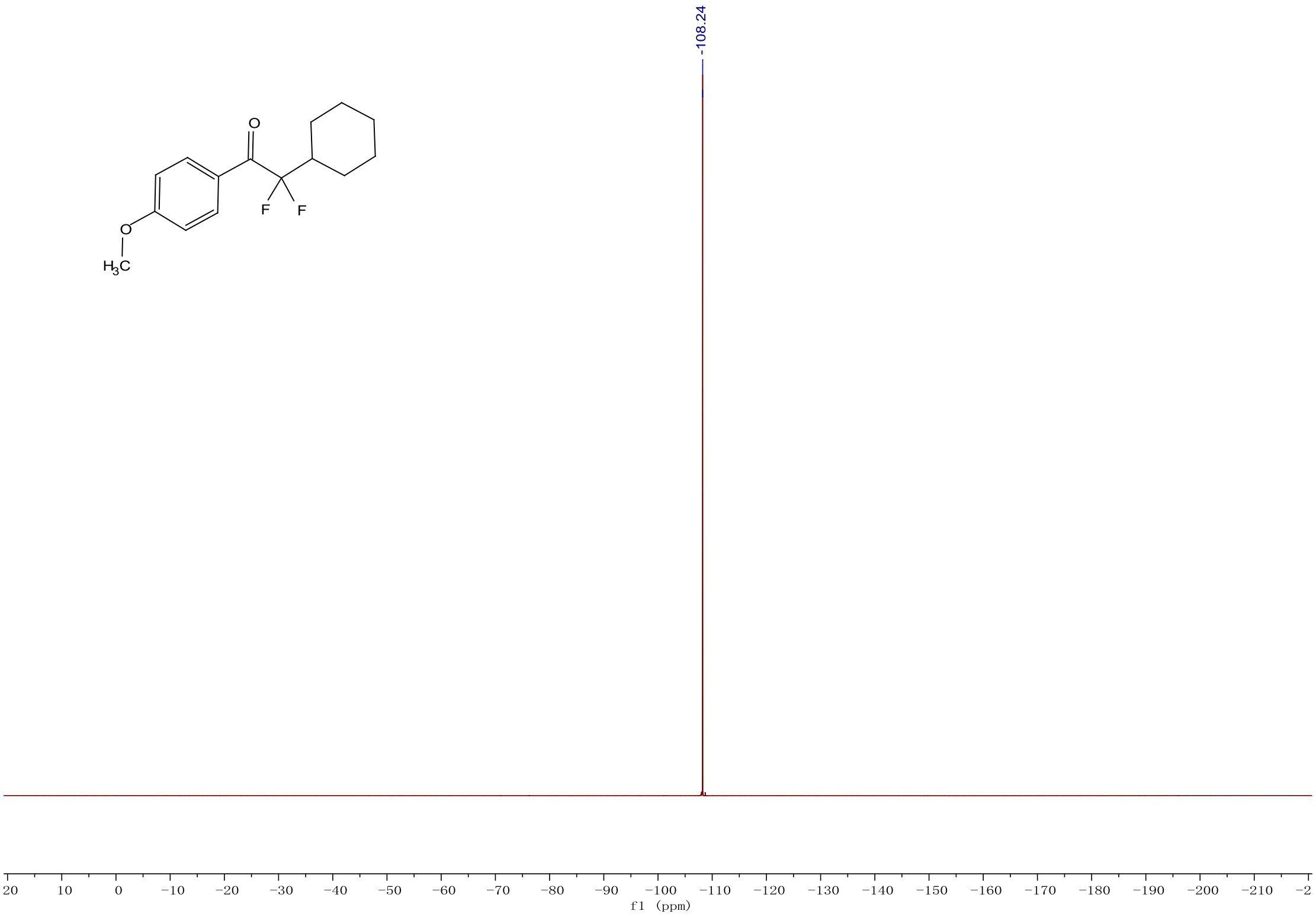

^{13}**C NMR** spectrum (101 MHz, $CDCl_3$) of compound **3aa**

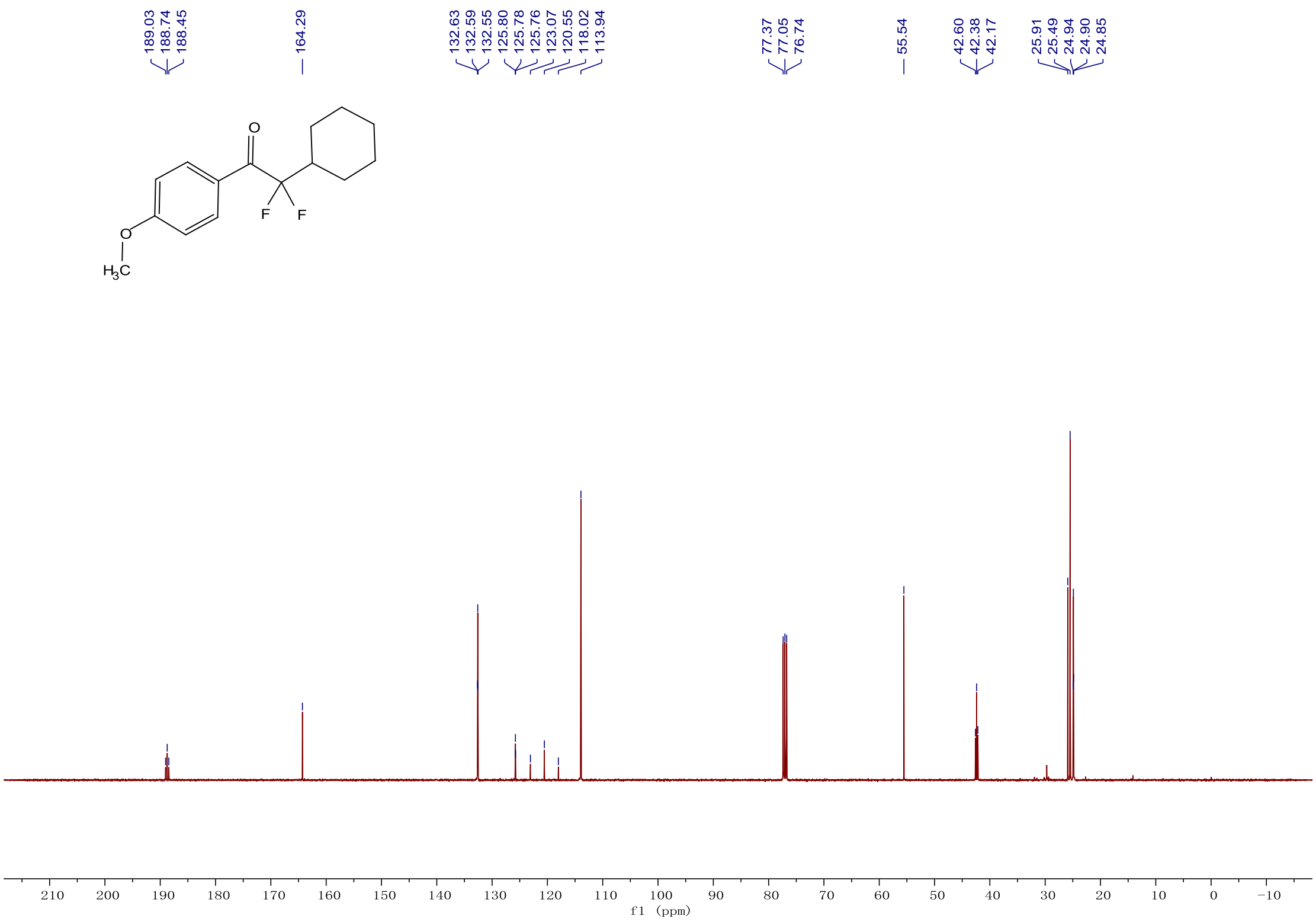

2-cyclohexyl-2,2-difluoro-1-(3-fluoro-4-methoxyphenyl)ethan-1-one (3ab)

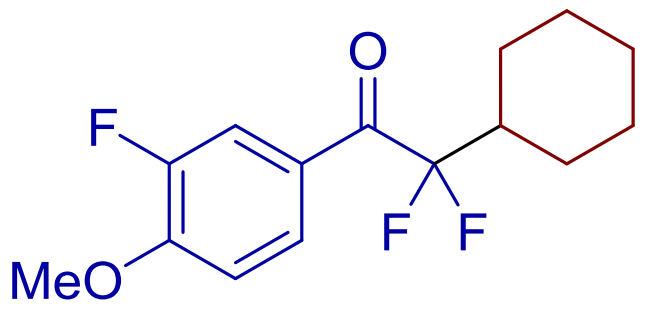

The product (39.5 mg, 69% yield) as white solid was purified with silica gel chromatography (petroleum ether/ ethyl acetate = 20/1).^{1}**H NMR** (400 MHz, Chloroform-*d*) δ 7.93-7.90 (m, 1H), 7.83 (dd, *J* = 12 Hz, *J* = 2.0 Hz， 1H), 7.02 (t, *J* = 8.4 Hz, 1H), 3.98 (s, 3H) 2.26-2.13 (m, 1H), 1.82-1.80 (m, 4H), 1.69 (d, *J* = 9.6Hz, 1H), 1.29-1.19 (m, 5H).^{19}**F NMR** (376 MHz, Chloroform-*d*) δ -108.06 (s, 2F), -133.77 (s, 1F). ^{13}**C NMR** (101 MHz, Chloroform-*d*) δ 188.0 (td, *J* = 30.3 Hz, *J* = 2.0 Hz), 152.9 (t, *J* = 22.2 Hz), 150.5, 127.9 (dd, *J* = 8.1 Hz, *J* = 4.0 Hz), 125.8-125.8 (m), 122.9 (t, *J* = 255.5 Hz), 117.7 (dt, *J* = 7.1 Hz, *J* = 4.0 Hz), 112.4, 56.3, 42.2 (t, *J* = 21.2 Hz), 25.9, 25.5, 24.9 (t, *J* = 5.0 Hz). **HRMS** (EI-TOF) m/z: $[M+Na]^+$ Calcd. for $C_{15}H_{17}F_3NaO_2$ 309.1073; Found 309.1082.

^{1}**H NMR** spectrum (400 MHz, $CDCl_3$) of compound **3ab**

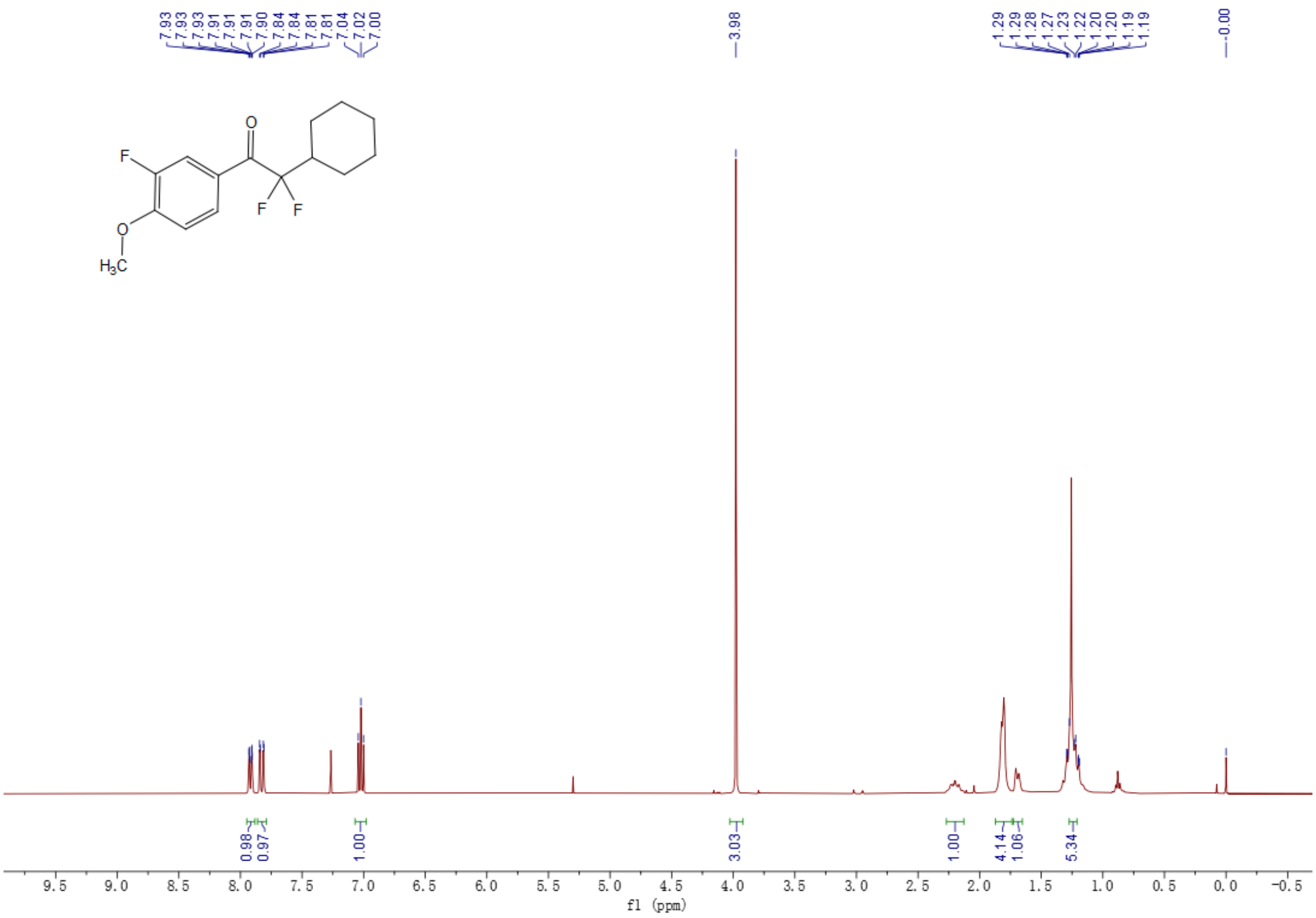

^{19}**F NMR** spectrum (376 MHz, $CDCl_3$) of compound **3ab**

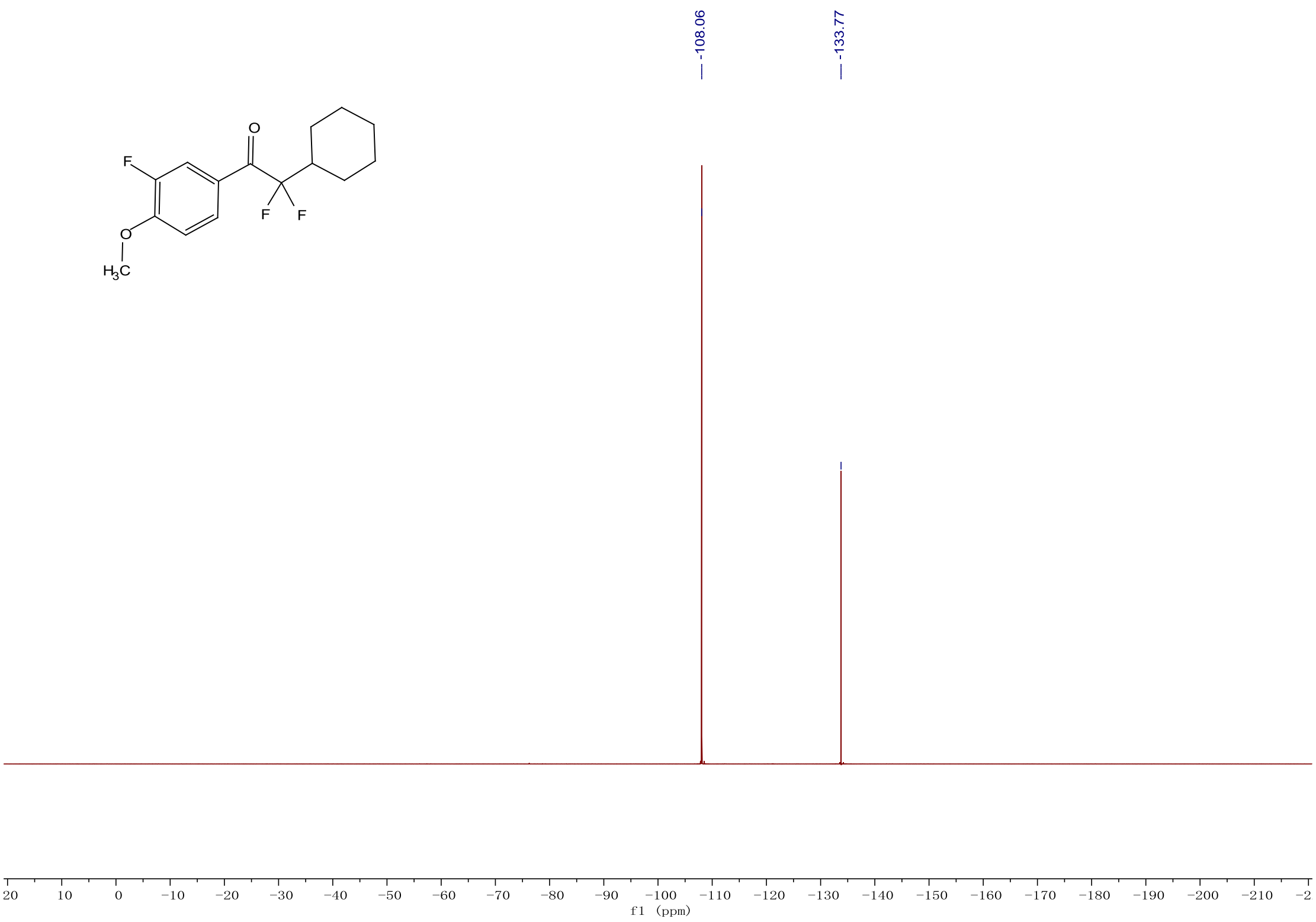

^{13}C NMR spectrum (101 MHz, $CDCl_3$) of compound **3ab**

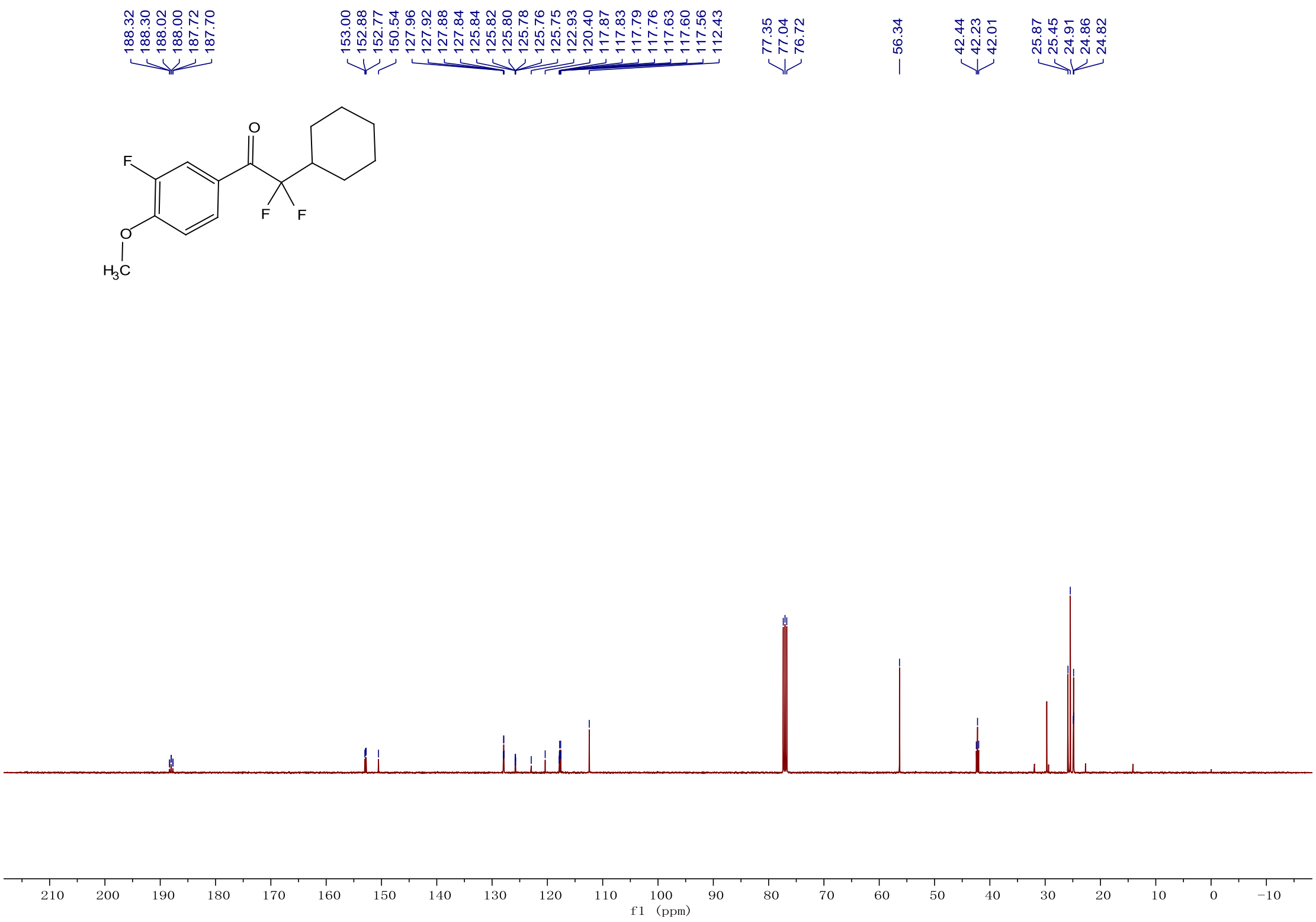

2-cyclohexyl-2,2-difluoro-1-(4-phenoxyphenyl)ethan-1-one (3ac)

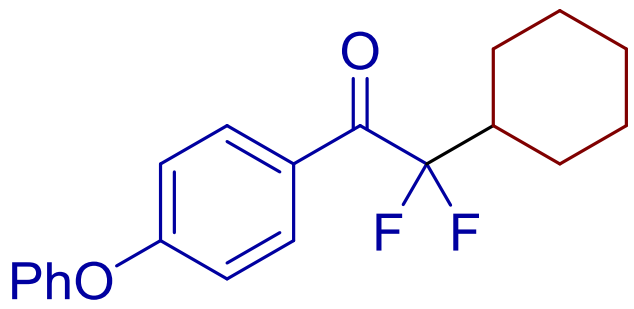

The product (51.5 mg, 78% yield) as colorless oil was purified with silica gel chromatography (petroleum ether/ ethyl acetate = 20/1). ^{1}**H NMR** (400 MHz, Chloroform-*d*) δ 8.07 (d, *J* = 9.2 Hz, 2H), 7.43-7.38 (m, 2H), 7.24-7.20 (m, 1H), 7.10-7.07 (m, 2H), 7.02-6.98 (m, 2H), 2.28-2.15 (m, 1H), 1.85-1.79 (m, 4H), 1.71-1.67 (m, 1H), 1.33-1.14 (m, 5H). ^{19}**F NMR** (376 MHz, Chloroform-*d*) δ -108.20 (s, 2F). ^{13}**C NMR** (101 MHz, Chloroform-*d*) δ 188.8 (t, *J* = 30.3 Hz), 163.0,155.0, 132.6 (t, *J* = 3.0 Hz), 130.2, 127.2 (t, *J* = 2.0 Hz), 125.0, 120.53, 120.47 (t, *J* = 249.5 Hz) ,117.1, 42.5 (t, *J* = 22.2 Hz), 25.9, 25.5, 24.9 (d, *J* = 5.0 Hz). **HRMS** (EI-TOF) m/z: $[M+Na]^+$ Calcd. for $C_{20}H_{20}F_2NaO_2$ 353.1324; Found 353.1333.

^{1}**H NMR** spectrum (400 MHz, $CDCl_3$) of compound **3ac**

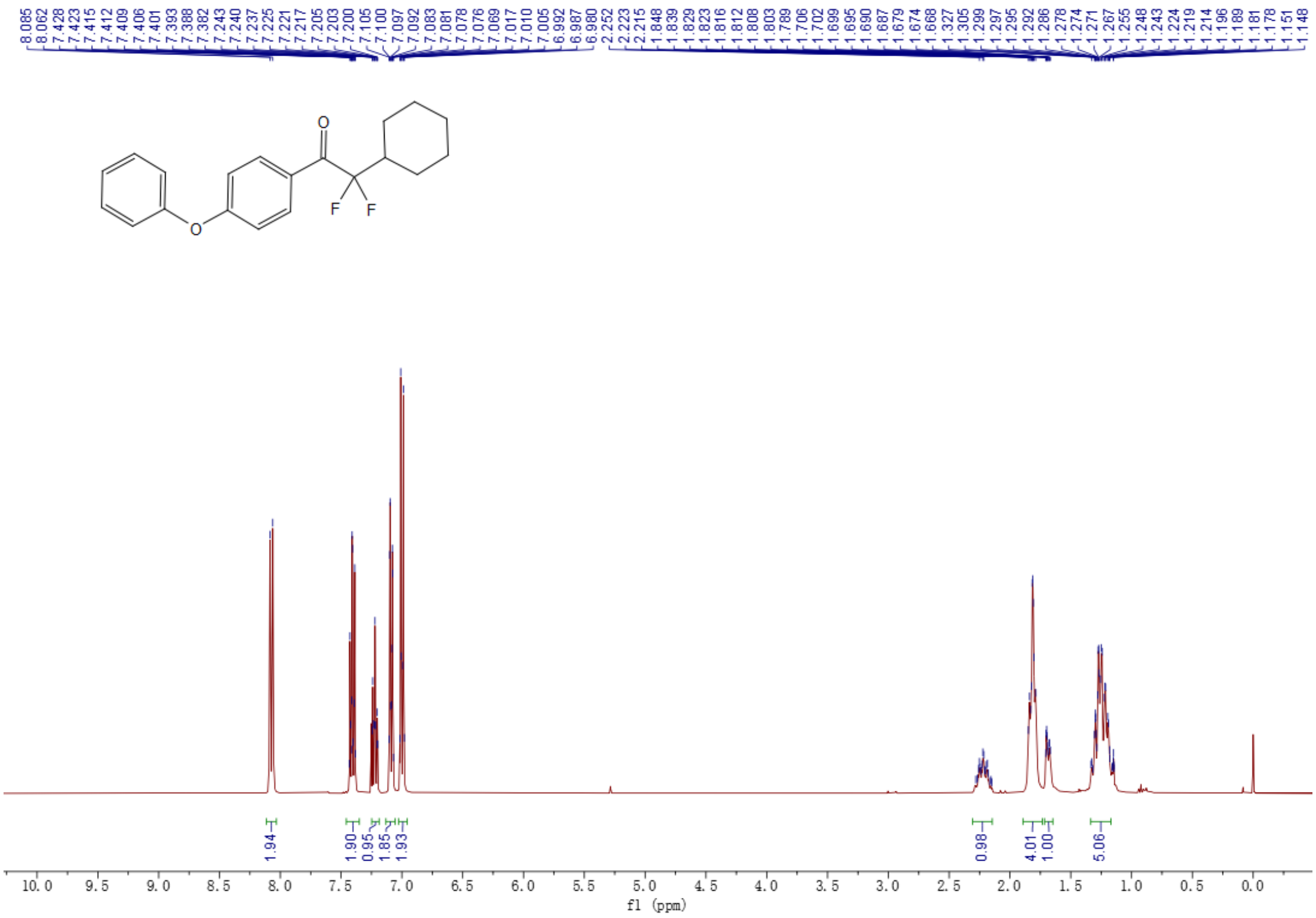

^{19}**F NMR** spectrum (376 MHz, $CDCl_3$) of compound **3ac**

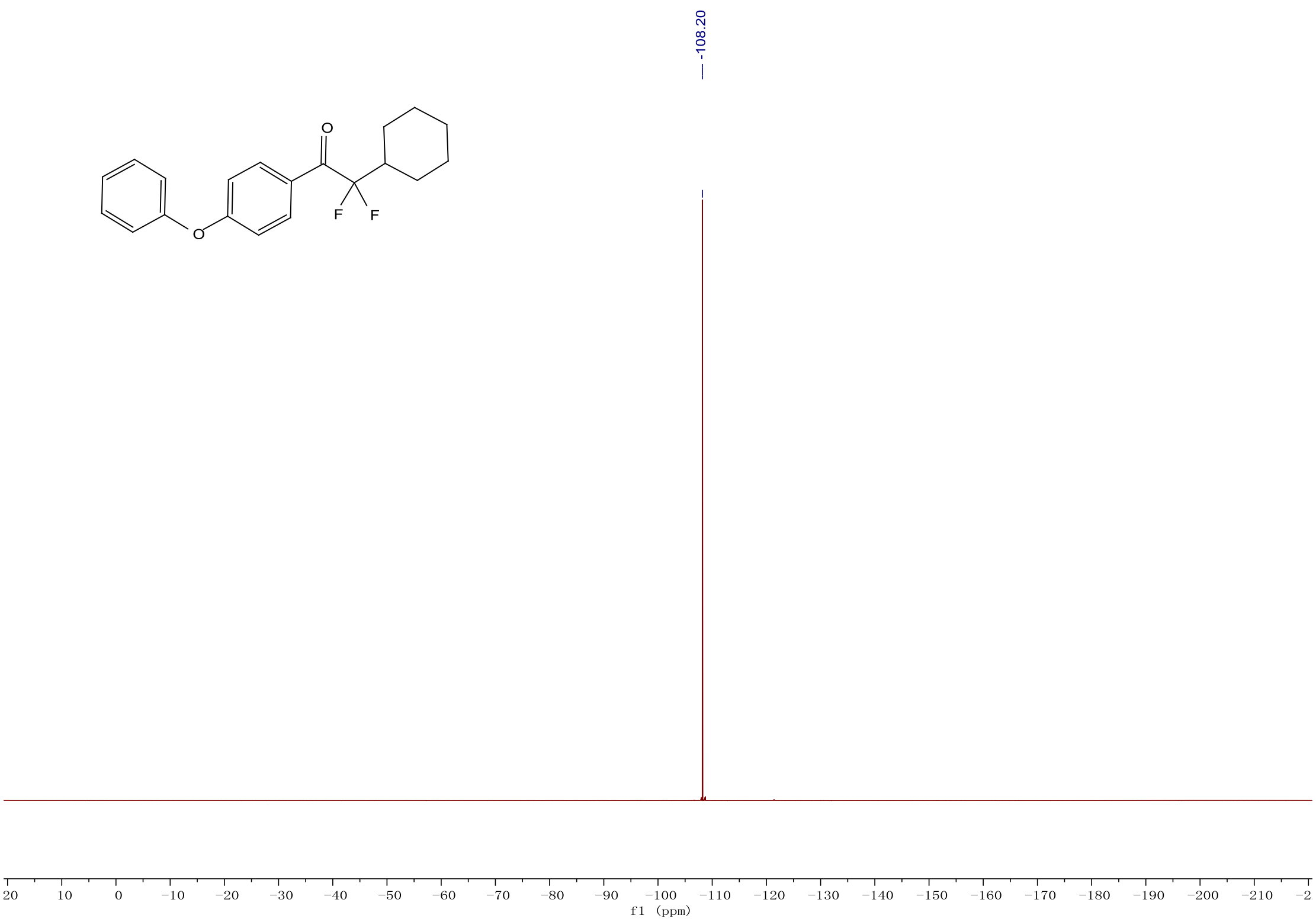

^{13}C NMR spectrum (101 MHz, $CDCl_3$) of compound **3ac**

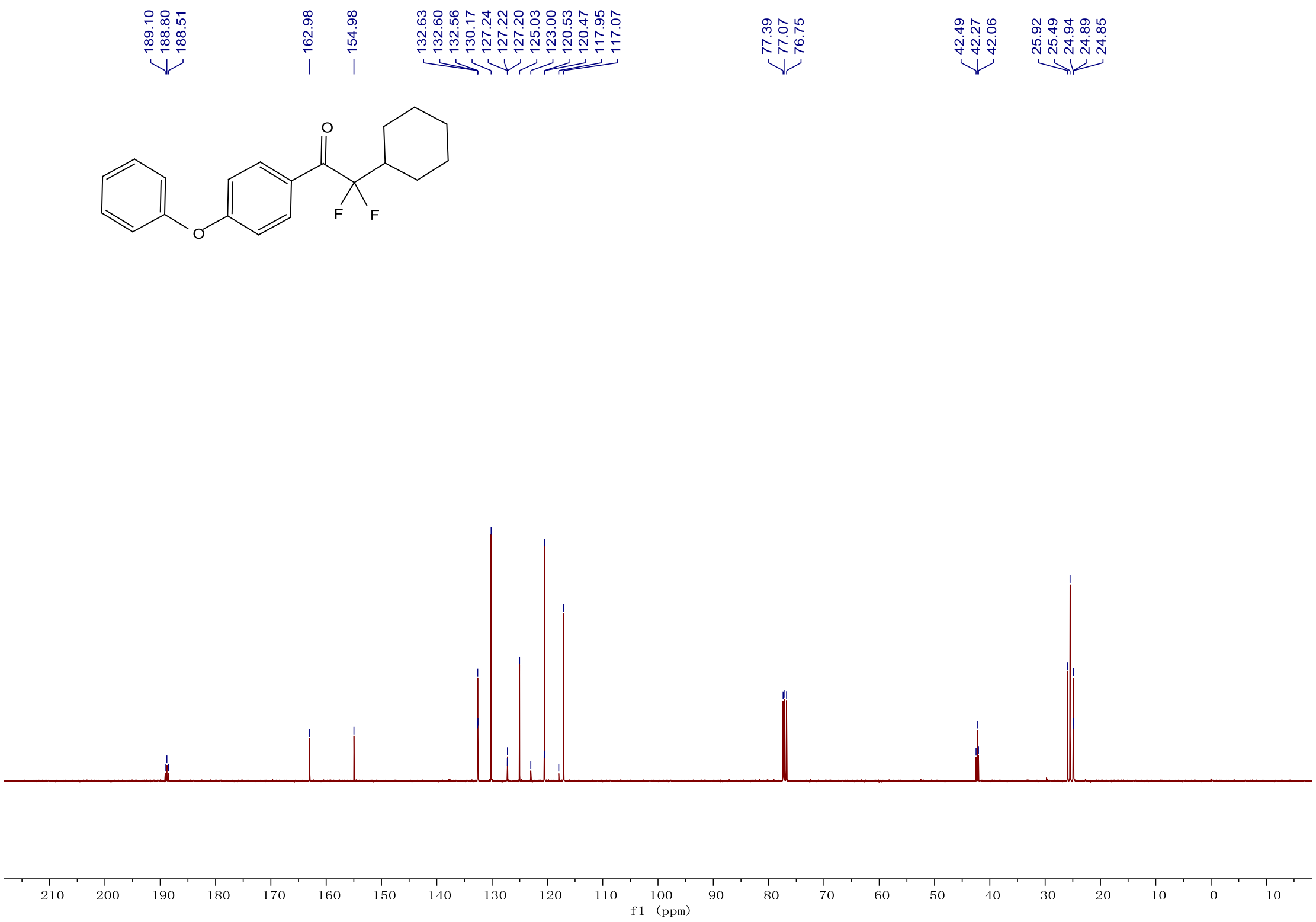

2-cyclohexyl-2,2-difluoro-1-(4-(trifluoromethoxy)phenyl)ethan-1-one (3ad)

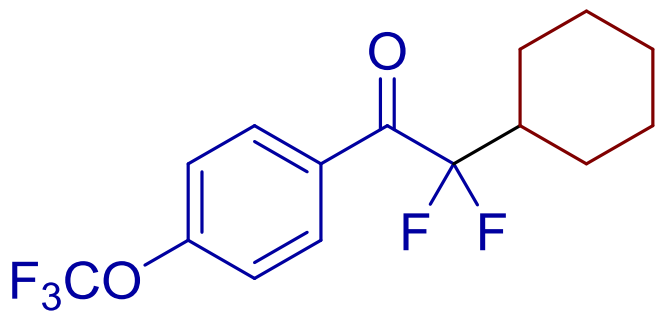

The product (40.6 mg, 63% yield) as colorless oil was purified with silica gel chromatography (petroleum ether/ ethyl acetate = 20/1). ^{1}**H NMR** (400 MHz, Chloroform-*d*) δ 8.15 (d, *J* = 8.8 Hz, 2H), 7.31 (d, *J* = 8.4 Hz, 2H) 2.27-2.17 (m, 1H), 1.84-1.81 (m, 4H), 1.71 (d, *J* = 10.8 Hz, 1H) 1.34-1.16 (m, 5H). ^{19}**F NMR** (376 MHz, Chloroform-*d*) δ -57.58 (s, 3F), -108.20 (s, 2F). ^{13}**C NMR** (101 MHz, Chloroform-*d*) δ 188.9 (t, *J* = 31.3 Hz), 153.3 (d, ,*J* = 1.0 Hz), 132.2 (t, *J* = 4.0 Hz), 131.0 (t, *J* = 2.0 Hz), 122.8 (t, *J* = 136.3 Hz), 120.2, 119.0 (t, *J* = 260.1 Hz), 41.9 (t, *J* = 21.2 Hz), 25.9, 25.4, 24.8 (t, *J* = 4.0 Hz). **HRMS** (EI-TOF) m/z: $[M+Na]^+$ Calcd. for $C_{15}H_{15}F_5NaO_2$ 345.0884; Found 345.0881.

^{1}**H NMR** spectrum (400 MHz, $CDCl_3$) of compound **3ad**

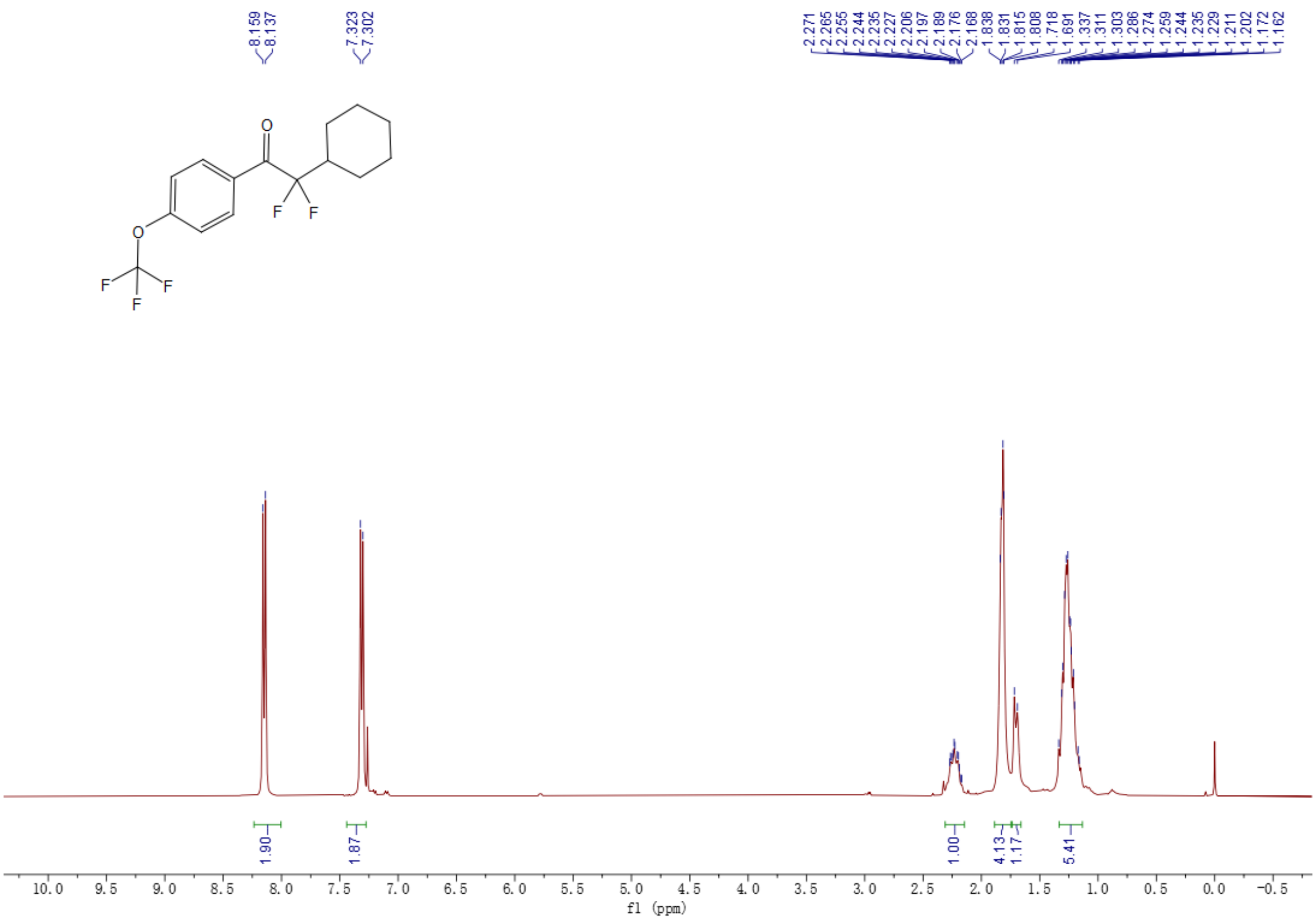

^{19}**F NMR** spectrum (376 MHz, $CDCl_3$) of compound **3ad**

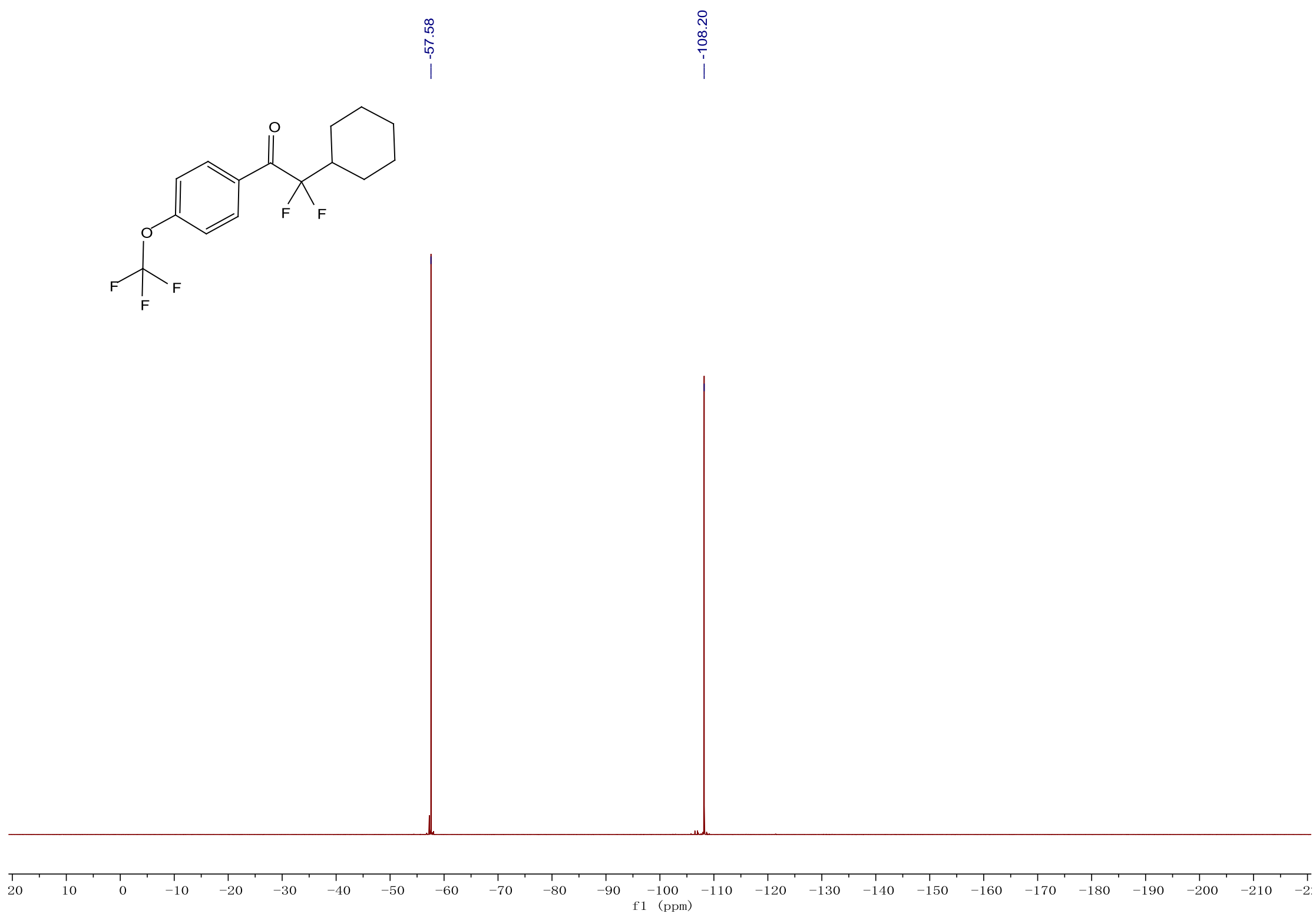

^{13}C NMR spectrum (101 MHz, $CDCl_3$) of compound **3ad**

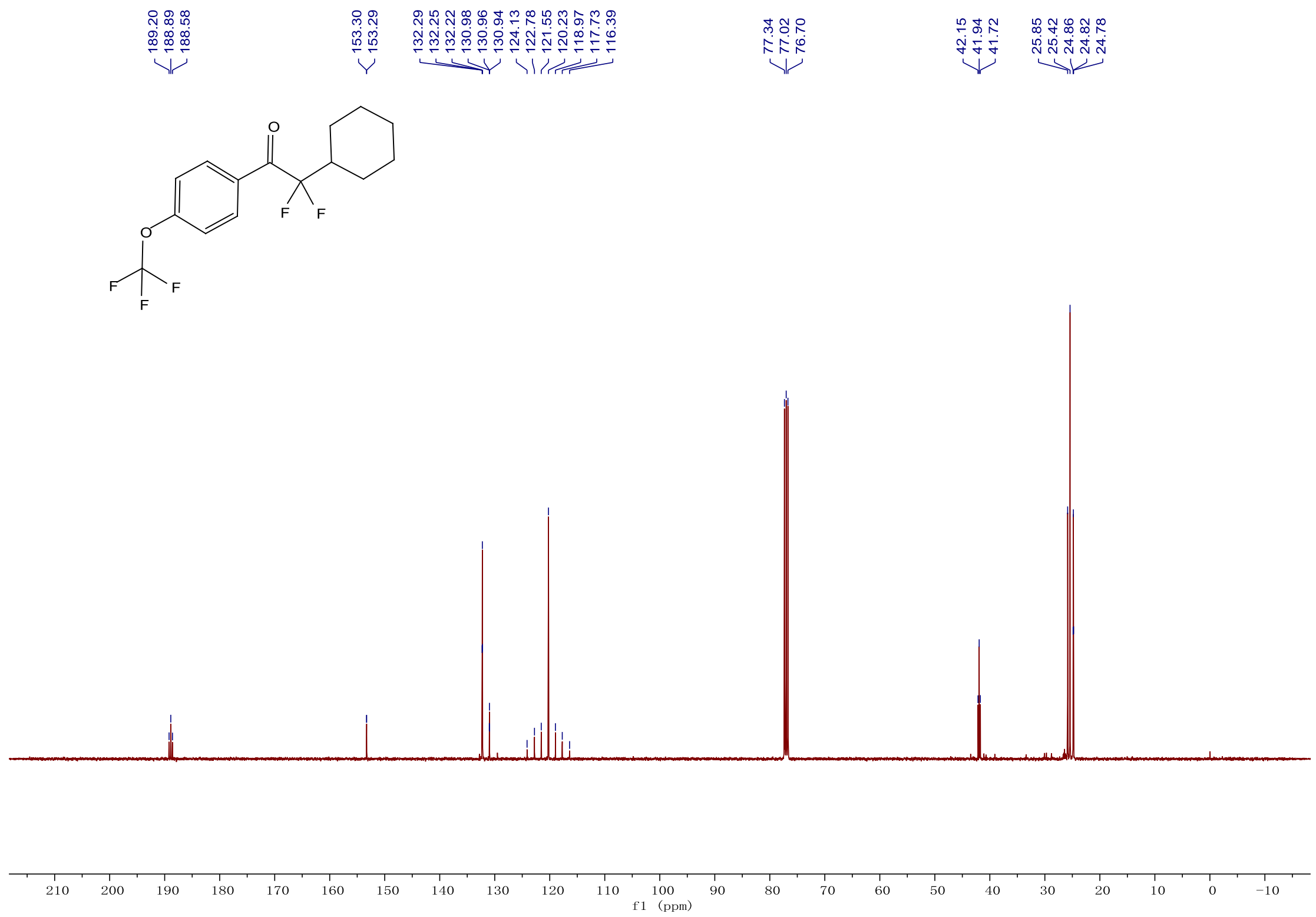

methyl (*S*)-2-((tert-butoxycarbonyl)amino)-3-(4-(2,2-difluorohexanoyl)phenyl) propanoate (3ae)

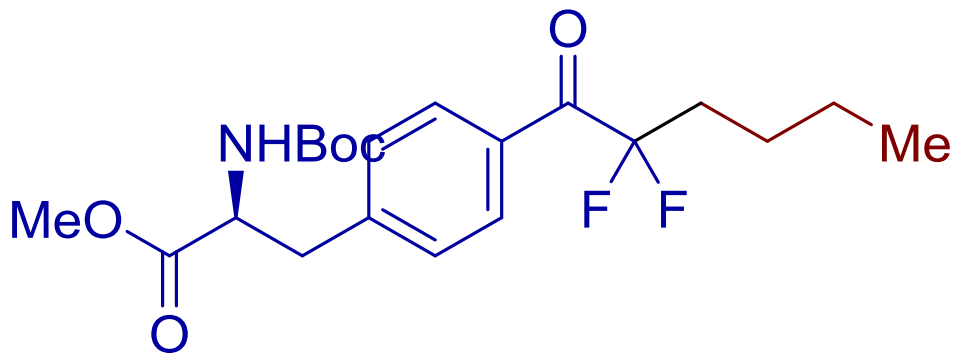

The product (48.0 mg, 58% yield) as white solid was purified with silica gel chromatography (petroleum ether/ ethyl acetate = 10/1). **^{1}H NMR** (400 MHz, Chloroform-*d*) δ 8.04 (d, *J* = 8.0 Hz, 2H), 7.27 (d, *J* = 8.4 Hz, 2H), 5.04 (d, *J* = 8.0 Hz, 1H), 4.64 (q, *J* = 6.4 Hz, 1H), 3.73 (s, 3H), 3.23 (dd, *J* = 13.6, J = 5.6 Hz, 1H), 3.10 (dd, *J* = 13.2, 6.0 Hz, 1H), 2.23 – 2.10 (m, 2H), 1.56 – 1.48 (m, 2H), 1.43 – 1.37 (m, 11H), 0.93 (t, *J* = 7.2 Hz, 3H). **^{19}F NMR** (376 MHz, Chloroform-*d*) δ -100.21 (s, 2F). **^{13}C NMR** (101 MHz, Chloroform-*d*) δ 189.2 (t, *J* = 31.3 Hz), 171.9, 154.9,143.2, 130.9, 130.4 (t, *J* = 3.0 Hz), 129.7, 119.9 (t, *J* = 253.5 Hz), 54.1, 52.4, 38.5,33.8 (t, *J* = 23.2 Hz), 28.3, 23.4 (t, J = 4.0 Hz), 22.5, 13.8. **HRMS** (EI-TOF) m/z: $[M+Na]^+$ Calcd. for $C_{21}H_{29}F_2NNaO_5$ 436.1906; Found 436.1914.

^{1}H NMR spectrum (400 MHz, $CDCl_3$) of compound **3ae**

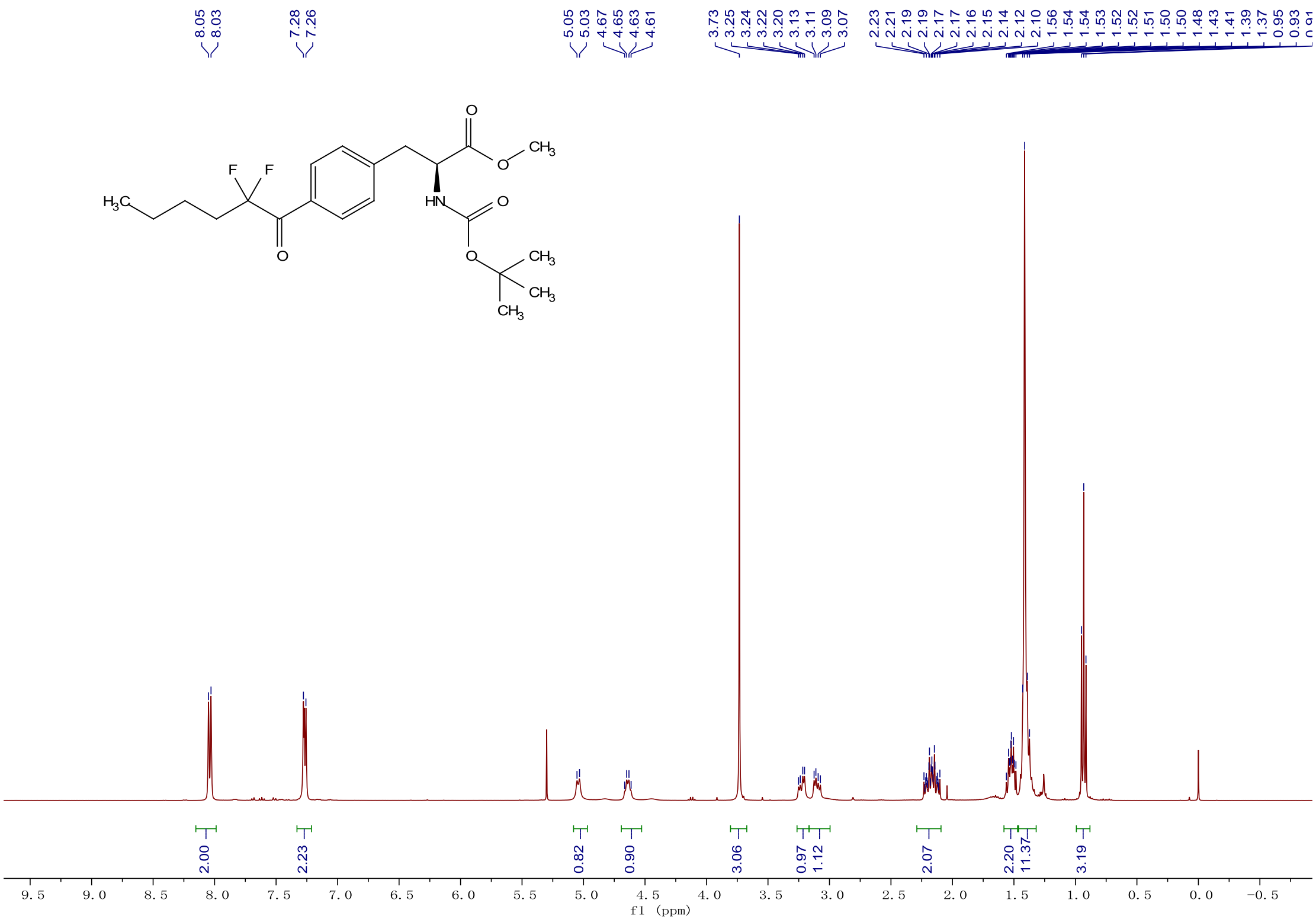

^{19}F NMR spectrum (376 MHz, $CDCl_3$) of compound **3ae**

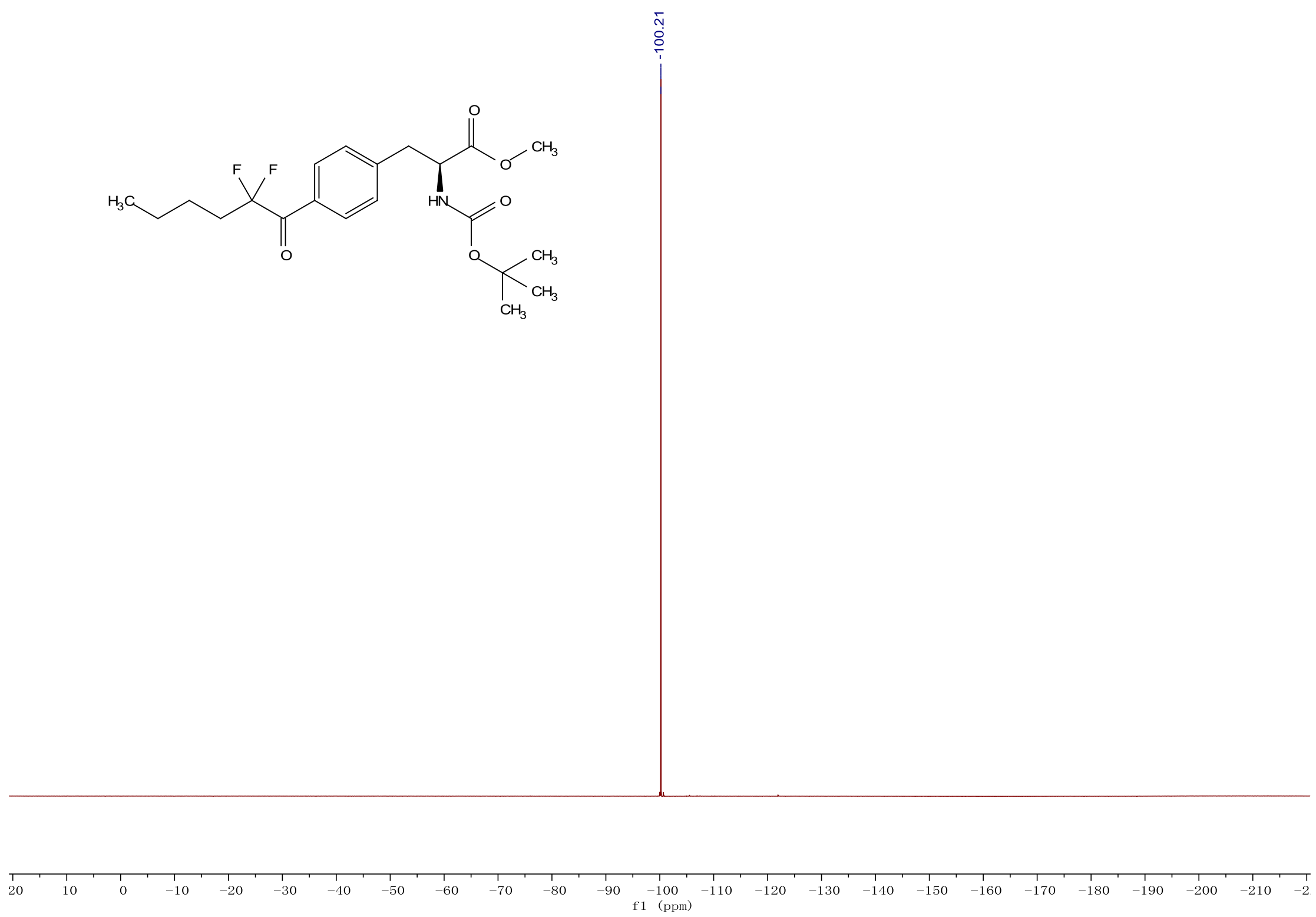

^{13}C NMR spectrum (101 MHz, $CDCl_3$) of compound **3ae**

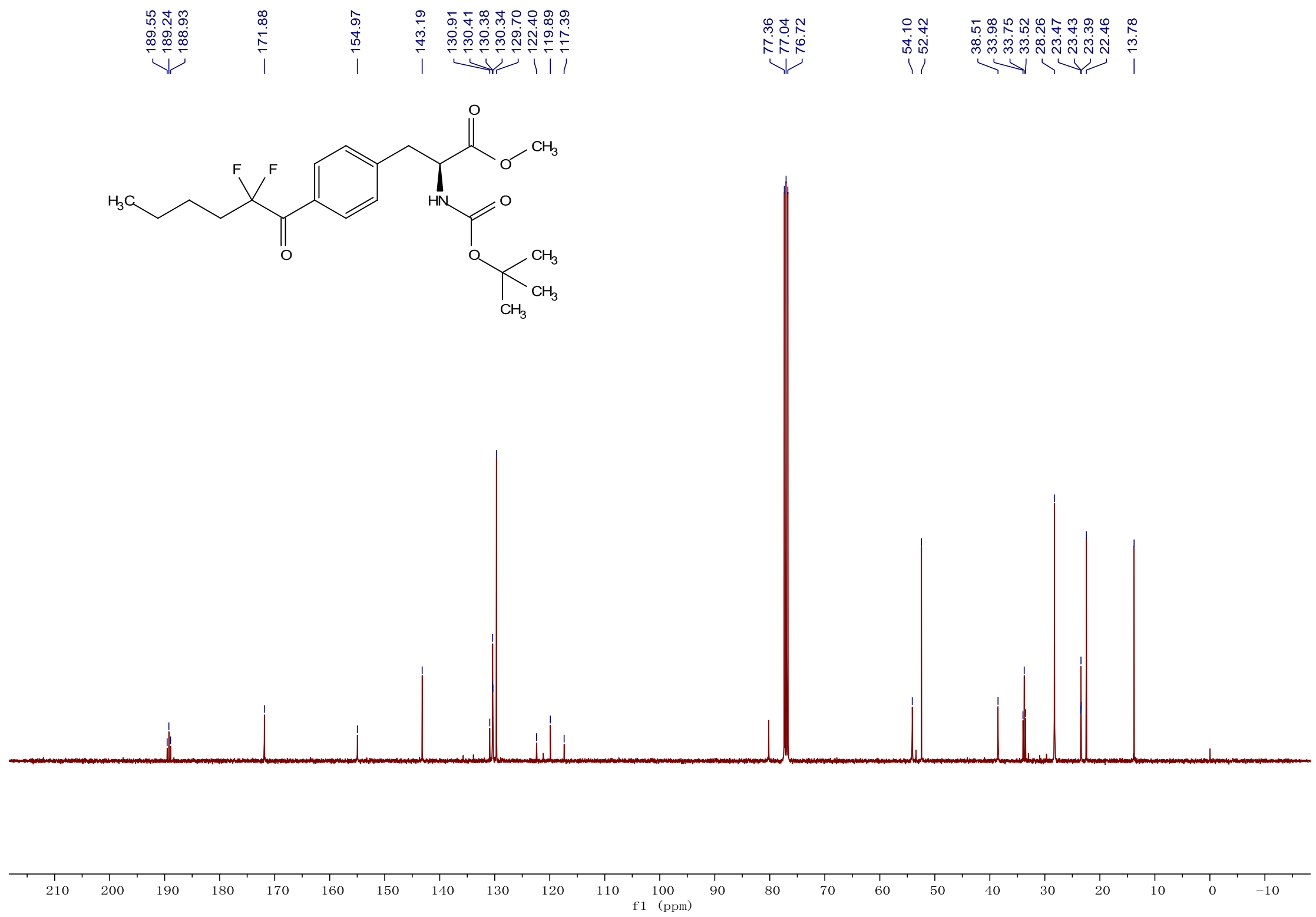

methyl (4-(6-(2,5-dimethylphenoxy)-2,2-difluoro-3,3-dimethylhexanoyl)benzoyl)-*D*-prolinate (3af)

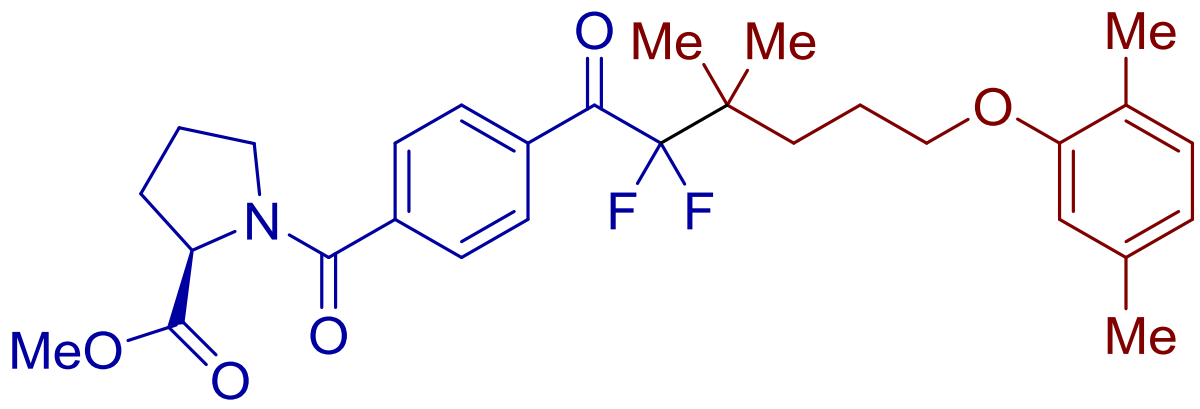

The product (54.6 mg, 53% yield) as light yellow oil was purified with silica gel chromatography (petroleum ether/ ethyl acetate = 5/1). **^{1}H NMR** (400 MHz, Chloroform-d, amide rotamers) δ 8.11 (d, *J* = 8.4 Hz, 1.6H), 8.07 (d, *J* = 8.0 Hz, 0.4H), 7.65 (d, *J* = 8.4 Hz, 1.6H), 7.46 (d, *J* = 8.4 Hz, 1.6H), 6.99 (d, *J* = 7.6 Hz, 1H), 6.65 (d, *J* = 7.2 Hz, 1H), 6.60 (s, 1H), 4.68 (dd, *J* = 8.4, *J* = 4.8 Hz, 0.8H), 4.28 (dd, *J* = 8.4, *J* = 2.8 Hz, 0.2H), 3.91 (t, *J* = 6.0 Hz, 2H), 3.79 (s, 2.4H), 3.63-3.59 (m, 1H), 3.56 (s, 0.6 H), 3.50-3.44 (m, 1H), 2.36-2.32 (m, 1H), 2.30 (s, 3H), 2.16 (s, 3H), 2.08-2.00 (m, 2H), 1.94-1.81 (m, 3H), 1.74-1.69 (m, 2H), 1.17 (s, 6H). **^{19}F NMR** (376 MHz, Chloroform-*d*) δ -107.20 (s, 2F). **^{13}C NMR** (101 MHz, Chloroform-*d*) δ 190.3 (t, *J* = 32.3 Hz), 172.5, 168.4, 156.9, 136.5, 135.6, 130.3, 130.2 (t, *J* = 5.1 Hz), 127.4, 126.7, 123.6, 122.0 (t, *J* = 259.6 Hz), 120.7, 111.9, 59.1, 52.4, 49.8, 40.5 (t, *J* = 21.2 Hz), 32.6, 29.4, 25.3, 24.0, 21.4, 21.1 (t, *J* = 4.0 Hz), 15.8. **HRMS** (EI-TOF) m/z: $[M+Na]^+$ Calcd. for $C_{21}H_{29}F_2NNaO_5$ 436.1906; Found 436.1914.

^{1}H NMR spectrum (400 MHz, $CDCl_3$) of compound **3af**

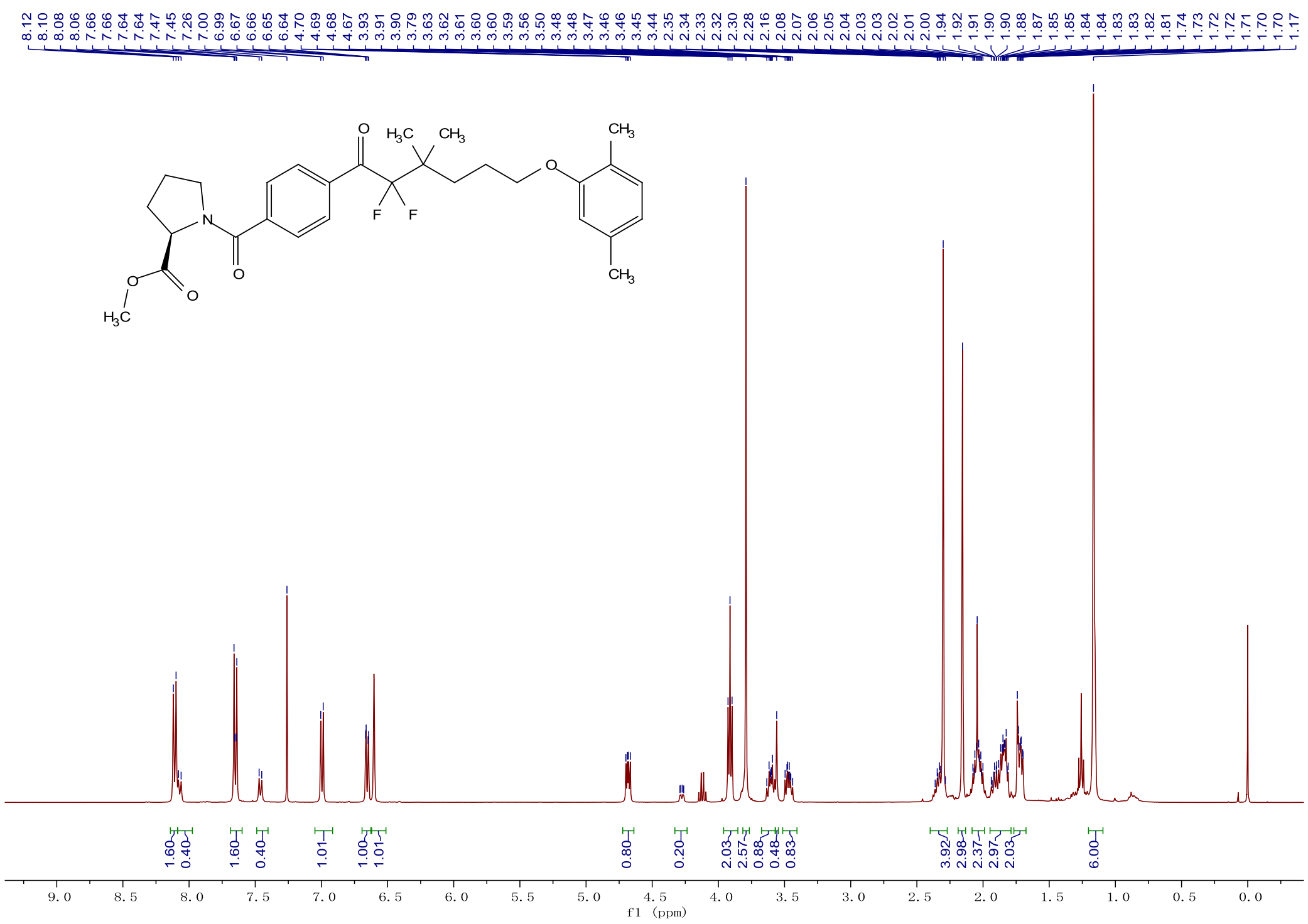

^{19}F NMR spectrum (376 MHz, $CDCl_3$) of compound **3af**

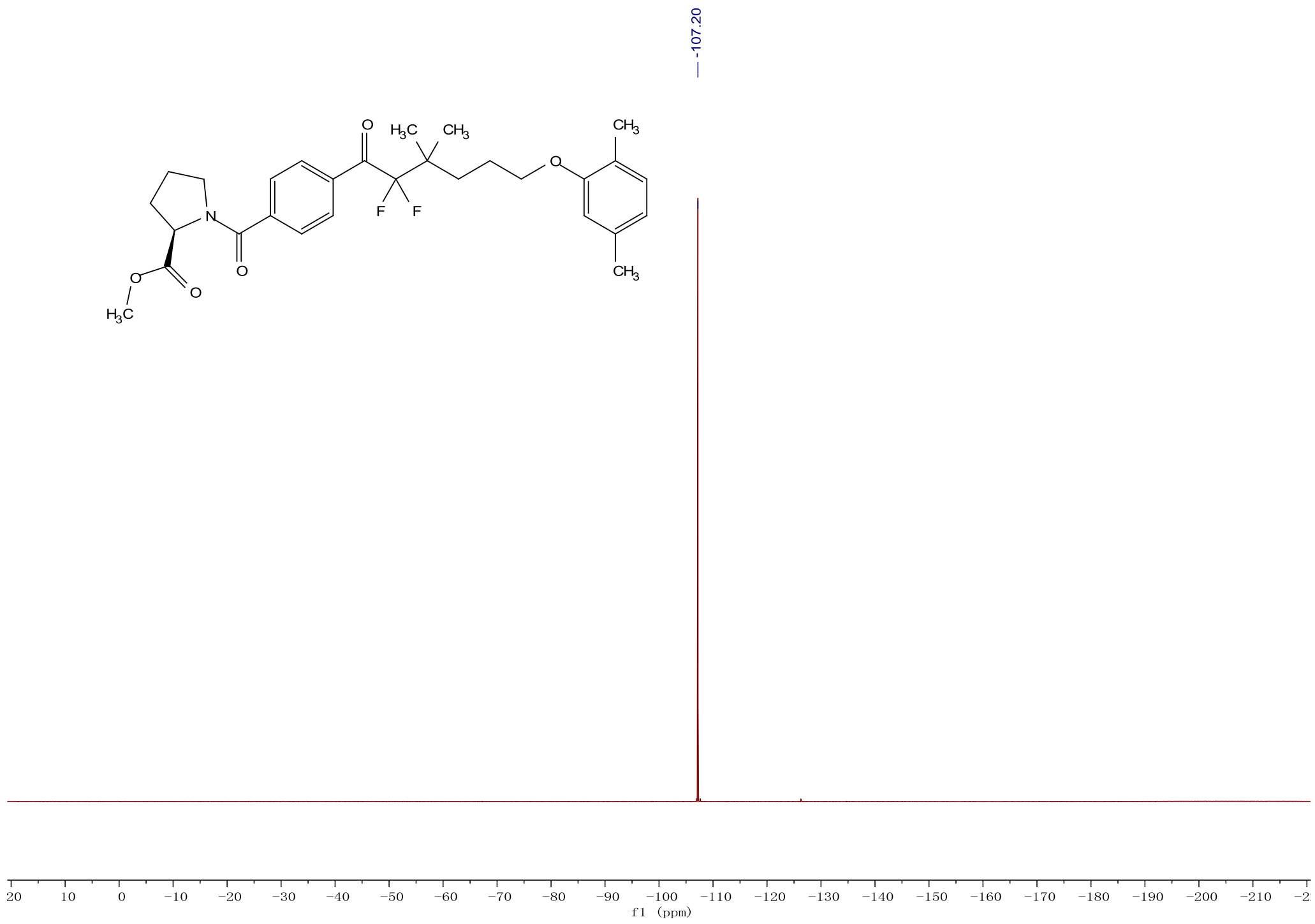

^{13}C NMR spectrum (101 MHz, $CDCl_3$) of compound **3af**

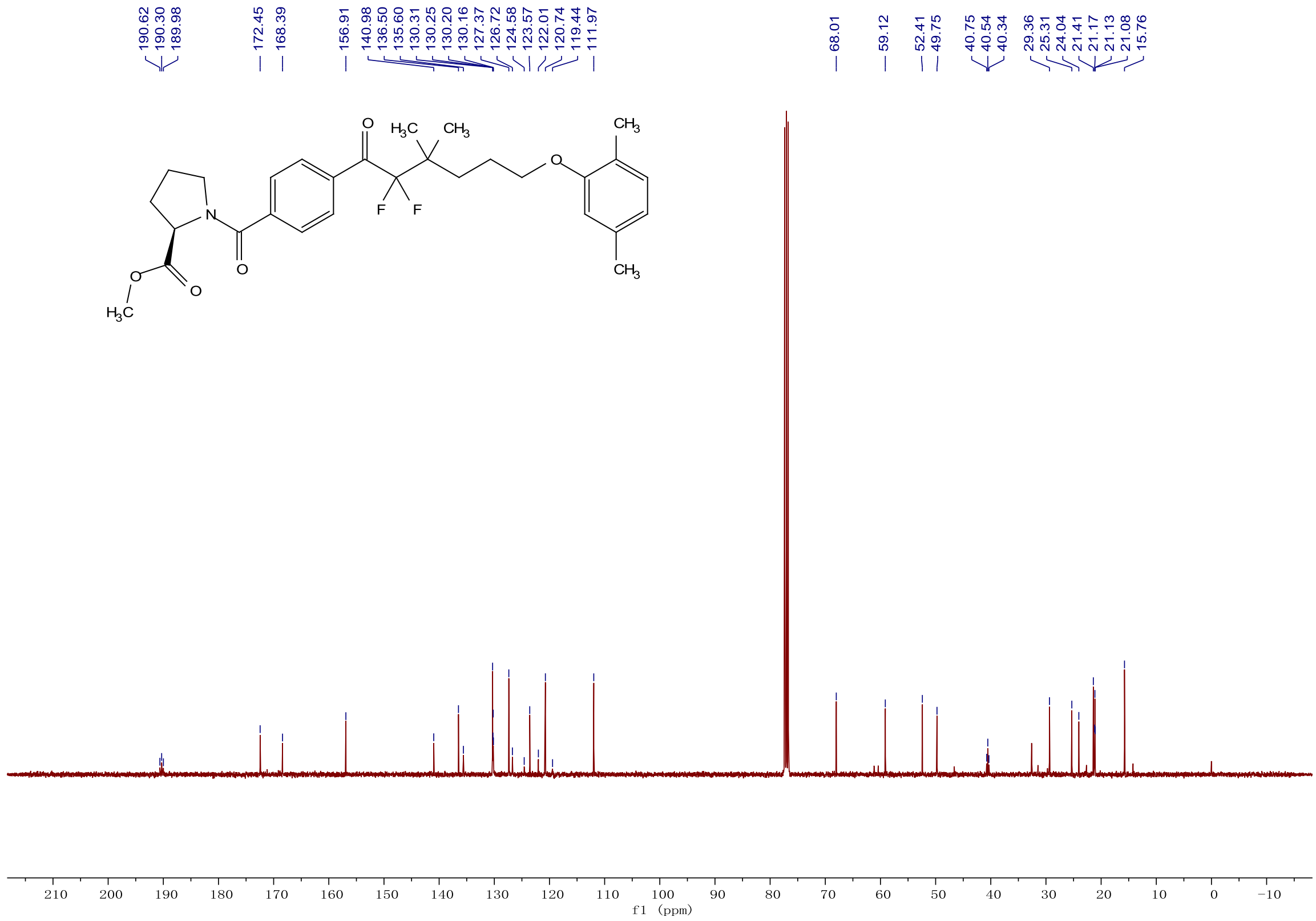

((3a*S*,5a*R*,8a*R*,8b*S*)-2,2,7,7-tetramethyltetrahydro-3a*H*-bis([1,3]dioxolo)[4,5-*b*:4',5'-*d*]pyran-3a-yl)methyl 4-(6-(2,5-dimethylphenoxy)-2,2-difluoro-3,3-dimethyl-hexanoyl)benzoate (3ag)

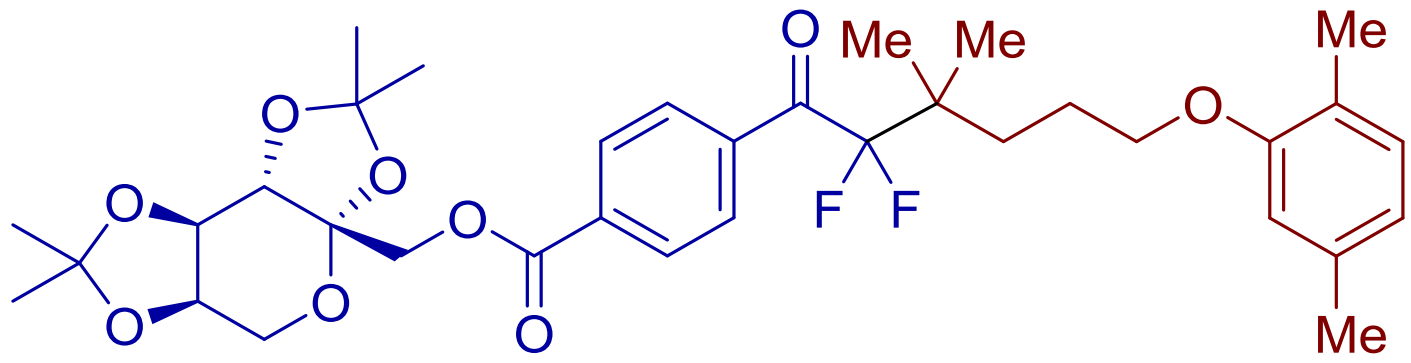

The product (72.4 mg, 56% yield) as colorless oil was purified with silica gel chromatography (petroleum ether/ ethyl acetate = 10/1). **^{1}H NMR** (400 MHz, Chloroform-d, amide rotamers) δ 8.16 (d, *J* = 8.4 Hz, 2H), 8.11 (d, *J* = 8.4 Hz, 2H), 6.99 (d, *J* = 7.2 Hz, 1H), 6.65 (d, *J* = 7.6 Hz, 1H), 6.60 (s, 1H), 4.71 (d, *J* = 12.0 Hz, 1H), 4.65 (dd, *J* = 8.0, *J* = 2.4 Hz, 1H), 4.44 (d, *J* = 2.4 Hz, 1H), 4.35 (d, *J* = 12.0 Hz, 1H), 4.26 (dd, *J* = 8.0, *J* = 1.2 Hz, 1H), 3.96 (dd, *J* = 12.8, *J* = 1.6 Hz, 1H), 3.91 (t, *J* = 6.0 Hz, 2H), 3.81 (d, *J* = 12.8 Hz, 1H), 2.30 (s, 3H), 2.15 (s, 3H), 1.88-1.81 (m, 2H), 1.75-1.70 (m, 2H), 1.55 (s, 3H), 1.46 (s, 3H), 1.36 (s, 3H), 1.35 (s, 3H), 1.17 (s, 6H). **^{19}F NMR** (376 MHz, Chloroform-*d*) δ -107.20 (s, 2F). **^{13}C NMR** (101 MHz, Chloroform-*d*) δ 190.4 (t, *J* = 32.3 Hz), 164.9, 156.9, 137.9, 136.5, 134.1, 130.3, 130.0 (t, *J* = 4.0 Hz), 129.8, 123.6, 121.9 (t, *J* = 259.6 Hz), 120.8, 111.9, 109.2, 108.9, 101.5, 70.8, 70.7, 70.1, 68.0, 65.9, 61.4, 40.6 (t, *J* = 20.2 Hz), 32.6 (t, *J* = 3.0 Hz), 26.5, 25.9, 25.5, 24.0, 21.4, 21.1 (t, *J* = 4.0 Hz), 21.0, 15.8. **HRMS** (EI-TOF) m/z: $[M+Na]^+$ Calcd. for $C_{35}H_{44}F_2O_9$ 669.2846; Found 669.2850.

^{1}H NMR spectrum (400 MHz, $CDCl_3$) of compound **3ag**

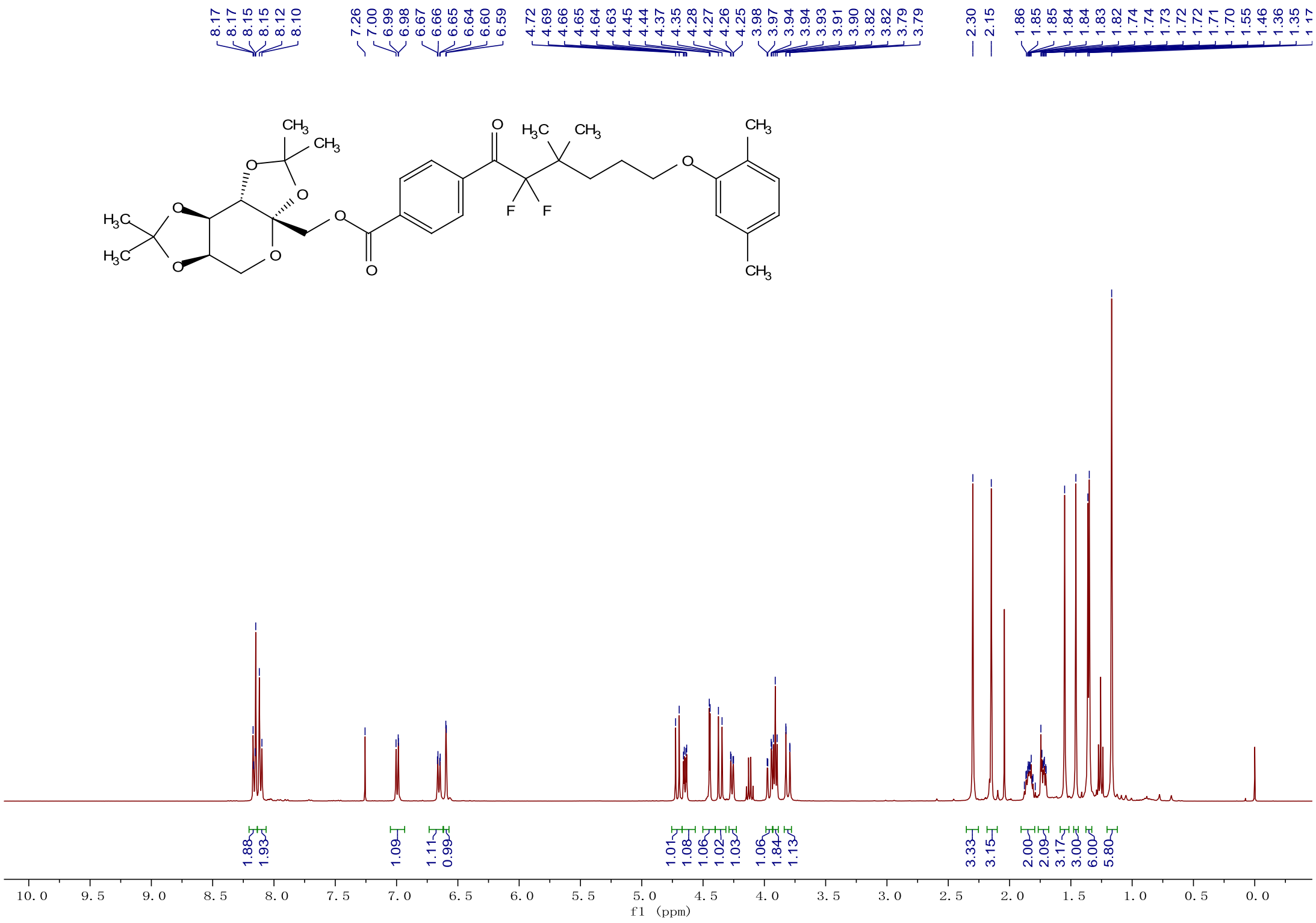

^{19}F NMR spectrum (376 MHz, $CDCl_3$) of compound **3ag**

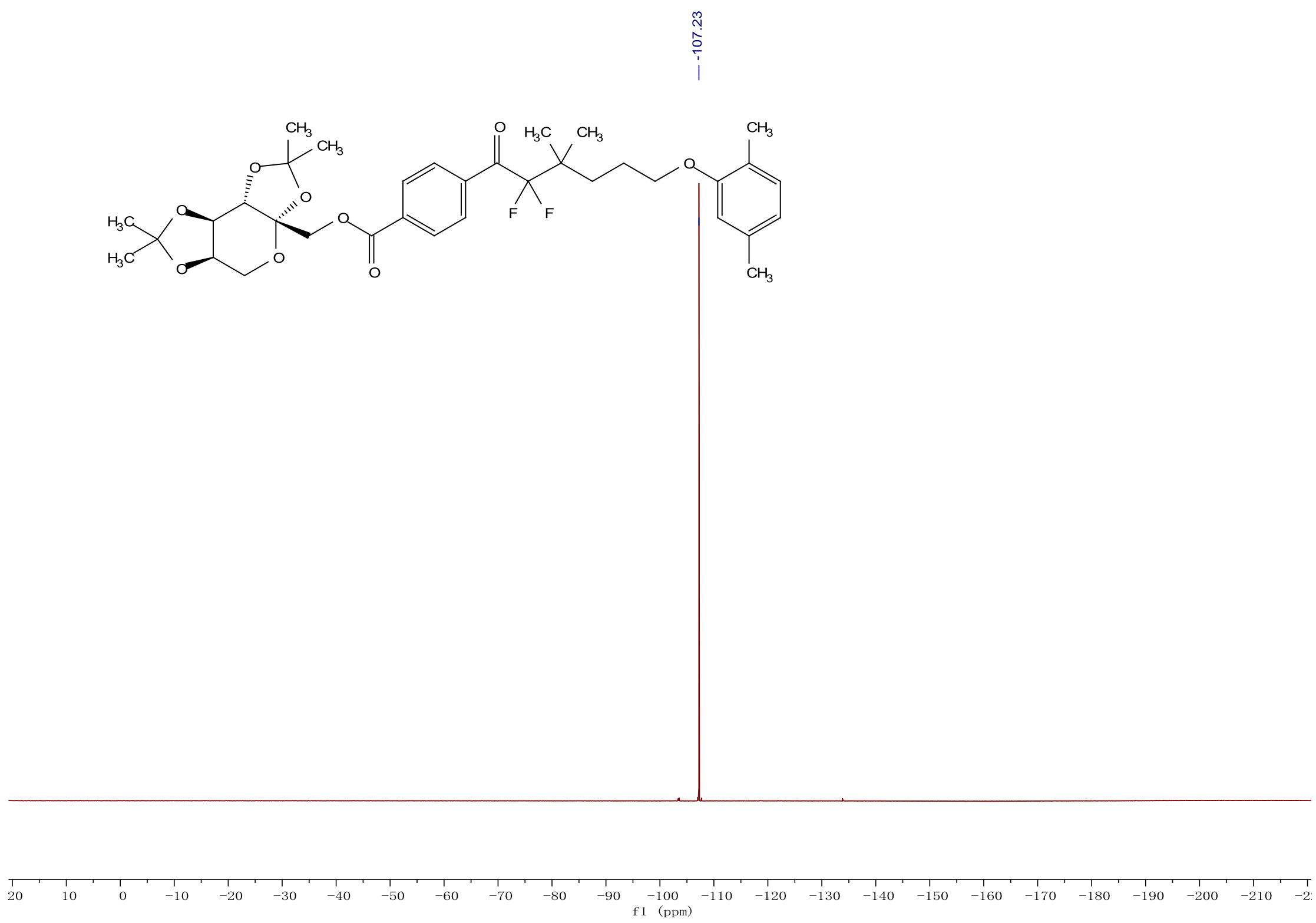

^{13}C NMR spectrum (101 MHz, $CDCl_3$) of compound **3ag**

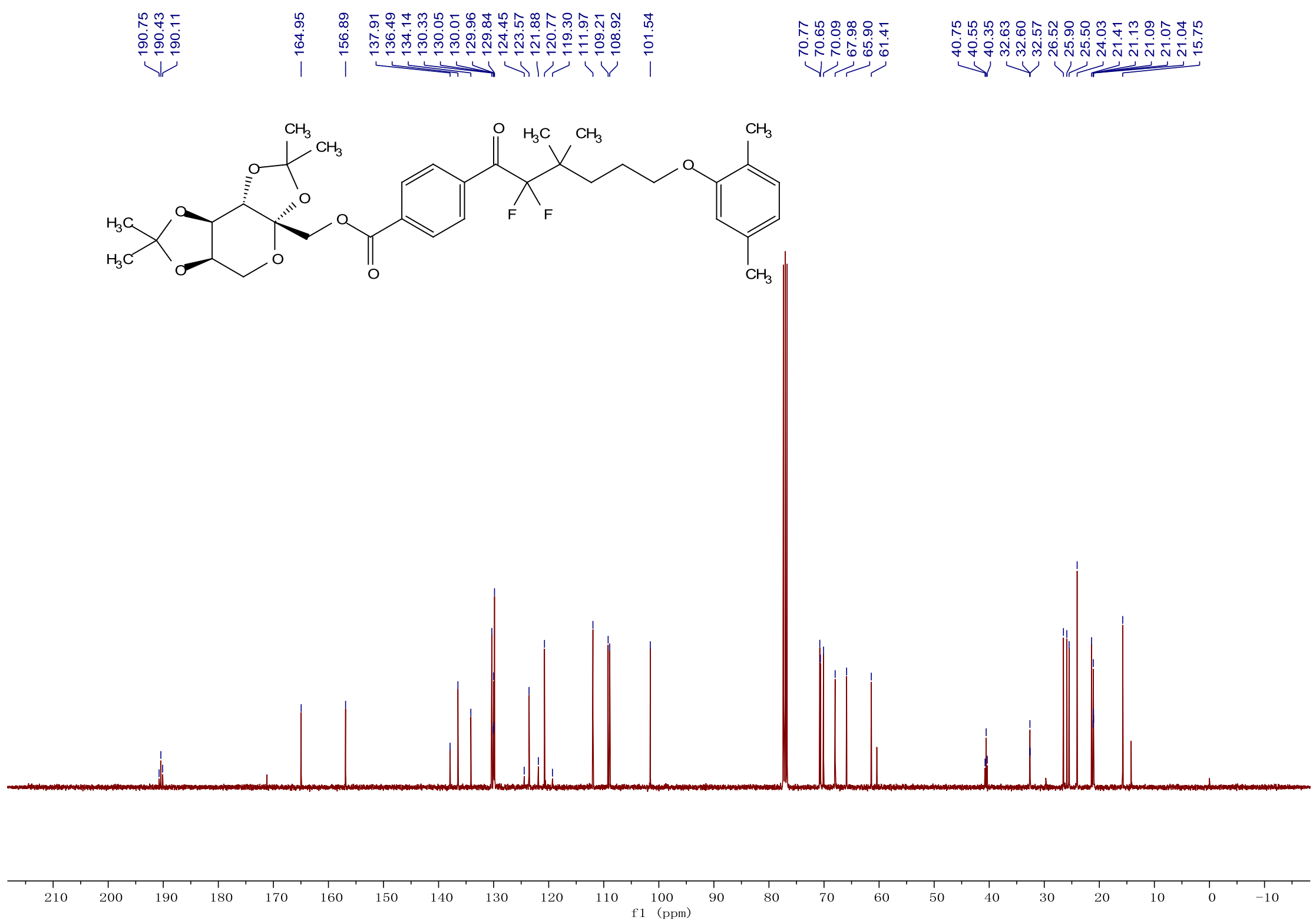

6-(2,5-dimethylphenoxy)-2,2-difluoro-3,3-dimethyl-1-phenylhexan-1-ol (4a)

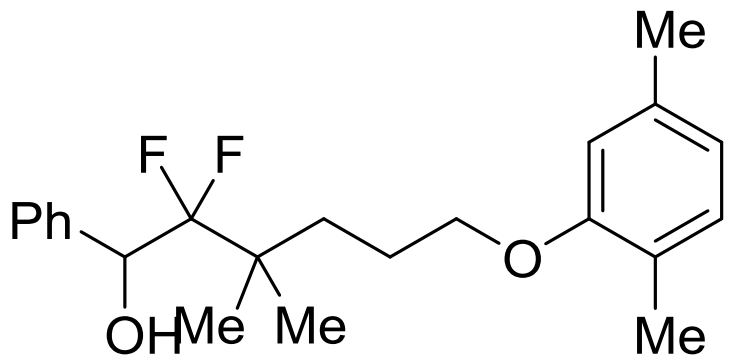

The product (60.2 mg, 83% yield) as white solid was purified with silica gel chromatography (petroleum ether/ ethyl acetate = 50/1). **^{1}H NMR** (400 MHz, Chloroform-*d*) δ 7.51-7.50 (m, 2H), 7.44-7.39 (m, 3H), 7.07 (d, *J* = 7.6 Hz, 1H), 6.73 (d, *J* = 7.6 Hz, 1H), 6.68 (s, 1H), 5.08-5.01 (m, 1H), 3.97 (t, *J* = 6.0 Hz, 2H), 2.40 (brs, 1H), 2.38 (s, 3H), 2.26 (s, 3H), 1.95-1.84 (m, 2H), 1.82-1.74 (m, 2H), 1.19 (s, 3H), 1.14 (s, 3H). **^{19}F NMR** (376 MHz, Chloroform-*d*) δ -110.5 (d, *J* = 259.4 Hz), -119.4 (d, *J* = 255.7 Hz). **^{13}C NMR** (101 MHz, Chloroform-*d*) δ 157.0, 138.1, 136.5, 130.4, 128.8, 128.4, 128.2, 124.5 (dd, *J* = 256.5 Hz, *J* = 251.5 Hz), 123.6, 120.7, 112.0, 74.2 (dd, *J* = 33.3 Hz, *J* = 25.2 Hz), 68.3, 41.0 (t, *J* = 22.2 Hz), 33.8, 24.3, 22.0 (t, *J* = 4.0 Hz), 21.9 (t, *J* = 4.0 Hz), 21.4, 15.8. **HRMS** (EI-TOF) m/z: $[M+Na]^+$ Calcd. for $C_{22}H_{28}F_2O_2Na$ 385.1950; Found 385.1950.

^{1}H NMR spectrum (400 MHz, $CDCl_3$) of compound **4a**

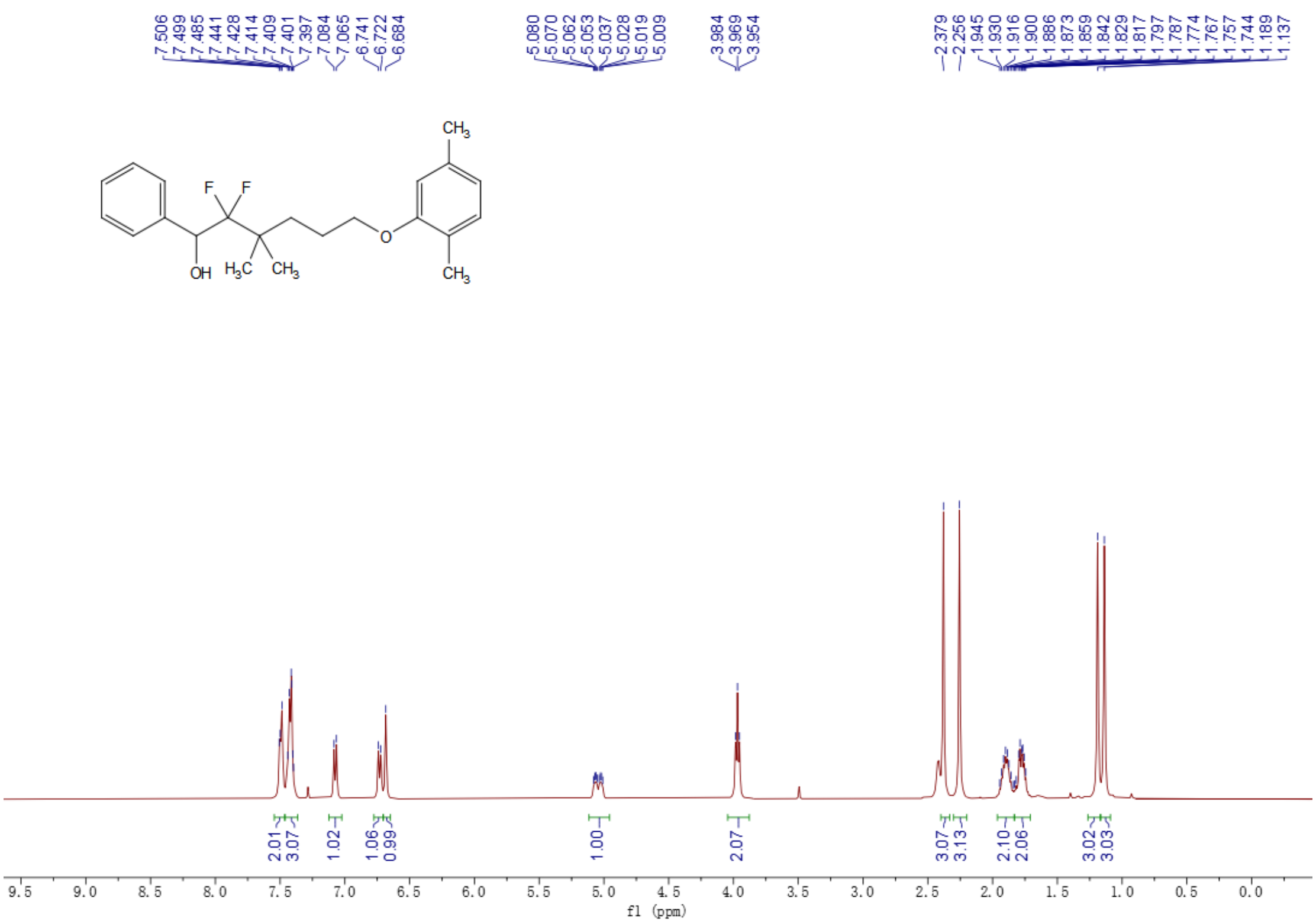

^{19}F NMR spectrum (376 MHz, $CDCl_3$) of compound **4a**

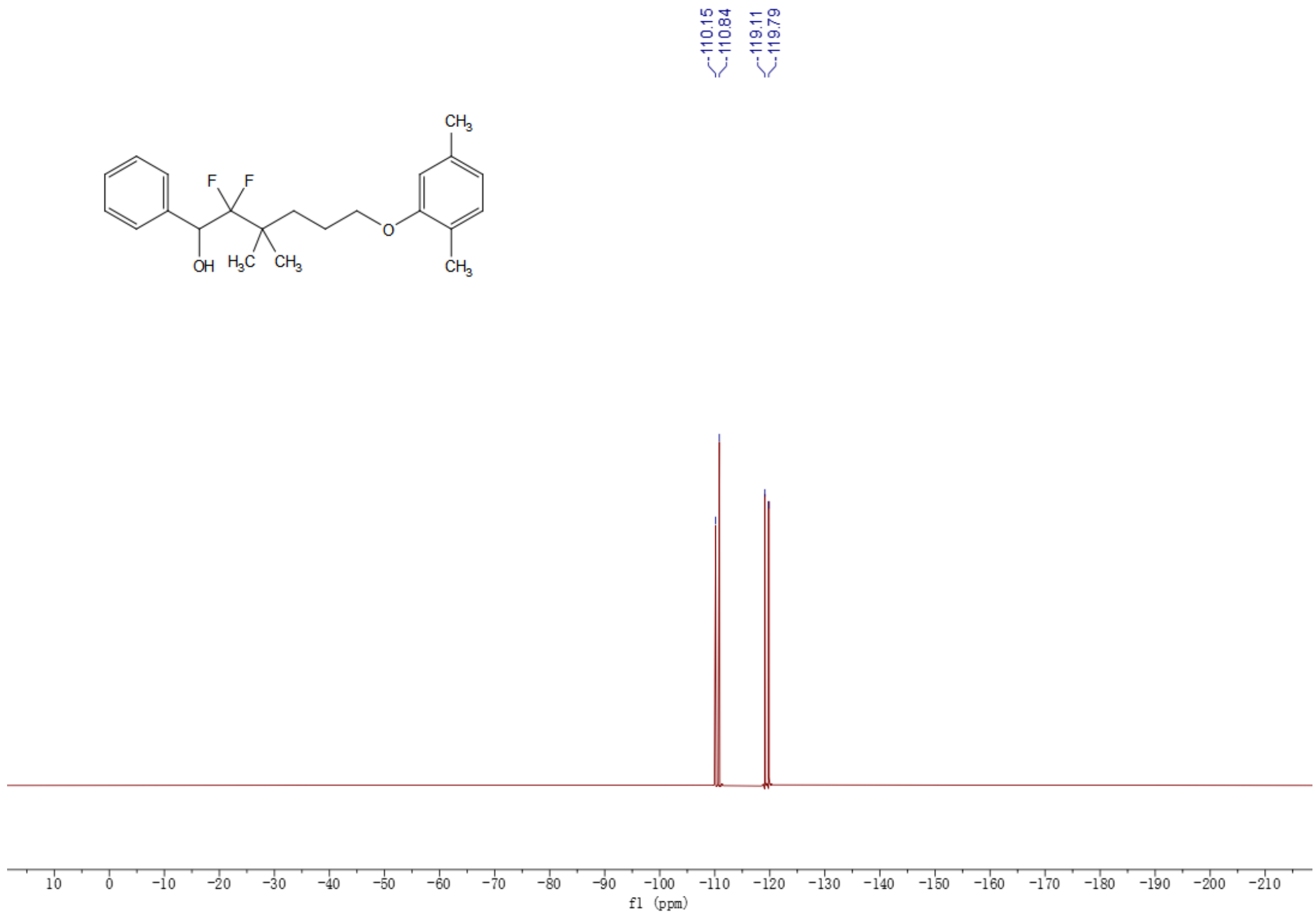

^{13}**C NMR** spectrum (101 MHz, $CDCl_3$) of compound **4a**

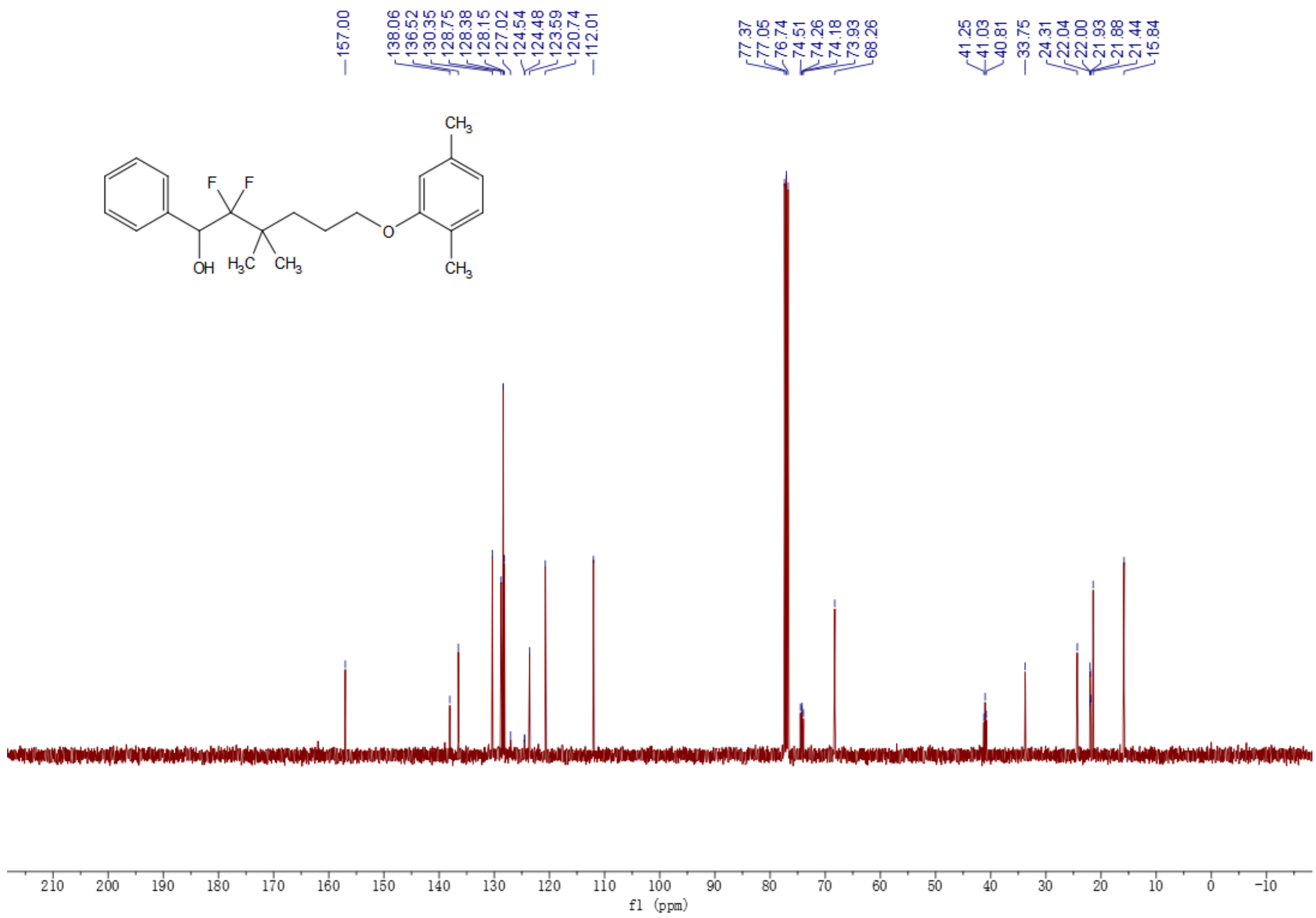

8-(2,5-dimethylphenoxy)-4,4-difluoro-5,5-dimethyl-3-phenyloct-1-en-3-ol (4b)

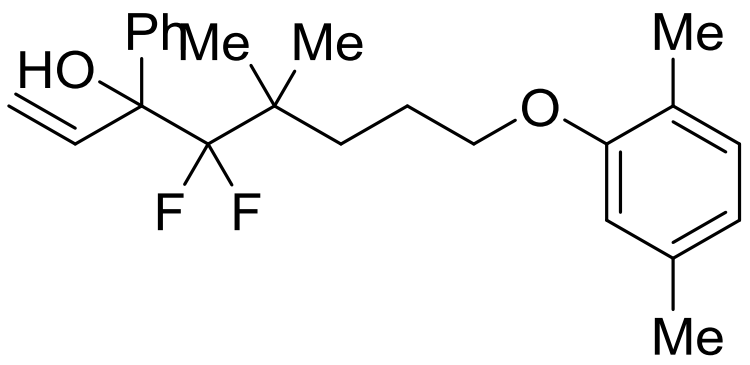

The product (55.9 mg, 72% yield) as colorless oil was purified with silica gel chromatography (petroleum ether/ ethyl acetate = 50/1). **^{1}H NMR** (400 MHz, Chloroform-*d*) δ 7.53 (d, *J* = 7.6 Hz, 2H), 7.26-7.15 (m, 3H), 6.92 (d, *J* = 7.6 Hz, 1H), 6.65 (dd, *J* = 17.2 Hz, *J* = 10.8 Hz, 1H), 6.57 (d, *J* = 7.6 Hz, 1H), 6.50 (s, 1H), 5.27 (d, *J* = 17.2 Hz, 1H), 5.18 (d, *J* = 10.8 Hz, 1H), 3.72 (t, *J* = 6.4 Hz, 2H), 2.38 (s, 1H), 2.23 (s, 3H), 2.09 (s, 3H), 1.67-1.59 (m, 2H), 1.51-1.46 (m, 2H), 0.84 (s, 3H), 0.75(s, 3H). **^{19}F NMR** (376 MHz, Chloroform-*d*) δ -108.6 (d, *J* = 263.2 Hz), -109.6 (d, *J* = 266.9 Hz). **^{13}C NMR** (101 MHz, Chloroform-*d*) δ 157.1, 140.7 (dd, *J* = 5.1 Hz, *J* = 3.0 Hz), 140.3 (d, *J* = 3.0 Hz), 136.5, 130.3, 128.0, 127.7, 126.5, 125.3 (dd, *J* = 261.6 Hz, *J* = 258.6 Hz), 123.6, 120.6, 114.4, 112.0, 79.9 (t, *J* = 28.3 Hz), 68.4, 42.9 (t, *J* = 22.2 Hz), 34.3 (t, *J* = 4.0 Hz), 24.4, 23.2 (t, *J* = 5.1 Hz), 22.8 (t, *J* = 5.1 Hz), 21.5, 15.9. **HRMS** (EI-TOF) m/z: $[M+Na]^+$ Calcd. for $C_{24}H30F_2O_2Na$ 411.2106; Found 411.2108.

^{1}H NMR spectrum (400 MHz, $CDCl_3$) of compound **4b**

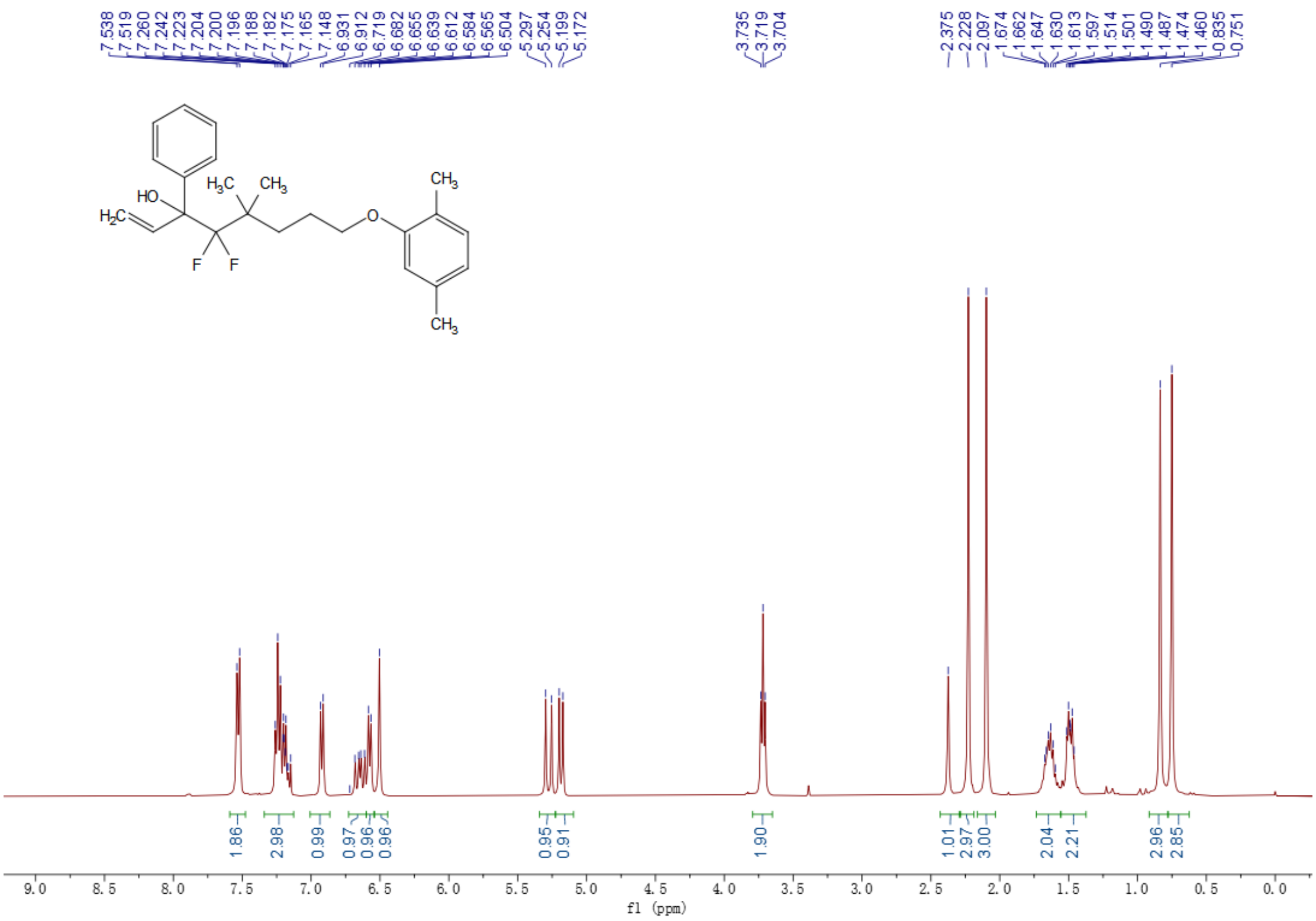

^{19}F NMR spectrum (376 MHz, $CDCl_3$) of compound **4b**

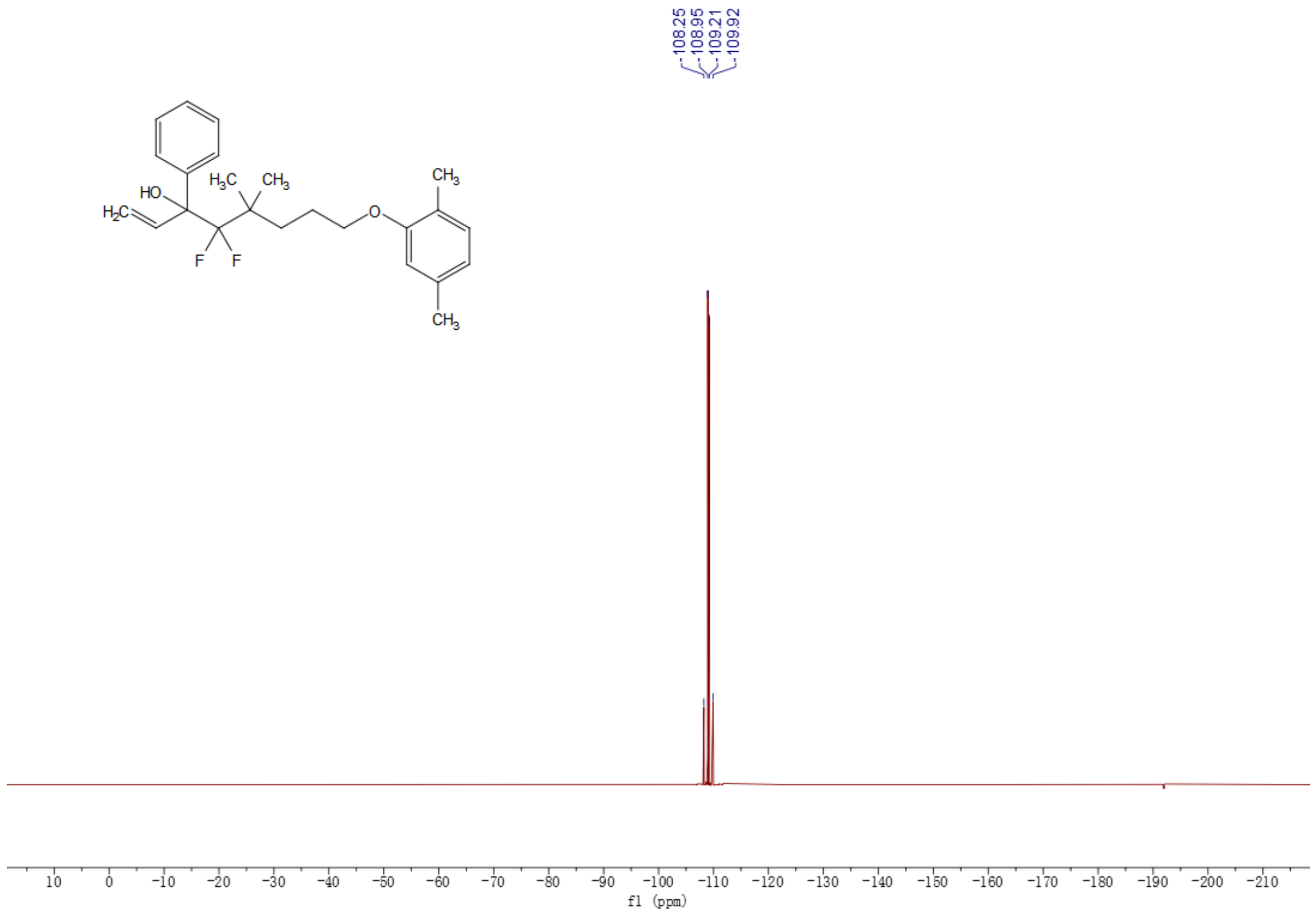

^{13}**C NMR** spectrum (101 MHz, $CDCl_3$) of compound **4b**

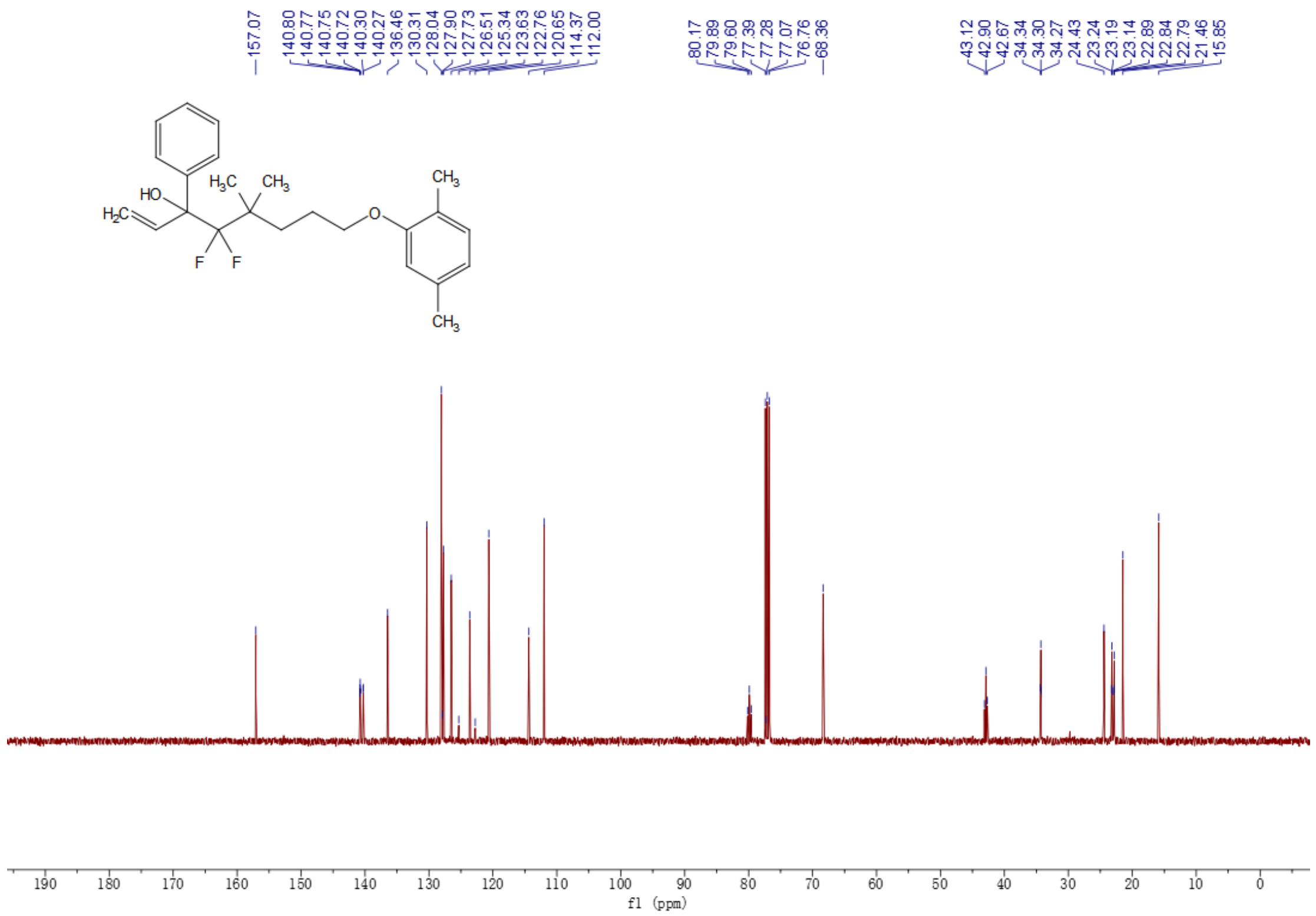

ethyl -8-(2,5-dimethylphenoxy)-4,4-difluoro-5,5-dimethyl-3-phenyloct-2-enoate (4c)

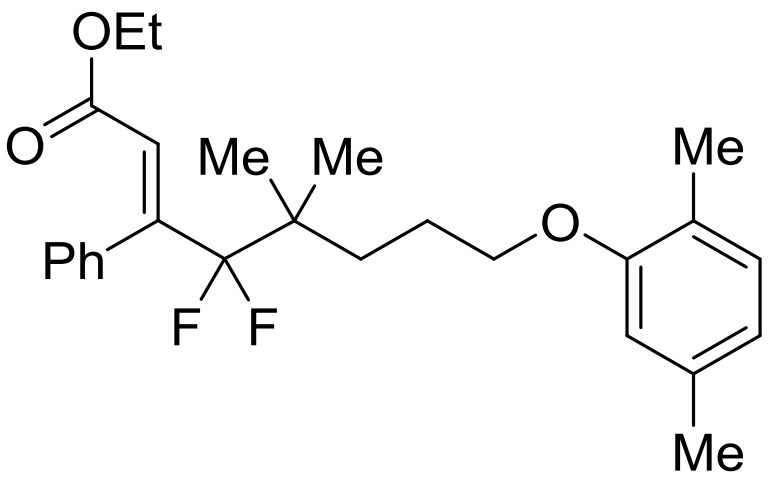

The product (53.4 mg, 62% yield) as colorless oil was purified with silica gel chromatography (petroleum ether/ ethyl acetate = 100/1). **^{1}H NMR** (400 MHz, Chloroform-*d*) δ 7.28-7.19 (m, 5H), 6.92 (d, *J* = 7.6 Hz, 1H), 6.58 (d, *J* = 7.6 Hz, 1H), 6.49 (s, 1H), 6.31 (s, 1H), 3.89 (q, *J* = 6.8 Hz, 2H), 3.73 (t, *J* = 6.4 Hz, 2H), 2.23 (s, 3H), 2.08 (s, 3H), 1.65-1.59 (m, 2H), 1.47-1.43 (m, 2H), 0.93 (t, *J* = 7.2 Hz, 3H), 0.84 (s, 6H). **^{19}F NMR** (376 MHz, Chloroform-*d*) δ -104.5. **^{13}C NMR** (101 MHz, Chloroform-*d*) δ 165.5, 156.9, 148.1 (t, *J* = 4.0 Hz), 136.5, 135.1, 130.3, 129.5, 128.6, 127.8, 124.6 (t, *J* = 10.1 Hz), 124.4 (t, *J* = 253.5 Hz), 123.6, 120.7, 112.0, 68.1, 60.6, 42.1 (t, *J* = 23.2 Hz), 33.5 (t, *J* = 3.0 Hz), 24.2, 21.8 (t, *J* = 4.0 Hz), 21.4, 15.8, 13.8. **HRMS** (EI-TOF) m/z: $[M+Na]^+$ Calcd. for $C_{26}H_{32}F_2O_3Na$ 453.2212; Found 453.2211.

^{1}H NMR spectrum (400 MHz, $CDCl_3$) of compound **4c**

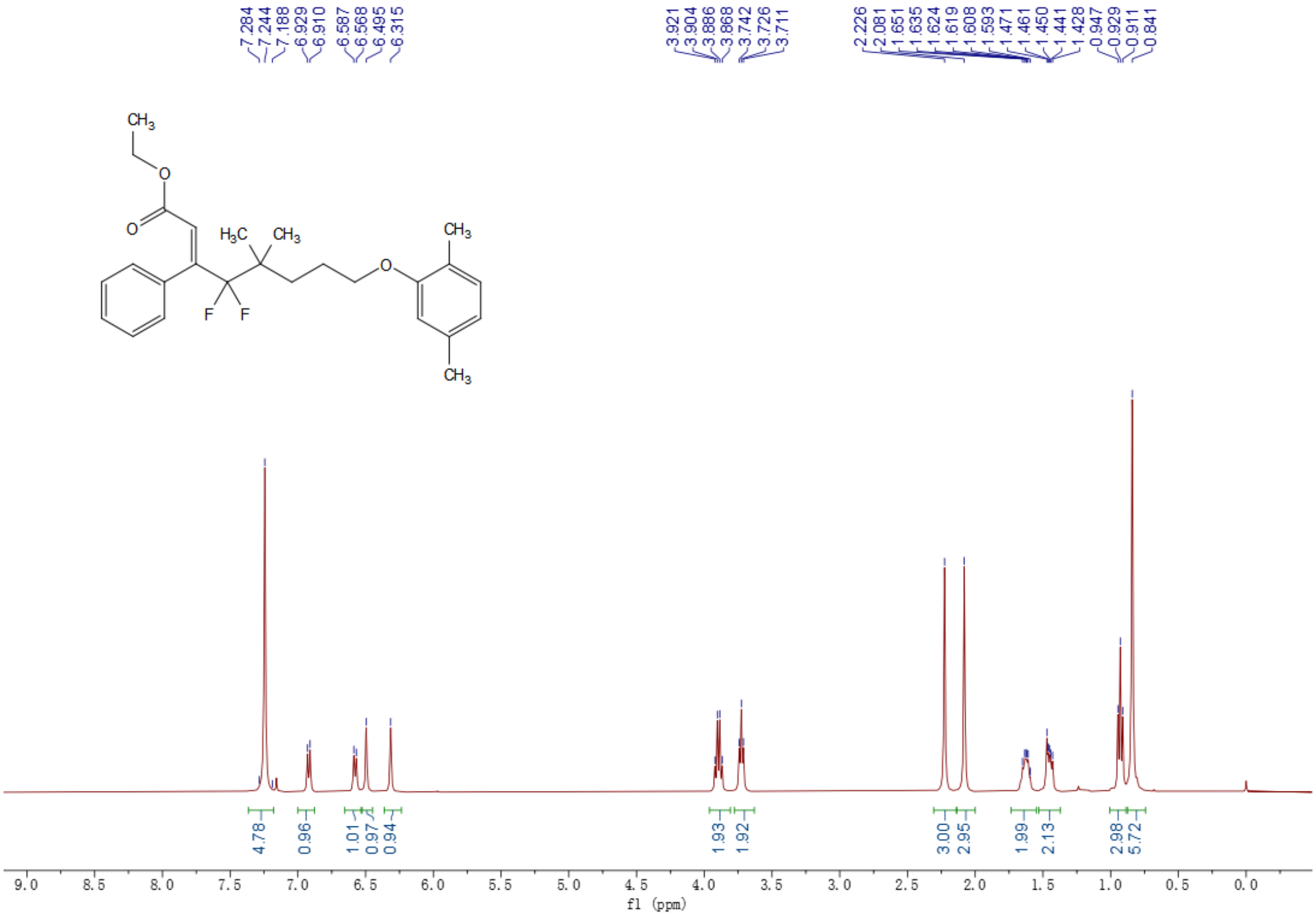

^{19}F NMR spectrum (376 MHz, $CDCl_3$) of compound **4c**

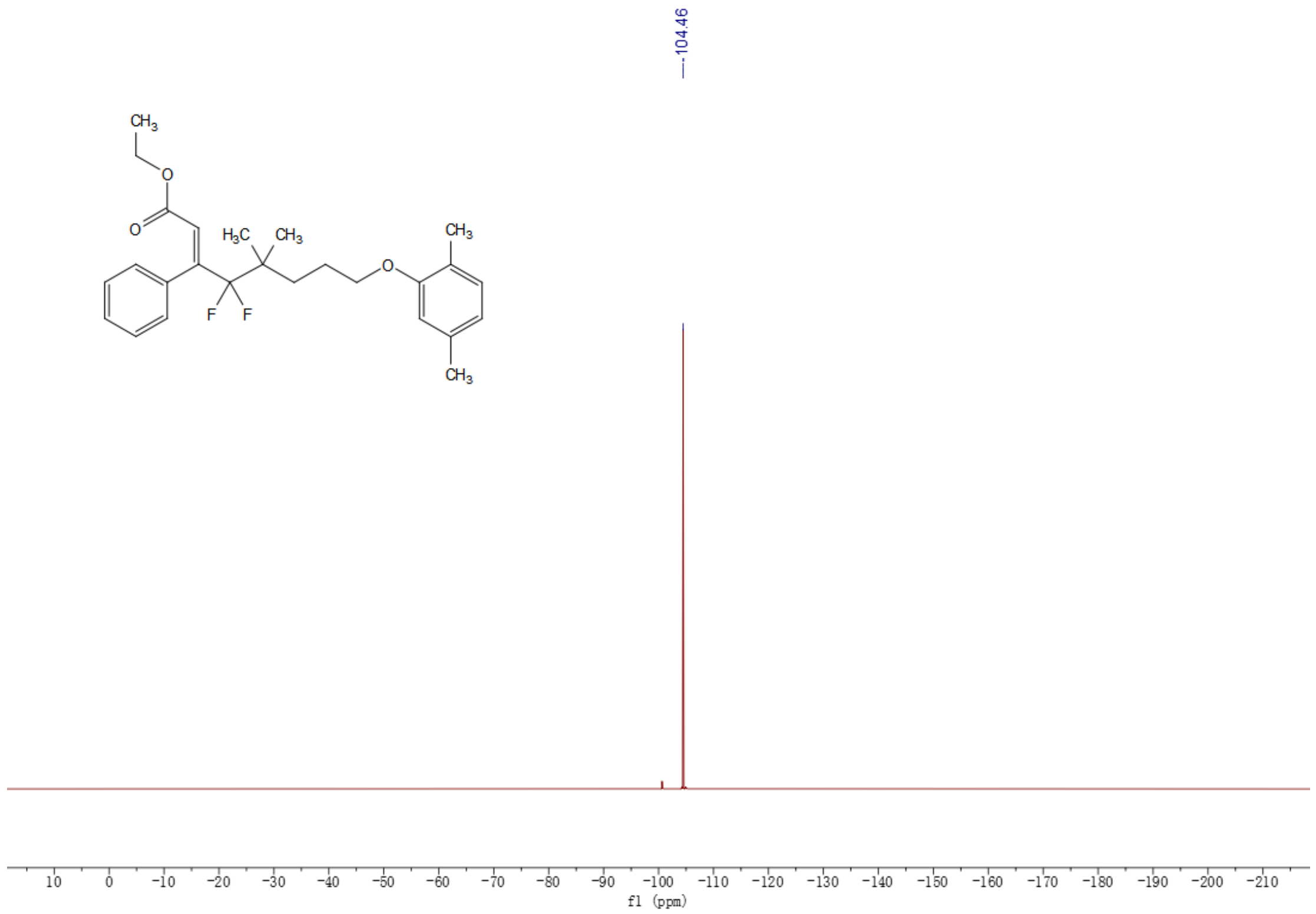

^{13}C NMR spectrum (101 MHz, $CDCl_3$) of compound **4c**

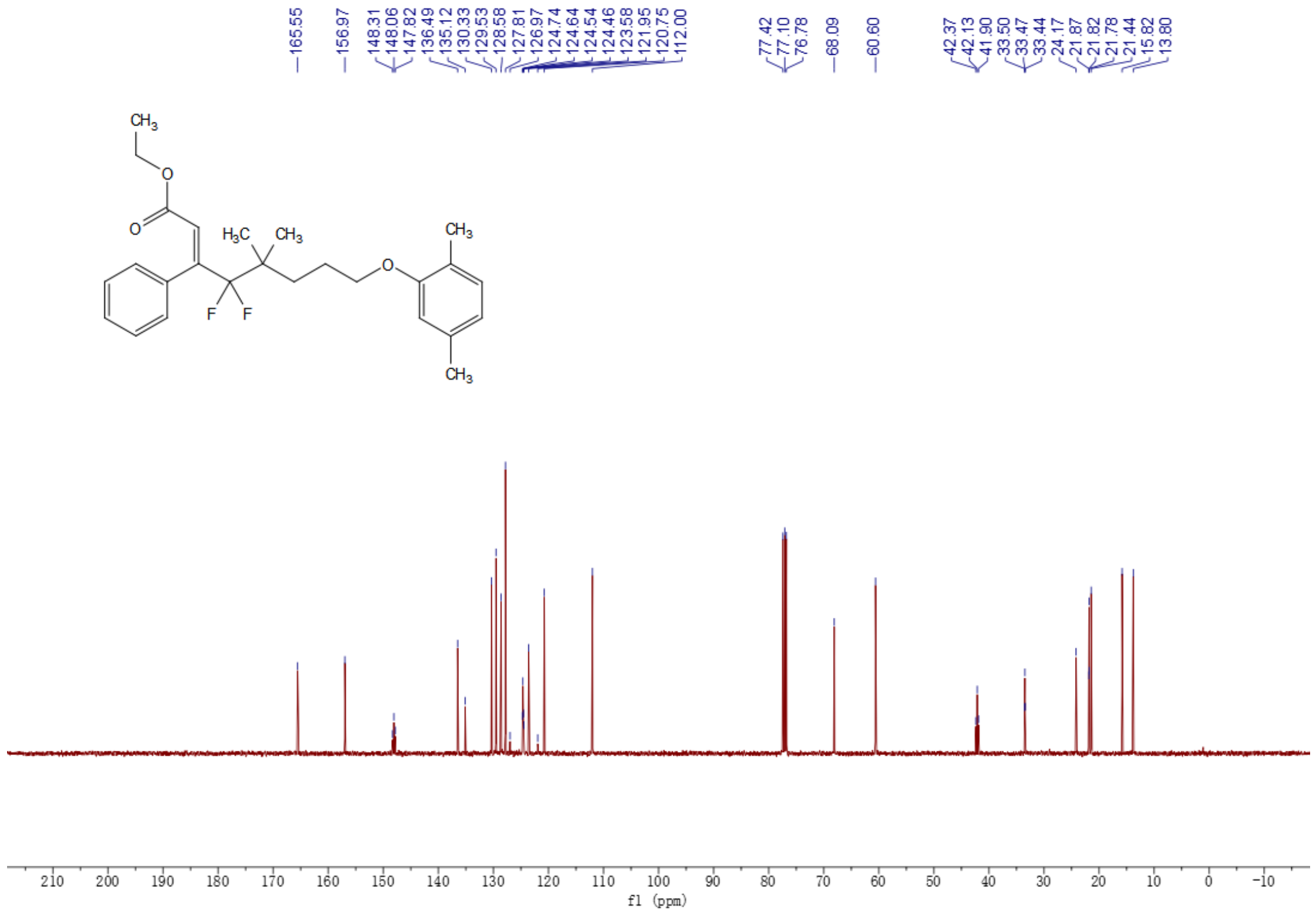

Supplement: Supplementary file 1 [file OPEN-15-e70285-s001.pdf]
